# A Fully Bayesian Approach to Adult Skeletal Age Estimation: Multivariate Latent Trait Modeling With Markov Chain Monte Carlo Sampling

**DOI:** 10.1002/ajpa.70289

**Published:** 2026-06-07

**Authors:** Nils Müller‐Scheeßel, Katharina Fuchs, Christoph Rinne

**Affiliations:** ^1^ Institute for Prehistoric and Protohistoric Archaeology Kiel University Kiel Germany

**Keywords:** age‐at‐death estimation, Bayesian approach, estimation of hazard model parameters, paleodemography, transition analysis

## Abstract

**Objectives:**

This paper investigates the potential of Bayesian transition analysis for improving age‐at‐death estimation in osteological datasets, utilizing the software NIMBLE and JAGS (Just Another Gibbs Sampler). We aim to explore the applicability of this method to different skeletal datasets including different age markers.

**Materials and Methods:**

Transition analysis models estimations of skeletal age markers based on age‐at‐transition and population priors. This study extends the methodology by integrating multiple latent traits into a Bayesian framework. Our probit regression model estimates age‐at‐death and Gompertz distribution parameters directly and without relying on predefined reference populations for age‐at‐transition or population priors. The analysis includes data from three distinct sources, which contain diverse sets of age markers with known age‐at‐death.

**Results:**

The model demonstrates good performance across the datasets and successfully estimates mortality patterns. Sample size does matter, but even smaller samples can still yield useful results. The same is true for the number of traits. A simple multiple‐probit regression will underestimate age ranges, but calibration improves upon this. Even with missing data, our model produces robust estimates.

**Discussion:**

The results highlight the model's effectiveness in improving age‐at‐death estimations and mortality patterns across diverse populations. This Bayesian transition analysis offers a reliable and flexible method for age estimation in bioarchaeological applications on datasets with varying qualities of morphological age indicators. Even compatibility between populations is not mandatory. It is only necessary that there be an observer‐consistent age marker evaluation per skeletal assemblage and that the trait(s) in question change monotonically with biological age.

## Graphical abstract

Ordered probit regression is used as a latent trait model, with age at death estimated from a Gompertz distribution. Combined with Bayesian Markov Chain Monte Carlo sampling, this approach eliminates the need for reference priors for transition ages or population parameters.

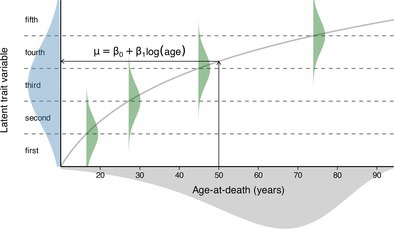

## 1 Introduction

More than 20 years ago, the Rostock Manifesto set the stakes for any estimation method from osteological material: “Osteologists must recognize that what is of interest in paleodemographic research is Pr(*a*|*c*), the probability that the skeletal remains are from a person who died at age *a*, given the evidence concerning *c*, the characteristics of the skeletal remains […] f(*a*), the probability distribution of age‐at‐death (i.e., lifespan) in the target population of interest […] must be estimated *before* Pr(*a*|*c*) can be assessed. That is to say, to calculate Pr(*a*|*c*) it is necessary to first estimate f(*a*), the probability distribution of lifespans in the target population” (Hoppa and Vaupel 2002, 2). In essence, this means that osteologists must understand the mortality patterns or lifespans of the study population *before* they can make any meaningful statements about the probability of an individual having died at a particular age. Thus, the Rostock Manifesto insists that the traditional approach of estimating skeletal age in osteology—assigning morphological variation with specific age ranges derived from reference collections with known age‐at‐death—is seriously flawed.

In this way, the Rostock Manifesto seemingly presents a chicken‐and‐egg conundrum: How can I have the one without the other? How am I supposed to estimate the mortality profile of a given population without having the age‐at‐death estimates of the individuals first? So far, the problem seemed unsolvable, and that is why several workaround approaches were conceived. In our paper, we present a new approach to circumvent this problem, implementing a thoroughly Bayesian workflow.

Since the publication of the Rostock Manifesto, many new methods for better estimates have been proposed, based on other skeletal characteristics or the development of new modeling approaches. However, a general method that does not depend on a reference collection, and that thus fulfills the central claim of the Rostock Manifesto, is still lacking to date. Indeed, one central motivation for the development of the well‐known ABDOU Transition Analysis was the basic assumption that an age‐at‐death distribution of a population *cannot* be calculated before the individual ages at death are known, because archaeological samples are generally too small (Boldsen et al. 2002, 73, 77). However, to the best of our knowledge, this claim has never been tested, and in the present paper we would like to challenge it.

The main steps in traditional Bayesian approaches towards age‐at‐death estimation for a given skeletal assemblage have been summarized as follows (Nikita et al. 2018):

− Evaluate and record stages/grades/phases of the morphological trait or traits according to the method of choice for each skeleton/bone (e.g., Lovejoy and colleagues' auricular surface, phases 1–8)
− Get the conditional distribution of the age‐at‐transition from one score of a given trait to the next as a function of age from a reference sample with known age‐at‐death
− Obtain parameters of the probability distribution (e.g., uniform or Gompertz) for the population's age‐at‐death distribution, for example, from another sample
− Combine the scores of observed traits, the conditional distribution of the traits, and the population parameters into age estimates

In our approach, the last three steps are combined into one Bayesian model with Markov Chain Monte Carlo (MCMC) sampling, whereby the parameters are estimated simultaneously. This has three striking advantages: First, given that the trait morphology changes monotonously in relation to biological age, there is no need for a reference sample. The parameters of the age‐at‐transition from one stage to the next are estimated during calculation from the population under scrutiny. Second, there is no need for specifying an informed population age‐at‐death distribution. The respective parameters are also modeled directly from the population, via the Gompertz distribution. Finally, within the model, age‐at‐death is also estimated simultaneously, with averages and age ranges. We think that by combining the steps mentioned above into one model, our approach comes very close to the demands of the Rostock Manifesto.

## 2 Previous Approaches

The beginning of the modern era of paleodemography can be pin‐pointed quite exactly to the famous paper “Farewell to Paleodemography” by Bocquet‐Appel and Masset (1982). It ensued not only a heated debate but with a lag of some years initiated methodological attempts to overcome the shortcomings outlined by the authors (see Courgeau 2012, 175–182; Konigsberg and Frankenberg 2021, 19–20).

In this regard, our approach owes much to L. W. Konigsberg. Already in 1992, Konigsberg and Frankenberg (1992) insisted on the importance of estimating population parameters. Building on earlier work by especially T. Gage (e. g. Gage and Dyke 1986; Gage 1988), Konigsberg then turned to hazard analysis and probit Bayesian modeling with MCMC (Konigsberg and Holman 1999; Konigsberg and Frankenberg 2002; Konigsberg and Herrmann 2002; Konigsberg et al. 2008; Prince et al. 2008). His OpenBUGS‐code (Konigsberg and Frankenberg 2013, esp. 163–165) was reused by Sasaki and Kondo (2016) which was important for our model. Konigsberg (2015) even developed a multivariate probit regression model. However, his approach estimates neither the Gompertz parameters nor the threshold parameters but takes them, again, as given. His algorithm essentially estimates the same age range (roughly 45–65 years at 50% HDIs) for all individuals (Konigsberg 2015, 376 fig. 7). This probably partly attributes to his choice of morphological traits (i.e., closure of cranial sutures, which are only weakly correlated with age) and the parameters of his simulation, which led to a population with a very low mortality rate (and thus many individuals with fully closed sutures). The R‐scripts Konigsberg provides on his website (http://faculty.las.illinois.edu/lylek/JFS08/JFS08.htm; accessed: 2025/03/23) were used in a number of studies (e. g., Muñoz et al. 2018; Hens and Godde 2022; Godde and Hens 2025). Building on the Konigsberg scripts, Nikita et al. (2018) provide a workflow as a set of R‐scripts to implement transition analyses in the conventional way.

Konigsberg was also one of the authors of what is now mainly understood as transition analysis, as initially laid down by Boldsen et al. (2002), see also already Boldsen (1997). The ABDOU transition analysis is an app that underwent several transitions and subsequently branched into two different approaches: The first, TA3‐ML, uses a random generalized linear model (Milner et al. 2024, 190). Though it is still available online (https://github.com/rer145/ta3 [accessed: 2026/03/06]) the latest update was in January 2021. The second branch, TA3‐TA, is true to the original idea and relies on logistic regression to derive ages‐at‐transition (Milner et al. 2024, 190; Boldsen et al. 2026). It seems to have been released just recently online (https://adbou.dk/transition‐analysis/ [accessed: 2026/03/04]). One of the central aims of the TA‐team was the incorporation of more traits and traits that better correlate with age (Milner and Boldsen 2012; Getz 2020; Galimany and Getz 2022; Boldsen et al. 2026). Apparently older versions of ABDOU transition analysis were criticized recently because the population parameters differ significantly from the ones calculated from known age‐at‐death and from those derived from other methods (Clark et al. 2020; Simon and Hubbe 2024). Overall, transition analysis is still relatively rarely applied (Clark et al. 2023, 157).

A different approach was adopted by the French school (Caussinus and Courgeau 2010; Courgeau 2012; Séguy et al. 2013; Łukasik et al. 2021). They also chose a Bayesian approach with MCMC sampling but decided to conceive of both age and traits as categorical data, not as continuous data as it is done here. They argued that a “parametric […] model […] may fail to capture past situations where these models were not verified” (Courgeau 2012, 181f.). Consequently, they used Dirichlet distributions for both age and traits with priors derived from reference populations. We see no advantages in conceiving the data as categorical as it makes it more difficult to follow the analysis and the results. Furthermore, it seems that a multivariate solution does not exist, which forced Łukasik et al. (2021, 600) to resort to a complicated pre‐arrangement of the osteological data in their analysis.

An approach that deliberately deviates from the “Rostock Manifesto” was presented by Hens and Godde (2020). They implemented a Bayesian Multiple Linear Regression (BMLR) using rstanarm in the study of palatal suture fusion. This involved treating age as dependent variable which reverses the logic of transition analysis. They argued that this approach better fits the behavior of the aging variables which obliterate in a manner that cannot be properly grasped by transition analysis. While the prior for the Bayesian regression analysis was weak, this approach still needs a training dataset.

A very different turn on age estimation is taken with seriation of an age marker (Lovejoy et al. 1985, 3). Gilmore and Grote (2012) modified the Miles method of tooth wear by looking at the whole population under scrutiny and determining an average wear rate which then is used to calculate individual ages. Building on Cave and Oxenham (2016) who matched seriated wear rates to a population with known age distribution, McFadden et al. (2019) present regression formulas for population percentages per age‐at‐death interval. These formulas derive from modern high mortality populations and the ratio of the below 15 years‐old‐individuals to all individuals of a population. The idea is to thus distribute individuals from a population seriated according to some age‐related trait percentage‐wise.

A new application for subadults was recently proposed by Stull et al. (2023). They provide a flexible array of functions and a complex set of test statistics to decide which models perform best for which specific trait, and argue for different transformations of age depending on trait. Most notably, they allow the concurrent use of both ordinal and continuous response variables. However, Stull et al. (2023), too, use a fixed age distribution as prior and known conditional trait responses. Thus, the authors themselves concede that their algorithm is still only “semi‐Bayesian” in practice (Stull et al. 2022, 752).

Also recently, random forest regression (RFR) was added as a further method to estimate the age of adults (McCormick 2023). While RFR is doubtlessly vastly superior over simple linear regression, it still depends on a training data‐set, thus a reference population. The same is true for the very sophisticated method introduced by Navega et al. (2022). They solved the problem of age estimation with the help of deep random neural networks. The software is available online. The authors put together a training set with 64 age‐related traits that also formed the basis for one of our case studies. Still, their algorithm also depends on a reference sample.

**FIGURE 1:**
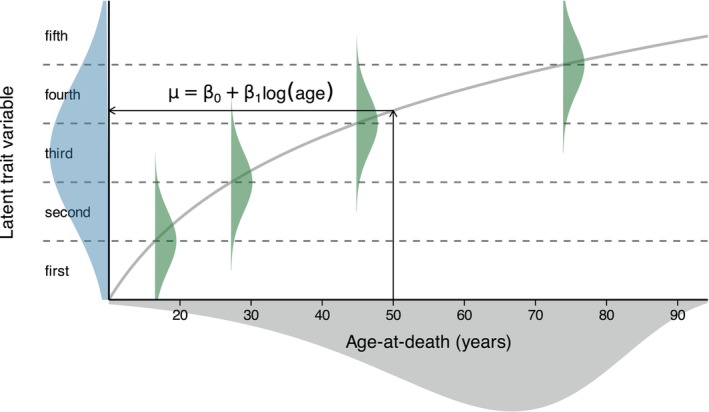
Schematic visualization of the ordered probit regression as latent trait approach with age‐at‐death estimated from a Gompertz distribution (gray area). The green Gaussian distributions symbolize the transitions between the trait stages (here, arbitrarily, 5 stages, and therefore 4 transitions). The regression line with its parameters intercept ($\beta_{0}$) and slope ($\beta_{1}$) determines the position of the latent trait variable. Please note that age is scaled logarithmically and that the line is therefore bending (cf. Konigsberg 2015, 373 fig. 4). In real‐life applications, the latent trait variable does not need to be centered as it is here and the transitions will almost certainly not be as regularly spaced as they are here.

**FIGURE 2:**
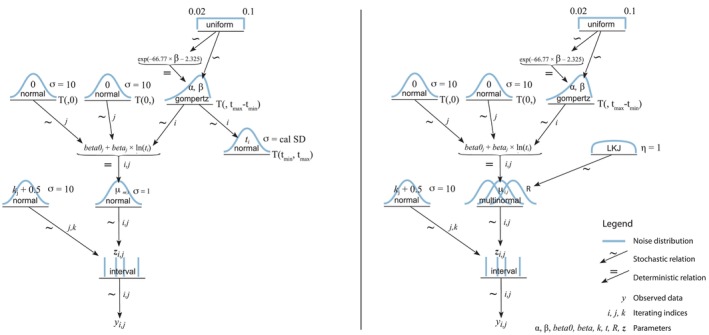
Multiple‐normal (left) and multivariate normal (right) ordered probit regression model of the Bayesian approach to adult skeletal age estimation, following the conventions of J. Kruschke (2015); see (Campitelli and Macbeth 2014 for a discussion of this type of display).

Navitainuck et al. (2024) outline an approach consisting of six steps how to arrive at posterior age estimates. In practice, it shares the basic principles with transitional analysis but adds the estimation of parameters for describing the distribution of the population analyzed. Thus, their approach already takes a step into the direction we propose in the present paper and we therefore look at it more closely. For four of the methods their algorithm yielded identical ages for the individuals (Navitainuck et al. 2024, 8 fig. 2). Overall, they computed very low mean ages and age ranges for the individuals, much lower than that by traditional methodology (ibid. 16). Accordingly, what they present as population densities or age distributions is not plausible from a biological point of view as only a few allow individuals of an age more than 45 years. The biological impossibility is even more the case where traits have been combined (ibid. 11 fig. 5, truncated spline or truncated Gompertz‐Makeham). It seems that the algorithm tried to find a value where the difference between the individuals is minimized, given the overlapping in age of the trait expression. This would at least explain why the ages were compressed instead of spread across the probability space. For example, for the pubic symphysis after Brooks and Suchey, for which all trait expressions are present (Navitainuck et al. 2024, 7 fig. 1), the age distribution shows a marked peak at 35 years and already approaches zero at 45 years, thus negates the possibility that there are individuals older than that. However, according to their Supporting Information 6, the estimation hinged on a grid of 1–100 years. Therefore, from a logical point of view, if the highest score has been recorded at least once, 100 years *must* be in the range of the probability distribution for this population.

In practice, all approaches building on the Konigsberg scripts or ABDOU transition analysis are single or multiple‐probit (or logit) regressions, but they take the ages‐at‐transition and the population priors as given. Most of the other newer methods numbered above also depend on reference samples. So, while most of them claim to be “Bayesian” in character because they make use of Bayes's theorem, the French school does not regard them as fully Bayesian: “the unknown parameters are always assumed to be fixed, whereas a Bayesian method will assume them to be random” (Courgeau 2012, 259). Approaches which take the whole age structure of the population under scrutiny into account either lack validation studies (seriating approaches by Gilmore and Grote 2012 and McFadden et al. 2019) or produce unconvincing results (Navitainuck et al. 2024). Our method builds on previous research by combining probit regression with seriation and an empirically grounded general model of human mortality, the Gompertz distribution and thus fulfills the French demand that the parameters should all be random.

## 3 Methods

### 3.1 Ordered Probit Regression as Latent Trait Approach

The Bayesian approach presented here is based on univariate (one trait) or multivariate (two or more traits) ordered (or ordinal) probit regression (Figure 1). The foundations for that have been discussed extensively in the past (e.g., Konigsberg 2015; see also above on previous approaches). Each trait, consisting of ordinal categories, is thought of as representing a latent continuous variable, or, to formulate it differently, it is assumed that the individual values of the categories are controlled by an underlying (i.e., latent) process. This process, in turn, is supposed to be of a Gaussian nature, and each level of the trait covers one specific part of the ensuing cumulative (i.e., probit) curve. This implies that the underlying process is necessarily non‐decreasing. The transitions from one level to the next—the thresholds—are not fixed but are also modeled with a Gaussian normal distribution each. The covariate “age‐at‐death” in our application is also not fixed but is modeled by a Gompertz distribution.

In the following, we explain the parameters of our models in more detail. The multiple‐normal model assumes conditional independence of the traits (Figure 2, left) while the multivariate normal (Figure 2, right) takes correlations between the traits into account. Still, both models share most of their components, the differences are highlighted below.

The observed data are written as *y*_*i,j*_ where *i* indexes individuals from 1 to N and *j* indexes traits (skeletal age “indicators”) from 1 to J. Because the data are recorded for ordinal categorical traits the values for each trait are in the form of positive integers. Traits can be in the form of binaries, such as “open” versus “closed” for a crude scoring of suture closure (scored as 1 and 2). Individual traits may also vary across studies because of “collapsed” scores, as for example in the original Todd (1920) phases for the pubic symphysis with scores 1–10 versus the T2 scores in Katz and Suchey (1986) with scores from 1 to 6. Or new scores may be added such as Berg's (2008) addition of a score 7 to the Suchey‐Brooks (Brooks and Suchey 1990) 6‐score‐system. We use *k*_*j*_ to indicate the positive integer scores within a given scoring system where the number of scores within trait *j* is *K*_*j*_.

$$i=1,\ldots ,N,j=1,\ldots ,J,k=1,\ldots ,K_{j}.$$

In presenting the MCMC model nodes with stochastic relations are written with a tilde (“∼”) for “distributed as” and nodes with deterministic relations are written with an equal sign. The Gompertz model has two parameters *α* and *β*, where *α* is the log hazard at age zero (adjusted to the minimum “adult” age, generally 15 years) and *β* is the slope of the log hazard against age.

The prior for the *β* parameter is:

(1) $$\beta ∼U\left(a=0.02,b=0.1\right)$$

where *U(a, b)* is a uniform distribution with the density function having a value of *1/(b–a)* between *a* and *b*. The range of *a* to *b* for *β* is approximately equal to that from Sasaki and Kondo (2016) for 134 lifetables, where the minimum value is 0.025 for Chile in 1909 and the maximum value is 0.1061 for Albania in 2011. The *α* parameter is given as:

(2) $$\alpha =\exp\left(-66.77\times \beta -2.325\right)$$

where −66.77 and −2.325 are from Figure 1 in Sasaki and Kondo (2016, 529). Taking the logarithms of both sides of Equation (2) gives a linear equation around which Sasaki and Kondo (2016) give an error variance. We have ignored this term as its inclusion leads to numerical instability in our fully Bayesian model. As a consequence of treating the *α* term as a linear transform of *β* in the log scale, our Gompertz model has a single parameter (*β*).

The prior for individual ages, which we write as *t*_*i*_, where *t* is the time of death (age‐at‐death), is:

(3) $$t_{i}∼\text{Gompertz}\left(\alpha ,\beta ,a=15,b=100\right)$$

where Gompertz (*α, β*) represents the probability density function for

$$f\left(t|\alpha ,\beta \right)=\alpha \times \exp\left(\beta \times t\right)\times \exp\left(-\frac{\alpha }{\beta }\left(e^{\beta \times t}-1\right)\right)$$

starting at zero and truncated at a maximum of 100 years—minimum age. In some examples we use a higher minimum age than 15 years. In the paleodemographic setting the appropriate minimum age is that age at which all “adult” skeletons will have reached completion of a particular indicator.

As previously mentioned, the symbol *j* indexes the ordinal categorical traits (age “indicators”) with *j* = 1, …, *J*. For example, in the Buckberry and Chamberlain (2002) auricular surface method *J* = 5. The number of states per trait is given as *K*_*j*_. In the original Buckberry and Chamberlain scoring system *K*_*1*_ = 5, *K*_*2*_ = 5, *K*_*3*_ = 3, *K*_*4*_ = 3 and *K*_*5*_ = 3, so for example *k*_*1*_ = 1, …, 5 while *k*_*3*_ = 1, …, 3. With *K*_*j*_ states per trait *j* there are *K*_*j*_ − 1 thresholds for each trait. The thresholds within each trait are represented using the symbol *γ*, so that for the first trait within Buckberry and Chamberlain's scoring system the thresholds are $\gamma_{k_{1}=1}<\gamma_{k_{1}=2}<\gamma_{k_{1}=3}<\gamma_{k_{1}=4}$. So that the model is identifiable, all $\gamma_{k_{j}=1}=1.5$ (Kruschke 2015, 677).

The priors within each trait *j* for the thresholds with more than two states are:

(4) $$\gamma_{k=2,\ldots ,K_{j}}∼\mathrm{TN}\left(\mu =k+0.5,\sigma =10,a,b=\infty \right)$$

where TN (·) is a truncated normal distribution with left truncation at *a* and right truncation at *b*. Because the thresholds must be ordered, for the JAGS‐model, *a* = 1.5, and the simulated thresholds from Equation (4) are sorted in ascending order. NIMBLE does not have a sort‐function, so there $a=\gamma_{k-1}$.

For each individual there is a vector of latent traits **z**_*i*_ of length *J* (number of traits). For the simple probit model with conditional independence, the elements of these vectors are modeled separately with normal distributions:

(5a) $$z_{i,j}∼N\left(\mu_{i,j},\sigma =1\right)$$

For identifiability, *σ* is fixed at unity (1).

For the complex probit model, these vectors have a multivariate normal distribution:

(5b) $$z_{i}∼\mathrm{MVN}\left(\mu_{i,j},R\right)$$

where there is a “flat” prior by Lewandowski et al. (2009) for the correlation matrix:

(6) $$R∼\mathrm{LKJ}\left(\eta =1\right)$$

For *η* = 1, this prior is uniform over the space of correlation matrices.

The terms in the vector **μ**_*i*_ are:

(7) $$\mu_{i,j}={\text{beta}}_{0_{j}}+{\text{beta}}_{j}\times \ln\left(t_{i}\right)$$

with the priors for the *beta*_*0*_ *and beta* parameters within each trait *j* being:

$${\text{beta}}_{0_{j}}∼\mathrm{TN}\left(\mu =0,\sigma =10,a=-\infty ,b=0\right),$$

(8) $${\text{beta}}_{j}∼\mathrm{TN}\left(\mu =0,\sigma =10,a=0,b=\infty \right)$$

JAGS and NIMBLE both have a statement dinterval that can be used in an “as distributed” setting but which is difficult to write in a succinct format. We write this function as:

(9) $$\begin{array}{c} y_{i,j}=\left\{\begin{array}{cc} 1 & \text{if}\;z_{i,j}\leq 1.5,\\ k_{j} & \text{if}\;\gamma_{j,k_{j}-1}<z_{i,j}\leq \gamma_{j,k_{j}},k_{j}=2,\ldots ,K_{j}-1\\ K_{j} & \text{if}\;\gamma_{j,K_{j}-1}<z_{i,j} \end{array}\right. \end{array}$$

Because dinterval indexes categories from zero instead of one, the observed trait levels and thresholds are internally shifted by one unit.

The proper initialisation of the chains is of eminent importance in probit regression. Each chain starts with different parameter values to enhance mixing, and most parameters pick random values from uniform distributions. For Gompertz *β* the init value is in the range 0.02–0.1, for *beta* the range is 0.5–1, and for *beta*_*0*_–10–3. The init value for age (*t*_*i*_) is 20–40, assuming that the starting age is 0. The correlation matrix is for all chains initialized with an identity matrix (ones in the diagonal and zeros otherwise). The thresholds $k_{j=2,\ldots ,K_{j}}$ are initialized with (*k* + 0.5) and the vector of latent traits **z**_*i*_ with ($y_{i,j}-U\left(-0.2,0.2\right)$).

With one of the datasets, we also test the effect of missing data. The missing data is imputed automatically within the simple multiple ordinal regression based on the other observed values and the likelihood (Plummer 2017, 15).

### 3.2 The Issue of Conditional Independence

Most methods have circumvented the mathematical problems that ensue when the multivariate normal and thus covariation and correlation matrices are involved by assuming conditional independence between traits. This assumption is obviously unwarranted and will lead to shrinkage of the resulting age estimate. Thus, the age range is narrower than it should be, and this in turn leads to lower coverage than claimed by the age range.

Already Boldsen et al. (2002) tried to deal with the problem by shifting the probability density by a fixed amount so that it again covers the proposed range. This method was further refined by other authors (Fieuws et al. 2016; Sgheiza 2022) but the solutions proposed remain “ad hoc”. The problem with the approximation is that it is far from certain that the posterior predictive distribution will be of Gaussian shape as assumed by this approach (Fieuws et al. 2016, 492). Navega et al. (2022, 12) add a regression uncertainty model to their calculations, and the range of the estimates is conditioned on the standard deviation of this model.

Essentially, these solutions calibrate the posterior estimations. For the Bayesian approach, Gelman et al. (2023, 1) go as far as stating that “posterior predictive recalibration cannot work in general”, as this tends to understate uncertainty and will pull the posterior estimates away from the prior (Gelman et al. 2023, 6).

A different approach targets the prior predictive distribution, so the definition of the model itself. Unfortunately, simply adding a random effect per individual as proposed by Thevissen et al. (2010, 40–41) is no solution either as random effects per individual and per observation “win” against the non‐deterministic relationship of age. Konigsberg (2015) included the correlation matrix for the correlation between traits into his calculations but this presupposes, of course, that the correlation is known and universally fixed. This is also true for Stull et al. (2023) who also use a multivariate normal with covariance matrix to model the mutual dependency of variables. To cut down time, however, they group variables with the same correlation coefficients to reduce the number of terms in the calculation. Furthermore, they, too, use a fixed age distribution as prior and known conditional trait responses.

Our multivariate model should take care of this issue and estimate approximately correct coverage rates. However, for incomplete cases or for cases where a multivariate model would take too long to run, it might be necessary to run the simple multiple‐normal model. If used with more than one trait, such a model will underestimate age because it is not taking into account the correlation between the predicting latent variables. Therefore, we opted for adding “white noise”—a Gaussian normal distribution prior—to the estimation of age in the “simple” multiple ordinal regression. The essential element of this “white noise” is the amount of spread as defined by σ which calibrates the uncertainty. Per definition of calibration, this cannot be taken from the data but incorporates an element of subjectivity. Here, we take the uncertainty of the model from the literature.

### 3.3 Further Modeling Decisions

While a logit likelihood would be equally feasible and has, indeed, been employed in the past (e.g., Boldsen et al. 2002; Thevissen et al. 2010), the differences between logit and probit are negligible, and the choice for one or the other is mostly attributed to different historical trajectories within the disciplines. For bioarchaeology, probit regression has been the method of choice for most researchers, so we decided to stick to it as well. Given the large errors associated with age‐at‐death estimation, it is very doubtful whether the specific modeling approach (probit vs. logit) makes any difference at all (Konigsberg 2015, 369). The probit model has the additional benefit that there is a multivariate variant (multinormal), which is lacking for the logit (ibid.); for the logit, it has to be approximated by the multi‐t‐distribution (Hirk et al. 2019).

Central for the model is the Gompertz distribution for age‐at‐death estimates where its second parameter (α) is conditioned on its first (β), due to the established high correlation between the two (Müller‐Scheeßel et al. 2024; Sasaki and Kondo 2016). In this context, we would like to challenge the assumption by Navitainuck et al. (2024) that the population probability distribution necessarily has to have three parameters. In light of the large uncertainties in the resulting age ranges, such models seem to offer few benefits and would only introduce volatility in the model. We think that one strength of our Gompertzian model is its simplicity, and that the broad calculated age ranges make sure that the deterministic linkage of the Gompertz parameters does not superimpose too strict a model.

We follow the common approach to take the logarithm of age, which allows for more variability in higher stages (Konigsberg et al. 2016, 568). Furthermore, because in our model the thresholds that determine the age estimates are free to float, larger gaps between thresholds equal broader age estimates. Thus, our model accounts for heteroscedasticity.

One important aspect of ordered probit regression is the problem of identifiability (Chib and Greenberg 1998). This issue arises because the ordinal response is, by definition, without scale; a latent variable with normal distribution thus needs anchor points. Therefore, in ordered probit regression, at least two of the parameters intercept and standard deviation or thresholds have to be fixed (Jeliazkov et al. 2008). To ensure identifiability of the multivariate model, we use a correlation matrix instead of a covariance matrix, because a correlation matrix effectively limits the covariance at unity, and we fix the first threshold at the arbitrary value of 1.5, following the convention of Kruschke (2015, 677). As prior for the correlation matrix of the multivariate normal distribution, we decided to use the LKJ distribution (Lewandowski et al. 2009) with Cholesky decomposition. In the “simple” ordered probit model, the standard deviation of the latent variable is fixed at unity (= 1) and the first threshold is, again, fixed at 1.5.

To run the models, we use R v. 4.3.1 (R Core Team 2023) and either the NIMBLE framework, for the multivariate model, or JAGS, with the simple model. NIMBLE also follows the BUGS/JAGS script language but precompiles the models for speed (de Valpine et al. 2017). We use JAGS along with the rjags (Plummer et al. 2022) and runjags (Denwood 2016) packages to interface with JAGS (Plummer 2003), which utilizes the Gibbs algorithm to generate Monte Carlo Markov Chains (MCMC). The results of both pipelines are processed with the coda package (Plummer et al. 2006). Each model is executed with three (JAGS) or four (NIMBLE) chains and continues for as many steps as needed to meet the required quality thresholds. Chain thinning is applied as minimally as possible. The quality of the resulting models is primarily evaluated using two indicators: (1) the potential scale reduction factor (PSRF), which measures chain convergence and should be below 1.1 (Kruschke 2015, 181), and (2) the effective sample size (ESS), which controls for autocorrelation and ideally should be at least 10,000 (Kruschke 2015, 184).

**FIGURE 3:**
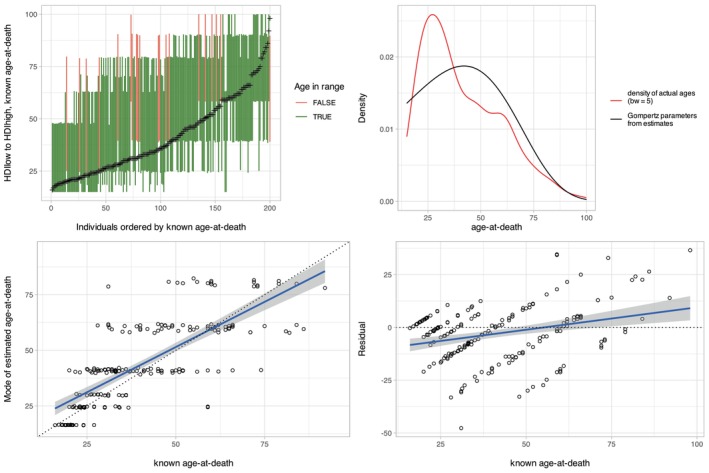
Estimation of age‐at‐death for dataset “Allmales” (*n* = 200, pubic symphysis after Brooks and Suchey 1990). Top left—comparison between known age‐at‐death and the estimated 95% HDIs, colored green if the true age lies within the range and red if not; top right—density of known age‐at‐death and Gompertz function with estimated parameters; bottom left—known age‐at‐death versus mode of estimated age‐at‐death, with the dotted line marking complete equivalence and the regression line shown in blue; bottom right—known age‐at‐death versus residuals (known age‐at‐death minus mode of estimated age‐at‐death), with the dotted line marking complete equivalence and the regression line (“residual age slope” in Table 1) shown in blue.

If a probability distribution truly follows a Gaussian function, then the average measures mode, arithmetic mean and median are identical. The same is true for the highest density interval (HDI, 95% = 2.5–97.5 percentiles) compared with an equal‐tailed interval. However, the latter will represent skewed posterior distributions less precisely than will HDIs (Kruschke 2015, 342–343), as will the arithmetic mean and median in relation to the mode. In our view, there are good arguments to use the mode as the most meaningful point estimate (Kruschke 2015, 206) and the HDI as credible interval. In the following, we always refer to the mode if point estimates are addressed. The only exceptions are the Gompertz parameters. The empirical deterministic link between α and β is only preserved with the arithmetic mean, so the Gompertz function in Figure 3, for example, is constructed with the means, not the modes of α and β.

In fact, in the literature, so far there seems to be little consideration that there are different possibilities for how to express the point estimate from individual estimation ranges. While some use the mode (Ferrante et al. 2015, 1785), others (McCormick 2023, 276) use the mean, without further justification. In the Supplement, we show that the choice of how to express the point estimate has a profound effect on some age estimation quality criteria, while it leaves others unaffected, due to the fact that they are based on the full probability range.

### 3.4 Goodness‐Of‐Fit Measures

As Stull et al. (2022, 752) rightly point out, there is still no consensus on how to express the quality of the relationship between true and estimated age best. To make things even worse, the same indicator is labeled differently by different authors and different measures are named the same by others. Navega et al. (2022, 14) name four attributes for assessing the quality of age estimates: “An age‐at‐death prediction model […] should be accurate, unbiased, valid, and efficient”.

Accuracy is the difference between estimated and true age. There are several ways to measure this difference. We term the *mean absolute error* between estimated and true age “inaccuracy”. An alternative way of measuring accuracy is the *root mean squared error* (RMSE) between estimated and true age. Stull et al. (2022, 752) introduced the *test mean negative log posterior* (TMNLP) as a single metric to measure the quality of the agreement between the observed variable, that is, the known age, and the simulated expected outcome. The TMNLP calculates the negative averaged log probability at the point of observation (= known age‐at‐death). We add here the *continuous ranked probability score* (CRSP) (Gneiting and Raftery 2007, 366–367) which is also based on the posterior probability density. However, whereas the TMNLP is a strictly local measure, as it measures the probability density only at the point of known age, the CRSP is a global measure, which takes into account the whole structure of the probability density (Bosse et al. 2022, 9). In both cases, TMNLP and CRSP are to be interpreted similarly to “inaccuracy” or RMSE, so that lower is better.

*Residual age slope* concerns systematic over‐ or underestimation, assessed by the slope of the regression line of the residuals, the difference between estimated and true age‐at‐death. We add *Bias* as the mean deviation of estimated age from true age‐at‐death. The Pearson correlation coefficient measures the correlation between these two values.

An estimate is valid when the estimated age is within the predictive interval for a given level of uncertainty. The percentage of correctly classified individuals within this level (e.g., 95%) has been termed “realized coverage” (e.g., Konigsberg 2015). Coverage is tested with the exact binomial test to see whether the difference between observed and expected cases is significant (ibid. 374).

Finally, efficiency is the width of the prediction interval, which should be as narrow as possible (while still being valid).

## 4 Material

We test the Baysian models with three datasets—one univariate and two multivariate. The first is the dataset of only male individuals (“Allmales”) with known age‐at‐death used by Konigsberg et al. (2008). It consists of five different sub‐samples (the Los Angeles Coroner's Office, the Terry Anatomical Collection, the U.S. Korean War Dead, Balkan genocide victims, and the University of Chiang Mai Anatomical Collection; for details, see Konigsberg et al. 2008; data collection from the Terry Anatomical Collection and from pubic symphyseal casts of the Korean War Dead supported by the National Science Foundation under grant BCS97‐27386 awarded to Lyle W. Konigsberg). The dataset contains the known age‐at‐death of 1766 individuals and information on the six Suchey–Brooks stages of the pubic symphysis and is made available on the homepage of L. Konigsberg (http://faculty.las.illinois.edu/lylek/JFS08/JFS08.htm; accessed: 2024/08/30). Because of the structure of the dataset comprising mainly (very young) war dead and genocide victims, it more closely resembles a “catastrophic” than an “attritional” population (see Margerison and Knüsel 2002 for this distinction) and is therefore unlikely to follow a Gompertz distribution strictly. To reduce computational time and data storage requirements, we sampled 200, 100, 50, 25, and 10 individuals randomly (without replacement) from the complete dataset of 1766. This is meant to demonstrate the effect of different sample sizes on the estimates. Furthermore, we also cover the case of lower mortality, by sampling from the “Allmales” dataset with Gompertz β = 0.05. This is mainly to see how the Goodness‐of‐fit measures change when the data is more strictly Gompertzian.

Thankfully, Navega et al. (2022) made the data they used to develop and test their method of Deep Random Neural Networks (DRNNAGE; funded by Fundação para a Ciência e Tecnologia, grant number SFRH/BD/99676/2014) available (https://github.com/dsnavega/DRNNAGE/blob/master/data/CAMSAD.rda; accessed: 2024/08/30), and we use this dataset for our second case study. The dataset consists of a structured sample of 250 male and 250 female individuals from the Coimbra Identified Skeletal Collection (CISC) and the 21st Century Identified Skeletal Collection (XXI‐ISC), both hosted at the Department of Life Sciences at the University of Coimbra, Portugal. The individuals were selected so that the age‐at‐death distribution of the final sample was homogenous and as uniform as possible. Similar to how we proceeded with the first example, from the original dataset of 500 individuals, we selected 200 individuals (without replacement) in such a way that these individuals resembled a “natural” population following a Gompertz function with β = 0.035. The CAMSAD dataset comprises 64 traits; 26 of them have three stages, 38 have only two. Starting from five traits, we subsequently increased the number of traits randomly in steps of 5, 10, 15, 25, and 40 to the full number of 64 traits to follow the changes in the quality criteria. In contrast to the other datasets, which start at 15 years, the CAMSAD dataset starts at 19 years. Therefore, for the CAMSAD dataset, the minimum age is 19.

Because computation time was too long for the full dataset, we fall back on “simple” multiple ordered probit regression. For the maximum of 64 traits, we therefore apply calibration by adding Gaussian noise, with a standard deviation of 7.5 years, to the age estimate. This value is determined as follows: for the complete CAMSAD dataset, the 95% interval is 30.057 (Navega et al. 2022, 17 table 4). Because 95% roughly equals two standard deviations (which would be 94.6%), a likely candidate for an appropriate calibration interval is 1 sigma = +/−7.5 (30 divided by 2 [one half of the range] and again by 2 standard deviations).

Third, we use a real‐life dataset, the skeletal population of the crypt from Christ Church, Spitalfields, London (Buckberry and Chamberlain 2002; Molleson and Cox 1993). Buckberry and Chamberlain (2002; grant sponsors: Universities of Sheffield, York, and Leeds) revised method of age estimation, via the auricular surface of the illium, decomposes the structure and appearance of the auricular surface into five traits (TO, transverse organization; ST, surface texture; MI, micro‐porosity; MA, macroporosity; AP, apical changes), which are separated into five stages (first two traits) and three stages (last three traits), respectively. The appendix of Buckberry and Chamberlain (2002) contains the known age‐at‐death as well as the stages of the five traits.

The raw Spitalfields dataset was run with the multivariate normal model. However, we also test for incomplete data by removing, successively, 20, 30, 40 and 50 per‐cent of the trait entries, to mirror the likely rates of missing data in real‐life osteoarchaeological collections. Because incomplete nodes are not allowed in multivariate models (see above), again we used the “simple” multiple ordered probit model.

The three datasets differ markedly with regard to the complexity and interpretive power of the morphological age indicators. The 64 traits of the CAMSAD dataset (from the cranial and palatal sutures, clavicle, first rib, pubic symphysis, sacroiliac complex, acetabulum, as well as musculoskeletal and joint degenerative traits of the appendicular and axial skeleton; each with a maximum of three stages) were developed from existing methods, aiming towards a multi‐morphological and most comprehensive assessment (for further details, see the original paper by Navega et al. 2022). One could say that these data capture the full complexity of age‐associated skeletal changes, including their heterogeneity with respect to estimation precision and accuracy (see Fuchs et al. 2026). In contrast, the “Allmales” and Spitalfields datasets are each based on only a single osteological marker: the pubic symphysis (6 stages) and the auricular surface of the ilium (5 traits, 3–5 stages; see above). For both markers, regardless of the bony features assessed (texture, topography, margins), biases are well documented, that is, their use result into a systemic underestimation of older ages (Boldsen et al. 2022; San Millán et al. 2013) or work better *for* older ages (Buckberry and Chamberlain 2002; San Millán et al. 2013). However, we specifically use these different data sets to test our model, as they reflect the “osteological reality” of skeletal age information.

## 5 Results

### 5.1 “Allmales” Dataset

For the different sample sizes of the “Allmales” dataset, the number of saved steps and thinning varied (*n* = 200: 50,000/16,000; *n* = 100: 100,000/3000; *n* = 50: 100,000/2000; *n* = 25: 100,000/500; *n* = 10: 400,000/10,000). While in all cases the ESS value exceeded 10,000, for *n* = 50 and *n* = 10, the maximum PSRF was higher than 1.1 (Tables S1–S7). Both cases concerned the highest trait category, which contained only a few entries.

For the dataset *n* = 200 (Table 1), the estimated mean age was 2.96 years higher than the mean known age‐at‐death (“Bias”). Younger individuals are overestimated and older individuals are underestimated, as indicated by the residual age slope of 0.21 (Figure 3, bottom right), but Pearson's correlation coefficient is 0.75 and thus highly significant. The mean absolute age deviance between known and estimated age‐at‐death is 9.88 years, while the RMSE is 13.38 years, the TMNLP is 4.45 and the CRPS is 7.76. The median range of age estimates of credible intervals at the 95% level is 50.72 years, and this produces a coverage of 90%, which is somewhat lower than the expected 95.0% (Figure 3, top left). The estimated Gompertz parameter is not a very good fit to the empirical age structure of the “Allmales” individuals (Figure 3, top right), as its age mode is much higher than that of the empirical distribution. It has to be remembered, though, that the “Allmales” dataset is not a Gompertzian population.

**TABLE 1:** Goodness‐of‐fit measures for different sample sizes of the “Allmales” dataset.

	*n* = 200	*n* = 100	*n* = 50	*n* = 25	*n* = 10	Lower mortality (Gompertz beta = 0.05)
Bias	2.96	4.62	6.98	8.03	3.59	−0.78
Pearson corr. coeff.	0.75	0.69	0.75	0.82	0.59	0.71
Pearson corr. coeff. *p*	0	0	0	0	0.07	0
Residual age slope	0.21	0.23	0.07	−0.02	0.31	0.19
Inaccuracy	9.88	10.75	11.56	10.83	15.29	10.43
RMSE	13.38	15.32	14.94	14.34	19.15	14.02
TMNLP	4.45	4.40	4.46	4.18	4.62	4.41
CRPS	7.76	8.78	9.42	8.92	9.66	6.97
Median range (at 95%)	50.72	51.42	55.18	53.3	56.0	52.54
Coverage 95%	90	86	90	88	90	93

Figure 3 (bottom left) shows clearly that the thresholds, that is, the transitions from one stage to the next, are not spaced evenly across the age range, but do not follow a strictly logarithmic logic either. Table 2 compares the thresholds between the stages for known age‐at‐death with the estimated values. They diverge by only months or, at most, a few years. For all thresholds, the credible intervals of the 95% level include the computed values for known age‐at‐death.

**TABLE 2:** Known versus estimated thresholds for dataset “Allmales” (*n* = 200, pubic symphysis after Brooks and Suchey 1990).

	Thresholds for known age‐at‐death	Thresholds for estimated age‐at‐death (mode)	HDIlow	HDIhigh
1|2	19.6	20.5	16.3	43.9
2|3	25.6	26.5	22.0	53.8
3|4	29.1	30.5	25.7	58.9
4|5	45.4	47.3	40.7	79.9
5|6	74.7	80.6	62.4	114.9

When the sample size is reduced, the quality measures for goodness‐of‐fit show no marked decline for *n* = 100, *n* = 50 and *n* = 25 compared with *n* = 200 (Table 1). Interestingly, for most measures, the sample of size *n* = 25 even surpasses the sample size of *n* = 50. Only with a size of *n* = 10 do the quality measures deteriorate markedly.

For all datasets, only in a few cases does coverage reach the expected level; mostly it is about 5% below the level it should achieve. However, most of the exact binomial tests for ranges between 10% and 95% are not significant (Tables S8–S13). For comparison, we also run this test for the dataset with low mortality with the original ranges provided by Suchey and Brooks (Brooks and Suchey 1990, 233 table 1) and with those newly published by Godde and Hens (2025, 15 table 6), particularly meant for lookup. Both provide coverages for the 95% confidence level that are decidedly below 90% (Table S14).

**FIGURE 4:**
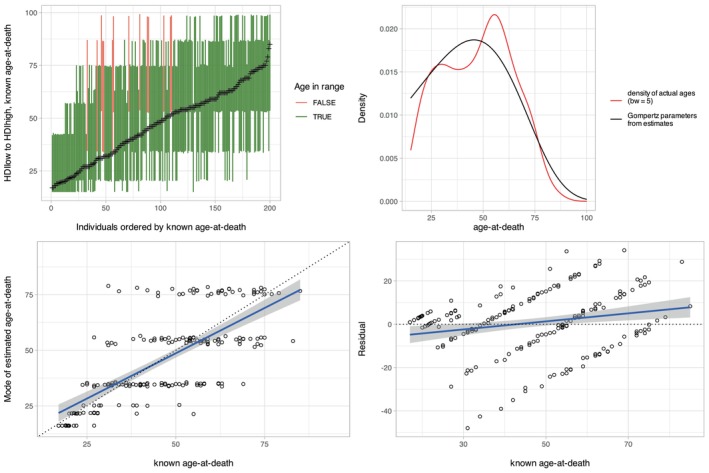
Estimation of age‐at‐death for dataset “Allmales” (*n* = 200, pubic symphysis after Brooks and Suchey 1990) with lower mortality (Gompertz β = 0.05). For explanations, see Figure 3.

In comparison with the original “Allmales” dataset (*n* = 200), the inaccuracy and the coverage of the sampled dataset with lower mortality (Gompertz β = 0.05) are of particular interest (Figure 4; Table 1 last column). Inaccuracy is a little bit higher: 10.43, compared with 9.88 of the original dataset. This obviously resembles the much higher average age, which leaves more room for uncertainty. On the other hand, coverage is very close to the expected range, and this is true for all observed percentage rates (Table 3).

**TABLE 3:** Expected and observed coverage with exact binomial test for “Allmales” dataset with low mortality (Gompertz β = 0.05). None of the *p* values are significant.

Expected coverage	*n* observed	Percentage observed	CI lower (95%)	CI upper (95%)	*p*
0.1	21	0.105	0.066	0.156	0.813
0.2	45	0.225	0.169	0.289	0.377
0.3	65	0.325	0.261	0.395	0.441
0.4	82	0.41	0.341	0.482	0.773
0.5	95	0.475	0.404	0.547	0.525
0.6	120	0.6	0.529	0.668	1
0.7	140	0.7	0.631	0.763	1
0.8	165	0.825	0.765	0.875	0.426
0.9	178	0.89	0.838	0.93	0.637
0.95	186	0.93	0.885	0.961	0.192

Using the mean instead of the mode of the point estimate of age changes some of the quality measures quite strongly (Tables S15 and S16; Figures S1 and S2). Bias is then 8.3 (*n* = 200) and 4.3 years (lower mortality). The residual age slope is also higher, at 0.32 (*n* = 200) and 0.30 (lower mortality), respectively. Inaccuracy and RMSE change inconsistently: Both measures are higher for *n* = 200 (11.70 and 14.72 years) but lower for the dataset with lower mortality (9.87 and 12.87 years). In contrast, TMNLP and CRPS as well as coverage are unaffected, because they are based on the posterior probability distributions of the estimates and not on the point estimates.

### 5.2 CAMSAD

The model for the CAMSAD dataset in most cases met the quality criteria in terms of maximum PSRF and minimum ESS after 20,000 saved steps with a thinning of 5000 (all configurations). With 40 and 64 traits, in each case one of the thresholds does not meet the quality criteria (threshold 1 of trait 30 for 40 traits, threshold 1 of trait 3 for 64 traits), but this seems admissible given the high number of traits (Tables S17–S23).

**FIGURE 5:**
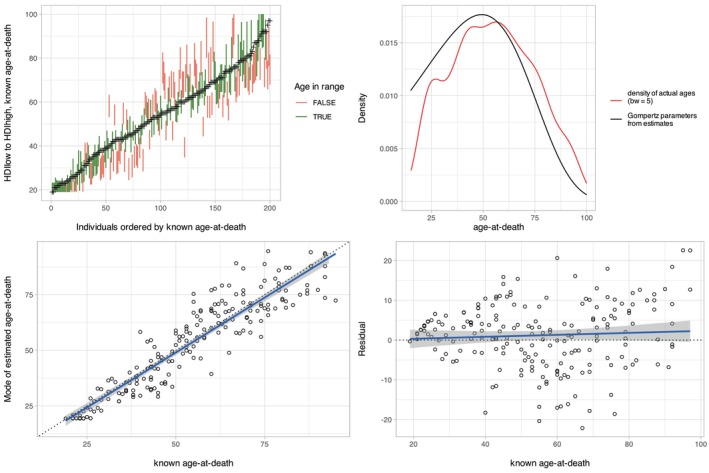
Estimation of age‐at‐death for the reduced dataset CAMSAD (*n* = 200), 64 traits from several anatomical regions (Navega et al. 2022). For explanations, see Figure 3.

With an increasing number of traits, the goodness‐of‐fit measures improve markedly. Even with an increase from 40 to 64 traits, there is still a noticeable improvement. However, overall, the improvements become more marginal after 15 traits. With the full set of 64 traits, the true mortality pattern is obviously accurately estimated, with a bias of −1.21 (Figure 5, top right) and a residual age slope of only 0.03 (Figure 5, bottom right and left), as well as an inaccuracy of only 6.46 years (Table 4, second‐last column).

**TABLE 4:** Goodness‐of‐fit measures for the reduced CAMSAD dataset (*n* = 200) from several anatomical regions (Navega et al. 2022), with an increasing number of traits as well as the entire set of 64 traits with calibration of the uncertainty (sd = 7.5 years).

Number of traits	5	10	15	25	40	64 (uncal)	64 (cal)
Bias	−9.57	−6.25	−3.51	−3.29	−2.13	−1.18	−0.95
Pearson corr. coeff.	0.84	0.89	0.91	0.91	0.92	0.92	0.92
Pearson corr. coeff. *p*	0	0	0	0	0	0	0
Residual age slope	0.27	0.14	0.10	0.08	0.05	0.02	0.04
Inaccuracy	11.37	8.69	7.37	7.2	6.57	6.46	6.42
RMSE	14.48	11.7	9.28	8.92	8.46	8.22	8.25
HDI_within	78	72	73	64.5	62	55	91.5
HDI_Diff_median	31.27	21.37	18.2	14.86	12.47	10.26	30.68
TMNLP	4.44	4.42	4.39	4.42	4.40	4.40	4.39
CRPS	7.64	6.28	5.31	5.25	5.05	5.12	4.41

**FIGURE 6:**
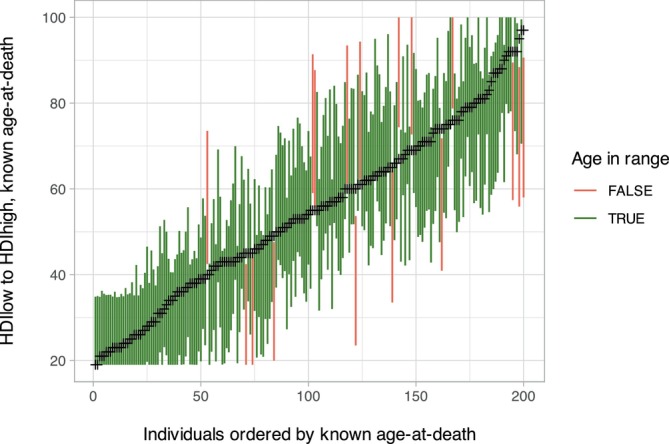
The reduced CAMSAD dataset (*n* = 200) with all 64 traits from several anatomical regions (Navega et al. 2022), with calibration of the uncertainty. Comparison between known age‐at‐death and the estimated 95% HDIs, colored green if the true age lies within the range and red if not.

With more traits, also the 95% HDI range decreases, until it reaches a minimum of 10.31 years with all 64 traits. However, this small range is deceptive, because it contains only 53.5 per‐cent of the true values. The assumption of conditional independence between traits, the problem outlined above, apparently does not hold. Instead, a strong correlation between at least some of the traits needs to be taken into account. With a calibration of the age estimates 1*σ* = 7.5 years, the HDI widens considerably, to 31.2 years. With such a high value, 91.5 per‐cent of the true values are incorporated (Figure 6; Table 4, last column) and exact binomial tests coverage levels from 0.1 to 0.95 are all not significant except for the last (Table S24). It is interesting to note that bias, inaccuracy, TMNLP and CRPS also profit from calibration, while residual age slope and RMSE slightly deteriorate in comparison with those for the uncalibrated values.

### 5.3 Spitalfields

The multivariate normal model for the Spitalfields dataset took 12,000 saved steps with a thinning of 500,000 (so, $6\cdot 10^{9}$ runs altogether). It met the quality criteria in terms of maximum PSRF for most parameters, only threshold 2 of trait MA was higher than 1.1. For the missing data, the “simple” multiple models used 50,000 steps and a thinning of 10,000 each to reach sufficient values in terms of the quality criteria (Tables S25–S30).

**FIGURE 7:**
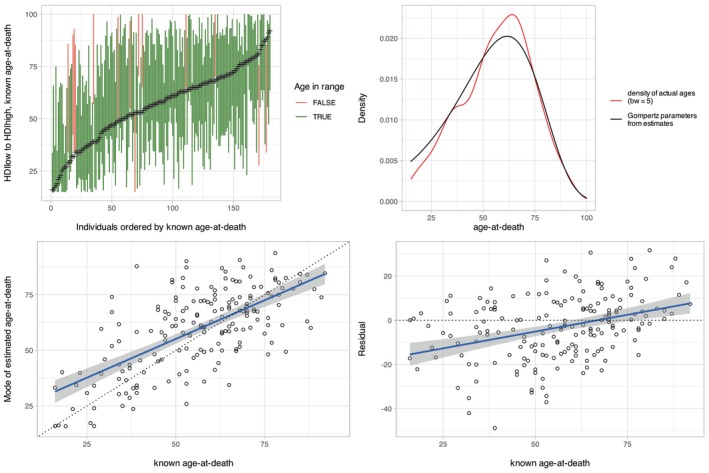
Estimation of age‐at‐death for the Spitalfields dataset with the multivariate normal model (auricular surface of the ilium, Buckberry and Chamberlain 2002). For explanations, see Figure 3.

The mean age of the individuals was overestimated by 3.18 years (Table 5: Bias). Generally, young individuals were overestimated and older ones underestimated, as can be seen in the residual age slope of 0.30 (Figure 7, bottom left and right). The different measures of the level of accuracy were 11.68 (inaccuracy), 14.92 (RMSE), 4.42 (TMNLP) and 7.81 (CRPS).

**TABLE 5:** Spitalfields. Goodness‐of‐fit measures for the entire dataset and the dataset with partly missing data (auricular surface of the ilium, Buckberry and Chamberlain (2002)).

	Full dataset	Missing data			
		20%	30%	40%	50%
Bias	3.18	2.51	−2.71	6.62	−16.16
Pearson corr. coeff.	0.66	0.68	0.59	0.64	0.55
Pearson corr. coeff. *p*	0	0	0	0	0
Residual age slope	0.30	0.28	0.34	0.30	0.46
Inaccuracy	11.68	11.22	13.13	13.58	18.79
RMSE	14.92	14.32	16.79	16.71	22.98
HDI_within	92.22	88.89	88.89	94.44	73.89
HDI_Diff_median	48.42	43.6	48.36	54.74	45.74
TMNLP	4.42	4.32	4.39	4.40	4.54
CRPS	7.81	7.81	8.48	8.31	11.62

Despite slightly overestimating the true age, the parameters for the Gompertz distribution estimate the true mortality very well (Figure 7, top right). The median age range at the credible HDI of 95% is 48.42 years, which captures 92.2% of the true values. This age is only slightly lower than expected, and an exact binomial test, accordingly, is not significant. This is true for most of the ranges (Table 6). Choosing the mean instead of the mode as point estimate, again, only affects bias, residual age slope, inaccuracy and RMSE (Table S31; Figure S3). Bias is only slightly worse (3.53 years), but the residual age slope deteriorates markedly (0.42). In contrast, inaccuracy (10.86 years) and RMSE (13.77 years) profit from using the arithmetic mean.

**TABLE 6:** Spitalfields. Expected and observed coverage with exact binomial test of the multivariate normal model (significant *p* values in bold).

Expected coverage	*n* observed	Percentage observed	CI lower (95%)	CI upper (95%)	*p*
0.1	11	0.061	0.031	0.107	0.083
0.2	27	0.15	0.101	0.211	0.112
0.3	49	0.272	0.209	0.343	0.464
0.4	70	0.389	0.317	0.464	0.82
0.5	88	0.489	0.414	0.564	0.823
0.6	100	0.556	0.48	0.629	0.224
0.7	113	0.628	0.553	0.699	**0.042**
0.8	131	0.728	0.657	0.791	**0.019**
0.9	153	0.85	0.789	0.899	**0.034**
0.95	166	0.922	0.873	0.957	0.088

As is to be expected, if the missing of observations is simulated, the accuracy decreases (Table 5). Still, the responses of the estimates vary considerably: While 20 per‐cent of missing data seems to make little difference—indeed, the accuracy measures are even a little bit better than with the full dataset—the differences are more marked, with 30 per‐cent and 40 per‐cent, though still acceptable. Only with 50 per‐cent of missing data are the estimates more off the mark. For both 30 per‐cent and 50 per‐cent, for one of the traits, the lowest level was knocked out completely (Tables S32–S36). It contained only a few entries, but, obviously, with the lowest level missing, all estimates were made “younger”, which led to a negative bias. Because the Spitalfields population is comparatively old, it contained more entries of the highest level, and therefore in none of the scenarios do we see that all of the highest levels were removed. It is interesting to note, though, that for 40 per‐cent missing, for one of the traits, only one entry remained, which probably led to the higher positive Bias. Most of the exact binomial tests are significant (Table S37–S40), except, interestingly, for the sample with 40% missing data, underlining the fact that the HDI ranges do not encompass enough cases for most levels of credibility.

The age‐at‐transition for the thresholds of the five traits for the full dataset are shown in Table 7. For most of the thresholds, the estimated age‐at‐transition are close to those computed from known age‐at‐death. Quite off is threshold 2|3 of trait MA, because with 50.8 years, its age‐at‐transition it is lower than that of threshold 1|2, with 59.9 years. Threshold 2|3 also yielded an unsatisfactory quality measure (see above), and it is one example where mode (displayed here), mean and median of the probability density of this parameter greatly differ (see Table S25). Furthermore, at first sight, it seems puzzling that the estimates, as well as the known ages, for the highest thresholds are partly far beyond the 100 years mark, thus also far beyond the highest age‐at‐death in the dataset, which is 92. We should remember that the threshold only marks the mode of a probability distribution whose prior is Gaussian. The model as it stands is only able to accommodate the actual data by setting the mode of the last threshold far beyond the data (Kruschke 2015, 696–698 with fig. 23.11).

**TABLE 7:** Spitalfields. Age of transition from known and estimated age‐at‐death for five traits of the auricular surface of the ilium (TO—transverse organization; ST—surface texture; MI—micro‐porosity; MA—macroporosity; AP—apical changes; Buckberry and Chamberlain 2002). MI, MA, and AP only have three levels each.

	Age of transition for known age‐at‐death				Age of transition for estimated age‐at‐death			
	1|2	2|3	3|4	4|5	1|2	2|3	3|4	4|5
TO	10.8	27.4	41.2	114.4	16.6	35.5	48.8	89.2
ST	14.5	26.6	71.2	160.4	16.3	28.3	75.5	134.7
MI	20.4	35			17.6	32.2		
MA	56.1	86.7			59.9	50.8		
AP	24.2	166.6			31.5	113		

## 6 Discussion

Above, we analyzed three datasets that have also been used in the past to develop and demonstrate three different methods of age‐at‐death estimation of adult individuals. Our choice was not intended to make any statement on the appropriateness of the methods employed but was mainly dictated by the availability of data. In the following, we compare and discuss the results of the different datasets in order to facilitate understanding of the possibilities and limitations of our approach.

### 6.1 Accuracy

As stated in the methodology section, accuracy is the systematic deviation of the age estimate from the true age‐at‐death. This deviation is measured by inaccuracy, RMSE, TMNLP and CRPS. Within each dataset, it is obvious that each of accuracy measures generally improves with a larger sample (“Allmales”), with more traits (CAMSAD) or with a lesser number of missing values (Spitalfields dataset). In the best‐case scenarios, inaccuracy is about 10 years for the Suchey–Brooks stages, about 12 years for the Buckberry–Chamberlain stages and as low as about 6 years for the DRNNAGE stages. So, a high number of traits generally increases accuracy, but it does not do so uniformly. Despite the fact that the Buckberry–Chamberlain method incorporates five traits, it is less accurate than the Suchey–Brooks method in this case. Several other studies showed different results for accuracy (compare San Millán et al. 2013, where the former scores better than the latter). Beyond the 15 traits in the CAMSAD dataset, the accuracy only improves slightly, so while there is no doubt that a combination of methods usually leads to more precise age estimates (Buckberry 2015, 327), there is a trade‐off between the improvement in the estimates and the effort involved in recording more traits.

We do not claim to settle the debate about the best method for demonstrating the correlation between estimates and known age‐at‐death. But, for sample‐based estimation, as used in the present study, it seems that CRPS is more suitable than TMNLP to grasp accuracy in a single value. This is visible in all three datasets: the decrease in sample size (“Allmales”), the sequential estimation of more and more traits (CAMSAD dataset); and the increase in missing values (Spitalfields dataset). Despite the obvious expectation that with more traits, the accuracy increases and that with a smaller sample size or with more missing values, it decreases, this was not the case with TMNLP in all cases, but it was with CRPS in most cases. For the best‐case scenario of the Suchey–Brooks stages, the CRPS was 7.8 (high mortality) and 7.0 (low mortality), respectively. Interestingly, for the Buckberry–Chamberlain stages, it was also 7.8, which was already reached with five traits of the CAMSAD dataset. The full set yielded a CRPS value of 5.1.

In relation to both TMNLP and CRPS, it is worth highlighting that they are based on a comparison of the true age‐at‐death with the raw posterior distributions of estimated ages. For the other quality measures, one has to decide if the point estimate is based on the arithmetic mean, the median or the mode. While these will be very similar in many cases, they will differ in some cases. Because of an averaging effect, the mean will lead to estimates that are drawn to the average age of the entire population. This in turn may lead to a better inaccuracy value in comparison with median or mode, but will increase bias and lower correlation between true age‐at‐death and point estimate. In contrast, both TMNLP and CRPS (as well as the HDI) will be unaffected by the choice of the point estimate. When it comes to probability estimates, researchers should be more careful with the point estimate they choose and carefully justify why they have chosen one over the other.

### 6.2 Bias and Residual Age Slope

Above, we defined bias as the mean deviation of estimated from known age‐at‐death. In the best‐case scenarios, bias is less than +/−3 years. For the full CAMSAD dataset and the “Allmales” dataset with lower mortality, it approaches 1 year. However, with a lower number of traits with only a few stages each, there appears to be a risk of underestimation compared with true age (see CAMSAD dataset). In contrast, residual age slope is the systematic deviation of the residuals, the difference between estimated and true age‐at‐death. For the “Allmales” dataset, residual age slope is around 0.2 for the best‐case scenarios, for Spitalfields it is 0.3, but for the CAMSAD data it is only 0.03. While it seems obvious that the many traits of the CAMSAD dataset pay off in terms of a low residual age slope, the fact that the Buckberry–Chamberlain data show a higher residual age slope than the Suchey–Brooks data is surprising. Again, seemingly, a higher number of traits does not guarantee low residual age slope. So, our approach does not by itself circumvent the problem seen with regular regression analysis that young individuals are overestimated and older ones underestimated (Aykroyd et al. 1997). Obviously, the higher number of stages of the Suchey–Brooks method “anchor” the estimates more firmly at the lower and upper ends. Generally, it has been claimed that more traits with fewer stages minimize the problem of misclassification (Shirley and Ramirez Montes 2015). However, Rizos et al. (2024) show for the DRNNAGE system that this is not necessarily the case.

Konigsberg (2015), 372–373 with (fig. 6) has called into question the statement of Milner and Boldsen (2012, 100) that “point estimates of age‐at‐death are not affected by the lack of conditional independence” and assumes that this is only true when many age indicators are used. The reduction in bias observed with the CAMSAD dataset when more and more traits are incorporated can be regarded as a point in Konigsberg's favor.

### 6.3 Age Ranges, Precision and Efficiency

Ideally, age ranges should be both precise and efficient: they should be as narrow as possible (= efficient) while still encompassing the true age‐at‐death (= precise). Bearing in mind the high variation in skeletal age markers, it is clear that we are dealing here with a trade‐off. If the estimated age ranges are to include all cases correctly, they would have to be so wide that they would be of little practical use. Therefore, confidence (or, in the case of Bayesian applications, credibility) intervals are given that ideally bracket the correct number of cases at the given confidence or credibility level (coverage).

For the “Allmales” dataset, the Suchey–Brooks stages result in age ranges of slightly over 50 years. The fact that this dataset is a composite dataset that does not resemble a natural “Gompertz population” explains why, for example, at 95% coverage is only 90% and thus below expectation. For the sampled “Allmales” dataset with lower mortality following a Gompertz distribution, the reported coverage is met. For the Buckberry–Chamberlain dataset, the multivariate model arrives at age ranges slightly below 50 years and mostly meets the expected coverage. In contrast, the CAMSAD dataset demonstrates a case in which the age range is estimated far too optimistically, which can only be remedied by calibration. Then, an age range of slightly more than 30 years seems realistic. Calibrating the ages does not change the other accuracy measures much (and when it does, it does so mostly for the better).

### 6.4 Comparison With Other Studies

In the following comparison, we concentrate on studies which tested the same age markers we used in our test cases (Suchey‐Brooks‐phases for pubis symphysis, Buckberry‐Chamberlain for auricular surface, DRNNAGE for multiple indicators).

It is well known that the Suchey‐Brooks‐method, such as others, tends to underestimate older individuals. In a Spanish study, inaccuracy was 14.38 years and bias −12.74 years (San Millán et al. 2013, 1746 table 2), while coverage was 85.8% (ibid. 1745 table 1). In a study of a modern white South‐African population, bias was −16.2 years for male and −11.6 years for female individuals (Joubert et al. 2019), compared to, for example, 2.96 years for the original Allmales data set (*n* = 200) or −0.78 years for the sample with lower mortality. The inaccuracy of 9.56 years for the original data set is lower than reported for a Modern Greek sample, in which the value ranged between 10.41 and 12.42, depending on sex and side (Xanthopoulou et al. 2018, p. table 4). It must be acknowledged that this may be due to the specific structure of the Allmales data set, which includes many very young individuals for whom the estimated age ranges are smaller than for the older age groups. With a higher modal age, the inaccuracy is indeed higher and age ranges are at about 10.4 years. This is still at the lower end of the Greek study. For several Chinese samples, the inaccuracy spans from 8.83 to 13.7 years (Xiong et al. 2023). In a comparison study, inaccuracy was reported as 12.97 years, with a coverage of 84% (Bailey and Vidoli 2023, 185 table 5).

Some of the original age ranges for the Suchey‐Brooks‐method are also larger than 50 years, but most are narrower (Brooks and Suchey 1990, 233 table 1). Combining the original data set with Bayesian methodology, new age ranges were recently provided (Godde and Hens 2025). The latter are slightly narrower, but more or less agree with the original ones, which is not surprising since they are based on the same data. However, in a test neither of them provided the coverage promised (95%) (Godde and Hens 2025, 16 table 7).

Some of the age ranges Buckberry and Chamberlain specify for their stages are quite narrow, but most cover a span of about 40–50 years (Buckberry and Chamberlain 2002, 237 table 12). For the original analysis, an *r*‐value of 0.63 is reported (Buckberry and Chamberlain 2002, 235). With the present approach, it is even slightly higher (0.66). Quality measures of testing the Buckberry/Chamberlain method are in the same range or worse than our results. For example, Mulhern and Jones (2005, 63 table 2) calculated an inaccuracy of 13.03 years and a bias of −0.04 years for a sample from the Terry and Huntington Collections. Falys et al. (2006, 510) report an overall inaccuracy of 9.8 years for individuals from St Bride's crypt, and separate *r*‐values for females and males of 0.554 and 0.629, respectively. Hens and Belcastro (2012, 3 table 4) found an inaccuracy of 11.8 and 13.9 years for males and females of a Sardinian sample, respectively. Bias was 4.0 and 2.6 years. San Millán et al. (2013, 1746 table 2) report an inaccuracy of 11.24 years and a bias of 0.41 years, the coverage is 93.7% years (ibid. 1745 table 1). In the case of Maaranen and Buckberry (2018, 26 table 2), coverage (percentage of individuals within specified range) was as low as 73.5 percent, inaccuracy was 15.7 years and bias 13.7 years. Bailey and Vidoli (2023, 185 table 5) report that 88% of their individuals were within the specified range, inaccuracy was 12.77 years, and bias was 6.44 years.

For the newly established DRNNAGE, the authors report an age range of 30.1 years for all 64 traits (Navega et al. 2022). However, Rizos et al. (2024, 922 table 4) stated an overall inaccuracy of 14.5 years instead of the around 6 years reported in the original study. Despite the claims of the original study, Rizos et al. found it very difficult to consistently score the traits. For the complete data set of 500 individuals, Navega et al. (2022, 17 table 4) report an MAE of 5.899 and a bias of 0.118. Interestingly, while the MAE of our model is 6.45 and thus slightly higher by 9%, its residual age slope is only 0.02 which is better by the factor 5. Especially in the older age categories, the DRNNAGE model performs more poorly than our model. However, this might also be due to the fact that by simulating a Gompertz population, we necessarily excluded some of the older individuals which might be particularly problematic in terms of their traits' expression.

Overall, the estimates of our model seem to be at least as reliable as those from the other approaches, but more tests are necessary to verify this.

### 6.5 Advantages of Our Approach

As the results from the “Allmales” dataset demonstrated, sample size does matter, but obviously only to a certain extent. The statement by Boldsen et al. (2002, 77) that “the Rostock protocol almost certainly requires samples larger than those found in most forensic or archaeological situations” is unwarranted, as useful results can be obtained with as little as 25 individuals.

Another important advantage of the approach presented here is the fact that there is no need for the so‐called “invariance hypothesis”, which “assumes that the conditional distribution of stage indicators at a given age is constant over time” (Séguy et al. 2013, 171). For our method, the only requirement is that the trait in question change monotonously—but not necessarily continuously—with biological age. Our model demands consistency within a population, but not necessarily between populations. That means that inter‐observer error is far less of a problem. Until now, priors for age‐at‐transition have been taken from reference populations. The main problem is that age of transition can be population specific (see Godde and Hens 2012; Konigsberg et al. 2016, 573, for concrete examples). As a consequence, age‐at‐transition established for one population might not be appropriate for another population. Within our approach, these age‐at‐transition estimates are estimated on the fly and are allowed to differ between populations.

Several authors have highlighted the need for appropriate population priors (e.g., Godde and Hens 2012; Kim and Algee‐Hewitt 2021; Nikita et al. 2018). Navitainuck et al. (2022) have shown that differences in estimated population means are directly related to the mean age of the reference population of the method in question, a phenomenon known as age mimicry (ibid.). In our approach, the population prior is also computed on the fly. Therefore, it does not rely on priors from reference populations and age mimicry is avoided, even on the population level.

As a corollary of our method, the Gompertz hazard parameters of the populations under study are estimated. As the comparison between the resulting function curves and the density distribution shows, these describe the populations reasonably well.

Our model could also easily be extended towards the inclusion of continuous variables; however, currently only very few age traits of continuous morphological change for adults exist (e.g., bone mineral density: Navega et al. 2018, and, with somewhat less optimistic results, Bethard et al. 2019).

### 6.6 Shortcomings

One significant limitation of the model concerns its applicability in terms of the possible age range of the human life span. The application of the Gompertz distribution implies an earliest starting point for the estimate at the age of 15 (cf. Wood et al. 2002, 137). Making it usable for the entire possible age range would require a different probability distribution. This could be the so‐called Siler distribution (Wood et al. 2002, 147–150), but more work is required to establish its fit to the subadult age range.

If population mortalities do not follow a Gompertz distribution, for example, in the case of cemeteries holding the dead from catastrophic events or in uniform samples, such as the full CAMSAD dataset, it is to be expected that the age estimates and their ranges will be compromised. Apart from having a dataset that conforms to the basic assumption that the included traits are monotonically associated with age, it is important that it also has instances of all scores of a trait be represented, which is a corollary of the requirement that the adult mortality conforms to a Gompertz distribution. This puts a constraint on the lower limit of the size of the population analyzed, which must be large enough that it comprises all stages. As shown above, depending on the trait(s) and their distribution, a size of 25+ individuals might still be sufficient.

In multivariate ordinal regression (but also when using other algorithms; see Jeliazkov et al. 2008, 131), missing values are not allowed (Plummer 2017, 15). Given the often‐incomplete nature of osteological material, this is a major drawback of the method. In such cases, one can resort to “simple” multiple ordinal regression, as in such regressions missing values of the variable(s) to be predicted are no complication, as missing values are treated as parameters to be estimated and thus are sampled from the prior distribution. For the CAMSAD dataset, the calibration value was taken from the literature, which worked quite well. If there are sufficient entries without missing values, the multivariate model could still be useful to find expected HDI ranges, which could then be used to calibrate the uncertainties of the simple model. In this setting, the dataset for the multivariate model would be limited to the complete cases and used to come up with sensible ranges for the HDIs. The goal would then be to arrive at similar ranges by setting the uncertainty of the multiple ordinal regression to an appropriate value.

The LKJ prior might not be the best choice if the variables are strongly correlated. This would, for example, be the case for the cranial sutures (Konigsberg 2015, 372 table 4). The approach will also likely produce mixed results if the traits used are only poorly correlated with age. The poor performance is related to the fact that then there will be no clear value peak in the ordinal data. This, again, relates specifically to the cranial sutures, which have been known for a long time to correlate only weakly with age (ibid. 371 table 2).

Finally, the approach outlined above will, admittedly, be of limited use to osteologists when dealing with a single individual, as is typical in forensics but also occurs in archaeology. Instead, it is designed for studies that analyze the age‐at‐death and mortality profiles for a skeletal assemblage. In single‐case studies, if the osteologists have appropriate population data at hand, there is the possibility to add the scores of the single individual in order to model them together. However, a very large population could also pose a problem due to computational limits. For instance, the dataset “Allmales”, used in our study, which comprises more than 1700 individuals, had to be reduced to 200 individuals to be calculated within a manageable time span. Large sample sizes will test the researcher's patience due to the time‐consuming calculations, but split‐ups could be a solution.

## 7 Conclusion

We hope we have shown that a Bayesian version of transition analysis faithful to the Rostock Manifesto is possible. While we do not claim that our approach solves all problems of adult age‐at‐death estimation, we do claim that it may help to alleviate at least some of them. Instead of employing one step for defining a reference population and another one for defining the transition parameters, we combined these two steps into a single model, meaning that reference populations for the age‐at‐transition, as well as the priors, become unnecessary. This may be especially of relevance when there is no adequate reference population available, that is, for archaeological skeletal assemblages from older periods.

Our approach can be applied to any kind of skeletal characteristic that changes with advancing age. Already 10 years ago, Buckberry (2015, 330) stated that future “[computer interfaces] must include probability densities for popular and traditional methods, to facilitate re‐analysis of data collected over the last 20–30 years”. We claim that our approach provides such an interface. If the results of the age estimation process had always been reported in full detail (scores per method, individual, and trait), it would actually be possible to apply our approach on a large scale to data gathered in past research.

There is still considerable room for improvement, especially in the handling of missing age traits in relation to group‐wise weighing of correlated traits or to calibration varying with age. Still, our approach offers a true alternative to current approaches. It seems to be especially fruitful when applied to material with very different preservation stages and with as many age traits as possible. This should also be useful when analyzing commingled complexes with only partially articulated skeletons. Undoubtedly, smaller sample sizes increase the danger of spurious elements exerting more influence on the results. A healthy skepticism about one's own data is always helpful, but we hope that we have shown that even smaller samples might yield useful results.

Our method code in JAGS, NIMBLE and R is freely available, and a user‐friendly package named “baytaAAR” has been submitted to CRAN. Its vignettes contain typical workflows, and we explicitly encourage the community to further develop the method by testing it with additional and diverse known‐age‐at‐death samples.

## Author Contributions

**Katharina Fuchs:** writing – original draft, writing – review and editing, methodology. **Christoph Rinne:** writing – original draft, writing – review and editing, visualization, software, data curation, validation. **Nils Müller‐Scheeßel:** conceptualization, funding acquisition, writing – original draft, methodology, visualization, writing – review and editing, data curation, supervision, software.

## Funding

This work was funded by the Deutsche Forschungsgemeinschaft (DFG, German Research Foundation)–Projektnummer 560057566.

## Ethics Statement

This work only uses published data.

## Conflicts of Interest

The authors declare no conflicts of interest.

## Supporting information


**Table S1:** Allmales dataset samples summarized diagnostics.
**Table S2:** MCMC basic diagnostics Allmales sample size *n* = 200.
**Table S3:** MCMC basic diagnostics Allmales sample size *n* = 100.
**Table S4:** MCMC basic diagnostics Allmales sample size *n* = 50.
**Table S5:** MCMC basic diagnostics Allmales sample size *n* = 25.
**Table S6:** MCMC basic diagnostics Allmales sample size *n* = 10.
**Table S7:** MCMC basic diagnostics Allmales lower mortality, sample size *n* = 200.
**Table S8:** Exact binomial test for Allmales (*n* = 200).
**Table S9:** Exact binomial test for Allmales (*n* = 100).
**Table S10:** Exact binomial test for Allmales (*n* = 50).
**Table S11:** Exact binomial test for Allmales (*n* = 25).
**Table S12:** Exact binomial test for Allmales (*n* = 10).
**Table S13:** Exact binomial test for Allmales with lower mortality (*n* = 200).
**Table S14:** Exact binomial test for two Allmales data‐sets with the Suchey‐Brooks method and with original and Bayesian age ranges.
**Table S15:** Goodness‐of‐fit measures for the Allmale data‐set (*n* = 200), with the arithmetic mean as point estimate.
**Table S16:** Goodness‐of‐fit measures for the Allmale data‐set with lower mortality (*n* = 200), with the arithmetic mean as point estimate.
**Table S17:** CAMSAD dataset, diagnostic data.
**Table S18:** MCMC diagnostic data for CAMSAD dataset (*n* traits = 5).
**Table S19:** MCMC diagnostic data for CAMSAD dataset (*n* traits = 10).
**Table S20:** MCMC diagnostic data for CAMSAD dataset (*n* traits = 15).
**Table S21:** MCMC diagnostic data for CAMSAD dataset (*n* traits = 25).
**Table S22:** MCMC diagnostic data for CAMSAD dataset (*n* traits = 40).
**Table S23:** MCMC diagnostic data for CAMSAD dataset (*n* traits = 64).
**Table S24:** Exact binomial test for CAMSAD dataset (*n* traits = 64) with calibrated age‐at‐death estimate.
**Table S25:** Spitalfields dataset summarized diagnostics.
**Table S26:** MCMC diagnostic data Spitalfields dataset.
**Table S27:** MCMC diagnostic data Spitalfields dataset (20% missing).
**Table S28:** MCMC diagnostic data Spitalfields dataset (30% missing).
**Table S29:** MCMC diagnostic data Spitalfields dataset (40% missing).
**Table S30:** MCMC diagnostic data Spitalfields dataset (50% missing).
**Table S31:** Spitalfields. Goodness‐of‐fit measures for the whole data‐set, mit arithmetic mean as point estimate.
**Table S32:** Spitalfields. Count per trait and level for complete data‐set.
**Table 33:** Spitalfields. Count per trait and level for 20% missing data.
**Table 34:** Spitalfields. Count per trait and level for 30% missing data.
**Table 35:** Spitalfields. Count per trait and level for 40% missing data.
**Table 36:** Spitalfields. Count per trait and level for 50% missing data.
**Table 37:** Exact binomial test for Spitalfields (20% missing).
**Table 38:** Exact binomial test for Spitalfields (30% missing).
**Table 39:** Exact binomial test for Spitalfields (40% missing).
**Table 40:** Exact binomial test for Spitalfields (50% missing).
**Figure S1:** Estimation of age‐at‐death for data‐set “allmales” (*n* = 200). Left top—comparison between known age‐at‐death and the estimated 95%‐HDIs, colored green if the true age lies within the range and red if not; top right—density of known age‐at‐death and Gompertz function with estimated parameters; bottom left—known age‐at‐death versus arithmetic mean of estimated age‐at‐death, the dotted line marks complete equivalence, regression line in blue; bottom right—known age‐at‐death versus residuals (known age‐at‐death minus arithmetic mean of estimated age‐at‐death), the dotted line marks complete equivalence, regression line in blue.
**Figure S2:** Estimation of age‐at‐death for data‐set “allmales” (*n* = 200). Left top—comparison between known age‐at‐death and the estimated 95%‐HDIs, colored green if the true age lies within the range and red if not; top right—density of known age‐at‐death and Gompertz function with estimated parameters; bottom left—known age‐at‐death versus arithmetic mean of estimated age‐at‐death, the dotted line marks complete equivalence, regression line in blue; bottom right—known age‐at‐death versus residuals (known age‐at‐death minus arithmetic mean of estimated age‐at‐death), the dotted line marks complete equivalence, regression line in blue.
**Figure S3:** Estimation of age‐at‐death for data‐set Spitalfields with the multivariate normal model. Left top—comparison between known age‐at‐death and the estimated 95%‐HDIs, colored green if the true age lies within the range and red if not; top right—density of known age‐at‐death and Gompertz function with estimated parameters; bottom left—known age‐at‐death versus arithmetic mean of estimated age‐at‐death, the dotted line marks complete equivalence, regression line in blue; bottom right—known age‐at‐death versus residuals (known age‐at‐death minus arithmetic mean of estimated age‐at‐death), the dotted line marks complete equivalence, regression line in blue.

## Data Availability

All data and code is available online and at Github (https://github.com/ISAAKiel/MCMC_transition_analysis).
