# Supplementary material for: A Fully Bayesian Approach to Adult Skeletal Age Estimation: Multivariate Latent Trait Modeling With Markov Chain Monte Carlo Sampling
Source: Am J Biol Anthropol. 2026 Jun 7;190(2):e70289. doi: 10.1002/ajpa.70289 (PMC13243134; doi:10.1002/ajpa.70289)
Supplement: Supplementary file 1 — Table S1: Allmales dataset samples summarized diagnostics. Table S2: MCMC basic diagnostics Allmales sample size n = 200. Table S3: MCMC basic diagnostics Allmales sample size n = 100. Table S4: MCMC basic diagnostics Allmales sample size n = 50. Table S5: MCMC basic diagnostics Allmales sample size n = 25. Table S6: MCMC basic diagnostics Allmales sample size n = 10. Table S7: MCMC basic diagnostics Allmales lower mortality, sample size n = 200. Table S8: Exact binomial test for Allmales (n = 200). Table S9: Exact binomial test for Allmales (n = 100). Table S10: Exact binomial test for Allmales (n = 50). Table S11: Exact binomial test for Allmales (n = 25). Table S12: Exact binomial test for Allmales (n = 10). Table S13: Exact binomial test for Allmales with lower mortality (n = 200). Table S14: Exact binomial test for two Allmales data‐sets with the Suchey‐Brooks method and with original and Bayesian age ranges. Table S15: Goodness‐of‐fit measures for the Allmale data‐set (n = 200), with the arithmetic mean as point estimate. Table S16: Goodness‐of‐fit measures for the Allmale data‐set with lower mortality (n = 200), with the arithmetic mean as point estimate. Table S17: CAMSAD dataset, diagnostic data. Table S18: MCMC diagnostic data for CAMSAD dataset (n traits = 5). Table S19: MCMC diagnostic data for CAMSAD dataset (n traits = 10). Table S20: MCMC diagnostic data for CAMSAD dataset (n traits = 15). Table S21: MCMC diagnostic data for CAMSAD dataset (n traits = 25). Table S22: MCMC diagnostic data for CAMSAD dataset (n traits = 40). Table S23: MCMC diagnostic data for CAMSAD dataset (n traits = 64). Table S24: Exact binomial test for CAMSAD dataset (n traits = 64) with calibrated age‐at‐death estimate. Table S25: Spitalfields dataset summarized diagnostics. Table S26: MCMC diagnostic data Spitalfields dataset. Table S27: MCMC diagnostic data Spitalfields dataset (20% missing). Table S28: MCMC diagnostic data Spitalfields dataset (30% missing). Table S29: MCMC d [file AJPA-190-e70289-s001.pdf]

A Fully Bayesian Approach to Adult Skeletal Age Estimation: Multivariate Latent Trait Modeling with Markov Chain Monte Carlo sampling

Supplement

Nils Müller-Scheeßel*   Katharina Fuchs†   Christoph Rinne‡

13. March 2026

Contents

Modelling background **3**

Modelling prerequisites **4**

1 Allmales **5**
- 1.1 Diagnostic data . . . 5
  - 1.1.1 Summarized diagnostic data . . . 5
  - 1.1.2 Allmales n = 200 . . . 5
  - 1.1.3 Allmales n = 100 . . . 12
  - 1.1.4 Allmales n = 50 . . . 17
  - 1.1.5 Allmales n = 25 . . . 20
  - 1.1.6 Allmales n = 10 . . . 23
  - 1.1.7 Allmales n = 200 with lower mortality (Gompertz $\beta = 0.05$) . . . 25
- 1.2 Exact Binomial Tests . . . 32
  - 1.2.1 Allmales n = 200 . . . 32
  - 1.2.2 Allmales n = 100 . . . 32
  - 1.2.3 Allmales n = 50 . . . 32
  - 1.2.4 Allmales n = 25 . . . 33
  - 1.2.5 Allmales n = 10 . . . 33
  - 1.2.6 Allmales n = 200 with lower mortality (Gompertz $\beta = 0.05$) . . . 33
  - 1.2.7 Suchey-Brooks method . . . 33
- 1.3 Arithmetic mean as point estimate of age-at-death . . . 34
  - 1.3.1 Allmales n = 200 . . . 34
  - 1.3.2 Allmales n = 200 with lower mortality (Gompertz $\beta = 0.05$) . . . 35

2 CAMSAD **37**
- 2.1 Diagnostic data . . . 37
  - 2.1.1 Summarized diagnostic data . . . 37
  - 2.1.2 5 traits . . . 37
  - 2.1.3 10 traits . . . 44
  - 2.1.4 15 traits . . . 52
  - 2.1.5 25 traits . . . 61
  - 2.1.6 40 traits . . . 71
  - 2.1.7 64 traits . . . 78
- 2.2 Exact Binomial Test . . . 98

*Institute for Prehistoric and Protohistoric Archaeology - Kiel University nils.mueller-scheessel@ufg.uni-kiel.de
†Institute for Prehistoric and Protohistoric Archaeology - Kiel University, k.fuchs@ufg.uni-kiel.de
‡Institute for Prehistoric and Protohistoric Archaeology - Kiel University, crinne@ufg.uni-kiel.de

3 Spitalfields **99**
3.1 Diagnostic data 99
3.1.1 Summarized diagnostic data 99
3.1.2 Full set 99
3.1.3 20% missing 107
3.1.4 30% missing 114
3.1.5 40% missing 121
3.1.6 50% missing 128
3.2 Arithmetic mean as point estimate of age-at-death 135
3.3 Counts per trait and level with missing data 135
3.4 Exact Binomial Tests 137

References **139**

Modelling background

Despite the fact that Markov Chain Monte Carlo (MCMC) sampling was already proposed as a possible avenue in the Rostock volume (Konigsberg & Herrmann, 2002) and OpenBUGS code was also presented by L. Konigsberg on his website, most applications work with Maximum Likelihood Estimation (MLE). While this can be substantially faster than MCMC sampling, it is less flexible and might not be able to come to a solution if the model is too complex.

There are several R-packages specifically for latent variable analysis (notably blavaan: Merkle & Rosseel, 2018). However, they cannot be used here because the model we need is more complicated because the covariate age at death is itself to be modeled and is not observed as usually expected by these packages. Therefore, we decided for JAGS/NIMBLE with its greater flexibility. The implementation of JAGS in R is more user-friendly than NIMBLE, especially when it comes to parallel processing, but only NIMBLE currently allows the implementation of the multivariate model. A very popular choice in relation to MCMC sampling is also *Stan* but *Stan* currently lacks an equivalent of the `dinterval` distribution which is central in our approach.

To ensure identifiability of the multivariate model, there are two basic strategies: 1) use a correlation matrix instead of a covariance matrix, which effectively limits the covariance at unity, and fix one of the cutpoints; 2) use a covariance matrix, but fix two of the cutpoints (Jeliazkov, Graves, & Kutzbach, 2008, p. 130). The latter case has the difficulty that it will not work with binary variables with only two levels because this implies the existence of only one cutpoint (which would preclude a lot of the traits of the CAMSAD data-set, for example). But even with more than two levels, we had difficulties to devise a model with satisfactory results. The main challenge is to define the covariance matrix of the multivariate normal (or, more precisely, the precision matrix as the normal distribution in JAGS is defined by the precision τ $(=\frac{1}{\sigma^2})$, not the standard deviation σ). In the literature, there are some suggestions for this problem, which have been nicely summarised by Th. Riecke on the Google discussion group "Hierarchical Modeling in Ecology". The most obvious solutions are the `Inverse Wishart` and the `scaled Inverse Wishart` distributions because they are conjugate priors for the precision matrix of the multivariate normal. However, they are looked at critically in the literature (Lemoine, 2019, pp. 923–925; Riecke, Sedinger, Williams, Leach, & Sedinger, 2019; Schuurman, Grasman, & Hamaker, 2016), and we also found that it is very difficult to adjust the parameters meaningfully. Therefore, we opted for the first scenario with the correlation matrix and decided for the so-called Lewandowski-Kurowicka-Joe (LKJ) distribution (Lewandowski, Kurowicka, & Joe, 2009) with Cholesky decomposition as reasonable prior. This makes it possible to define a more or less arbitrary correlation matrix which is then used as prior for the multivariate normal distribution.

There are at least five ways how to implement an ordered probit regression model in the BUGS/JAGS script language. The first is the code as published in the classic BUGS examples (e. g. example inhaler, with `logit` instead of `phi`). It builds on a combination of the categorical probability distribution `dcat` and `phi`, the cumulative standard normal probability, so the standard deviation of the latent variable is automatically fixed at unity (= 1). The second as implemented in Kruschke (2015, p. 676) resembles the first. However, it uses the cumulative normal probability distribution which allows for varying standard deviations. Kruschke fixes two thresholds to secure identifiability. The third seems to be an implementation of `Stata` code and uses a very peculiar combination of the `dcat`-distribution and the `step`-function of JAGS. The fourth is the implementation as used here (code adapted from Bayesian methods by O. Kirchkamp, p. 89). On first glance, it seems that it is not probit in the proper meaning of the word as it does not use the cumulative normal distribution explicitly. However, it achieves the same outcome via the `dinterval` function in combination with the normal (or multivariate normal) distribution. Furthermore, it is the only solution which easily allows for a multivariate normal distribution as latent variables. Fifth, from JAGS 3.4 onwards there is a dedicated probability distribution for the ordered probit model, termed `dordered.probit` (JAGS manual, p. 60). It is a little bit slower than the interval-solution used here, but shows better convergence. So, overall there would be a gain in time when using `dordered.probit`. Unfortunately, the algorithm seems to run into numerical problems with our model and crashes ungracefully. Furthermore, the `dordered.probit` model would also not generalise, as does solution four, to the multivariate normal model.

Modelling prerequisites

The calculations were made in R using RStudio. The structure of the code is essentially based on the structure of the text. The backbone of the raw code is the file '1_start.R', it uses the function `source()` to reference specific parts of the calculation.

The PDF is generated from the Markdown document '1_start.Rmd', which is essentially derived from '1_start.R'. It is used to facilitate reproducibility and to provide additional information that is not printed in the text, e.g. tables (supplementary data).

- Depending on the hardware, the subsequent code can run for several hours or even a few days.
- The variable `runNewMCMC` can be set TRUE or FALSE to avoid this (s.b.).
- Install "Just Another Gibbs Sampler" (JAGS: Plummer, 2003) if you want to run the Bayesian analyses anew.
- Version 4.3 - as used here - can be downloaded in pre-compiled form for a number of OS here: https://sourceforge.net/projects/mcmc-jags/ .
- The manual can be found here: https://people.stat.sc.edu/hansont/stat740/jags_user_manual.pdf

Install required packages and set some options.

```
require(pacman) || install.packages("pacman")
```

```
## Loading required package: pacman
## [1] TRUE
```

```
pacman::p_load(coda, dplyr, flexsurv, ggplot2, ggpubr, kableExtra, MASS, Metrics,
               nimble, parallel, rjags, runjags, scoringRules, tidyr)
```

```
options(dplyr.summarise.inform = FALSE)
source("./functions/bayes_helper.R")
source("./2_process_data.R")
source("./functions/bay_ta_jags.R")
source("./functions/bay_ta_nimble_norm.R")
source("./functions/bay_ta_nimble_mnorm.R")
source("./functions/bay_ta_nimble.R")
source("./functions/gompertz_nimble.R")
```

Important note!

Set `runNewMCMC=TRUE` to run new MCMC analyses. The preset `runNewMCMC=!TRUE` (NOT TRUE) uses the MCMC chains from the last calculation. Because all MCMC processes are seeded, they should give the same results every time they are run. By using previously saved chains, all processes (i.e., knit) are much faster. As already mentioned, **re-execution of the MCMC code can take several hours or even days**.

```
runNewMCMC = !TRUE
```

In addition, we save the preprocessed files in a specific folder:

- ./preprocessed_files/
- ./images/

1 Allmales

Supplementary information for the Allmales dataset (Konigsberg, Herrmann, Wescott, & Kimmerle, 2008).

```
source("./3a_allmales_200.R") # can take 1 - 5 minute(s)
source("./3b_allmales_100.R") # can take 1 - 3 minute(s)
source("./3c_allmales_50.R") # can take a minute
source("./3d_allmales_25.R") # can take a minute
source("./3e_allmales_10.R") # can take a minute
source("./3f_allmales_200_old_age.R") # can 1 - 5 a minute(s)
source("./3g_allmales_SB_binomial.R")
```

We calculated the MCMC statistics for different sample sizes (s. main text Table 1). The subsequent tables provide additional diagnostic data of the calculation.

1.1 Diagnostic data

1.1.1 Summarized diagnostic data

Summarized diagnostic data (e.g. PSRF) for the Allmales dataset samples.

Table 1: Allmales dataset samples summarized diagnostics.

	PSRF_max	PSRF_upper_max	ESS_min
sample 200	1.009	1.009	12346.8
sample 100	1.008	1.008	11051.7
sample 50	1.250	1.251	22159.6
sample 25	1.099	1.099	12264.8
sample 10	1.250	1.250	400002.0
lower mortality	1.005	1.006	11141.3

1.1.2 Allmales n = 200

Table 2: MCMC basic diagnostics Allmales sample size n = 200.

	PSRF Point est.	PSRF Upper C.I.	Mean	Median	Mode	ESS	MCSE	HDImass	HDIlow	HDIhigh
b	1.0001512	1.0003953	0.0342988	0.0290458	0.0221897	25989.0	0.0000985	0.95	0.0200002	0.0725883
a	1.0000205	1.0001838	0.0135790	0.0140617	0.0167294	34013.6	0.0000398	0.95	0.0001233	0.0245143
M	1.0000168	1.0001794	40.4119574	39.9751000	47.0191537	34005.7	0.1160408	0.95	6.8848000	81.9867000
beta0	1.0004025	1.0013925	-18.8504438	-18.8069000	-18.8392804	13813.7	0.0602492	0.95	-33.2224000	-5.0554100
beta	1.0004159	1.0014388	6.1187967	6.0971600	6.0082547	12635.8	0.0200392	0.95	1.6121800	10.5389000
thresh[1,1]	NaN	NaN	0.5000000	0.5000000	0.4996965	0.0	NaN	0.95	0.5000000	0.5000000
thresh[1,2]	1.0002076	1.0005186	2.2101620	2.1486800	2.0694783	14637.7	0.0057976	0.95	0.9412040	3.5214500
thresh[1,3]	1.0003675	1.0009915	3.0706242	3.0072800	2.9144268	12971.9	0.0088524	0.95	1.1849900	4.8844800
thresh[1,4]	1.0003188	1.0011167	5.5116888	5.4321800	5.3295891	12346.8	0.0169498	0.95	1.9500800	8.7906600
thresh[1,5]	1.0003710	1.0013488	7.8615651	7.7380400	7.5686049	12422.8	0.0238731	0.95	2.9148800	12.5210000
thresh_age_log[1,1]	1.0002574	1.0007773	3.1632306	3.1012100	3.0248527	44939.2	0.0012290	0.95	2.8509600	3.8174900
thresh_age_log[1,2]	1.0000906	1.0004820	3.4506894	3.3944100	3.2814411	41399.0	0.0011756	0.95	3.1101900	3.9953000
thresh_age_log[1,3]	1.0000468	1.0002865	3.5944070	3.5453100	3.4252936	39563.1	0.0011242	0.95	3.2668500	4.0930300
thresh_age_log[1,4]	1.0001844	1.0005004	4.0054538	3.9707300	3.8731930	27590.4	0.0011364	0.95	3.7249300	4.3964000
thresh_age_log[1,5]	1.0016561	1.0022815	4.4137176	4.3658300	4.3087241	20645.5	0.0017093	0.95	4.1419500	4.7526500
thresh_age[1,1]	1.0001701	1.0005438	24.4985116	22.2249000	20.4567212	39871.2	0.0355846	0.95	16.2662000	43.9112000
thresh_age[1,2]	1.0001161	1.0004610	32.4935868	29.7970000	26.4683575	38328.5	0.0439882	0.95	22.0274000	53.7871000
thresh_age[1,3]	1.0000758	1.0003202	37.3680496	34.6505000	30.4520086	36900.7	0.0476037	0.95	25.6651000	58.9331000
thresh_age[1,4]	1.0006462	1.0010054	55.9588062	53.0235000	47.2562483	26303.6	0.0741310	0.95	40.6630000	79.8910000
thresh_age[1,5]	1.0091426	1.0094833	87.2204987	78.7146000	80.6323767	35553.8	0.3707692	0.95	62.3930000	114.9430000
age.s[1]	1.0000126	1.0001064	63.3652305	62.7433000	61.4830424	48297.7	0.0602587	0.95	39.1716000	89.5932000
age.s[2]	1.0008951	1.0017846	23.6481934	19.9585000	16.5474900	24442.6	0.0733280	0.95	15.0000000	48.0354000
age.s[3]	1.0000576	1.0001999	63.3586154	62.7031000	61.0824854	48184.6	0.0604572	0.95	38.7692000	89.5199000
age.s[4]	1.0001954	1.0007349	48.1141349	45.4832000	42.1136196	35324.8	0.0763682	0.95	24.8056000	79.5290000
age.s[5]	1.0001229	1.0002084	48.1350812	45.5207000	40.3762640	35388.8	0.0761106	0.95	24.5406000	78.7069000
age.s[6]	1.0000793	1.0001766	48.0722782	45.4766000	39.9673487	34603.5	0.0768596	0.95	24.3813000	78.9332000
age.s[7]	1.0004122	1.0011274	38.2601033	34.4189000	30.0824761	27618.1	0.0834891	0.95	18.1352000	70.2581000
age.s[8]	1.0000680	1.0002109	63.2823702	62.5882000	57.8683297	47798.0	0.0608316	0.95	39.9624000	90.9248000
age.s[9]	1.0003151	1.0007830	32.2302970	28.1180000	24.4869682	25690.6	0.0844489	0.95	15.0070000	62.9666000
age.s[10]	1.0000754	1.0003167	38.1829759	34.3481000	30.2688924	28440.4	0.0819938	0.95	18.0336000	70.0407000
age.s[11]	1.0002744	1.0009308	48.1333629	45.5643000	41.4358737	34613.8	0.0768677	0.95	24.6248000	79.1713000
age.s[12]	1.0002589	1.0005675	38.2824718	34.4009000	30.0713213	28420.7	0.0825652	0.95	18.4203000	71.0422000
age.s[13]	1.0001226	1.0004678	78.1010880	78.8149000	80.5437406	48494.8	0.0520932	0.95	58.3949000	99.9594000
age.s[14]	1.0003284	1.0004759	23.6094951	19.9638000	16.3637262	25028.4	0.0717418	0.95	15.0000000	47.8649000
age.s[15]	0.9999542	0.9999888	63.3333373	62.6755000	61.4445508	46120.6	0.0617957	0.95	39.5674000	90.3636000
age.s[16]	1.0001067	1.0003286	48.1402106	45.5828000	39.8691014	33883.8	0.0780348	0.95	24.9114000	79.5804000

age.s[17]	0.9999582	0.9999837	38.2643103	34.3584000	30.3573908	28210.3	0.0826964	0.95	19.0076000	71.1926000
age.s[18]	0.9999961	1.0001046	48.1127015	45.4641000	41.7091003	34927.0	0.0764277	0.95	24.6174000	79.2208000
age.s[19]	1.0000258	1.0000383	48.0790456	45.4342000	40.5697284	34171.3	0.0776484	0.95	24.5035000	79.0452000
age.s[20]	0.9999585	0.9999614	63.3853932	62.7202000	60.5819070	47361.6	0.0612314	0.95	39.6383000	90.3078000
age.s[21]	0.9999631	0.9999827	38.2281997	34.4144000	29.7209455	28428.7	0.0821726	0.95	18.8551000	70.5313000
age.s[22]	1.0005744	1.0012681	23.6078487	19.9515000	16.4006899	24189.3	0.0731478	0.95	15.0000000	47.7168000
age.s[23]	1.0001125	1.0001220	78.1738046	79.0053000	81.2081151	48620.2	0.0520176	0.95	58.3328000	99.9110000
age.s[24]	1.0000549	1.0001789	78.1491659	78.8274000	78.7721914	49116.4	0.0516666	0.95	58.3634000	99.8775000
age.s[25]	1.0001783	1.0003493	23.6000663	19.9851000	16.2261292	23981.9	0.0731698	0.95	15.0001000	47.5671000
age.s[26]	0.9999778	1.0000690	63.4069126	62.6927000	61.4276749	46971.8	0.0611391	0.95	39.5825000	90.0544000
age.s[27]	1.0000299	1.0002619	48.1451332	45.5866000	39.7860092	35833.5	0.0759233	0.95	24.4157000	79.1855000
age.s[28]	0.9999755	1.0000055	48.0589553	45.4105000	40.0987199	34143.9	0.0775785	0.95	24.2077000	78.7681000
age.s[29]	1.0002943	1.0006540	23.5969863	19.9380000	16.3965144	25087.2	0.0719434	0.95	15.0002000	47.6217000
age.s[30]	1.0001245	1.0004840	48.1188735	45.5073000	40.6748512	35320.0	0.0764978	0.95	25.0467000	79.7144000
age.s[31]	1.0000153	1.0000935	48.1262535	45.4729000	40.9491246	33661.5	0.0782063	0.95	25.0043000	79.6169000
age.s[32]	1.0001247	1.0004448	38.2918033	34.4044000	29.9141757	28570.4	0.0824360	0.95	18.3373000	70.6365000
age.s[33]	1.0000240	1.0001351	48.0698790	45.4663000	41.0684187	35198.4	0.0764080	0.95	24.5763000	79.1047000
age.s[34]	1.0001056	1.0004611	63.3029706	62.6377000	61.3095902	46405.4	0.0612443	0.95	39.4193000	89.5683000
age.s[35]	1.0000078	1.0000153	63.3286036	62.6386000	59.5926201	45581.7	0.0619118	0.95	39.1663000	89.6233000
age.s[36]	0.9999973	1.0000248	78.2021956	78.8785000	78.6925033	47760.4	0.0524107	0.95	58.2041000	99.7366000
age.s[37]	1.0006241	1.0012651	23.6840306	20.0443000	16.3435183	25075.8	0.0723780	0.95	15.0001000	48.1280000
age.s[38]	1.0000148	1.0002083	48.0549344	45.4499000	40.6285045	35753.0	0.0756972	0.95	24.8958000	79.4452000
age.s[39]	1.0006013	1.0010188	23.6652152	19.9661000	16.3222090	24242.4	0.0731261	0.95	15.0002000	48.4372000
age.s[40]	1.0003247	1.0007645	32.2012605	28.0838000	24.1889134	25627.1	0.0847584	0.95	15.0045000	63.2000000
age.s[41]	0.9999789	1.0000354	38.2138999	34.3448000	30.1680660	28245.2	0.0827575	0.95	18.3151000	70.1399000
age.s[42]	1.0001005	1.0004620	48.1112279	45.4581000	40.6163925	33262.4	0.0785339	0.95	24.8084000	79.5914000
age.s[43]	0.9999688	0.9999907	63.3110879	62.6231000	57.8323124	46283.1	0.0616841	0.95	39.6501000	90.3305000
age.s[44]	1.0003970	1.0005020	32.1896827	28.1213000	24.5425814	25714.3	0.0841070	0.95	15.0048000	62.6843000
age.s[45]	0.9999874	1.0000715	78.2596174	78.9853000	80.2003418	50001.0	0.0514067	0.95	58.3957000	99.9603000
age.s[46]	1.0001191	1.0005097	63.3091892	62.6155000	61.7186107	47418.0	0.0611491	0.95	39.7531000	90.6318000
age.s[47]	1.0002414	1.0007217	48.1158111	45.5252000	39.5056822	35753.5	0.0754984	0.95	25.1386000	79.3209000
age.s[48]	1.0000818	1.0004183	48.1464743	45.5955000	40.1759815	35621.5	0.0758979	0.95	25.0743000	79.3410000
age.s[49]	1.0005247	1.0010718	23.6571481	20.0318000	16.4069827	23818.7	0.0735887	0.95	15.0001000	48.0548000
age.s[50]	1.0001251	1.0004249	38.2276063	34.4100000	29.7226688	28113.4	0.0827928	0.95	18.5520000	70.4448000
age.s[51]	0.9999518	0.9999592	63.3493144	62.6798000	59.5623134	46465.8	0.0615200	0.95	38.5146000	89.1864000
age.s[52]	1.0006221	1.0011022	23.6194421	19.9291000	16.3955963	24249.6	0.0733126	0.95	15.0000000	47.7671000
age.s[53]	1.0001444	1.0005638	48.1233108	45.5307000	41.2912908	34807.9	0.0769782	0.95	24.6947000	79.3471000
age.s[54]	1.0001259	1.0004974	48.1618673	45.4867000	41.4605070	34426.4	0.0772994	0.95	24.5754000	78.9356000
age.s[55]	1.0000376	1.0001956	48.0911923	45.4747000	40.9988879	34303.6	0.0774406	0.95	24.2100000	78.7391000

age.s[56]	1.0000231	1.0001513	48.1483500	45.5671000	40.4102332	35734.9	0.0756501	0.95	24.4746000	78.8921000
age.s[57]	1.0003425	1.0008470	32.1566272	28.0878000	24.5405618	26103.4	0.0834435	0.95	15.0558000	62.7787000
age.s[58]	1.0000627	1.0001779	38.2547819	34.3654000	30.0924850	28321.6	0.0827890	0.95	18.4369000	70.7335000
age.s[59]	1.0002513	1.0004340	23.6647401	19.9870000	16.4860095	24423.5	0.0729944	0.95	15.0000000	47.8344000
age.s[60]	1.0001422	1.0005686	78.1302960	78.8686000	81.1967064	50001.0	0.0511210	0.95	58.4275000	99.9922000
age.s[61]	1.0000558	1.0003448	48.1520918	45.5180000	40.9870907	34459.9	0.0773423	0.95	24.4113000	79.0171000
age.s[62]	0.9999657	1.0000312	48.0738818	45.4851000	41.6054295	34710.4	0.0763080	0.95	25.2642000	79.3491000
age.s[63]	1.0002136	1.0005660	32.1551887	27.9858000	24.3728789	25297.5	0.0853355	0.95	15.0393000	63.1759000
age.s[64]	1.0002603	1.0008913	48.0908033	45.5439000	39.1248630	34984.3	0.0765781	0.95	24.8711000	79.3721000
age.s[65]	1.0001027	1.0003215	63.3804955	62.7394000	58.7330792	45289.4	0.0625499	0.95	39.0947000	89.7614000
age.s[66]	1.0001616	1.0002943	63.2099786	62.5568000	59.1861864	46644.5	0.0616161	0.95	39.8032000	90.4890000
age.s[67]	1.0000997	1.0004598	78.1784951	78.9625000	80.3954745	48895.7	0.0521605	0.95	58.2133000	99.9999000
age.s[68]	1.0000573	1.0003057	48.0823500	45.4823000	41.5799444	34276.6	0.0773762	0.95	25.0143000	79.8241000
age.s[69]	1.0001033	1.0004113	63.3248323	62.6756000	61.4943022	45424.8	0.0620569	0.95	39.5167000	90.0119000
age.s[70]	1.0002941	1.0006818	23.6312588	19.9695000	16.3886828	23908.3	0.0738027	0.95	15.0003000	47.8174000
age.s[71]	1.0000802	1.0001549	63.2840994	62.6539000	59.4239637	45765.6	0.0620604	0.95	39.0448000	89.8982000
age.s[72]	1.0001307	1.0003724	48.1246099	45.5091000	40.5068235	34935.9	0.0763952	0.95	25.4573000	79.8572000
age.s[73]	1.0014922	1.0022134	23.6545350	20.0093000	16.3855231	24603.9	0.0728654	0.95	15.0003000	48.0075000
age.s[74]	1.0001823	1.0005021	38.2527307	34.4427000	29.5255397	27790.2	0.0831228	0.95	18.2299000	70.0357000
age.s[75]	1.0000851	1.0002770	78.1692829	78.9012000	80.1695702	50001.0	0.0514572	0.95	58.3204000	99.9650000
age.s[76]	1.0003494	1.0007169	38.2113904	34.4176000	30.0897134	27141.6	0.0836519	0.95	18.4332000	69.7960000
age.s[77]	1.0002428	1.0009395	48.2236778	45.6516000	40.8525723	34093.9	0.0776324	0.95	24.9140000	79.5500000
age.s[78]	1.0000281	1.0002056	63.2613712	62.5537000	60.7659246	47766.4	0.0605800	0.95	39.5115000	90.2389000
age.s[79]	1.0000058	1.0000338	78.2049032	78.9799000	82.3584240	48786.2	0.0518204	0.95	58.3181000	99.9160000
age.s[80]	1.0000403	1.0001367	48.1851770	45.6006000	40.9803480	34906.4	0.0767926	0.95	25.1049000	79.7084000
age.s[81]	1.0000553	1.0001653	63.4099119	62.7387000	61.2858634	46955.3	0.0611972	0.95	39.5852000	90.2880000
age.s[82]	1.0004228	1.0006712	32.1640270	28.0932000	24.3747360	25493.7	0.0846221	0.95	15.0088000	62.7523000
age.s[83]	1.0003162	1.0006089	32.2283418	28.1752000	24.4963556	25217.0	0.0847961	0.95	15.0021000	62.8086000
age.s[84]	1.0002241	1.0006763	48.1646194	45.4958000	40.9724278	34571.3	0.0773255	0.95	24.7883000	79.6804000
age.s[85]	1.0001186	1.0004549	48.1077034	45.5112000	41.3148149	34997.2	0.0763099	0.95	25.0256000	79.2841000
age.s[86]	1.0002396	1.0006094	32.2519789	28.0792000	24.1888179	25103.9	0.0855790	0.95	15.0220000	63.2219000
age.s[87]	1.0000111	1.0001160	78.2056940	78.9608000	79.8779678	49016.9	0.0518284	0.95	58.2155000	99.9261000
age.s[88]	1.0000948	1.0005107	78.2314335	78.9780000	79.9573783	49359.0	0.0512118	0.95	58.5095000	99.9456000
age.s[89]	1.0000632	1.0002689	63.2990273	62.5856000	60.2235357	45132.3	0.0628474	0.95	38.9926000	90.1873000
age.s[90]	1.0006591	1.0010196	23.6057156	20.0009000	16.5614496	25148.1	0.0715188	0.95	15.0002000	47.6702000
age.s[91]	0.9999842	1.0000549	48.0939295	45.6032000	40.7578147	34533.0	0.0770371	0.95	25.0004000	79.5664000
age.s[92]	1.0007670	1.0012585	23.5885307	20.0008000	16.3789526	24456.9	0.0718239	0.95	15.0001000	47.1555000
age.s[93]	1.0000277	1.0001575	48.0960340	45.4385000	41.8963296	33839.4	0.0782016	0.95	24.7152000	79.3856000
age.s[94]	0.9999972	1.0001105	48.0431361	45.4851000	40.7596162	33421.6	0.0778416	0.95	25.1330000	79.1358000

age.s[95]	1.0001782	1.0006670	63.3330343	62.6678000	60.5762031	46577.2	0.0616870	0.95	38.9886000	90.1375000
age.s[96]	1.0000350	1.0001817	32.1720284	28.0722000	24.2025909	26052.7	0.0839753	0.95	15.0316000	63.0807000
age.s[97]	1.0000593	1.0000700	63.3363413	62.6897000	60.0520249	43770.8	0.0635215	0.95	39.4424000	90.3191000
age.s[98]	1.0005105	1.0012174	32.2418671	28.0954000	24.3221355	24851.9	0.0861219	0.95	15.0269000	63.0449000
age.s[99]	0.9999984	1.0000052	63.3684314	62.6206000	61.1889679	48177.1	0.0605335	0.95	39.5193000	90.4333000
age.s[100]	1.0000575	1.0003040	32.1877658	28.0753000	24.7826295	25985.3	0.0836860	0.95	15.0333000	62.7612000
age.s[101]	1.0000473	1.0002531	78.2615192	78.9633000	78.0459777	49113.7	0.0517308	0.95	58.3317000	99.8741000
age.s[102]	1.0000775	1.0001937	48.1479786	45.5667000	41.1044930	34458.7	0.0771427	0.95	25.1170000	79.5679000
age.s[103]	1.0000085	1.0001332	78.1291213	78.8957000	80.6701578	50001.0	0.0513194	0.95	58.1463000	99.8354000
age.s[104]	1.0000796	1.0001985	63.2963304	62.7096000	61.2627352	46457.6	0.0618040	0.95	39.3535000	90.2094000
age.s[105]	0.9999760	0.9999873	63.3120047	62.6214000	61.8052653	47517.5	0.0609573	0.95	39.0233000	90.0198000
age.s[106]	1.0001423	1.0005040	38.2763462	34.4034000	29.8723452	28334.4	0.0825983	0.95	18.7155000	70.9482000
age.s[107]	1.0000935	1.0003986	48.1036028	45.4895000	40.7361446	34982.6	0.0769113	0.95	24.8027000	79.5382000
age.s[108]	1.0001786	1.0004958	63.3142466	62.7165000	59.6120564	47610.6	0.0606895	0.95	39.2869000	89.8584000
age.s[109]	1.0000109	1.0001451	63.2917315	62.6401000	59.3057289	46957.6	0.0614057	0.95	38.8522000	89.6610000
age.s[110]	0.9999981	1.0000652	48.0707068	45.4728000	40.7522248	34493.7	0.0770418	0.95	24.7785000	79.2828000
age.s[111]	1.0001309	1.0004973	32.1709606	28.0365000	24.8068005	26011.7	0.0839286	0.95	15.0404000	63.2266000
age.s[112]	1.0002927	1.0006083	32.1148347	28.0454000	24.9323509	26138.2	0.0826697	0.95	15.0057000	62.4444000
age.s[113]	1.0001077	1.0002808	23.5680985	19.9425000	16.3897991	25411.8	0.0710667	0.95	15.0005000	48.0463000
age.s[114]	1.0003451	1.0006943	78.2305336	78.9751000	79.2142899	52883.4	0.0497037	0.95	58.4554000	99.9978000
age.s[115]	1.0000230	1.0002119	48.1274667	45.5216000	41.0669624	35716.0	0.0756956	0.95	24.6061000	78.9721000
age.s[116]	1.0001762	1.0004534	63.3540316	62.6806000	60.9180995	46364.8	0.0617988	0.95	39.4941000	90.1352000
age.s[117]	1.0002693	1.0010771	48.0450833	45.4661000	41.5297701	35816.5	0.0751063	0.95	24.1490000	78.3968000
age.s[118]	1.0000290	1.0002463	38.2174334	34.2925000	29.6281827	27960.0	0.0830605	0.95	18.2460000	70.5893000
age.s[119]	1.0004673	1.0008262	32.1307653	28.0592000	24.3461323	26203.9	0.0833638	0.95	15.0004000	62.8073000
age.s[120]	1.0000206	1.0001414	48.1136536	45.4914000	41.1245709	34452.7	0.0771718	0.95	25.1377000	79.5496000
age.s[121]	1.0001967	1.0002665	63.3108905	62.5550000	58.1119067	47017.4	0.0610915	0.95	39.4507000	90.0957000
age.s[122]	1.0000033	1.0001497	48.0458464	45.4204000	41.5228693	34908.1	0.0766597	0.95	23.9757000	78.7649000
age.s[123]	1.0000404	1.0001584	63.4196926	62.7630000	60.2597096	44734.5	0.0626945	0.95	39.0701000	89.6507000
age.s[124]	1.0001313	1.0004196	63.3348798	62.7048000	58.1443399	45655.5	0.0620516	0.95	39.4078000	90.1515000
age.s[125]	1.0000134	1.0001759	63.3873123	62.6897000	60.7192061	46343.0	0.0615517	0.95	39.4172000	90.0868000
age.s[126]	0.9999859	1.0000788	48.0787872	45.4107000	40.8691373	33700.8	0.0780740	0.95	24.7396000	79.0382000
age.s[127]	0.9999584	0.9999751	63.3127822	62.6489000	60.5607314	46168.0	0.0617126	0.95	39.3005000	89.8972000
age.s[128]	1.0000985	1.0003345	63.4363101	62.7752000	61.0704957	45338.1	0.0623266	0.95	39.4645000	90.2009000
age.s[129]	1.0004499	1.0006083	23.6474142	19.9823000	16.3884881	25034.8	0.0721853	0.95	15.0000000	48.0065000
age.s[130]	1.0002029	1.0003116	32.1982291	28.1026000	24.7037164	26328.8	0.0833096	0.95	15.0005000	63.1289000
age.s[131]	1.0000040	1.0001394	63.3724431	62.7176000	58.8198737	47869.9	0.0608939	0.95	39.1417000	89.8854000
age.s[132]	1.0001850	1.0006120	48.0544283	45.3840000	41.0431453	35302.0	0.0764376	0.95	24.4710000	79.3802000
age.s[133]	1.0005340	1.0007326	23.6003526	20.0269000	16.3916614	24344.3	0.0720480	0.95	15.0001000	47.1247000

age.s[134]	1.0000130	1.0001894	63.3053080	62.6229000	59.6305088	46576.9	0.0613865	0.95	39.2329000	89.6552000
age.s[135]	1.0000382	1.0002071	32.1996317	28.1032000	24.7123310	25682.2	0.0845580	0.95	15.0480000	62.9078000
age.s[136]	1.0003795	1.0009074	38.2508061	34.3864000	29.9042758	28152.7	0.0825090	0.95	18.9662000	70.9036000
age.s[137]	1.0000712	1.0004043	48.0805514	45.5140000	40.7709804	34428.4	0.0768278	0.95	24.1778000	78.3691000
age.s[138]	1.0000328	1.0000695	63.3626825	62.7792000	60.1894302	48431.2	0.0603320	0.95	39.2653000	90.2545000
age.s[139]	1.0004932	1.0013551	32.2011762	28.1197000	24.5463295	25247.8	0.0850785	0.95	15.0070000	62.9453000
age.s[140]	1.0000371	1.0002828	48.1320409	45.5133000	40.1853057	34570.5	0.0766595	0.95	25.3701000	79.6473000
age.s[141]	0.9999790	1.0000602	63.2672182	62.5345000	59.7864895	48082.1	0.0602656	0.95	39.4462000	89.9274000
age.s[142]	0.9999900	1.0000931	63.3627163	62.7614000	59.2427030	47312.8	0.0607665	0.95	39.3028000	89.7657000
age.s[143]	1.0000427	1.0003001	63.2850932	62.6180000	61.6991555	46909.4	0.0612984	0.95	39.4795000	90.4192000
age.s[144]	0.9999716	1.0000517	63.3125489	62.6595000	58.0694626	45889.2	0.0620602	0.95	39.0175000	89.6543000
age.s[145]	1.0006336	1.0009533	23.5884790	19.9496000	16.4014587	25324.0	0.0707991	0.95	15.0000000	47.6960000
age.s[146]	1.0005711	1.0008468	23.5790120	19.9443000	16.3881159	24734.4	0.0718710	0.95	15.0000000	47.7598000
age.s[147]	1.0002992	1.0006471	48.0873512	45.5011000	40.8398061	35182.4	0.0764917	0.95	24.7964000	79.4705000
age.s[148]	1.0000762	1.0003156	47.9984925	45.3817000	41.5310006	35973.2	0.0752682	0.95	24.0639000	78.6545000
age.s[149]	1.0000374	1.0000410	63.3649874	62.6860000	60.7846923	45458.0	0.0621615	0.95	40.0620000	90.7086000
age.s[150]	1.0000604	1.0001670	48.0974403	45.5114000	40.0232199	34754.4	0.0766092	0.95	24.5540000	79.1060000
age.s[151]	1.0004081	1.0007709	23.6868596	20.0311000	16.5228487	23781.4	0.0739511	0.95	15.0013000	47.8514000
age.s[152]	1.0000444	1.0002863	63.2886758	62.6457000	61.2012771	47962.9	0.0607011	0.95	39.6580000	90.5698000
age.s[153]	0.9999951	1.0001262	63.4135772	62.8328000	58.9374663	47589.1	0.0608357	0.95	38.7862000	89.3567000
age.s[154]	1.0001492	1.0003562	48.0700341	45.4027000	42.0863179	34932.5	0.0764586	0.95	24.6970000	79.0192000
age.s[155]	0.9999714	1.0000368	48.2048513	45.7002000	41.3553133	34093.0	0.0774453	0.95	24.3859000	78.9899000
age.s[156]	0.9999818	0.9999932	63.2659162	62.5261000	61.2489325	47216.0	0.0609430	0.95	39.1696000	89.8606000
age.s[157]	1.0000468	1.0001284	63.2706379	62.4654000	58.5411042	47373.4	0.0608301	0.95	39.2122000	89.7213000
age.s[158]	1.0001820	1.0003030	78.1862003	78.8916000	79.6974153	48874.7	0.0517285	0.95	58.2625000	99.8820000
age.s[159]	1.0003748	1.0004194	23.5883532	19.9283000	16.4119361	24288.9	0.0734181	0.95	15.0000000	47.9474000
age.s[160]	1.0003280	1.0012477	48.0182290	45.4070000	40.0510055	35358.0	0.0757433	0.95	24.9946000	79.4508000
age.s[161]	0.9999752	1.0000596	63.2924818	62.6534000	59.2115577	47199.0	0.0613548	0.95	39.2716000	90.4026000
age.s[162]	0.9999590	0.9999931	38.2010022	34.3070000	29.7450874	28607.4	0.0821494	0.95	18.5126000	70.5309000
age.s[163]	0.9999866	1.0000495	63.2633350	62.5355000	59.6302362	46089.3	0.0615846	0.95	38.7312000	89.2336000
age.s[164]	1.0000186	1.0000390	48.0771196	45.4556000	40.5909248	34842.0	0.0765069	0.95	24.6073000	78.8485000
age.s[165]	1.0001771	1.0004107	32.2404528	28.1900000	24.2040686	25297.7	0.0852828	0.95	15.0581000	63.1538000
age.s[166]	1.0000360	1.0002588	63.2477711	62.6122000	60.9809383	46771.8	0.0615395	0.95	39.3794000	90.1523000
age.s[167]	1.0000490	1.0002836	78.3307861	79.0834000	80.8183613	49331.6	0.0514843	0.95	58.5616000	99.9987000
age.s[168]	1.0000314	1.0000759	63.3605566	62.6768000	60.8866289	47203.8	0.0609170	0.95	39.5215000	89.9724000
age.s[169]	1.0002400	1.0008781	78.1692410	78.9004000	79.2459623	47344.5	0.0527183	0.95	58.3200000	99.9893000
age.s[170]	1.0000560	1.0001800	32.1660097	28.0689000	24.5189647	26113.0	0.0835631	0.95	15.0656000	62.7265000
age.s[171]	1.0001256	1.0005230	48.1169499	45.4972000	40.9792415	33405.1	0.0784602	0.95	24.8407000	79.3834000
age.s[172]	1.0001390	1.0005454	48.0737227	45.5237000	40.4550409	34773.1	0.0762876	0.95	25.4696000	79.5223000

age.s[173]	0.9999669	1.0000132	78.2419891	79.0297000	80.7721997	49000.2	0.0518113	0.95	58.3501000	99.9198000
age.s[174]	0.9999829	1.0000069	63.2949690	62.6046000	58.0956095	44924.8	0.0629255	0.95	39.4855000	90.2671000
age.s[175]	1.0000311	1.0001097	48.0695732	45.4009000	41.1647928	35331.0	0.0760865	0.95	25.3032000	79.4645000
age.s[176]	0.9999888	1.0000812	48.0820426	45.4074000	41.6332370	34633.3	0.0772615	0.95	24.3254000	78.9081000
age.s[177]	1.0001399	1.0006543	48.1469044	45.4982000	40.3318372	34990.9	0.0765255	0.95	24.6181000	78.9454000
age.s[178]	1.0001670	1.0005565	63.2930828	62.6588000	58.3506470	45425.1	0.0624353	0.95	38.7757000	89.7481000
age.s[179]	0.9999890	1.0000649	78.1933556	78.9268000	81.5759583	48710.8	0.0519328	0.95	58.1193000	99.6765000
age.s[180]	0.9999608	0.9999925	63.3114561	62.6464000	59.0223686	46339.8	0.0620234	0.95	38.6794000	89.5316000
age.s[181]	1.0002121	1.0005614	32.1804970	28.0776000	24.7111916	25239.6	0.0849828	0.95	15.0117000	62.9012000
age.s[182]	1.0000624	1.0003508	63.3599621	62.6762000	61.4105001	46042.3	0.0618125	0.95	39.9447000	90.5888000
age.s[183]	1.0008361	1.0012243	23.6840963	19.9935000	16.3918885	24225.4	0.0737772	0.95	15.0000000	48.2026000
age.s[184]	0.9999663	0.9999869	48.0875649	45.5658000	40.6743601	35520.4	0.0759240	0.95	24.6853000	79.3379000
age.s[185]	1.0002125	1.0006718	38.2845903	34.4694000	30.2619018	28021.7	0.0828861	0.95	18.5551000	70.3603000
age.s[186]	1.0001463	1.0004983	48.0927846	45.4633000	40.5376796	35655.1	0.0756116	0.95	25.2567000	79.6483000
age.s[187]	0.9999953	1.0000774	63.2994446	62.6280000	60.5122127	45917.1	0.0617738	0.95	39.3885000	89.6988000
age.s[188]	1.0000687	1.0002793	32.2193689	28.0979000	24.3888032	25390.0	0.0855558	0.95	15.0311000	63.2515000
age.s[189]	1.0002702	1.0005784	32.1602430	28.0341000	24.4829009	26632.6	0.0828695	0.95	15.0909000	62.8751000
age.s[190]	1.0000739	1.0002472	48.1049476	45.5171000	39.9290781	34980.7	0.0767255	0.95	25.0110000	79.5786000
age.s[191]	1.0005474	1.0008929	32.2028763	28.1073000	24.5098543	26331.3	0.0829652	0.95	15.0092000	62.9300000
age.s[192]	0.9999809	1.0000501	63.3416393	62.6450000	59.8660794	47439.6	0.0609631	0.95	39.4711000	90.1436000
age.s[193]	1.0002542	1.0006294	32.1725923	28.0384000	24.1669271	25791.2	0.0845622	0.95	15.0486000	62.9764000
age.s[194]	1.0000926	1.0003693	63.3441550	62.7099000	61.4638342	45155.4	0.0625048	0.95	39.0182000	89.7069000
age.s[195]	1.0000614	1.0001093	48.0902305	45.4778000	40.7034394	34283.0	0.0771425	0.95	25.1694000	79.5053000
age.s[196]	1.0000017	1.0001410	48.1561137	45.5486000	41.1655109	34703.7	0.0774103	0.95	24.5327000	79.5471000
age.s[197]	1.0003933	1.0010140	32.1106918	28.0293000	24.3884480	26687.4	0.0824768	0.95	15.0077000	62.8387000
age.s[198]	1.0000233	1.0002223	78.2488011	78.9752000	78.6864758	49167.0	0.0517398	0.95	58.4268000	99.9501000
age.s[199]	1.0002379	1.0004814	23.6269171	19.9648000	16.3878223	23959.5	0.0737076	0.95	15.0004000	48.0199000
age.s[200]	1.0004837	1.0008419	23.6477790	19.9832000	16.3091757	23829.5	0.0740528	0.95	15.0001000	47.9287000

1.1.3 Allmales n = 100

Table 3: MCMC basic diagnostics Allmales sample size n = 100.

	PSRF Point est.	PSRF Upper C.I.	Mean	Median	Mode	ESS	MCSE	HDImass	HDIlow	HDIhigh
b	1.0008475	1.0017575	0.0344220	0.0292494	0.0221521	32519.9	0.0000867	0.95	0.0200001	0.0708998
a	1.0001873	1.0005865	0.0134317	0.0138719	0.0144568	43990.5	0.0000350	0.95	0.0001232	0.0244804
M	1.0001912	1.0005738	40.8250035	40.5048000	39.9752662	44141.4	0.1016625	0.95	2.4164100	77.2353000
beta0	1.0010515	1.0038521	-18.8447044	-18.8361500	-18.5484448	11833.5	0.0667177	0.95	-33.0283000	-4.0964900
beta	1.0011654	1.0042858	5.9575947	5.9461300	5.8266955	11051.7	0.0215014	0.95	1.3558400	10.3119000
thresh[1,1]	NaN	NaN	0.5000000	0.5000000	0.4997358	0.0	NaN	0.95	0.5000000	0.5000000
thresh[1,2]	1.0014853	1.0050696	2.5331484	2.4476550	2.2686363	12729.9	0.0078399	0.95	0.9887240	4.1861200
thresh[1,3]	1.0013972	1.0049174	3.3656950	3.2826800	3.2398601	11635.2	0.0110026	0.95	1.2754400	5.5763100
thresh[1,4]	1.0013460	1.0048202	4.9078772	4.8115500	4.6174219	11060.3	0.0166266	0.95	1.7821800	8.0711100
thresh[1,5]	1.0012412	1.0044895	7.1942718	7.0561800	6.9417809	11153.1	0.0238330	0.95	2.6918100	11.7176000
thresh_age_log[1,1]	1.0003526	1.0005403	3.2503059	3.1960600	3.1116664	68012.9	0.0010077	0.95	2.8996400	3.8649200
thresh_age_log[1,2]	1.0001913	1.0003827	3.6023408	3.5575350	3.4591983	60982.1	0.0009148	0.95	3.2504800	4.0947600
thresh_age_log[1,3]	1.0002287	1.0005355	3.7465979	3.7086450	3.6113683	52107.5	0.0009222	0.95	3.4133100	4.2003600
thresh_age_log[1,4]	1.0004302	1.0010634	4.0163113	3.9821800	3.9074602	32464.9	0.0011037	0.95	3.7042300	4.4112100
thresh_age_log[1,5]	1.0017500	1.0028307	4.4292219	4.3756350	4.3431538	22112.6	0.0019089	0.95	4.1126800	4.8198000
thresh_age[1,1]	1.0004777	1.0008408	26.7296304	24.4360000	22.2444346	58693.7	0.0314204	0.95	17.3553000	46.5635000
thresh_age[1,2]	1.0002786	1.0005674	37.6765968	35.0766000	31.3770424	55920.8	0.0388757	0.95	24.8346000	58.6926000
thresh_age[1,3]	1.0003178	1.0007223	43.3659540	40.7985500	36.5142698	48178.3	0.0444898	0.95	29.4184000	65.2235000
thresh_age[1,4]	1.0004954	1.0011419	56.7095714	53.6338000	48.7014257	32074.7	0.0748523	0.95	39.6829000	80.9706000
thresh_age[1,5]	1.0075055	1.0077592	91.3007740	79.4903000	72.0344242	59115.9	0.5047320	0.95	60.0863000	122.1890000
age.s[1]	1.0010670	1.0021398	25.1730582	21.1896000	16.6097135	28650.0	0.0725199	0.95	15.0000000	52.4147000
age.s[2]	1.0004004	1.0013316	50.9107696	48.4493000	43.8164388	52421.3	0.0611511	0.95	27.9080000	81.4457000
age.s[3]	1.0005132	1.0012768	36.0138068	32.0011500	28.1605451	33819.8	0.0770505	0.95	16.3025000	68.4609000
age.s[4]	1.0000947	1.0003569	63.4631947	62.8262500	62.5104710	84102.0	0.0460310	0.95	39.4652000	90.5178000
age.s[5]	1.0003191	1.0005978	43.4375691	39.9593000	35.4828413	40572.1	0.0702391	0.95	22.2433000	75.8053000
age.s[6]	1.0003419	1.0008713	36.0368665	32.0617000	27.2523491	34150.5	0.0767948	0.95	15.8755000	67.8085000
age.s[7]	1.0012185	1.0023543	25.1609020	21.1906500	16.6407951	27139.4	0.0747359	0.95	15.0001000	52.3481000
age.s[8]	1.0007492	1.0016274	36.0262051	32.0449500	28.0059768	34455.6	0.0763010	0.95	16.1737000	68.2029000
age.s[9]	1.0001231	1.0004062	78.0023965	78.7945500	78.6906883	89529.1	0.0388166	0.95	57.7166000	99.9828000
age.s[10]	1.0013982	1.0027633	25.1276585	21.2014000	16.6416767	28116.5	0.0726712	0.95	15.0000000	51.7989000
age.s[11]	1.0000362	1.0001748	63.5044319	62.8725500	60.9932278	85355.9	0.0456607	0.95	39.2313000	90.3777000
age.s[12]	1.0000332	1.0000756	63.4868185	62.7819000	60.8853460	81537.2	0.0470283	0.95	39.0736000	90.4367000
age.s[13]	1.0001211	1.0004925	63.5051973	62.8916500	62.1579532	83021.8	0.0465338	0.95	39.2772000	90.4949000
age.s[14]	1.0002935	1.0008926	50.9468932	48.5149000	42.3863477	52506.2	0.0613440	0.95	27.8506000	81.6450000
age.s[15]	1.0001847	1.0005505	50.9509519	48.5188500	42.7987375	51019.4	0.0623747	0.95	27.4912000	81.4386000
age.s[16]	0.9999961	1.0000566	78.1329700	78.9417000	80.2105668	90195.7	0.0385776	0.95	57.9596000	99.8814000

age.s[17]	1.0004295	1.0011134	43.4363317	40.0254000	34.6097536	39968.1	0.0706916	0.95	21.7021000	75.3972000
age.s[18]	1.0001020	1.0004318	78.1316197	78.9223000	79.3309223	87181.3	0.0392527	0.95	57.9609000	99.9998000
age.s[19]	1.0000239	1.0001387	63.5186290	62.8979500	58.8905967	84334.0	0.0461798	0.95	39.2009000	90.4540000
age.s[20]	1.0001004	1.0002599	50.9283101	48.4892500	43.4916640	49485.9	0.0632844	0.95	27.3657000	81.0815000
age.s[21]	1.0008557	1.0019453	36.0076798	32.0311000	27.4755220	33801.4	0.0772842	0.95	16.2577000	68.3954000
age.s[22]	1.0001247	1.0003412	78.0487514	78.8304000	78.8650990	83261.9	0.0402811	0.95	57.8988000	99.9819000
age.s[23]	1.0005217	1.0012535	36.0486739	32.0293500	27.6217881	32618.1	0.0785939	0.95	16.2377000	68.4071000
age.s[24]	1.0006866	1.0015230	36.0728582	32.0430500	27.7699293	33719.9	0.0774623	0.95	15.8098000	68.1515000
age.s[25]	1.0001690	1.0003537	63.4838420	62.8492500	60.4032263	82802.4	0.0466904	0.95	38.7522000	90.2109000
age.s[26]	1.0000792	1.0001490	63.5047776	62.9040000	62.1275941	82963.1	0.0464120	0.95	39.2815000	90.3421000
age.s[27]	1.0000357	1.0000598	63.4350289	62.8184500	60.3981388	83077.5	0.0464562	0.95	39.0227000	90.2270000
age.s[28]	1.0005082	1.0011719	36.0187668	32.0244500	27.4565349	34304.4	0.0765842	0.95	16.2926000	68.3800000
age.s[29]	1.0013284	1.0030512	25.1543687	21.2043000	16.7981889	27904.8	0.0732120	0.95	15.0002000	52.1481000
age.s[30]	0.9999862	1.0000006	63.5345946	62.8730500	60.5263560	84126.1	0.0461615	0.95	38.7147000	89.8730000
age.s[31]	1.0001815	1.0004486	63.5491387	62.9365500	61.0601604	79473.0	0.0475675	0.95	39.1180000	90.1836000
age.s[32]	1.0002077	1.0005526	50.9223345	48.4994500	42.9724072	49349.8	0.0631259	0.95	27.4155000	80.8659000
age.s[33]	1.0003923	1.0009058	50.9174853	48.4587000	42.6099532	50962.8	0.0621729	0.95	27.8955000	81.6068000
age.s[34]	1.0006404	1.0014312	36.0064410	31.9911500	28.1735898	32723.1	0.0784964	0.95	15.7762000	67.9651000
age.s[35]	1.0001456	1.0003795	78.0642792	78.8724000	79.9585259	85004.2	0.0398314	0.95	57.8423000	99.9000000
age.s[36]	1.0000482	1.0001116	63.4878404	62.8080000	61.1738084	82741.5	0.0466080	0.95	38.4875000	89.8660000
age.s[37]	1.0000235	1.0000903	63.5041544	62.9217500	61.0974587	80675.0	0.0470157	0.95	39.4154000	90.5255000
age.s[38]	1.0002087	1.0005695	50.9206019	48.5025000	44.0907493	50140.2	0.0625451	0.95	27.7655000	81.3439000
age.s[39]	1.0007058	1.0014576	25.1719333	21.1996500	16.6377716	27985.1	0.0734587	0.95	15.0000000	52.2864000
age.s[40]	1.0001908	1.0005186	78.0697488	78.8778500	80.7082324	86061.5	0.0395324	0.95	57.9956000	99.9996000
age.s[41]	1.0001676	1.0003919	43.4560318	39.9727500	35.1517593	39872.3	0.0710730	0.95	21.9246000	75.6869000
age.s[42]	1.0002487	1.0006018	50.9325226	48.5406500	43.6726825	52057.3	0.0613356	0.95	27.8825000	81.3914000
age.s[43]	1.0003692	1.0008975	50.9212134	48.4579000	43.1624637	51204.1	0.0619402	0.95	27.9579000	81.3870000
age.s[44]	1.0001583	1.0002014	63.5205139	62.8573000	61.2055100	80671.1	0.0470885	0.95	39.0874000	90.1311000
age.s[45]	1.0001807	1.0003116	63.5319225	62.9132000	59.4027770	82658.0	0.0465788	0.95	39.0558000	90.3885000
age.s[46]	1.0001885	1.0005930	50.9499170	48.5009000	43.0931117	50936.2	0.0621071	0.95	28.4347000	82.0624000
age.s[47]	1.0001947	1.0002960	63.5130234	62.9046000	60.7170057	89461.5	0.0448014	0.95	39.1229000	90.2869000
age.s[48]	1.0001158	1.0004156	63.5083975	62.8213000	58.8848481	82001.6	0.0466378	0.95	39.5593000	90.6630000
age.s[49]	1.0004063	1.0010086	36.0435384	32.0387000	27.8924270	33805.0	0.0769713	0.95	16.1049000	68.0430000
age.s[50]	1.0002817	1.0008431	50.9175658	48.5215000	43.2252511	51138.6	0.0619258	0.95	27.4960000	80.8491000
age.s[51]	1.0000874	1.0003327	63.5716239	62.9077500	60.7200536	79990.0	0.0474336	0.95	39.5192000	90.9387000
age.s[52]	1.0002121	1.0005348	50.9701879	48.5546000	43.6689693	51047.9	0.0622452	0.95	27.9250000	81.7568000
age.s[53]	1.0001628	1.0003903	63.4554779	62.8171500	60.2434403	80383.9	0.0473001	0.95	39.1174000	90.3445000
age.s[54]	1.0007714	1.0012803	25.1491607	21.1896000	16.6197735	28687.5	0.0723993	0.95	15.0001000	52.3617000
age.s[55]	1.0000499	1.0001969	63.4983571	62.8683000	62.5292585	82868.8	0.0463899	0.95	39.7075000	90.7711000

age.s[56]	1.0011437	1.0022389	25.1932702	21.2104000	16.6409961	29560.0	0.0715783	0.95	15.0000000	52.6687000
age.s[57]	1.0009995	1.0019052	25.1694687	21.1738000	16.6161630	28463.2	0.0725574	0.95	15.0002000	52.4880000
age.s[58]	1.0007014	1.0016069	35.9757704	31.9599500	27.8035492	33376.4	0.0775655	0.95	16.1436000	68.1929000
age.s[59]	1.0003686	1.0009027	43.4503954	40.0187000	34.9800075	37509.2	0.0731038	0.95	21.9755000	75.5577000
age.s[60]	1.0013956	1.0029900	25.1902282	21.2042500	16.5909907	28840.8	0.0724533	0.95	15.0001000	52.6593000
age.s[61]	1.0001888	1.0007038	63.5271519	62.9009000	60.1331310	83877.7	0.0463847	0.95	38.7930000	90.2082000
age.s[62]	1.0003029	1.0006937	43.5030843	40.0447500	35.1487214	39463.0	0.0714792	0.95	22.0412000	75.6748000
age.s[63]	1.0000666	1.0000707	63.4826401	62.8796500	59.4253164	84127.4	0.0462191	0.95	38.7371000	89.9217000
age.s[64]	1.0011244	1.0022061	25.1562158	21.2111000	16.7892802	29251.5	0.0714009	0.95	15.0000000	52.0173000
age.s[65]	1.0000769	1.0003070	78.0128510	78.8053500	80.0113733	85274.7	0.0398791	0.95	57.9150000	99.9781000
age.s[66]	1.0016189	1.0030394	25.1473456	21.1814500	16.6472844	28226.4	0.0729014	0.95	15.0002000	52.4476000
age.s[67]	1.0002405	1.0006523	50.9399086	48.4793000	42.9658046	51676.8	0.0615961	0.95	27.9621000	81.4852000
age.s[68]	1.0000416	1.0002172	78.0761026	78.8569000	80.1962029	88676.3	0.0389541	0.95	57.8046000	99.8603000
age.s[69]	1.0004038	1.0010846	36.0097596	32.0396000	27.8533690	32826.9	0.0781056	0.95	16.2182000	68.1366000
age.s[70]	1.0012451	1.0024305	25.1278824	21.1759000	16.6438813	28605.2	0.0722850	0.95	15.0001000	52.2406000
age.s[71]	1.0001106	1.0003024	50.9564995	48.5267000	43.3339872	48566.5	0.0635997	0.95	27.6703000	81.1853000
age.s[72]	1.0001235	1.0001935	63.5243609	62.8773500	59.6879443	83764.9	0.0464600	0.95	39.5353000	91.0165000
age.s[73]	1.0003005	1.0006539	50.9596093	48.5048500	44.2057797	49722.2	0.0629563	0.95	27.6354000	81.2104000
age.s[74]	1.0002398	1.0006840	43.4360294	39.9439500	34.8968793	40834.8	0.0700002	0.95	22.0158000	75.4363000
age.s[75]	1.0001720	1.0004155	50.9843133	48.5224500	43.8520898	50350.1	0.0625489	0.95	27.9505000	81.4389000
age.s[76]	0.9999875	0.9999902	63.4674210	62.8186500	59.9417586	83527.8	0.0462955	0.95	39.3612000	90.4446000
age.s[77]	1.0003783	1.0009470	43.4706143	40.0503500	34.7729075	39921.4	0.0708228	0.95	22.1892000	75.9065000
age.s[78]	1.0004613	1.0009715	36.0813001	32.0654000	27.7985352	33434.6	0.0777334	0.95	15.7601000	68.1017000
age.s[79]	1.0006026	1.0013501	36.0852424	32.0656500	27.6479911	34332.1	0.0767705	0.95	16.3473000	68.6099000
age.s[80]	1.0004907	1.0012581	36.0250414	31.9931500	27.8004735	33110.6	0.0779918	0.95	16.0623000	68.1921000
age.s[81]	1.0004744	1.0012286	36.0869648	32.0384000	28.2062606	32437.6	0.0790265	0.95	15.9284000	68.3286000
age.s[82]	1.0000624	1.0002401	63.4789097	62.8008500	59.8924701	84125.2	0.0462621	0.95	39.3779000	90.6726000
age.s[83]	1.0015290	1.0028776	25.1722008	21.1897500	16.6289608	28486.0	0.0727757	0.95	15.0001000	52.2321000
age.s[84]	1.0002417	1.0005757	50.8962668	48.4712500	43.8826907	52439.4	0.0613045	0.95	27.4866000	81.2864000
age.s[85]	1.0014027	1.0026023	25.1858130	21.2113000	16.7784929	28541.7	0.0725185	0.95	15.0001000	52.4240000
age.s[86]	1.0005796	1.0013192	36.0334880	32.0193500	27.7787264	33101.9	0.0781800	0.95	15.9714000	68.3154000
age.s[87]	1.0000203	1.0000981	63.4737118	62.8561000	61.7539085	86285.8	0.0455832	0.95	39.2341000	90.3962000
age.s[88]	0.9999878	1.0000150	78.0378255	78.8662500	81.3731301	89142.7	0.0388940	0.95	57.9580000	99.9982000
age.s[89]	1.0007270	1.0020253	36.0440094	32.0259500	27.4802523	32890.8	0.0784558	0.95	16.0165000	68.1304000
age.s[90]	1.0001476	1.0004378	50.9511311	48.5470000	43.8965433	49682.9	0.0630169	0.95	27.8818000	81.6548000
age.s[91]	1.0007499	1.0016538	25.1262775	21.1828000	16.4699650	28407.6	0.0726669	0.95	15.0002000	52.1008000
age.s[92]	1.0001086	1.0002519	63.5587607	62.9976000	60.4292557	87640.8	0.0452566	0.95	39.0109000	90.1390000
age.s[93]	1.0002217	1.0006168	50.9352453	48.4667000	43.8949296	50638.0	0.0626015	0.95	27.2842000	81.3176000
age.s[94]	1.0002516	1.0005087	50.9276444	48.5419000	44.5133563	49479.8	0.0629710	0.95	27.4003000	81.0789000

age.s[95]	1.0015886	1.0030549	25.1567572	21.1532000	16.6432063	29344.3	0.0717340	0.95	15.0002000	52.4690000
age.s[96]	1.0005326	1.0013540	36.0780282	32.1026000	26.9098929	33260.9	0.0778616	0.95	15.8182000	68.0362000
age.s[97]	1.0000440	1.0001661	63.5535707	62.9021000	61.2041118	81891.9	0.0469102	0.95	38.7597000	90.0123000
age.s[98]	1.0003816	1.0009784	43.4311725	40.0208000	35.3383915	39918.2	0.0707621	0.95	22.1806000	75.7889000
age.s[99]	1.0001147	1.0001214	78.0894447	78.9019000	80.5295712	85455.6	0.0396891	0.95	58.0003000	99.9981000
age.s[100]	1.0004451	1.0008412	43.4282397	40.0473500	35.4762669	40393.6	0.0702609	0.95	21.5970000	75.1073000

1.1.4 Allmales n = 50

Table 4: MCMC basic diagnostics Allmales sample size n = 50.

	PSRF Point est.	PSRF Upper C.I.	Mean	Median	Mode	ESS	MCSE	HDImass	HDIlow	HDIhigh
b	0.9999955	1.0000474	0.0359104	0.0305882	0.0224896	60432.3	0.0000662	0.95	0.0200001	0.0735623
a	1.0000015	1.0000854	0.0126087	0.0126856	0.0007853	70163.7	0.0000280	0.95	0.0001232	0.0242792
M	1.0000017	1.0000859	43.1854546	43.7740000	49.6112085	70226.3	0.0812589	0.95	7.7376900	81.9893000
beta0	1.0000141	1.0000492	-18.3205683	-18.3222500	-17.8855468	23455.3	0.0481619	0.95	-32.7806000	-3.3350500
beta	1.0000182	1.0000624	5.6562992	5.6452650	5.5482449	22159.6	0.0150619	0.95	1.0633400	9.9230200
thresh[1,1]	NaN	NaN	0.5000000	0.5000000	0.4997358	0.0	NaN	0.95	0.5000000	0.5000000
thresh[1,2]	1.0001050	1.0004448	2.1612506	2.0299800	1.7866888	30871.2	0.0048164	0.95	0.8236000	3.8052600
thresh[1,3]	1.0000836	1.0003463	2.4718350	2.3377200	2.2071844	29031.1	0.0056485	0.95	0.9020950	4.3172000
thresh[1,4]	0.9999981	1.0000658	4.9447415	4.7807100	4.6775115	24656.5	0.0119089	0.95	1.7659100	8.3947800
thresh[1,5]	0.9999949	1.0000465	7.4041498	7.1629350	6.9098652	24941.2	0.0166817	0.95	2.9073500	12.3048000
thresh_age_log[1,1]	1.0001695	1.0002925	3.3302321	3.2875900	3.1845628	91820.4	0.0009297	0.95	2.9318800	3.9544200
thresh_age_log[1,2]	1.0000450	1.0001186	3.6360082	3.6031300	3.5209876	88562.1	0.0008075	0.95	3.2400900	4.1391000
thresh_age_log[1,3]	1.0000143	1.0000722	3.6933097	3.6619850	3.5710973	83484.5	0.0008076	0.95	3.3125800	4.1847300
thresh_age_log[1,4]	1.0000659	1.0000831	4.1566318	4.1241350	4.0519692	51346.0	0.0010408	0.95	3.7892300	4.5611600
thresh_age_log[1,5]	1.0000583	1.0000752	4.6457107	4.5576700	4.5071883	42389.6	0.0021377	0.95	4.1902000	5.2195100
thresh_age[1,1]	1.0000174	1.0000654	29.0785058	26.7782500	23.7356971	89396.1	0.0286127	0.95	17.7793000	50.3601000
thresh_age[1,2]	1.0000111	1.0000687	39.0815777	36.7129500	32.7924741	85846.0	0.0337490	0.95	24.6733000	61.4091000
thresh_age[1,3]	1.0000152	1.0000651	41.3173429	38.9384500	34.6281293	80799.4	0.0356953	0.95	26.2309000	63.6968000
thresh_age[1,4]	1.0042222	1.0042303	66.2552504	61.8143500	57.4395828	68400.5	0.1190408	0.95	42.4980000	93.0457000
thresh_age[1,5]	1.2501064	1.2506512	190.6379777	95.3611500	49.5814085	100002.0	15.3578859	0.95	63.5567000	181.3190000
age.s[1]	0.9999808	0.9999810	53.0075271	51.1322500	46.8516611	81300.5	0.0524137	0.95	27.5317000	84.1004000
age.s[2]	0.9999713	0.9999726	52.9602299	51.0893500	47.0560595	81897.0	0.0522541	0.95	27.0737000	83.5635000
age.s[3]	1.0000311	1.0000538	27.1429896	22.6167000	17.0401223	53657.3	0.0585009	0.95	15.0003000	58.2140000
age.s[4]	1.0000151	1.0000378	68.7637717	68.8743500	66.5070485	100002.0	0.0420410	0.95	44.5712000	94.8456000
age.s[5]	1.0000163	1.0000230	27.1514889	22.6322000	16.9079396	54447.4	0.0582010	0.95	15.0000000	58.5680000
age.s[6]	1.0000349	1.0000518	82.2579074	83.6897000	85.7641093	81261.0	0.0385127	0.95	62.3486000	99.9990000
age.s[7]	0.9999834	1.0000066	53.0501740	51.0579000	46.1533430	78269.0	0.0535389	0.95	27.2172000	83.8039000
age.s[8]	1.0000589	1.0001234	27.1345711	22.6064500	16.8867086	53697.7	0.0584955	0.95	15.0001000	58.2264000
age.s[9]	0.9999945	1.0000055	52.9609575	51.0094000	45.7469157	82089.8	0.0522830	0.95	27.4541000	84.1764000
age.s[10]	1.0000935	1.0002218	38.3278911	34.1853000	29.1791931	60506.9	0.0608892	0.95	16.6312000	71.7707000
age.s[11]	0.9999880	1.0000359	43.7483225	40.1405500	34.0633939	65827.2	0.0582034	0.95	21.2906000	77.5963000
age.s[12]	1.0000088	1.0000753	38.3637232	34.2330500	29.0030541	58890.4	0.0619012	0.95	16.7042000	72.1347000
age.s[13]	1.0000218	1.0001191	68.7093150	68.7929000	66.7856969	100002.0	0.0421317	0.95	43.6843000	94.1205000
age.s[14]	1.0000357	1.0000953	38.3722136	34.2204500	28.9867533	60640.8	0.0608764	0.95	16.8601000	72.1103000
age.s[15]	1.0000215	1.0001302	52.9845036	51.0328000	46.6858052	79174.7	0.0533178	0.95	27.2420000	84.0913000
age.s[16]	1.0000055	1.0000647	53.0754937	51.1187000	46.0935983	81363.5	0.0525842	0.95	27.4776000	84.3053000

age.s[17]	0.9999728	0.9999793	53.0216877	51.0942000	47.3723964	76213.7	0.0542843	0.95	27.1271000	83.8375000
age.s[18]	0.9999758	0.9999872	53.0095053	51.1041500	46.7041072	79420.8	0.0530681	0.95	27.6193000	84.1282000
age.s[19]	0.9999958	1.0000237	38.3773255	34.2655500	28.6075463	61524.4	0.0605604	0.95	16.5305000	71.7525000
age.s[20]	0.9999858	1.0000176	53.0663993	51.1678500	45.1915800	82614.9	0.0523992	0.95	27.6165000	84.5385000
age.s[21]	1.0000971	1.0002054	27.1508133	22.5766000	16.8702233	55511.7	0.0577028	0.95	15.0001000	58.4246000
age.s[22]	1.0000203	1.0001517	68.7691381	68.8895500	69.1102476	100002.0	0.0419842	0.95	44.2344000	94.6781000
age.s[23]	1.0000433	1.0001930	38.4021454	34.2419000	28.9464430	61291.4	0.0607804	0.95	16.7556000	72.1940000
age.s[24]	1.0000623	1.0001930	27.1344846	22.5597500	16.8992480	57033.4	0.0569816	0.95	15.0004000	58.5735000
age.s[25]	1.0000535	1.0001275	27.1109691	22.5618500	16.8841125	54815.1	0.0576877	0.95	15.0001000	58.0184000
age.s[26]	0.9999908	1.0000441	52.9833764	51.0392000	45.5953757	78407.8	0.0534590	0.95	27.7641000	84.2815000
age.s[27]	0.9999927	1.0000507	27.1374029	22.5880500	17.0460694	54430.4	0.0580298	0.95	15.0002000	58.3313000
age.s[28]	0.9999894	1.0000104	38.3950737	34.2434500	28.8177347	59954.9	0.0613967	0.95	16.7682000	72.1948000
age.s[29]	0.9999955	1.0000337	53.0080352	51.1045500	46.6876199	80791.1	0.0527889	0.95	27.8287000	84.5887000
age.s[30]	1.0002472	1.0004021	27.1698313	22.6014000	17.0757364	55035.2	0.0579751	0.95	15.0000000	58.6721000
age.s[31]	1.0000484	1.0002222	68.7676766	68.9016000	68.1004029	100002.0	0.0421364	0.95	44.1985000	94.5439000
age.s[32]	1.0001385	1.0004388	38.3425670	34.2385500	29.1693530	61643.5	0.0603602	0.95	16.5414000	71.9046000
age.s[33]	1.0000763	1.0003406	68.7300442	68.8481500	70.0653734	99534.3	0.0423144	0.95	44.0517000	94.7452000
age.s[34]	1.0000372	1.0000946	68.8046013	68.9492000	69.6971779	100821.6	0.0420602	0.95	44.3076000	94.7596000
age.s[35]	0.9999956	1.0000388	52.9930475	51.0742500	45.8041570	78894.4	0.0533520	0.95	27.7335000	84.5958000
age.s[36]	1.0000039	1.0000210	53.0043140	51.0231500	45.4242603	81548.1	0.0525246	0.95	27.7519000	84.6051000
age.s[37]	1.0000073	1.0000535	53.0403780	51.1581000	45.9312649	79778.4	0.0529927	0.95	27.4494000	84.1149000
age.s[38]	1.0000726	1.0002307	27.1541415	22.6178000	17.0630355	54304.5	0.0580796	0.95	15.0006000	58.2889000
age.s[39]	1.0000147	1.0001118	68.7524180	68.8681000	68.3350640	100002.0	0.0421640	0.95	44.3183000	94.8434000
age.s[40]	1.0000874	1.0002853	27.1633143	22.6448000	17.0663821	53762.9	0.0583874	0.95	15.0003000	58.3058000
age.s[41]	0.9999822	0.9999839	53.0246138	51.1312000	44.7456965	79618.4	0.0531867	0.95	26.7281000	83.5195000
age.s[42]	1.0000414	1.0001840	82.2787764	83.6897000	85.4139101	81156.0	0.0383761	0.95	62.3768000	99.9991000
age.s[43]	0.9999827	1.0000127	68.7429000	68.8619500	68.5854374	97593.9	0.0426217	0.95	44.2673000	94.7616000
age.s[44]	1.0000107	1.0001064	52.9769035	51.0387500	46.1657845	81118.1	0.0526036	0.95	27.9103000	84.5552000
age.s[45]	0.9999887	1.0000156	68.7564611	68.8178000	66.4999417	99662.3	0.0422952	0.95	44.2356000	94.7828000
age.s[46]	1.0000425	1.0001604	38.3292766	34.2008500	29.3498878	61142.5	0.0606264	0.95	16.3727000	71.6432000
age.s[47]	1.0000229	1.0001251	68.7633777	68.8616500	67.9243400	100002.0	0.0422396	0.95	43.7268000	94.4164000
age.s[48]	1.0000755	1.0003287	52.9839931	51.0182000	46.4714692	80495.0	0.0528092	0.95	27.3927000	83.9297000
age.s[49]	1.0000493	1.0000808	68.7984889	68.9176000	68.7881372	100002.0	0.0421733	0.95	44.2410000	94.6419000
age.s[50]	1.0000264	1.0001677	68.7625919	68.8588500	68.7233503	100002.0	0.0422515	0.95	44.0186000	94.6476000

1.1.5 Allmales n = 25

Table 5: MCMC basic diagnostics Allmales sample size n = 25.

	PSRF Point est.	PSRF Upper C.I.	Mean	Median	Mode	ESS	MCSE	HDImass	HDIlow	HDIhigh
b	1.0001424	1.0002856	0.0354214	0.0307933	0.0224247	47543.9	0.0000696	0.95	0.0200009	0.0690449
a	0.9999913	1.0000340	0.0125841	0.0125132	0.0121433	59119.1	0.0000298	0.95	0.0001233	0.0241684
M	0.9999925	1.0000374	43.2433145	44.2439500	54.2148161	59238.7	0.0862522	0.95	8.1376700	81.9917000
beta0	1.0007039	1.0024537	-18.9134060	-18.8697000	-19.2269909	12834.4	0.0643096	0.95	-33.5127000	-4.4395400
beta	1.0007000	1.0024542	5.9234619	5.8940250	5.7544689	12264.8	0.0202946	0.95	1.3240300	10.2736000
thresh[1,1]	NaN	NaN	0.5000000	0.5000000	0.4997358	0.0	NaN	0.95	0.5000000	0.5000000
thresh[1,2]	1.0001919	1.0007362	2.7616508	2.5717000	2.2123318	20409.7	0.0082630	0.95	0.9009190	5.0933200
thresh[1,3]	1.0001988	1.0007781	2.7593891	2.5682500	2.0285177	20393.6	0.0082631	0.95	0.8744700	5.0572800
thresh[1,4]	1.0003402	1.0013196	4.2863195	4.0902200	3.9050835	15939.4	0.0134483	0.95	1.3699400	7.5224500
thresh[1,5]	1.0005132	1.0018757	6.8573571	6.6155950	6.2403221	14676.4	0.0209554	0.95	2.4523300	11.6697000
thresh_age_log[1,1]	1.0001406	1.0001711	3.2829714	3.2484250	3.1799863	80914.3	0.0009905	0.95	2.8478600	3.8986900
thresh_age_log[1,2]	1.0000581	1.0002382	3.6774162	3.6575850	3.5706916	77894.1	0.0008792	0.95	3.2429000	4.1795200
thresh_age_log[1,3]	1.0000445	1.0001965	3.6769980	3.6562550	3.6317978	77248.5	0.0008833	0.95	3.2489600	4.1876500
thresh_age_log[1,4]	1.0002156	1.0005583	3.9441339	3.9276800	3.8956019	54928.1	0.0009652	0.95	3.5417900	4.3823200
thresh_age_log[1,5]	1.0024833	1.0029422	4.4088400	4.3688550	4.3669627	29459.5	0.0017800	0.95	3.9676500	4.8366900
thresh_age[1,1]	1.0000216	1.0000657	27.7465666	25.7497000	22.6273612	77549.4	0.0296072	0.95	16.1914000	47.4396000
thresh_age[1,2]	1.0000757	1.0002790	40.7801212	38.7676000	34.6005382	72472.0	0.0390302	0.95	24.2152000	63.1201000
thresh_age[1,3]	1.0000563	1.0002355	40.7638044	38.7161000	34.1621153	73087.6	0.0388460	0.95	24.3262000	63.2974000
thresh_age[1,4]	1.0068638	1.0073088	53.0776689	50.7889000	48.0014689	55377.0	0.0643198	0.95	32.4901000	77.0823000
thresh_age[1,5]	1.0990584	1.0992728	94.3896480	78.9531500	31.0180681	98767.6	2.0833136	0.95	50.6567000	122.5640000
age.s[1]	1.0001640	1.0005938	37.5180325	33.8657000	29.2290514	44845.4	0.0682719	0.95	16.4650000	69.4799000
age.s[2]	1.0000408	1.0001361	61.8499795	61.2739500	58.7565085	90401.7	0.0469810	0.95	36.2766000	90.0255000
age.s[3]	1.0000132	1.0000250	61.8239776	61.2294500	60.3753013	88086.2	0.0476627	0.95	35.5724000	89.3807000
age.s[4]	1.0001378	1.0003697	25.6506350	21.6915500	16.7890526	39103.8	0.0619247	0.95	15.0002000	52.2176000
age.s[5]	1.0008787	1.0016225	25.7078417	21.6757000	16.6178667	38949.2	0.0623532	0.95	15.0011000	52.6199000
age.s[6]	1.0001183	1.0004344	49.0655831	46.7216500	41.2112142	64245.7	0.0569711	0.95	24.8371000	79.6996000
age.s[7]	1.0000675	1.0002288	61.8094665	61.2309000	59.9819632	95559.5	0.0456825	0.95	35.5861000	89.4101000
age.s[8]	1.0000669	1.0002175	37.4994678	33.8196500	28.6865714	47948.1	0.0662762	0.95	16.2878000	69.5856000
age.s[9]	1.0000834	1.0002594	49.0487618	46.6321500	41.0246289	62665.5	0.0578987	0.95	25.3581000	80.6053000
age.s[10]	1.0001195	1.0002177	49.0443588	46.6647500	41.9719419	61761.0	0.0582052	0.95	24.8354000	79.7236000
age.s[11]	1.0004044	1.0007976	25.6945848	21.6677500	16.5753993	38573.1	0.0629353	0.95	15.0001000	52.9766000
age.s[12]	1.0000407	1.0001381	61.8743289	61.1937000	57.7840010	90145.7	0.0470334	0.95	35.6906000	89.5337000
age.s[13]	1.0001412	1.0003965	25.6850748	21.6691500	16.6197417	39657.9	0.0617784	0.95	15.0001000	52.3263000
age.s[14]	1.0000467	1.0002175	77.5996507	78.5603000	80.2358769	86851.1	0.0410016	0.95	56.1918000	99.8253000
age.s[15]	1.0000713	1.0002171	61.8342005	61.2359000	58.9201517	85894.9	0.0480838	0.95	36.3394000	89.9495000
age.s[16]	1.0001009	1.0003270	37.4924324	33.7299500	29.3993505	46948.9	0.0668768	0.95	16.1390000	69.3684000

age.s[17]	0.9999984	1.0000141	61.8648245	61.2488500	60.8614410	86394.5	0.0480739	0.95	36.7345000	90.5751000
age.s[18]	1.0002284	1.0005292	49.0395626	46.5789500	42.1837977	60938.8	0.0587580	0.95	24.5076000	79.6718000
age.s[19]	1.0001731	1.0004848	37.4885776	33.8186000	29.0241520	47461.9	0.0663577	0.95	16.1877000	69.1543000
age.s[20]	1.0002476	1.0005200	25.6946413	21.6846000	16.7894722	40567.9	0.0612626	0.95	15.0001000	52.6722000
age.s[21]	0.9999743	0.9999832	77.5693419	78.5165500	80.0439792	92692.9	0.0395406	0.95	56.5017000	99.9331000
age.s[22]	1.0001944	1.0007382	77.5706892	78.5592500	80.5220351	88808.7	0.0404262	0.95	56.4203000	99.9508000
age.s[23]	1.0000640	1.0002992	61.8340128	61.2420000	57.9540815	90091.9	0.0471008	0.95	35.8399000	89.6564000
age.s[24]	1.0003348	1.0007931	37.5157940	33.8167000	28.5256494	45689.1	0.0676459	0.95	16.3784000	69.4609000
age.s[25]	0.9999870	1.0000041	61.8327865	61.2169500	59.6639859	91983.9	0.0465376	0.95	36.1173000	89.8600000

1.1.6 Allmales n = 10

Table 6: MCMC basic diagnostics Allmales sample size n = 10.

	PSRF Point est.	PSRF Upper C.I.	Mean	Median	Mode	ESS	MCSE	HDImass	HDIlow	HDIhigh
b	1.0000011	1.0000165	0.0431675	0.0391581	0.0274939	404088.2	0.0000277	0.95	0.0200000	0.0793474
a	1.0000038	1.0000259	0.0088501	0.0071582	0.0008657	403835.4	0.0000110	0.95	0.0001232	0.0225336
M	1.0000043	1.0000265	53.8937938	58.3972000	71.4944914	403856.8	0.0315573	0.95	13.8755000	81.9931000
beta0	0.9999972	1.0000007	-18.0235563	-17.8675000	-17.3864733	400002.0	0.0109568	0.95	-31.6634000	-4.2936700
beta	0.9999999	1.0000052	6.1447997	6.0903550	6.0678008	400002.0	0.0035066	0.95	1.7990400	10.5422000
thresh_age_log[1,1]	1.0000037	1.0000249	2.9993211	2.9781650	2.8938932	400002.0	0.0005165	0.95	2.4383600	3.6951500
thresh_age_log[1,2]	1.0000138	1.0000310	3.4193716	3.4120400	3.3680909	400002.0	0.0005072	0.95	2.8450000	4.0436700
thresh_age_log[1,3]	1.0000105	1.0000373	3.4207009	3.4134450	3.3534376	400002.0	0.0005075	0.95	2.8428900	4.0434400
thresh_age_log[1,4]	1.0000042	1.0000275	3.9519062	3.9677300	4.0277732	400002.0	0.0004083	0.95	3.4410600	4.4085800
thresh_age_log[1,5]	1.0000403	1.0000738	4.3360560	4.3354450	4.3670572	400002.0	0.0004004	0.95	3.8464000	4.7559900
thresh_age[1,1]	1.0000068	1.0000347	21.1503796	19.6517000	17.3955214	400002.0	0.0111795	0.95	9.8767500	37.4284000
thresh_age[1,2]	1.0000039	1.0000252	32.1478493	30.3271500	25.9352681	400002.0	0.0164778	0.95	15.4816000	53.8286000
thresh_age[1,3]	0.9999999	1.0000158	32.1915107	30.3696500	25.6427004	400002.0	0.0164899	0.95	15.4615000	54.0093000
thresh_age[1,4]	1.0021486	1.0021821	53.7959436	52.8642500	48.9154159	400002.0	0.0234829	0.95	29.2238000	78.8864000
thresh_age[1,5]	1.2496676	1.2501807	80.4467394	76.3589500	23.6032364	400002.0	0.3293889	0.95	43.9062000	111.6630000
age.s[1]	1.0000065	1.0000135	21.5894454	18.5557000	15.7112461	400002.0	0.0144488	0.95	15.0000000	38.4868000
age.s[2]	1.0000029	1.0000302	77.3480921	78.6562000	81.4889932	425526.2	0.0190576	0.95	54.8753000	99.9995000
age.s[3]	1.0000043	1.0000112	62.8318340	62.9296500	63.4934999	402183.1	0.0234874	0.95	35.0111000	91.0795000
age.s[4]	1.0000035	1.0000298	47.6388675	45.5720500	40.9298814	400002.0	0.0251705	0.95	20.5093000	79.3514000
age.s[5]	1.0000154	1.0000255	29.8864563	26.1629000	20.7114298	400002.0	0.0205364	0.95	15.0000000	57.0414000
age.s[6]	0.9999952	1.0000001	47.6369679	45.5881500	37.3414131	400002.0	0.0251904	0.95	20.3184000	79.2324000
age.s[7]	1.0000059	1.0000270	47.6578456	45.6370500	39.1400284	406893.8	0.0249567	0.95	20.3869000	79.2927000
age.s[8]	1.0000058	1.0000325	77.3418351	78.6759500	80.8100757	400002.0	0.0196851	0.95	54.7387000	99.9998000
age.s[9]	0.9999989	1.0000006	62.8342111	62.9364000	64.0124831	400002.0	0.0235305	0.95	34.8563000	90.8707000
age.s[10]	0.9999965	0.9999967	62.8665202	62.9712000	63.6578131	400002.0	0.0235450	0.95	34.9566000	90.9480000

1.1.7 Allmales n = 200 with lower mortality (Gompertz $\beta = 0.05$)

Table 7: MCMC basic diagnostics Allmales lower mortality, sample size n = 200.

	PSRF Point est.	PSRF Upper C.I.	Mean	Median	Mode	ESS	MCSE	HDImass	HDIlow	HDIhigh
b	1.0005776	1.0011136	0.0367300	0.0318363	0.0229989	27745.2	0.0000969	0.95	0.0200002	0.0737861
a	1.0001001	1.0003704	0.0119618	0.0116712	0.0080635	36376.1	0.0000381	0.95	0.0001233	0.0239631
M	1.0000949	1.0003323	45.0501890	46.5200000	55.8863714	36281.9	0.1102408	0.95	8.8714400	81.9857000
beta0	1.0015464	1.0055563	-19.2603636	-19.1816000	-19.1014570	12668.1	0.0623469	0.95	-33.5055000	-5.7829400
beta	1.0015998	1.0057281	6.3979441	6.3517200	6.2118279	11535.7	0.0212132	0.95	1.9407400	10.9381000
thresh[1,1]	NaN	NaN	0.5000000	0.5000000	0.4996965	0.0	NaN	0.95	0.5000000	0.5000000
thresh[1,2]	1.0011539	1.0042945	1.9173857	1.8310600	1.6489742	15760.6	0.0049318	0.95	0.8500140	3.1158900
thresh[1,3]	1.0014920	1.0054799	2.6064382	2.5156300	2.4621387	13781.3	0.0072696	0.95	1.0417100	4.2125200
thresh[1,4]	1.0013883	1.0050473	5.4000626	5.3233200	5.5036032	11233.2	0.0171221	0.95	1.9138600	8.7019100
thresh[1,5]	1.0013049	1.0047625	7.8584266	7.7465900	7.8917714	11141.3	0.0251053	0.95	2.7960000	12.5659000
thresh_age_log[1,1]	1.0004881	1.0006211	3.0889223	3.0240300	2.9391722	42792.2	0.0012751	0.95	2.7675600	3.7450000
thresh_age_log[1,2]	1.0002006	1.0002090	3.3160643	3.2558800	3.1264838	40886.6	0.0012904	0.95	2.9604900	3.9083300
thresh_age_log[1,3]	1.0001023	1.0001059	3.4254580	3.3720300	3.2293318	40416.2	0.0012639	0.95	3.0574700	3.9756700
thresh_age_log[1,4]	1.0001415	1.0003794	3.8685259	3.8438500	3.7390321	35616.5	0.0010656	0.95	3.5450700	4.2725300
thresh_age_log[1,5]	1.0020791	1.0041306	4.2664816	4.2436900	4.2016639	23040.0	0.0011792	0.95	3.9906500	4.5598800
thresh_age[1,1]	1.0001927	1.0003043	22.7681771	20.5740000	18.7048134	38224.4	0.0342777	0.95	14.7345000	40.3384000
thresh_age[1,2]	1.0001209	1.0001538	28.5590981	25.9425000	22.5428206	37826.1	0.0422546	0.95	18.6220000	48.7301000
thresh_age[1,3]	1.0000788	1.0000944	31.7964356	29.1375000	24.9775176	37272.0	0.0455785	0.95	20.5889000	52.3354000
thresh_age[1,4]	1.0001981	1.0004700	48.8737246	46.7048000	40.0247126	33810.0	0.0556655	0.95	33.9002000	70.5655000
thresh_age[1,5]	1.0047339	1.0064988	72.6516896	69.6643000	65.4642926	25539.3	0.1177207	0.95	53.5430000	94.8060000
age.s[1]	1.0003966	1.0008513	43.1842793	40.2011000	34.7030714	31392.9	0.0819858	0.95	20.6865000	74.7265000
age.s[2]	1.0007166	1.0011575	29.1255047	25.1525000	21.7889742	24472.7	0.0805638	0.95	15.0016000	56.8458000
age.s[3]	1.0002185	1.0002851	58.8373495	57.7250000	55.2219227	40559.2	0.0693274	0.95	34.2740000	87.1697000
age.s[4]	1.0000598	1.0002042	58.8208946	57.8463000	53.7915621	42366.2	0.0672617	0.95	34.3061000	86.8599000
age.s[5]	1.0003358	1.0007733	43.2241893	40.1067000	34.6115725	31912.6	0.0818267	0.95	20.3103000	74.8020000
age.s[6]	1.0000622	1.0000836	58.8488087	57.7571000	54.8580735	41110.6	0.0686574	0.95	33.4324000	86.2486000
age.s[7]	1.0000432	1.0000671	58.8182983	57.7311000	53.5934180	40862.9	0.0687902	0.95	34.4420000	87.1486000
age.s[8]	0.9999843	1.0000884	75.1150333	75.6264000	75.7285544	50964.2	0.0544805	0.95	53.3491000	98.8844000
age.s[9]	1.0000772	1.0002449	58.7566029	57.6607000	53.3671485	41353.6	0.0682240	0.95	34.2728000	87.2019000
age.s[10]	1.0010502	1.0021390	29.0783906	25.1245000	21.8643284	25526.0	0.0783377	0.95	15.0011000	56.9792000
age.s[11]	1.0001846	1.0004344	58.7611730	57.7628000	51.5278749	42142.7	0.0677872	0.95	33.7201000	86.4572000
age.s[12]	1.0003290	1.0010297	43.2687603	40.1712000	35.6085067	30878.4	0.0830386	0.95	20.3664000	74.7111000
age.s[13]	0.9999771	0.9999949	58.8827672	57.8719000	54.6625721	41822.5	0.0679632	0.95	34.4274000	87.2505000
age.s[14]	1.0002209	1.0005692	58.8789098	57.8173000	53.6457826	41209.7	0.0685019	0.95	33.9140000	86.5207000
age.s[15]	1.0014983	1.0028539	22.3167778	19.1036000	16.0638195	24258.1	0.0644780	0.95	15.0000000	42.2241000

age.s[16]	1.0005708	1.0016107	29.1447547	25.1520000	21.5474192	24963.7	0.0800279	0.95	15.0027000	57.4823000
age.s[17]	1.0000823	1.0001800	58.8869407	57.7873000	54.5617867	41918.9	0.0677900	0.95	34.2918000	86.7989000
age.s[18]	1.0005015	1.0017406	58.8046360	57.7467000	55.2982329	42622.4	0.0671046	0.95	33.2764000	85.7945000
age.s[19]	1.0002320	1.0007388	43.2816526	40.1699000	34.6649585	31558.8	0.0820834	0.95	20.4363000	74.7456000
age.s[20]	1.0001602	1.0005298	75.0393996	75.5289000	75.3049617	50001.0	0.0546631	0.95	53.6203000	99.0263000
age.s[21]	1.0000817	1.0001551	75.1520920	75.6862000	77.1652457	50001.0	0.0546891	0.95	53.3865000	98.4573000
age.s[22]	1.0002167	1.0005781	43.2787409	40.1786000	35.0101022	32822.4	0.0805404	0.95	20.9708000	75.4801000
age.s[23]	1.0012371	1.0022453	33.5271054	29.5716000	25.2056254	26043.3	0.0826636	0.95	15.3322000	62.8314000
age.s[24]	1.0004045	1.0012811	43.3237058	40.2724000	34.4893173	31400.8	0.0829062	0.95	20.5002000	75.4727000
age.s[25]	1.0003830	1.0007099	43.2559111	40.1664000	34.1361359	31681.9	0.0820715	0.95	20.1710000	74.5394000
age.s[26]	1.0002696	1.0007511	43.2300874	40.2540000	34.8303352	31883.4	0.0813604	0.95	21.0087000	75.2025000
age.s[27]	1.0007461	1.0019313	33.5441205	29.5499000	25.4148311	25721.3	0.0834893	0.95	15.4768000	63.3981000
age.s[28]	1.0000849	1.0003632	58.7910063	57.6897000	55.1705692	41438.1	0.0680555	0.95	33.8459000	86.4383000
age.s[29]	1.0001674	1.0003340	75.1336373	75.6576000	75.3634819	50001.0	0.0545750	0.95	53.5586000	98.7074000
age.s[30]	1.0001723	1.0003214	58.7606110	57.7001000	54.0510914	40210.4	0.0690855	0.95	34.5157000	87.1020000
age.s[31]	1.0007624	1.0014366	22.2744424	19.0599000	16.0739781	25082.5	0.0632398	0.95	15.0004000	42.1312000
age.s[32]	1.0010146	1.0022268	33.6019398	29.6376000	25.0412498	25056.5	0.0849623	0.95	15.3540000	63.4843000
age.s[33]	1.0001868	1.0004578	58.7620648	57.7473000	54.2764629	41860.0	0.0676388	0.95	33.5953000	86.2788000
age.s[34]	1.0001103	1.0001927	58.8552426	57.8042000	55.1059256	40851.4	0.0687568	0.95	33.8819000	86.1566000
age.s[35]	1.0010297	1.0022171	29.1318696	25.1454000	21.6593085	23876.7	0.0813423	0.95	15.0077000	56.9381000
age.s[36]	1.0005048	1.0012059	43.3031847	40.1982000	34.1449369	31266.8	0.0828910	0.95	20.2467000	74.8166000
age.s[37]	1.0000676	1.0001246	58.7912067	57.6873000	55.6481617	43971.0	0.0658388	0.95	34.4920000	86.8236000
age.s[38]	1.0002517	1.0007299	43.2483586	40.2049000	34.2419466	33052.8	0.0804380	0.95	20.2847000	74.6040000
age.s[39]	1.0015702	1.0028879	22.3037567	19.0351000	16.0589540	23715.8	0.0655334	0.95	15.0001000	42.5769000
age.s[40]	1.0000125	1.0001700	75.0116321	75.5785000	75.8208004	48636.9	0.0557745	0.95	52.6869000	98.1496000
age.s[41]	1.0000686	1.0002901	58.7295303	57.6292000	52.3021545	41383.8	0.0683345	0.95	34.2493000	86.9554000
age.s[42]	1.0001596	1.0003040	75.1229017	75.6887000	76.5209753	50001.0	0.0548118	0.95	52.9391000	98.2777000
age.s[43]	1.0002276	1.0004495	43.3043434	40.1638000	34.8406443	31559.6	0.0823535	0.95	20.6228000	75.1011000
age.s[44]	1.0003649	1.0011205	43.2327957	40.2146000	34.1273203	32639.2	0.0806425	0.95	20.4514000	74.8263000
age.s[45]	1.0000548	1.0002326	58.7967255	57.7329000	53.4062869	43097.2	0.0669978	0.95	33.8833000	86.6147000
age.s[46]	1.0012546	1.0021658	22.3456579	19.0843000	16.0968646	24692.8	0.0647232	0.95	15.0002000	42.5084000
age.s[47]	1.0001264	1.0005831	58.7883747	57.7945000	54.1041000	40785.3	0.0688936	0.95	33.7835000	86.5022000
age.s[48]	1.0000252	1.0000851	75.0247856	75.5530000	76.2508090	50001.0	0.0548028	0.95	53.4308000	98.9925000
age.s[49]	1.0009914	1.0019261	29.1673806	25.2216000	21.5361709	25054.4	0.0798156	0.95	15.0008000	57.1620000
age.s[50]	0.9999935	1.0001017	58.8197824	57.6918000	55.6734245	42486.1	0.0676370	0.95	33.6170000	86.5059000
age.s[51]	1.0002456	1.0006963	43.2401578	40.1239000	35.6592294	31798.0	0.0817314	0.95	20.7350000	74.9773000
age.s[52]	1.0010255	1.0019261	29.1422983	25.1622000	21.4991223	24840.9	0.0802505	0.95	15.0051000	57.2048000
age.s[53]	1.0013807	1.0028485	29.1497219	25.1461000	21.5370416	25771.3	0.0784755	0.95	15.0015000	57.0652000
age.s[54]	1.0005740	1.0014293	43.2907075	40.1813000	34.4488428	31155.1	0.0828468	0.95	20.4589000	74.8414000

age.s[55]	1.0008083	1.0014257	22.2976568	19.0648000	16.2531893	24886.1	0.0637864	0.95	15.0000000	42.4328000
age.s[56]	1.0001845	1.0002319	75.1304859	75.6316000	76.1504787	50001.0	0.0546296	0.95	53.3991000	98.7335000
age.s[57]	1.0006888	1.0019844	43.3343918	40.3297000	34.3261544	31828.1	0.0816589	0.95	20.4066000	74.6488000
age.s[58]	1.0001836	1.0006420	43.2365875	40.1410000	35.3343009	32306.9	0.0813331	0.95	20.5319000	74.8117000
age.s[59]	1.0002156	1.0005617	43.2822811	40.1382000	34.1545975	31987.9	0.0816635	0.95	20.5814000	75.0061000
age.s[60]	1.0010153	1.0023073	29.1411932	25.1699000	21.7455502	24399.8	0.0808086	0.95	15.0003000	57.4295000
age.s[61]	0.9999747	1.0000588	75.1789527	75.7331000	75.8822903	50947.8	0.0540719	0.95	53.3913000	98.6055000
age.s[62]	1.0000608	1.0003820	75.1243714	75.6676000	77.4322410	50001.0	0.0546157	0.95	52.8727000	98.2752000
age.s[63]	1.0000371	1.0000910	58.7862325	57.6923000	55.0783173	41891.6	0.0679852	0.95	33.6810000	86.2836000
age.s[64]	1.0001453	1.0002661	58.8860083	57.7978000	55.6305601	42779.6	0.0671175	0.95	34.4677000	87.1157000
age.s[65]	1.0000009	1.0000717	75.0942947	75.5767000	74.7181755	50001.0	0.0548030	0.95	52.8543000	98.4329000
age.s[66]	1.0008923	1.0018876	33.5878768	29.5557000	25.1795535	25317.2	0.0846063	0.95	15.5293000	63.7269000
age.s[67]	1.0004733	1.0011524	33.5850538	29.5612000	25.1832302	24727.9	0.0854085	0.95	15.2048000	63.2417000
age.s[68]	1.0000438	1.0001764	58.8032625	57.7018000	55.1286910	41613.6	0.0681331	0.95	33.8368000	86.5790000
age.s[69]	1.0007660	1.0016431	29.1223009	25.1648000	21.9866181	25325.4	0.0790469	0.95	15.0011000	56.7906000
age.s[70]	1.0007411	1.0023742	43.2814183	40.2457000	35.1135103	32536.5	0.0805412	0.95	19.9596000	74.2179000
age.s[71]	1.0003000	1.0005171	43.2805111	40.1950000	34.8314606	31515.3	0.0824010	0.95	20.8239000	75.5096000
age.s[72]	1.0030050	1.0053189	22.3058370	19.0619000	16.1086186	23689.8	0.0657634	0.95	15.0004000	42.4973000
age.s[73]	1.0007486	1.0015172	29.1444747	25.2088000	21.8215266	25160.7	0.0792658	0.95	15.0021000	57.0114000
age.s[74]	1.0001827	1.0003020	43.3620388	40.2845000	33.7252611	31674.9	0.0824803	0.95	20.7430000	75.2174000
age.s[75]	1.0000171	1.0000474	58.8629520	57.9062000	55.6187108	38790.5	0.0701472	0.95	34.5084000	86.9431000
age.s[76]	1.0006318	1.0014161	43.2602528	40.2096000	34.1241823	29748.6	0.0844556	0.95	20.2796000	74.2893000
age.s[77]	1.0000330	1.0001520	58.8076901	57.7761000	55.8020594	43618.8	0.0663647	0.95	34.4930000	87.0207000
age.s[78]	1.0002980	1.0007202	43.2418764	40.1869000	34.1342446	31845.5	0.0817542	0.95	20.3170000	74.8295000
age.s[79]	1.0000490	1.0001780	58.8060879	57.7648000	56.4675633	40996.1	0.0683859	0.95	34.0115000	86.5128000
age.s[80]	1.0003437	1.0007794	43.2314321	40.1859000	34.9840841	32287.6	0.0808433	0.95	20.3740000	74.4651000
age.s[81]	1.0001915	1.0006255	43.2866198	40.1844000	34.2756851	31734.4	0.0821538	0.95	20.0175000	74.6228000
age.s[82]	1.0001273	1.0003823	43.2278777	40.1085000	35.0689134	31773.1	0.0822809	0.95	20.4192000	74.8410000
age.s[83]	1.0001650	1.0005610	58.7770430	57.6087000	54.1172634	42263.9	0.0676682	0.95	33.8703000	86.5252000
age.s[84]	1.0000689	1.0001465	75.1470566	75.6547000	77.0047452	50001.0	0.0545381	0.95	53.1035000	98.1872000
age.s[85]	1.0000468	1.0000899	58.8251334	57.8564000	52.7354062	41301.9	0.0681141	0.95	34.1156000	86.6395000
age.s[86]	1.0004571	1.0011557	43.2382695	40.1968000	34.0150354	32021.5	0.0813314	0.95	20.1415000	74.2912000
age.s[87]	1.0002303	1.0007884	58.8797576	57.7313000	52.8413859	42511.6	0.0674231	0.95	34.1478000	86.9870000
age.s[88]	1.0001963	1.0004867	43.2654765	40.1625000	34.4564459	33400.5	0.0800488	0.95	20.3561000	75.0521000
age.s[89]	1.0001844	1.0005430	43.2610593	40.2211000	34.8450829	32427.5	0.0812634	0.95	20.2973000	74.7984000
age.s[90]	1.0002678	1.0008659	43.2927637	40.1739000	34.8339642	31100.7	0.0833663	0.95	20.0939000	74.9778000
age.s[91]	0.9999466	0.9999634	58.7267395	57.6512000	54.7569558	40675.7	0.0686496	0.95	33.7227000	86.3510000
age.s[92]	1.0001006	1.0005301	75.0666628	75.6615000	76.5822879	50001.0	0.0548055	0.95	52.8078000	98.2569000
age.s[93]	1.0001733	1.0006409	75.0718064	75.6356000	78.9551793	50001.0	0.0550534	0.95	53.3197000	98.6979000

age.s[94]	1.0001429	1.0004632	58.8364901	57.7915000	54.5976619	41113.3	0.0681703	0.95	34.0146000	86.3339000
age.s[95]	1.0001145	1.0004881	58.7920805	57.8391000	52.8269121	39840.4	0.0695886	0.95	34.2280000	86.6852000
age.s[96]	0.9999905	1.0000672	58.7906171	57.7354000	54.5808624	42237.4	0.0673899	0.95	34.1011000	86.4750000
age.s[97]	1.0019000	1.0034717	22.3257984	19.0591000	16.0910408	24348.0	0.0648608	0.95	15.0000000	42.4760000
age.s[98]	1.0003746	1.0010285	43.3399600	40.1892000	34.5016386	32083.9	0.0815603	0.95	20.3908000	74.7867000
age.s[99]	1.0005951	1.0012124	29.1638909	25.1887000	21.6349611	24560.9	0.0802340	0.95	15.0012000	56.9397000
age.s[100]	1.0001411	1.0004991	43.2472248	40.1256000	35.0268591	30815.3	0.0829010	0.95	20.4289000	74.6761000
age.s[101]	0.9999829	1.0000240	58.8956578	57.8606000	54.9980856	42524.4	0.0673216	0.95	33.7798000	86.4358000
age.s[102]	1.0000319	1.0002161	58.8539737	57.8601000	54.2185328	42555.6	0.0673322	0.95	33.3102000	85.9820000
age.s[103]	1.0000063	1.0000711	75.0037565	75.5796000	76.7008496	50001.0	0.0549207	0.95	53.5065000	99.0390000
age.s[104]	1.0007639	1.0017867	43.3068125	40.1500000	35.1521348	32522.8	0.0812749	0.95	20.5766000	75.0702000
age.s[105]	1.0005741	1.0014322	43.1685767	40.1140000	34.6875005	31191.0	0.0825040	0.95	20.5211000	74.7376000
age.s[106]	1.0011194	1.0023884	33.6191227	29.5482000	25.1619448	25691.6	0.0842963	0.95	15.7868000	64.2184000
age.s[107]	1.0006331	1.0013112	43.2691872	40.1246000	34.3155291	30847.5	0.0830890	0.95	19.9870000	74.2214000
age.s[108]	1.0001963	1.0005792	58.7776639	57.6702000	54.6848710	41682.6	0.0677652	0.95	33.5811000	86.1991000
age.s[109]	1.0003334	1.0004090	75.0174024	75.5744000	77.1112938	50001.0	0.0549900	0.95	53.2842000	98.7205000
age.s[110]	1.0000464	1.0000613	58.8020849	57.6805000	53.2677719	41684.8	0.0681562	0.95	33.4937000	86.2643000
age.s[111]	1.0002198	1.0007782	75.0997849	75.6587000	75.3513415	50001.0	0.0547636	0.95	52.8747000	98.3464000
age.s[112]	1.0001515	1.0003373	75.0797162	75.6824000	77.6853003	49006.0	0.0556649	0.95	53.3248000	98.7995000
age.s[113]	1.0000559	1.0002635	75.0451663	75.5461000	77.6460121	50001.0	0.0545418	0.95	52.9041000	98.2228000
age.s[114]	1.0001294	1.0001642	75.0290250	75.6144000	76.1127499	50001.0	0.0547878	0.95	53.3615000	98.8531000
age.s[115]	1.0001348	1.0003606	58.8193756	57.6754000	54.2990157	40937.0	0.0688421	0.95	34.3051000	87.1804000
age.s[116]	1.0010987	1.0024701	33.6109417	29.6018000	25.2025237	26614.6	0.0822685	0.95	15.5437000	63.5217000
age.s[117]	1.0004182	1.0011632	43.2285743	40.1415000	33.8447942	32829.9	0.0805881	0.95	20.4575000	74.7835000
age.s[118]	1.0026617	1.0045440	22.3164652	19.0670000	16.1421336	24811.8	0.0639533	0.95	15.0002000	42.1996000
age.s[119]	1.0002184	1.0006317	58.8377731	57.7765000	55.5898303	42548.2	0.0674448	0.95	34.1489000	86.7887000
age.s[120]	1.0001127	1.0004053	58.8016583	57.7968000	56.3614616	42463.1	0.0673495	0.95	33.4753000	86.1861000
age.s[121]	1.0001684	1.0003827	75.1474119	75.7043000	78.1238893	50001.0	0.0547880	0.95	53.0757000	98.4086000
age.s[122]	1.0001217	1.0003525	43.2646899	40.2714000	34.3258159	32352.3	0.0808128	0.95	20.9160000	75.0219000
age.s[123]	1.0000545	1.0001595	58.8245162	57.7971000	55.7492630	42171.8	0.0674428	0.95	34.6448000	87.0761000
age.s[124]	1.0000152	1.0000726	58.7544695	57.7287000	54.4789098	43523.2	0.0667066	0.95	33.8210000	86.4517000
age.s[125]	1.0000794	1.0001175	58.8690406	57.7238000	54.8187622	41374.6	0.0683847	0.95	34.1946000	87.0243000
age.s[126]	1.0003042	1.0007700	58.9058393	57.8607000	52.6844338	38990.6	0.0702637	0.95	34.1563000	86.6145000
age.s[127]	1.0000063	1.0001749	58.7253245	57.6891000	53.9564988	42169.4	0.0674088	0.95	34.4028000	86.8451000
age.s[128]	1.0003333	1.0010790	58.8510206	57.7592000	52.2817218	40665.5	0.0687979	0.95	34.2333000	86.6150000
age.s[129]	1.0001953	1.0005208	58.8036361	57.7176000	55.0728196	41575.1	0.0680709	0.95	34.4680000	87.0064000
age.s[130]	1.0004420	1.0008385	43.3116037	40.1950000	35.5395550	30900.8	0.0832284	0.95	20.0750000	74.6815000
age.s[131]	1.0000461	1.0001944	75.0946342	75.6366000	76.6949954	49041.6	0.0553834	0.95	53.1650000	98.6594000
age.s[132]	1.0004325	1.0010262	43.2898203	40.2736000	35.2867381	32952.6	0.0802838	0.95	20.2925000	74.7073000

age.s[133]	1.0004489	1.0011803	43.2616243	40.1521000	34.7873236	31867.3	0.0818371	0.95	20.4112000	74.7211000
age.s[134]	1.0000884	1.0004125	58.8118523	57.7903000	53.3522574	41531.2	0.0680555	0.95	33.7745000	86.5018000
age.s[135]	1.0018729	1.0033823	22.2707749	19.0269000	16.0848586	24498.5	0.0641921	0.95	15.0001000	42.2070000
age.s[136]	1.0001614	1.0006236	58.7191950	57.6503000	55.8300354	38166.1	0.0711440	0.95	34.1807000	86.8884000
age.s[137]	1.0000017	1.0000783	58.8280147	57.7688000	53.4079577	40512.6	0.0688478	0.95	34.0762000	86.3734000
age.s[138]	1.0002357	1.0006897	43.2615664	40.1897000	34.7667453	31094.9	0.0825210	0.95	20.2532000	74.6427000
age.s[139]	0.9999671	0.9999736	75.0412430	75.6035000	76.9711980	50001.0	0.0547635	0.95	52.9981000	98.4138000
age.s[140]	1.0000338	1.0000933	75.1202896	75.6606000	75.9168373	50001.0	0.0549511	0.95	52.7476000	98.1694000
age.s[141]	1.0000690	1.0001011	74.9608694	75.4013000	75.1693708	50360.1	0.0545307	0.95	52.8561000	98.3350000
age.s[142]	1.0004709	1.0012828	43.2737585	40.2138000	34.2540322	32132.5	0.0813472	0.95	20.3319000	74.5401000
age.s[143]	1.0009597	1.0019970	29.1342663	25.1489000	21.3225273	25118.7	0.0796869	0.95	15.0215000	56.9234000
age.s[144]	1.0000819	1.0004151	58.8621847	57.7520000	55.0902964	41714.6	0.0681289	0.95	34.2559000	87.0960000
age.s[145]	1.0014844	1.0030114	29.1029688	25.1157000	21.6185549	25326.4	0.0793053	0.95	15.0061000	57.1340000
age.s[146]	1.0003395	1.0011414	75.0720967	75.5686000	78.1411364	49161.1	0.0553223	0.95	53.2402000	98.7010000
age.s[147]	0.9999811	1.0000100	75.0990140	75.6063000	75.2452966	50001.0	0.0548303	0.95	52.9191000	98.3660000
age.s[148]	1.0000811	1.0001423	58.8037119	57.7935000	53.3225018	43448.1	0.0668742	0.95	34.0531000	87.1281000
age.s[149]	1.0024808	1.0036877	22.3671427	19.0880000	16.0785199	24006.1	0.0652373	0.95	15.0001000	42.5763000
age.s[150]	1.0000434	1.0001917	75.0402267	75.5060000	74.3746185	50001.0	0.0546359	0.95	52.5126000	97.9418000
age.s[151]	1.0005026	1.0015525	43.2460111	40.1988000	34.6645556	31534.4	0.0820341	0.95	20.1601000	74.6760000
age.s[152]	1.0008613	1.0018780	33.5559460	29.5822000	25.3054547	26618.5	0.0819353	0.95	15.0678000	62.9957000
age.s[153]	1.0029694	1.0049755	22.3242713	19.0838000	16.0947092	23868.9	0.0648724	0.95	15.0000000	42.4525000
age.s[154]	0.9999917	1.0000633	75.0429407	75.5158000	75.8057554	50001.0	0.0547271	0.95	52.5411000	97.9381000
age.s[155]	1.0007857	1.0016253	22.2877592	19.0951000	16.0705033	24061.5	0.0642028	0.95	15.0002000	41.9299000
age.s[156]	1.0004648	1.0016486	58.7969867	57.7051000	52.8476639	41544.1	0.0685540	0.95	34.3022000	87.1560000
age.s[157]	1.0000572	1.0001076	75.0874598	75.6324000	77.0103083	50001.0	0.0547570	0.95	53.7236000	99.2668000
age.s[158]	1.0003865	1.0011751	75.0463120	75.5540000	78.4621824	50001.0	0.0548001	0.95	53.2548000	98.5646000
age.s[159]	1.0010506	1.0023884	29.1657919	25.1524000	21.9150807	25061.3	0.0799495	0.95	15.0021000	57.1620000
age.s[160]	1.0004074	1.0012342	58.8444866	57.7682000	55.0571279	41132.5	0.0683658	0.95	34.4002000	86.8958000
age.s[161]	1.0007313	1.0014869	22.3227894	19.0761000	16.0855760	24246.2	0.0651831	0.95	15.0001000	42.3087000
age.s[162]	1.0000796	1.0002152	43.3074647	40.2634000	34.7251800	29764.7	0.0842219	0.95	20.7113000	74.5870000
age.s[163]	1.0002686	1.0004119	43.2924096	40.2504000	33.9419284	31003.2	0.0829582	0.95	20.9412000	75.2376000
age.s[164]	1.0000865	1.0001726	58.8425851	57.7677000	54.1705197	42845.5	0.0669938	0.95	34.4273000	86.9780000
age.s[165]	1.0005105	1.0014103	43.2848420	40.2461000	33.5847554	32215.1	0.0813364	0.95	20.8080000	75.2315000
age.s[166]	1.0017169	1.0029079	22.3587894	19.1068000	16.0788408	24305.9	0.0652430	0.95	15.0000000	42.9793000
age.s[167]	1.0004085	1.0011636	58.7066930	57.6404000	54.8534025	43016.6	0.0669202	0.95	34.0860000	86.6591000
age.s[168]	1.0002002	1.0005057	43.2939101	40.2069000	34.5107790	32322.9	0.0812179	0.95	20.1166000	74.5030000
age.s[169]	1.0000575	1.0000794	58.8373359	57.6859000	55.3630839	42245.0	0.0676858	0.95	34.5431000	87.3125000
age.s[170]	1.0002442	1.0007396	58.8062522	57.8361000	53.4545276	41190.2	0.0685316	0.95	34.2303000	86.9530000
age.s[171]	1.0000442	1.0001115	58.8254105	57.7573000	55.7068281	42267.0	0.0674986	0.95	33.9471000	86.5666000

age.s[172]	1.0012865	1.0025708	22.3578554	19.1195000	16.0836705	24341.9	0.0644832	0.95	15.0001000	42.4582000
age.s[173]	1.0005013	1.0011652	43.3287034	40.2082000	35.1637565	31039.9	0.0829174	0.95	20.0349000	74.4680000
age.s[174]	0.9999685	0.9999800	75.0346270	75.5544000	77.5479952	50001.0	0.0546365	0.95	53.6950000	98.8475000
age.s[175]	1.0001894	1.0005568	58.8589060	57.7059000	54.8596720	39147.7	0.0701541	0.95	33.9770000	86.5025000
age.s[176]	1.0003148	1.0009206	75.0588594	75.6518000	77.6546184	47882.3	0.0559040	0.95	53.0930000	98.5995000
age.s[177]	1.0001682	1.0006068	58.8044060	57.7563000	55.0881208	42932.5	0.0669212	0.95	34.1952000	86.8581000
age.s[178]	1.0002925	1.0009357	43.2396020	40.1570000	34.3130309	30344.9	0.0838606	0.95	20.1145000	74.4528000
age.s[179]	1.0003613	1.0009673	43.2724863	40.1201000	33.7505861	31498.8	0.0825329	0.95	20.2058000	74.6871000
age.s[180]	1.0002910	1.0009050	43.2105869	40.1127000	34.6465950	32016.5	0.0813419	0.95	20.4590000	74.9667000
age.s[181]	1.0010109	1.0021585	29.1103064	25.1243000	21.5477866	23745.4	0.0819547	0.95	15.0001000	56.8487000
age.s[182]	1.0001498	1.0005080	58.7780346	57.6743000	55.5577535	41808.9	0.0676901	0.95	34.4662000	86.8834000
age.s[183]	1.0000613	1.0001766	58.7623008	57.7520000	52.9496854	42599.3	0.0670385	0.95	33.8729000	86.3341000
age.s[184]	1.0003976	1.0011233	43.3117836	40.1648000	35.0654100	31613.8	0.0823143	0.95	20.1431000	74.4120000
age.s[185]	1.0002333	1.0007191	75.0797811	75.6431000	75.2621283	49032.0	0.0555166	0.95	52.8123000	98.4053000
age.s[186]	1.0001499	1.0005163	43.2337669	40.1447000	35.2083396	31903.7	0.0814798	0.95	20.7168000	75.0219000
age.s[187]	1.0003081	1.0008505	43.2707561	40.2292000	34.6030511	32338.9	0.0809724	0.95	20.4707000	74.6279000
age.s[188]	1.0001961	1.0005489	58.8176884	57.7948000	55.1904946	41875.1	0.0679788	0.95	33.6952000	86.4442000
age.s[189]	1.0003072	1.0008232	43.2562428	40.1672000	34.1674381	30661.6	0.0833679	0.95	21.0637000	75.5082000
age.s[190]	1.0001216	1.0003092	43.2437579	40.1621000	34.9042036	31452.9	0.0822514	0.95	20.3055000	74.4952000
age.s[191]	0.9999855	1.0000588	75.0988894	75.6160000	76.4433305	50001.0	0.0546292	0.95	53.1778000	98.4639000
age.s[192]	0.9999699	1.0000118	75.1269396	75.7600000	76.9006401	50001.0	0.0546489	0.95	53.1368000	98.5494000
age.s[193]	1.0001764	1.0007263	75.1004675	75.6316000	74.5822047	50001.0	0.0549394	0.95	53.0270000	98.5750000
age.s[194]	1.0005200	1.0014272	43.2403139	40.1997000	35.0716156	32886.1	0.0802062	0.95	19.9395000	74.2064000
age.s[195]	1.0006154	1.0014306	43.1892815	40.0797000	34.5612394	33177.5	0.0799602	0.95	20.1313000	74.3838000
age.s[196]	1.0000971	1.0001755	75.0890592	75.6119000	76.8382583	49034.6	0.0553288	0.95	53.0383000	98.4594000
age.s[197]	1.0005818	1.0014456	33.6082411	29.6248000	25.1452221	26764.9	0.0818999	0.95	15.0771000	63.0253000
age.s[198]	1.0027871	1.0049753	22.3321070	19.0565000	16.0759671	24055.2	0.0652957	0.95	15.0000000	42.5381000
age.s[199]	1.0001965	1.0006131	75.1102190	75.6473000	75.1961831	50001.0	0.0546615	0.95	52.6962000	98.0739000
age.s[200]	1.0001442	1.0005598	58.8334722	57.7791000	55.2373359	42279.2	0.0678557	0.95	33.4157000	86.2482000

1.2 Exact Binomial Tests

Exact binomial tests for ranges between 10% and 95% for the Allmales dataset samples.

1.2.1 Allmales n = 200

Table 8: Exact binomial test for Allmales (n = 200).

coverage	n_in	perc	CI_low	CI_up	p_value
0.10	20	0.100	0.062	0.150	1.000
0.20	36	0.180	0.129	0.240	0.536
0.30	55	0.275	0.214	0.342	0.488
0.40	72	0.360	0.294	0.431	0.279
0.50	97	0.485	0.414	0.557	0.724
0.60	120	0.600	0.529	0.668	1.000
0.70	135	0.675	0.605	0.739	0.441
0.80	150	0.750	0.684	0.808	0.092
0.90	165	0.825	0.765	0.875	0.001
0.95	180	0.900	0.850	0.938	0.003

1.2.2 Allmales n = 100

Table 9: Exact binomial test for Allmales (n = 100).

coverage	n_in	perc	CI_low	CI_up	p_value
0.10	9	0.09	0.042	0.164	0.868
0.20	22	0.22	0.143	0.314	0.617
0.30	29	0.29	0.204	0.389	0.913
0.40	33	0.33	0.239	0.431	0.184
0.50	47	0.47	0.369	0.572	0.617
0.60	52	0.52	0.418	0.621	0.104
0.70	65	0.65	0.548	0.743	0.276
0.80	72	0.72	0.621	0.805	0.059
0.90	80	0.80	0.708	0.873	0.002
0.95	86	0.86	0.776	0.921	0.000

1.2.3 Allmales n = 50

Table 10: Exact binomial test for Allmales (n = 50).

coverage	n_in	perc	CI_low	CI_up	p_value
0.10	1	0.02	0.001	0.106	0.058
0.20	5	0.10	0.033	0.218	0.079
0.30	12	0.24	0.131	0.382	0.441
0.40	19	0.38	0.247	0.528	0.885
0.50	21	0.42	0.282	0.568	0.322
0.60	26	0.52	0.374	0.663	0.252
0.70	29	0.58	0.432	0.718	0.088
0.80	36	0.72	0.575	0.838	0.159
0.90	38	0.76	0.618	0.869	0.003
0.95	45	0.90	0.782	0.967	0.104

1.2.4 Allmales n = 25

Table 11: Exact binomial test for Allmales (n = 25).

coverage	n_in	perc	CI_low	CI_up	p_value
0.10	1	0.04	0.001	0.204	0.508
0.20	2	0.08	0.010	0.260	0.207
0.30	6	0.24	0.094	0.451	0.664
0.40	8	0.32	0.149	0.535	0.541
0.50	12	0.48	0.278	0.687	1.000
0.60	14	0.56	0.349	0.756	0.688
0.70	17	0.68	0.465	0.851	0.829
0.80	20	0.80	0.593	0.932	1.000
0.90	21	0.84	0.639	0.955	0.308
0.95	22	0.88	0.688	0.975	0.127

1.2.5 Allmales n = 10

Table 12: Exact binomial test for Allmales (n = 10).

coverage	n_in	perc	CI_low	CI_up	p_value
0.10	0	0.0	0.000	0.308	0.613
0.20	0	0.0	0.000	0.308	0.228
0.30	0	0.0	0.000	0.308	0.039
0.40	2	0.2	0.025	0.556	0.334
0.50	4	0.4	0.122	0.738	0.754
0.60	5	0.5	0.187	0.813	0.534
0.70	5	0.5	0.187	0.813	0.179
0.80	7	0.7	0.348	0.933	0.430
0.90	9	0.9	0.555	0.997	1.000
0.95	9	0.9	0.555	0.997	0.401

1.2.6 Allmales n = 200 with lower mortality (Gompertz $\beta = 0.05$)

Table 13: Exact binomial test for Allmales with lower mortality (n = 200).

coverage	n_in	perc	CI_low	CI_up	p_value
0.10	21	0.105	0.066	0.156	0.813
0.20	45	0.225	0.169	0.289	0.377
0.30	65	0.325	0.261	0.395	0.441
0.40	82	0.410	0.341	0.482	0.773
0.50	95	0.475	0.404	0.547	0.525
0.60	120	0.600	0.529	0.668	1.000
0.70	140	0.700	0.631	0.763	1.000
0.80	165	0.825	0.765	0.875	0.426
0.90	178	0.890	0.838	0.930	0.637
0.95	186	0.930	0.885	0.961	0.192

1.2.7 Suchey-Brooks method

Exact binomial tests for Suchey-Brooks method with original (Brooks & Suchey, 1990) and Bayesian age ranges (Godde & Hens, 2025). "High mortality" refers to the Allmales data-set (n = 200) and "low mortality" to that with Gompertz $\beta = 0.05$.

Table 14: Exact binomial test for two Allmales data-sets with the Suchey-Brooks method and with original and Bayesian age ranges.

dataset	n_in	perc	CI_low	CI_up	p_value
high mortality, S-B	177	0.885	0.8324531	0.9256866	0.0002255
high mortality, bayes	170	0.850	0.7928413	0.8964505	0.0000001
low mortality, S-B	173	0.865	0.8096908	0.9091129	0.0000030
low mortality, bayes	161	0.805	0.7432143	0.8575081	0.0000000

1.3 Arithmetic mean as point estimate of age-at-death

For comparison, we calculated the Goodness-of-fit for the Allmales samples "n = 200" and "lower mortality" with the arithmetic mean as point estimate of age-at-death instead of the mode.

1.3.1 Allmales n = 200

Table 15: Goodness-of-fit measures for the Allmale data-set (n = 200), with the arithmetic mean as point estimate.

	Gof measures
Bias	8.32
corrPearson	0.76
corr_p	0.00
Residual_age_slope	0.32
Inaccuracy	11.70
RMSE	14.72
TMNLP	4.45
CRPS	7.76

The corresponding image for the calculation based on the mean for comparison with Figure 3 in the main text.

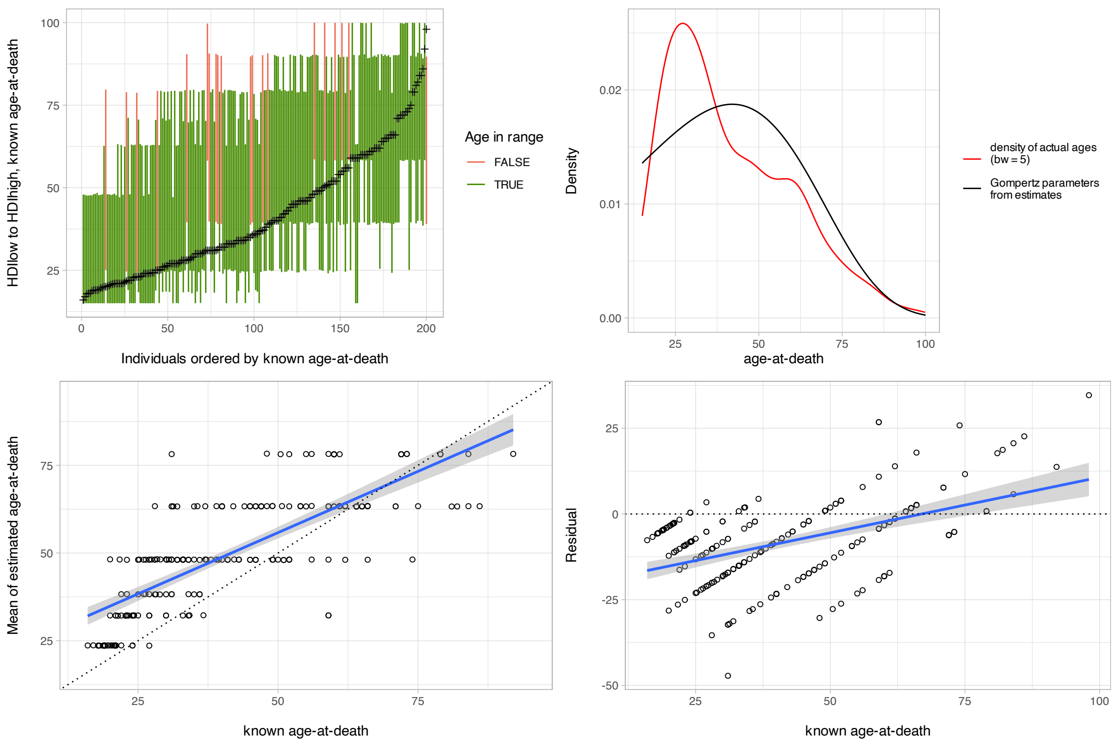

Figure 1: Estimation of age-at-death for data-set 'allmales' (n = 200). Left top – comparison between known age-at-death and the estimated 95%-HDIs, coloured green if the true age lies within the range and red if not; top right – density of known age-at-death and Gompertz function with estimated parameters; bottom left – known age-at-death vs. arithmetic mean of estimated age-at-death, the dotted line marks complete equivalence, regression line in blue; bottom right – known age-at-death vs. residuals (known age-at-death minus arithmetic mean of estimated age-at-death), the dotted line marks complete equivalence, regression line in blue.

1.3.2 Allmales n = 200 with lower mortality (Gompertz $\beta = 0.05$)

Table 16: Goodness-of-fit measures for the Allmale data-set with lower mortality (n = 200), with the arithmetic mean as point estimate.

	Gof measures
Bias	4.25
corrPearson	0.73
corr_p	0.00
Residual_age_slope	0.30
Inaccuracy	9.87
RMSE	12.87
TMNLP	4.41
CRPS	6.97
Coverage	93.00
HDI_Diff_median	52.54

For comparison with Figure 4 in the main text the Allmales dataset with lower mortality and arithmetic mean instead of mode.

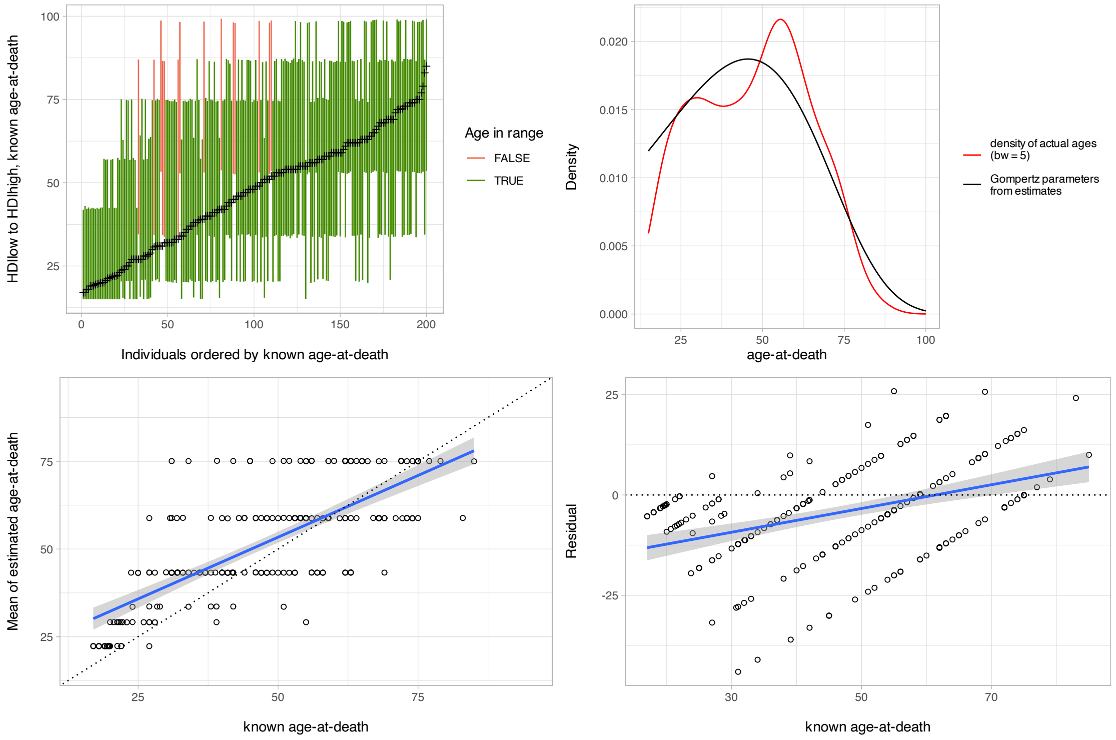

Figure 2: Estimation of age-at-death for data-set 'allmales' (n = 200). Left top – comparison between known age-at-death and the estimated 95%-HDIs, coloured green if the true age lies within the range and red if not; top right – density of known age-at-death and Gompertz function with estimated parameters; bottom left – known age-at-death vs. arithmetic mean of estimated age-at-death, the dotted line marks complete equivalence, regression line in blue; bottom right – known age-at-death vs. residuals (known age-at-death minus arithmetic mean of estimated age-at-death), the dotted line marks complete equivalence, regression line in blue.

2 CAMSAD

Supplementary information for the CAMSAD dataset (Navega, Costa, & Cunha, 2022).

2.1 Diagnostic data

2.1.1 Summarized diagnostic data

Summarized diagnostic data of the MCMC statistics.

Table 17: CAMSAD dataset, diagnostic data.

	5 traits	10 traits	15 traits	25 traits	40 traits	64 traits
PSRF_max	1.001	1.003	1.002	1.001	1.252	1.291
PSRF_upper_max	1.005	1.010	1.005	1.005	1.267	1.301
ESS_min	13210.600	11800.200	10034.200	10972.600	11340.900	11894.500

2.1.2 5 traits

Table 18: MCMC diagnostic data for CAMSAD dataset (n traits = 5).

	PSRF Point est.	PSRF Upper C.I.	Mean	Median	Mode	ESS	MCSE	HDImass	HDIlow	HDIhigh
b	1.0005987	1.0014396	0.0266824	0.0257804	0.0221034	18250.3	0.0000358	0.95	0.0200004	0.0358798
a	1.0002057	1.0007437	0.0191188	0.0194030	0.0197430	18581.4	0.0000376	0.95	0.0102840	0.0278988
M	1.0001383	1.0005915	29.9135982	30.0233000	30.4551942	18284.4	0.1085904	0.95	2.3797300	53.8417000
beta0[1]	0.9999616	0.9999770	-4.6156700	-4.5805300	-4.5549830	20001.0	0.0078355	0.95	-6.8268400	-2.4927600
beta0[2]	1.0007071	1.0027283	-15.8736286	-15.4389000	-14.7296581	14761.1	0.0285509	0.95	-22.7674000	-9.6091600
beta0[3]	1.0001097	1.0001368	-11.5024993	-11.2703000	-10.9849089	17031.9	0.0177635	0.95	-16.0781000	-7.2352200
beta0[4]	0.9999289	0.9999554	-17.1878076	-16.7305000	-15.6424285	13210.6	0.0315651	0.95	-24.6337000	-10.8712000
beta0[5]	1.0000036	1.0003567	-17.8310014	-17.3878000	-16.8716646	13667.2	0.0302098	0.95	-25.0428000	-11.7310000
beta[1]	1.0000025	1.0000524	1.4334775	1.4250200	1.4208944	20001.0	0.0020647	0.95	0.8765640	2.0197200
beta[2]	1.0006263	1.0025619	4.0512406	3.9516500	3.8572764	14819.3	0.0069188	0.95	2.5262100	5.7345300
beta[3]	1.0000355	1.0000458	3.6530290	3.5859000	3.4952487	16885.8	0.0051284	0.95	2.4302800	4.9615800
beta[4]	0.9999466	1.0000331	4.8175629	4.6949100	4.3647381	13251.0	0.0084041	0.95	3.0767500	6.7329000
beta[5]	0.9999831	1.0003200	4.8958519	4.7895100	4.6678375	13664.8	0.0077336	0.95	3.2749900	6.6935800
thresh[1,1]	NaN	NaN	0.5000000	0.5000000	0.4996355	0.0	NaN	0.95	0.5000000	0.5000000
thresh[2,1]	NaN	NaN	0.5000000	0.5000000	0.4996355	0.0	NaN	0.95	0.5000000	0.5000000
thresh[3,1]	NaN	NaN	0.5000000	0.5000000	0.4996355	0.0	NaN	0.95	0.5000000	0.5000000
thresh[4,1]	NaN	NaN	0.5000000	0.5000000	0.4996355	0.0	NaN	0.95	0.5000000	0.5000000
thresh[5,1]	NaN	NaN	0.5000000	0.5000000	0.4996355	0.0	NaN	0.95	0.5000000	0.5000000
thresh[3,2]	1.0001249	1.0002356	4.1838714	4.1239700	3.9989827	16752.3	0.0039747	0.95	3.2825800	5.2214100
thresh[5,2]	1.0001205	1.0008071	2.2692257	2.2347800	2.2051877	15020.5	0.0024751	0.95	1.7118500	2.8648300
thresh_age[1,1]	1.0001014	1.0006277	35.2968162	35.0517000	34.6217675	19056.0	0.0289958	0.95	27.4338000	43.2545000
thresh_age[2,1]	1.0003979	1.0016196	56.9385101	56.5019000	55.2024311	19292.9	0.0335480	0.95	48.5345000	66.1306000
thresh_age[3,1]	1.0001254	1.0004166	26.6809075	26.3114000	25.8743158	18138.7	0.0191536	0.95	22.2392000	32.1545000
thresh_age[4,1]	1.0004793	1.0012922	39.3769474	38.7832000	37.9660554	18140.1	0.0277368	0.95	33.1440000	47.0806000
thresh_age[5,1]	1.0002770	1.0008229	42.2491550	41.6725000	40.5524560	18103.3	0.0293820	0.95	35.6755000	50.5430000
thresh_age[3,2]	1.0002782	1.0011676	73.8563464	73.2947000	72.2792144	19384.5	0.0455717	0.95	61.9669000	86.1441000
thresh_age[5,2]	1.0001025	1.0005009	60.7997742	60.3701000	59.2686426	17962.4	0.0358762	0.95	51.9928000	70.1634000
age.s[1]	0.9999207	1.0000613	24.5385293	23.4096000	20.2682098	19070.6	0.0332201	0.95	19.0004000	33.4815000
age.s[2]	1.0001089	1.0006114	42.5122217	41.8597000	40.0419495	20001.0	0.0556096	0.95	27.6352000	58.0200000
age.s[3]	1.0002843	1.0007323	30.6369560	29.9289000	27.5384663	20001.0	0.0474045	0.95	19.0006000	42.8265000
age.s[4]	1.0000481	1.0005004	55.9006936	55.2031000	52.9337078	19034.6	0.0702890	0.95	38.0980000	75.5433000
age.s[5]	1.0003264	1.0011456	68.9327400	68.4588000	68.0661370	19580.6	0.0791705	0.95	48.2562000	90.8355000
age.s[6]	1.0000473	1.0001487	58.9407287	58.1956000	55.5103666	20001.0	0.0708717	0.95	40.0226000	78.8342000
age.s[7]	0.9999265	0.9999549	30.6883477	29.9731000	29.1625651	19257.5	0.0482091	0.95	19.0171000	42.7948000
age.s[8]	1.0006945	1.0015881	65.0337846	64.3058000	62.1982988	18313.7	0.0800268	0.95	44.2037000	86.1023000

age.s[9]	1.0000785	1.0004343	58.2918607	57.5874000	54.9218555	20001.0	0.0685985	0.95	40.0116000	77.6717000
age.s[10]	1.0002565	1.0005459	30.6465588	29.8776000	28.1105046	19411.2	0.0481577	0.95	19.0032000	42.8559000
age.s[11]	1.0005028	1.0012177	30.6368328	29.9833000	28.9412717	20001.0	0.0470991	0.95	19.0008000	42.5588000
age.s[12]	0.9999225	0.9999904	30.6875749	29.9494000	29.5954353	19582.0	0.0479380	0.95	19.0091000	42.8699000
age.s[13]	1.0004914	1.0017005	33.4941707	32.9313000	32.5536209	20001.0	0.0499707	0.95	19.6332000	46.5468000
age.s[14]	1.0002431	1.0009162	26.5925235	25.5517000	23.0186294	20001.0	0.0389221	0.95	19.0001000	37.0662000
age.s[15]	1.0000103	1.0004150	68.8022313	68.1845000	65.9578417	20001.0	0.0785285	0.95	47.9965000	90.8938000
age.s[16]	1.0001212	1.0005206	78.0955246	78.3097000	78.8556621	20001.0	0.0763335	0.95	59.2915000	99.2597000
age.s[17]	1.0000468	1.0003568	68.8649290	68.2889000	67.7521515	20001.0	0.0789680	0.95	47.8566000	91.2958000
age.s[18]	1.0001464	1.0008151	33.3800872	32.8471000	30.5830368	19312.0	0.0501127	0.95	19.7748000	46.2554000
age.s[19]	0.9999828	1.0002997	77.9503027	78.1347000	75.8629217	20001.0	0.0763108	0.95	59.6071000	99.6936000
age.s[20]	1.0005710	1.0016830	58.3574386	57.6500000	56.9415401	20001.0	0.0688796	0.95	40.0396000	77.7680000
age.s[21]	0.9999219	1.0000934	39.9453485	39.3042000	37.9245519	19223.2	0.0547462	0.95	25.7687000	55.0353000
age.s[22]	1.0000827	1.0006169	41.5382585	40.9964000	40.0089198	17588.0	0.0574799	0.95	27.6460000	56.9736000
age.s[23]	0.9999404	1.0001301	33.3967431	32.8428000	32.2866082	20001.0	0.0500347	0.95	19.7050000	46.4906000
age.s[24]	1.0001517	1.0002316	24.6262574	23.5047000	20.2427503	20001.0	0.0327307	0.95	19.0006000	33.6404000
age.s[25]	0.9999432	0.9999897	47.6389699	46.9445000	44.9564600	20001.0	0.0584678	0.95	32.8051000	64.7431000
age.s[26]	0.9998745	0.9999105	78.1815538	78.3552000	78.1147985	20001.0	0.0766784	0.95	59.8668000	99.7998000
age.s[27]	1.0000631	1.0005358	55.2346127	54.4944000	52.5014124	19238.0	0.0673841	0.95	38.0618000	73.9805000
age.s[28]	0.9998866	0.9999692	30.6146442	29.9035000	29.4808903	20001.0	0.0473051	0.95	19.0016000	42.7511000
age.s[29]	0.9999974	1.0000997	41.8254698	41.2265000	39.8450109	18485.9	0.0547264	0.95	28.3595000	56.9332000
age.s[30]	1.0000149	1.0002496	33.4293936	32.8999000	32.5155749	20001.0	0.0501118	0.95	20.0657000	46.8283000
age.s[31]	1.0001627	1.0007565	59.1146016	58.4549000	58.5776920	20001.0	0.0707416	0.95	40.4019000	79.1038000
age.s[32]	0.9999718	1.0000923	50.2897394	49.5307000	48.7860896	20001.0	0.0627192	0.95	33.5142000	67.3727000
age.s[33]	1.0000551	1.0005316	78.0490417	78.1826000	78.1634706	20001.0	0.0765973	0.95	59.5226000	99.3364000
age.s[34]	0.9999357	1.0000590	24.6249660	23.5564000	20.2920120	19537.3	0.0330151	0.95	19.0003000	33.6343000
age.s[35]	0.9999157	1.0000035	33.5681854	33.1182000	33.2883043	18908.9	0.0478881	0.95	21.5526000	46.7586000
age.s[36]	1.0001962	1.0010665	78.0880563	78.1926000	77.8662072	20001.0	0.0763914	0.95	59.2226000	99.3152000
age.s[37]	1.0002063	1.0011344	54.7201950	53.9963000	52.0856624	18846.0	0.0645183	0.95	38.0616000	72.2066000
age.s[38]	1.0000911	1.0003784	24.6188379	23.4627000	20.3022316	20384.2	0.0323470	0.95	19.0004000	33.7773000
age.s[39]	0.9999108	0.9999544	69.0003272	68.3666000	66.8350876	20001.0	0.0785446	0.95	47.8473000	90.7243000
age.s[40]	1.0003113	1.0015081	78.0869257	78.3746000	79.7781423	20001.0	0.0770857	0.95	59.7053000	99.7182000
age.s[41]	1.0002964	1.0010077	41.7773990	41.1707000	40.5349782	18000.9	0.0559008	0.95	28.0652000	56.9164000
age.s[42]	0.9999348	1.0000840	24.5972146	23.5020000	20.1593032	20001.0	0.0322894	0.95	19.0003000	33.5538000
age.s[43]	1.0001321	1.0006672	33.4688369	32.8897000	31.2610318	19496.8	0.0505568	0.95	20.1526000	46.9133000
age.s[44]	0.9999458	0.9999947	41.8203828	41.2487000	40.7542821	19428.1	0.0535781	0.95	27.7574000	56.7215000
age.s[45]	0.9999114	0.9999162	59.1983420	58.4081000	56.1544762	20001.0	0.0717036	0.95	41.1650000	80.1829000
age.s[46]	0.9999794	1.0000131	24.6224860	23.5008000	20.2907131	20001.0	0.0325481	0.95	19.0014000	33.6342000
age.s[47]	1.0005929	1.0021449	30.7347333	30.0191000	28.6061561	20001.0	0.0472350	0.95	19.1325000	42.8813000

age.s[48]	1.0001866	1.0010277	33.3859595	32.8240000	31.3671711	20001.0	0.0498417	0.95	20.5139000	47.2945000
age.s[49]	1.0004493	1.0010944	26.6168572	25.6054000	22.1203467	20001.0	0.0387587	0.95	19.0001000	36.9708000
age.s[50]	0.9999733	1.0002818	24.6266881	23.5171000	20.2377273	20001.0	0.0328291	0.95	19.0033000	33.7800000
age.s[51]	1.0000478	1.0000925	65.2445992	64.5362000	62.6544913	20001.0	0.0763100	0.95	44.6839000	86.3694000
age.s[52]	1.0001878	1.0004631	50.1750796	49.4108000	47.5121771	18735.9	0.0648884	0.95	34.2415000	68.3983000
age.s[53]	1.0001846	1.0010765	39.9602705	39.2768000	38.0435637	19491.9	0.0540663	0.95	26.0030000	55.0475000
age.s[54]	1.0003942	1.0010088	78.0016961	78.1287000	77.3413235	20001.0	0.0765003	0.95	59.0666000	99.2532000
age.s[55]	1.0001426	1.0007795	68.9040062	68.4461000	68.3824764	18927.3	0.0808259	0.95	48.0268000	90.6028000
age.s[56]	1.0004821	1.0012969	55.4219174	54.6505000	53.1996063	19203.6	0.0674535	0.95	38.2325000	74.1531000
age.s[57]	1.0000227	1.0001651	42.4092857	41.7792000	41.2432941	19437.9	0.0564280	0.95	27.9755000	58.4180000
age.s[58]	1.0000150	1.0004319	33.4410917	32.8660000	31.4659595	20001.0	0.0502039	0.95	20.0151000	46.7079000
age.s[59]	1.0001995	1.0009463	64.9353064	64.2199000	62.1809620	20001.0	0.0771254	0.95	44.6246000	86.7367000
age.s[60]	0.9999679	1.0000329	33.5513370	33.0338000	32.7318409	19226.6	0.0470315	0.95	21.0079000	46.0460000
age.s[61]	0.9999865	1.0002654	40.0175281	39.3487000	37.9921464	19568.0	0.0541116	0.95	26.2034000	55.1938000
age.s[62]	1.0001281	1.0008257	55.9604056	55.1697000	53.0875578	18656.1	0.0707402	0.95	37.7464000	74.8948000
age.s[63]	0.9999996	1.0002826	67.3871959	66.7813000	66.3064040	19344.1	0.0763019	0.95	48.1316000	89.3759000
age.s[64]	1.0000345	1.0005059	42.4824403	41.8418000	40.2796486	19603.9	0.0562960	0.95	27.6647000	58.1575000
age.s[65]	1.0004912	1.0021669	78.1447702	78.2705000	77.3350876	20001.0	0.0764899	0.95	59.6219000	99.3744000
age.s[66]	1.0002820	1.0008986	50.3017181	49.6018000	47.1693210	17875.5	0.0652378	0.95	34.3141000	67.7345000
age.s[67]	1.0003412	1.0014021	30.6515681	29.9102000	27.9038256	20457.3	0.0467622	0.95	19.0222000	43.0042000
age.s[68]	1.0003195	1.0008800	24.6165964	23.4928000	20.1017966	20001.0	0.0327006	0.95	19.0002000	33.6857000
age.s[69]	0.9999867	1.0001397	58.0545211	57.4939000	56.8862898	20001.0	0.0650678	0.95	41.5364000	77.0654000
age.s[70]	1.0004711	1.0014752	33.4596860	32.8918000	31.6304708	19593.0	0.0505746	0.95	20.7130000	47.4420000
age.s[71]	1.0003629	1.0006275	39.7526007	39.2470000	38.2183386	18991.6	0.0524141	0.95	26.8616000	54.9061000
age.s[72]	1.0005957	1.0010744	24.5976736	23.4698000	20.2062302	20001.0	0.0325413	0.95	19.0001000	33.6617000
age.s[73]	1.0007759	1.0029645	69.0391328	68.5962000	68.6454954	19302.7	0.0802210	0.95	48.1506000	91.2201000
age.s[74]	1.0007633	1.0027534	58.3270277	57.5684000	56.7561357	20001.0	0.0692006	0.95	40.2917000	78.0143000
age.s[75]	1.0002877	1.0011641	55.3940150	54.7935000	53.3423502	20001.0	0.0623225	0.95	39.0430000	73.0125000
age.s[76]	1.0000763	1.0005323	30.6862035	29.9950000	28.1727468	20001.0	0.0473091	0.95	19.0080000	42.8293000
age.s[77]	1.0003594	1.0015833	41.8631060	41.2915000	39.9452496	18107.3	0.0558573	0.95	28.1621000	57.4588000
age.s[78]	1.0001806	1.0009722	59.1284625	58.4836000	57.9138542	18782.2	0.0729807	0.95	40.6327000	79.1020000
age.s[79]	0.9999172	0.9999862	24.6179084	23.5195000	20.2119070	20001.0	0.0326744	0.95	19.0002000	33.6080000
age.s[80]	0.9999243	0.9999811	40.0388877	39.4242000	38.3152732	19467.9	0.0540630	0.95	25.9050000	55.0620000
age.s[81]	0.9999280	1.0001293	24.6185888	23.5365000	20.2800728	20001.0	0.0325140	0.95	19.0002000	33.7462000
age.s[82]	1.0001101	1.0003698	50.2608815	49.5842000	47.4437286	18808.8	0.0631376	0.95	34.3528000	67.8591000
age.s[83]	1.0000955	1.0004994	33.4191028	32.8940000	32.5423399	20009.4	0.0499384	0.95	19.8308000	46.3099000
age.s[84]	0.9999916	1.0002109	30.6637704	29.9433000	28.3581839	20001.0	0.0472152	0.95	19.0179000	42.7297000
age.s[85]	1.0002239	1.0010293	50.2673217	49.5562000	48.1650934	20062.2	0.0614489	0.95	34.3326000	67.8266000
age.s[86]	1.0007529	1.0024741	66.1543879	65.4782000	63.8322868	19915.2	0.0730563	0.95	46.9277000	86.9717000

age.s[87]	0.9999072	0.9999725	41.8540003	41.2997000	41.2300305	19402.1	0.0540523	0.95	28.0507000	57.0931000
age.s[88]	1.0000587	1.0004289	30.6007578	29.9073000	28.5413189	20001.0	0.0469735	0.95	19.0026000	42.6644000
age.s[89]	1.0004416	1.0018782	50.3289906	49.6395000	48.3784775	20001.0	0.0617013	0.95	34.3678000	68.0262000
age.s[90]	1.0002490	1.0012049	42.4736338	41.8263000	40.9675501	18922.8	0.0578324	0.95	27.5223000	58.2037000
age.s[91]	1.0000955	1.0001239	50.2850582	49.5978000	48.7788523	20001.0	0.0615534	0.95	34.7480000	68.0698000
age.s[92]	1.0004426	1.0013597	58.3287727	57.6509000	55.8428983	19105.4	0.0708392	0.95	40.3887000	78.1353000
age.s[93]	1.0002014	1.0002125	55.9184196	55.2323000	55.0447607	19223.2	0.0694950	0.95	38.4186000	75.3795000
age.s[94]	1.0002870	1.0010108	50.3433179	49.6664000	47.9411314	20001.0	0.0613315	0.95	34.3231000	67.7206000
age.s[95]	1.0001129	1.0007729	67.3389003	66.6640000	65.1878483	20001.0	0.0753532	0.95	47.4228000	88.8713000
age.s[96]	1.0000160	1.0001060	24.6099088	23.4492000	20.3690170	20001.0	0.0325935	0.95	19.0006000	33.6739000
age.s[97]	1.0006158	1.0020379	26.6656235	25.6663000	23.3105805	20001.0	0.0389469	0.95	19.0007000	37.0271000
age.s[98]	1.0010844	1.0026141	24.6173885	23.5157000	20.3269831	19500.6	0.0327385	0.95	19.0003000	33.5455000
age.s[99]	1.0003729	1.0013138	24.6311969	23.5281000	20.1102905	20001.0	0.0329061	0.95	19.0007000	33.7014000
age.s[100]	1.0001870	1.0009643	58.3255973	57.6218000	56.2582177	19308.3	0.0708663	0.95	39.8585000	77.3997000
age.s[101]	1.0005932	1.0016001	59.0956878	58.4370000	56.2998805	20001.0	0.0706654	0.95	39.8197000	78.3853000
age.s[102]	0.9999228	0.9999251	78.1843023	78.3693000	78.6516386	20001.0	0.0773507	0.95	59.7863000	99.9255000
age.s[103]	1.0009369	1.0029980	74.1393377	73.8452000	71.7483902	20001.0	0.0781517	0.95	54.1341000	96.3525000
age.s[104]	1.0002028	1.0008862	42.3738116	41.7581000	41.5133720	19492.9	0.0558521	0.95	28.0605000	58.0365000
age.s[105]	0.9999729	1.0001594	59.0221159	58.2734000	56.3072520	18931.4	0.0728907	0.95	39.8976000	78.6446000
age.s[106]	1.0004460	1.0010406	50.1771780	49.4175000	48.5447972	19487.5	0.0617512	0.95	33.8951000	67.1291000
age.s[107]	0.9999920	1.0003730	58.3325244	57.6351000	56.3958984	20001.0	0.0684831	0.95	40.5732000	77.5609000
age.s[108]	1.0000302	1.0004390	47.7118704	47.0019000	44.2580563	19505.5	0.0597379	0.95	32.1578000	64.3131000
age.s[109]	1.0001859	1.0010387	78.0141225	78.3157000	79.1173936	20001.0	0.0766519	0.95	59.4697000	99.5783000
age.s[110]	0.9999076	1.0000393	78.0113525	78.2583000	79.7179510	20001.0	0.0769540	0.95	58.9216000	99.1754000
age.s[111]	1.0006816	1.0017329	24.6078252	23.4819000	20.2202594	20001.0	0.0325938	0.95	19.0002000	33.6620000
age.s[112]	1.0000974	1.0002831	30.6580259	30.0288000	29.7319931	20001.0	0.0469278	0.95	19.0039000	42.7681000
age.s[113]	1.0001468	1.0005893	30.6092342	29.9170000	27.3219879	19273.0	0.0477482	0.95	19.0007000	42.6426000
age.s[114]	1.0000821	1.0006457	55.3623518	54.7567000	52.8470631	19298.2	0.0644810	0.95	38.7035000	73.0531000
age.s[115]	1.0007781	1.0029213	33.3409371	32.8243000	30.7364764	17868.8	0.0526856	0.95	20.2484000	46.6623000
age.s[116]	1.0008693	1.0027964	55.3184282	54.5738000	53.5750460	18342.7	0.0693115	0.95	37.9903000	74.2360000
age.s[117]	1.0002814	1.0006294	24.6455787	23.5054000	20.1852199	19079.4	0.0339133	0.95	19.0001000	33.7684000
age.s[118]	1.0000880	1.0004436	26.6203771	25.6245000	21.0574049	20001.0	0.0389819	0.95	19.0010000	37.0133000
age.s[119]	1.0000148	1.0003531	78.1776253	78.3136000	77.1753774	20001.0	0.0765567	0.95	59.3215000	99.2349000
age.s[120]	1.0001164	1.0007018	67.5593473	66.9024000	64.9233752	20001.0	0.0750981	0.95	47.7931000	88.9907000
age.s[121]	1.0006069	1.0024796	58.2739156	57.5532000	55.3051170	19568.6	0.0696828	0.95	40.4289000	77.8602000
age.s[122]	1.0009879	1.0038831	78.0585666	78.1818000	76.6404060	20001.0	0.0769784	0.95	58.8725000	99.1104000
age.s[123]	1.0001025	1.0001832	41.7072778	41.0815000	39.3065248	19336.6	0.0530520	0.95	28.1306000	56.5078000
age.s[124]	1.0000771	1.0005336	30.7305920	30.0642000	29.7414193	20001.0	0.0471541	0.95	19.0126000	42.6950000
age.s[125]	1.0001266	1.0004754	30.6219631	29.9011000	28.1425195	20001.0	0.0473285	0.95	19.0504000	42.8100000

age.s[126]	1.0001271	1.0006312	47.7042611	46.9954000	45.6538884	20001.0	0.0587422	0.95	32.1280000	64.2402000
age.s[127]	1.0003012	1.0010908	24.5810871	23.4424000	20.1951041	19383.5	0.0331453	0.95	19.0009000	33.6303000
age.s[128]	0.9999906	1.0002467	58.1068327	57.4611000	54.0153747	20001.0	0.0657819	0.95	40.8175000	76.5242000
age.s[129]	1.0004556	1.0018510	65.0654172	64.3117000	63.5582610	20001.0	0.0773587	0.95	44.7466000	87.1212000
age.s[130]	0.9998713	0.9999141	58.1281801	57.5046000	57.1173474	20001.0	0.0654761	0.95	40.6930000	76.1966000
age.s[131]	1.0000193	1.0002689	30.6680486	29.9723000	28.3928169	20001.0	0.0472452	0.95	19.0146000	42.6457000
age.s[132]	1.0001993	1.0010242	40.2777274	39.6075000	39.1226501	18564.1	0.0519985	0.95	26.8636000	54.2855000
age.s[133]	1.0001955	1.0010090	69.0996719	68.6514000	69.0555176	20001.0	0.0790614	0.95	48.0757000	91.3064000
age.s[134]	1.0002462	1.0005778	33.3766490	32.7961000	31.2128828	17994.6	0.0529378	0.95	19.9900000	46.7037000
age.s[135]	1.0001951	1.0010313	50.2999340	49.6184000	48.6285653	18134.1	0.0646938	0.95	34.0508000	67.2484000
age.s[136]	1.0003004	1.0011225	47.7221727	47.0039000	45.6067916	20001.0	0.0586734	0.95	32.2000000	64.1045000
age.s[137]	1.0006844	1.0019557	55.3458669	54.6385000	53.0959813	19630.7	0.0672236	0.95	37.8738000	74.1419000
age.s[138]	1.0005538	1.0019950	24.6063594	23.4682000	20.1882623	20001.0	0.0327390	0.95	19.0003000	33.7237000
age.s[139]	0.9999640	1.0000225	66.2225743	65.7044000	64.9328688	19314.6	0.0737958	0.95	46.5064000	86.2385000
age.s[140]	1.0009806	1.0026865	33.4205723	32.8289000	32.3274270	19436.7	0.0508515	0.95	19.8380000	46.7060000
age.s[141]	0.9999359	1.0000505	26.6281164	25.5862000	23.7560728	19206.0	0.0399156	0.95	19.0023000	37.1836000
age.s[142]	1.0000400	1.0004578	30.6540029	29.9741000	29.2796775	19565.7	0.0476454	0.95	19.0026000	42.7474000
age.s[143]	0.9999905	1.0002271	24.6484291	23.5458000	20.1590726	20001.0	0.0326572	0.95	19.0002000	33.7518000
age.s[144]	1.0001319	1.0007015	78.1213636	78.2112000	76.8621932	20001.0	0.0761898	0.95	59.8432000	99.9115000
age.s[145]	1.0000924	1.0007006	50.2795002	49.6047000	49.0577974	19602.0	0.0621954	0.95	34.0745000	67.4797000
age.s[146]	0.9999730	1.0000954	33.4597130	32.9452000	32.8145092	19466.5	0.0508368	0.95	19.7363000	46.4881000
age.s[147]	1.0000046	1.0002846	59.0091134	58.3077000	55.3549081	19476.5	0.0723024	0.95	40.0593000	78.9820000
age.s[148]	1.0005170	1.0021689	42.4182184	41.7032000	40.5780103	20001.0	0.0555097	0.95	28.0717000	58.3092000
age.s[149]	1.0000611	1.0003274	58.3316982	57.6285000	56.9132824	19122.2	0.0707456	0.95	40.1861000	77.5291000
age.s[150]	1.0000220	1.0002773	66.1889184	65.5356000	64.7615721	19583.2	0.0742384	0.95	46.8041000	86.8185000
age.s[151]	0.9998747	0.9999269	33.4574725	32.9412000	33.1947157	20001.0	0.0497477	0.95	20.1850000	46.8312000
age.s[152]	0.9998786	0.9999183	40.0246698	39.3505000	39.0160967	19473.9	0.0543203	0.95	25.7045000	54.8076000
age.s[153]	1.0001294	1.0007854	58.5042884	57.7635000	55.8673196	20001.0	0.0699836	0.95	40.1866000	78.3360000
age.s[154]	0.9999296	0.9999997	47.6853063	47.0266000	44.6440103	19388.7	0.0592835	0.95	32.1541000	64.0067000
age.s[155]	0.9999115	1.0000178	26.6284844	25.6375000	21.3142930	20001.0	0.0388936	0.95	19.0045000	37.0102000
age.s[156]	1.0012974	1.0045542	55.3669333	54.6306000	52.6622376	19224.5	0.0678529	0.95	37.2873000	73.6152000
age.s[157]	1.0010915	1.0039591	58.4259747	57.7455000	55.0231527	18350.0	0.0717773	0.95	40.7229000	78.3674000
age.s[158]	1.0000125	1.0003876	58.2936624	57.5560000	55.8384148	18540.7	0.0719746	0.95	40.4226000	77.9234000
age.s[159]	1.0006017	1.0016234	42.4618991	41.7489000	39.8906224	18966.5	0.0576798	0.95	27.9948000	58.6598000
age.s[160]	1.0000411	1.0005408	42.1956611	41.5027000	39.2422403	19352.7	0.0531367	0.95	28.9832000	57.1199000
age.s[161]	0.9999797	1.0002616	33.4213229	32.8489000	31.5568861	19358.2	0.0508203	0.95	19.8396000	46.5011000
age.s[162]	1.0002882	1.0013334	33.4251106	32.8540000	31.0468229	20001.0	0.0495617	0.95	20.5665000	47.1370000
age.s[163]	1.0001710	1.0003828	39.9886003	39.3712000	38.9470278	20001.0	0.0532363	0.95	26.2764000	55.3636000
age.s[164]	1.0002323	1.0010424	26.6139560	25.6171000	22.2581277	20001.0	0.0387304	0.95	19.0014000	37.0124000

age.s[165]	1.0000482	1.0003691	42.3847258	41.7250000	40.3709773	19552.9	0.0562656	0.95	27.9796000	58.3661000
age.s[166]	1.0001026	1.0002624	24.6156085	23.5423000	20.1425762	20001.0	0.0325826	0.95	19.0003000	33.5904000
age.s[167]	1.0000019	1.0000827	68.9293757	68.3486000	67.9827734	19320.7	0.0797176	0.95	48.1750000	90.9045000
age.s[168]	1.0001553	1.0006454	58.2719012	57.5862000	56.4422540	19272.9	0.0701193	0.95	39.9647000	77.3728000
age.s[169]	1.0002119	1.0011830	42.4666152	41.8618000	40.1810835	20001.0	0.0550749	0.95	27.8082000	57.9580000
age.s[170]	1.0000704	1.0004466	42.1785061	41.5668000	40.5348092	19073.6	0.0542991	0.95	28.1772000	57.1703000
age.s[171]	0.9999617	1.0000336	33.4112764	32.9084000	32.4934893	20001.0	0.0497288	0.95	20.1862000	46.6792000
age.s[172]	0.9999497	1.0000677	30.6328756	29.8923000	28.3415438	20633.3	0.0462162	0.95	19.0764000	42.7005000
age.s[173]	1.0002796	1.0010136	33.3693224	32.8353000	31.6133259	20001.0	0.0498695	0.95	20.0935000	46.7006000
age.s[174]	1.0006187	1.0024151	50.4425391	49.7864000	48.5918254	20001.0	0.0617226	0.95	33.7503000	67.3546000
age.s[175]	1.0001080	1.0006862	30.6528473	29.9752000	29.1015453	19389.8	0.0476220	0.95	19.0101000	42.6325000
age.s[176]	1.0004937	1.0019660	55.3028068	54.6381000	54.4834802	20001.0	0.0658275	0.95	38.1919000	73.9045000
age.s[177]	1.0009461	1.0030687	47.7846878	47.0976000	44.9419367	19458.9	0.0609135	0.95	31.8796000	64.3080000
age.s[178]	1.0000336	1.0003463	58.3067267	57.5407000	56.0393367	19319.7	0.0706843	0.95	40.4976000	78.3080000
age.s[179]	1.0004393	1.0010105	24.5961772	23.4699000	20.1726576	20412.7	0.0324060	0.95	19.0000000	33.6788000
age.s[180]	1.0000228	1.0000481	40.0122969	39.3936000	37.9643035	19325.5	0.0545289	0.95	26.3054000	55.4860000
age.s[181]	1.0003797	1.0015707	69.0464686	68.4368000	67.6967566	20001.0	0.0788431	0.95	48.6363000	91.6428000
age.s[182]	1.0003873	1.0012289	68.9999439	68.3764000	65.9070980	18857.5	0.0818518	0.95	49.0010000	92.3309000
age.s[183]	1.0001414	1.0006124	77.9488152	78.0520000	77.7049225	20001.0	0.0764277	0.95	58.9091000	99.2363000
age.s[184]	1.0001719	1.0009453	30.6281748	29.9135000	29.0759014	20001.0	0.0474882	0.95	19.0355000	42.9650000
age.s[185]	1.0002232	1.0007625	74.0891669	73.7868000	72.7674284	18902.2	0.0804211	0.95	53.6143000	96.0206000
age.s[186]	1.0002916	1.0009738	59.0942213	58.2847000	55.6765548	18879.8	0.0735564	0.95	40.3207000	78.9743000
age.s[187]	1.0002861	1.0013908	48.3455800	47.7323000	47.5269538	18083.4	0.0631196	0.95	32.0556000	64.8565000
age.s[188]	1.0003679	1.0012095	68.8727525	68.3161000	66.6299506	20001.0	0.0789917	0.95	47.0036000	90.4730000
age.s[189]	0.9999018	1.0000329	78.0226224	78.1862000	78.7751879	20001.0	0.0765503	0.95	58.9178000	98.9985000
age.s[190]	0.9999040	1.0000381	33.4971812	32.9154000	32.3699462	20001.0	0.0503150	0.95	20.0733000	46.8411000
age.s[191]	1.0003152	1.0008081	39.7489610	39.1250000	38.0548774	18313.7	0.0532803	0.95	26.4734000	54.3923000
age.s[192]	1.0001257	1.0008631	50.2795392	49.5805000	48.2047037	20001.0	0.0611484	0.95	34.5783000	67.8707000
age.s[193]	1.0001212	1.0004147	33.4063142	32.8042000	31.2434630	20001.0	0.0502213	0.95	20.3651000	47.2150000
age.s[194]	1.0000850	1.0006511	55.3870954	54.6729000	53.6655816	20001.0	0.0666544	0.95	37.4014000	73.5353000
age.s[195]	0.9999159	0.9999498	68.8831884	68.3154000	67.6299892	20001.0	0.0785990	0.95	48.0563000	91.0205000
age.s[196]	1.0003389	1.0013826	50.3210786	49.5861000	47.8311950	18763.6	0.0639359	0.95	34.2556000	67.7831000
age.s[197]	0.9999083	0.9999814	50.2557196	49.5343000	48.2877193	20001.0	0.0611701	0.95	34.4425000	67.8776000
age.s[198]	1.0004359	1.0010750	24.6335759	23.5103000	20.1525170	19945.9	0.0331445	0.95	19.0000000	33.7951000
age.s[199]	1.0000609	1.0005248	55.4098807	54.7161000	52.8045473	19522.9	0.0673160	0.95	37.6692000	73.8569000
age.s[200]	1.0008502	1.0028729	24.6392472	23.5197000	20.2615773	18142.6	0.0342143	0.95	19.0002000	33.6576000

2.1.3 10 traits

Table 19: MCMC diagnostic data for CAMSAD dataset (n traits = 10).

	PSRF Point est.	PSRF Upper C.I.	Mean	Median	Mode	ESS	MCSE	HDImass	HDIlow	HDIhigh
b	1.0007903	1.0014732	0.0296245	0.0294724	0.0290899	18231.7	0.0000304	0.95	0.0214769	0.0374170
a	1.0005192	1.0011433	0.0157089	0.0153514	0.0139568	18169.7	0.0000296	0.95	0.0081897	0.0236204
M	1.0005237	1.0011594	39.7103861	41.1308000	44.9481529	18261.6	0.0807520	0.95	17.1152000	58.8953000
beta0[1]	1.0000855	1.0002295	-4.2038935	-4.1863100	-4.1890447	20389.9	0.0067325	0.95	-6.1119000	-2.3702400
beta0[2]	0.9999299	0.9999801	-14.6665616	-14.4698000	-13.8823689	17860.6	0.0194360	0.95	-19.8227000	-9.8752800
beta0[3]	1.0001096	1.0007897	-9.1755547	-9.1042800	-9.2735754	19359.7	0.0108222	0.95	-12.1727000	-6.2977500
beta0[4]	1.0002397	1.0005375	-12.6421825	-12.5198000	-12.4524966	20001.0	0.0133869	0.95	-16.4183000	-9.0264600
beta0[5]	1.0002922	1.0011694	-18.8800633	-18.6846000	-18.3728623	17642.2	0.0201128	0.95	-24.2865000	-13.9130000
beta0[6]	1.0003536	1.0011128	-11.5438480	-11.3846000	-11.0168561	17864.2	0.0152236	0.95	-15.6412000	-7.8061100
beta0[7]	1.0007107	1.0028530	-20.8329901	-20.6252000	-19.7811257	16755.5	0.0227542	0.95	-26.7140000	-15.3767000
beta0[8]	1.0028263	1.0090830	-27.1081653	-26.8064000	-26.2155933	11800.2	0.0375016	0.95	-35.4574000	-19.8310000
beta0[9]	1.0001555	1.0008351	-12.2349198	-12.1154000	-11.8157687	19536.2	0.0132542	0.95	-15.9915000	-8.7638500
beta0[10]	1.0008563	1.0033733	-27.3416946	-27.0484000	-26.0074944	12138.3	0.0362594	0.95	-35.3697000	-20.0527000
beta[1]	1.0000104	1.0000996	1.3082287	1.3047000	1.3187666	20874.5	0.0017358	0.95	0.8276820	1.8042800
beta[2]	0.9999253	0.9999781	3.6906946	3.6448300	3.5530662	18395.2	0.0046224	0.95	2.5280300	4.9323800
beta[3]	1.0002288	1.0012180	2.9550048	2.9341600	2.9056828	19601.8	0.0030151	0.95	2.1198100	3.7672800
beta[4]	1.0002146	1.0006220	3.5351091	3.5040800	3.4656954	20449.9	0.0034197	0.95	2.6003300	4.5095600
beta[5]	1.0001621	1.0008355	5.0816704	5.0351700	4.9332017	17905.8	0.0049547	0.95	3.8505000	6.4323300
beta[6]	1.0002806	1.0009756	2.9152448	2.8801900	2.8174702	19166.7	0.0035571	0.95	2.0047600	3.8999500
beta[7]	1.0006562	1.0026572	5.6543017	5.6041500	5.4754003	17144.4	0.0056567	0.95	4.2210100	7.0859500
beta[8]	1.0029985	1.0095373	7.2142331	7.1411600	6.9725350	11901.2	0.0093704	0.95	5.3106000	9.2467700
beta[9]	1.0001009	1.0006174	3.2817613	3.2554500	3.1616635	20001.0	0.0032560	0.95	2.3675400	4.1655300
beta[10]	1.0008082	1.0031052	7.3944535	7.3186800	7.1025176	12275.4	0.0092052	0.95	5.4694600	9.3812500
thresh[1,1]	NaN	NaN	0.5000000	0.5000000	0.4996355	0.0	NaN	0.95	0.5000000	0.5000000
thresh[2,1]	NaN	NaN	0.5000000	0.5000000	0.4996355	0.0	NaN	0.95	0.5000000	0.5000000
thresh[3,1]	NaN	NaN	0.5000000	0.5000000	0.4996355	0.0	NaN	0.95	0.5000000	0.5000000
thresh[4,1]	NaN	NaN	0.5000000	0.5000000	0.4996355	0.0	NaN	0.95	0.5000000	0.5000000
thresh[5,1]	NaN	NaN	0.5000000	0.5000000	0.4996355	0.0	NaN	0.95	0.5000000	0.5000000
thresh[6,1]	NaN	NaN	0.5000000	0.5000000	0.4996355	0.0	NaN	0.95	0.5000000	0.5000000
thresh[7,1]	NaN	NaN	0.5000000	0.5000000	0.4996355	0.0	NaN	0.95	0.5000000	0.5000000
thresh[8,1]	NaN	NaN	0.5000000	0.5000000	0.4996355	0.0	NaN	0.95	0.5000000	0.5000000
thresh[9,1]	NaN	NaN	0.5000000	0.5000000	0.4996355	0.0	NaN	0.95	0.5000000	0.5000000
thresh[10,1]	NaN	NaN	0.5000000	0.5000000	0.4996355	0.0	NaN	0.95	0.5000000	0.5000000
thresh[3,2]	1.0003110	1.0015456	3.7863267	3.7661500	3.7013359	20001.0	0.0023696	0.95	3.1353800	4.4537300
thresh[5,2]	0.9998652	0.9998804	2.3019185	2.2878400	2.2541461	19299.7	0.0016533	0.95	1.8630800	2.7599200

thresh[7,2]	1.0000930	1.0006728	2.1272838	2.1137600	2.0963352	19201.2	0.0016992	0.95	1.6656800	2.5804200
thresh[8,2]	1.0012509	1.0042935	3.5286251	3.4984100	3.4490020	15317.9	0.0032891	0.95	2.7552700	4.3285500
thresh[10,2]	1.0004001	1.0015456	3.5762235	3.5496800	3.5525765	15565.0	0.0033821	0.95	2.7789700	4.4245800
thresh_age[1,1]	1.0005523	1.0011629	36.2288399	36.2281000	36.2008451	19114.9	0.0269589	0.95	28.9252000	43.4166000
thresh_age[2,1]	1.0000817	1.0003859	60.9476369	60.9306000	60.8935555	19427.5	0.0278691	0.95	53.2495000	68.3888000
thresh_age[3,1]	1.0003551	1.0012528	26.3470495	26.2664000	26.1549552	18159.9	0.0163513	0.95	22.1669000	30.7707000
thresh_age[4,1]	1.0003406	1.0005594	41.1388638	41.0233000	40.6389834	18061.2	0.0221778	0.95	35.5501000	47.0380000
thresh_age[5,1]	1.0004636	1.0016521	45.2579734	45.1832000	44.9868341	17767.3	0.0237294	0.95	39.1863000	51.3763000
thresh_age[6,1]	1.0000867	1.0004646	62.3617149	62.2487000	61.5748208	19044.0	0.0306381	0.95	54.2649000	70.7726000
thresh_age[7,1]	1.0004317	1.0013188	43.4625585	43.3562000	42.7322435	17561.0	0.0232792	0.95	37.4959000	49.4324000
thresh_age[8,1]	1.0003459	1.0015110	45.8853418	45.8620000	46.1738100	17237.5	0.0240869	0.95	39.8299000	52.1178000
thresh_age[9,1]	1.0005566	1.0017974	48.4041684	48.3649000	48.6218985	18511.8	0.0246546	0.95	41.9192000	54.8976000
thresh_age[10,1]	1.0006757	1.0017582	43.1611283	43.0838000	43.2332820	17463.0	0.0231512	0.95	37.3121000	49.2017000
thresh_age[3,2]	1.0004719	1.0016717	80.8591112	80.4898000	79.3712288	18774.2	0.0459890	0.95	69.0029000	93.4344000
thresh_age[5,2]	1.0001733	1.0005638	64.6542833	64.7114000	64.6528986	19301.2	0.0275891	0.95	57.3234000	72.2557000
thresh_age[7,2]	1.0000670	1.0005673	58.0322795	57.9913000	57.7271127	18656.5	0.0261506	0.95	51.3315000	65.2524000
thresh_age[8,2]	1.0003271	1.0015385	69.9519731	70.0174000	69.7874602	19483.7	0.0279578	0.95	62.2036000	77.3997000
thresh_age[10,2]	1.0003395	1.0013728	65.5158166	65.5672000	65.6358616	19104.6	0.0272868	0.95	58.1440000	72.8511000
age.s[1]	1.0001116	1.0002648	24.6418986	23.6446000	20.2098645	20001.0	0.0311586	0.95	19.0000000	33.2117000
age.s[2]	1.0001064	1.0006375	43.5103187	43.3147000	43.1062135	18720.2	0.0357993	0.95	33.9647000	53.0140000
age.s[3]	1.0000445	1.0004920	28.9205652	28.5533000	28.2228698	20001.0	0.0393458	0.95	19.0190000	38.6855000
age.s[4]	1.0005922	1.0021552	57.9981755	57.7905000	57.6697098	19413.9	0.0436284	0.95	46.1727000	69.7921000
age.s[5]	1.0002115	1.0007489	81.7690797	81.7152000	82.2575273	20001.0	0.0593586	0.95	66.2438000	97.9714000
age.s[6]	1.0004706	1.0009989	62.9754013	62.7613000	62.2738442	20001.0	0.0465266	0.95	49.6373000	75.3735000
age.s[7]	1.0002423	1.0005396	28.8709657	28.5087000	28.1287221	20001.0	0.0393220	0.95	19.0003000	38.6865000
age.s[8]	1.0001206	1.0004751	58.1387060	57.9213000	57.7272473	19489.2	0.0434032	0.95	46.2438000	69.9274000
age.s[9]	1.0003653	1.0013026	53.8556031	53.6245000	53.6229792	19289.7	0.0402327	0.95	42.6246000	64.4714000
age.s[10]	0.9999992	1.0002519	28.8574171	28.4776000	27.9358619	19665.7	0.0395828	0.95	19.0106000	38.6809000
age.s[11]	1.0000426	1.0002432	28.9325148	28.5430000	28.4746848	20001.0	0.0395912	0.95	19.0053000	38.7462000
age.s[12]	1.0003413	1.0014675	47.6744910	47.5193000	48.0747509	18657.4	0.0373058	0.95	37.7692000	57.5045000
age.s[13]	1.0000106	1.0002920	30.9278052	30.8397000	31.3412881	19390.9	0.0407115	0.95	19.7740000	41.0168000
age.s[14]	1.0005527	1.0016746	26.3650538	25.5451000	22.8304932	19503.4	0.0363086	0.95	19.0003000	35.7998000
age.s[15]	1.0000998	1.0005493	70.0212264	69.7088000	69.2561164	19435.6	0.0519772	0.95	55.8876000	84.3023000
age.s[16]	0.9999760	0.9999982	72.4602068	72.1049000	71.1404376	20001.0	0.0515760	0.95	58.4767000	86.8992000
age.s[17]	1.0002417	1.0007093	81.9293305	81.9323000	81.8343510	20001.0	0.0593528	0.95	67.0591000	98.7364000
age.s[18]	1.0000240	1.0001548	30.8712991	30.7543000	30.7816648	19274.7	0.0406337	0.95	19.9905000	41.2504000
age.s[19]	1.0001543	1.0008430	85.4595969	85.8503000	87.0446762	20001.0	0.0565551	0.95	71.7295000	99.9974000
age.s[20]	1.0004154	1.0008592	62.1828383	61.9885000	61.6236813	20001.0	0.0453618	0.95	49.9084000	75.0602000
age.s[21]	0.9999082	0.9999190	34.2617175	34.2454000	34.3232825	19158.9	0.0388184	0.95	23.6835000	44.6798000

age.s[22]	1.0002839	1.0011001	59.9477947	59.6928000	59.3346764	18939.7	0.0443553	0.95	48.6127000	72.3242000
age.s[23]	0.9999691	1.0001293	30.9204829	30.8047000	30.7988061	20001.0	0.0397965	0.95	19.5246000	40.7875000
age.s[24]	1.0002404	1.0009405	24.6463243	23.6349000	20.3006175	19519.6	0.0318027	0.95	19.0004000	33.3102000
age.s[25]	1.0001925	1.0008451	54.8305984	54.6149000	54.4758015	18987.5	0.0423887	0.95	43.8267000	66.5868000
age.s[26]	1.0000062	1.0003243	85.4439476	85.8320000	86.2523010	20001.0	0.0566808	0.95	71.6444000	99.9890000
age.s[27]	1.0008492	1.0035146	70.8308081	70.5192000	70.0789853	20001.0	0.0515859	0.95	56.8998000	85.3831000
age.s[28]	1.0007519	1.0029621	36.9519068	36.8321000	36.4664943	18709.3	0.0357407	0.95	27.3871000	46.5072000
age.s[29]	1.0004342	1.0014868	50.9676603	50.7654000	50.0806892	19187.0	0.0387062	0.95	40.5778000	61.5706000
age.s[30]	0.9998947	0.9999783	30.8774832	30.7311000	29.9745521	19075.4	0.0412310	0.95	19.5406000	40.9227000
age.s[31]	1.0000764	1.0003109	58.9605463	58.6935000	58.2126766	20001.0	0.0435126	0.95	46.8705000	70.7632000
age.s[32]	0.9999810	1.0001421	67.9544483	67.6386000	65.9275882	20001.0	0.0493324	0.95	54.6849000	81.9773000
age.s[33]	1.0001969	1.0010695	85.3773251	85.7525000	87.3925894	20001.0	0.0567792	0.95	71.4714000	99.9939000
age.s[34]	1.0000544	1.0006021	24.5845784	23.5893000	20.1567913	19291.9	0.0317591	0.95	19.0001000	33.0740000
age.s[35]	1.0004162	1.0017819	30.6914433	30.5854000	31.1533550	19325.1	0.0385963	0.95	20.2803000	40.7308000
age.s[36]	1.0000470	1.0004902	75.8590493	75.4820000	74.4318520	19456.7	0.0565318	0.95	61.0413000	91.8089000
age.s[37]	1.0003268	1.0013493	73.9222836	73.4932000	73.0857416	20001.0	0.0542494	0.95	59.5162000	89.4175000
age.s[38]	1.0001174	1.0004056	24.6778756	23.6394000	20.1278063	20001.0	0.0316503	0.95	19.0006000	33.5012000
age.s[39]	1.0004824	1.0021219	81.9049177	81.8377000	82.1065601	20001.0	0.0596374	0.95	67.5079000	99.1608000
age.s[40]	1.0002108	1.0008343	81.5505666	81.4666000	81.5676090	20001.0	0.0589874	0.95	66.6198000	98.2749000
age.s[41]	1.0001218	1.0008122	45.3593251	45.2005000	44.9431746	18578.0	0.0361499	0.95	35.8941000	55.0359000
age.s[42]	0.9998856	0.9999487	24.6774149	23.6334000	20.2415072	20001.0	0.0317069	0.95	19.0002000	33.5173000
age.s[43]	0.9999716	1.0001599	30.8680831	30.6885000	30.5202836	19258.7	0.0408626	0.95	19.5356000	40.9559000
age.s[44]	0.9999196	1.0000518	51.1950970	50.9631000	50.9975953	18547.7	0.0402068	0.95	41.1596000	62.3288000
age.s[45]	1.0006354	1.0022392	69.2058026	68.8484000	67.8468294	20001.0	0.0505820	0.95	55.3630000	83.2611000
age.s[46]	1.0006060	1.0020057	24.6762811	23.6529000	20.1731140	20001.0	0.0315704	0.95	19.0006000	33.4914000
age.s[47]	0.9999548	1.0002055	28.8787239	28.5329000	28.8486702	20001.0	0.0396104	0.95	19.0040000	38.6579000
age.s[48]	1.0001027	1.0004570	35.7037491	35.7059000	35.8636799	18738.0	0.0378147	0.95	25.6705000	45.8423000
age.s[49]	1.0001810	1.0005659	26.3683835	25.5643000	22.9106199	20001.0	0.0358431	0.95	19.0018000	35.8853000
age.s[50]	0.9999860	1.0001938	24.6255562	23.6481000	20.1617118	19600.8	0.0315846	0.95	19.0000000	33.2115000
age.s[51]	1.0002876	1.0009121	71.4436046	71.1005000	70.5935833	19595.8	0.0536236	0.95	57.0615000	86.2156000
age.s[52]	1.0002994	1.0011321	53.1458959	52.8697000	52.4557120	19461.1	0.0406584	0.95	42.1910000	64.2255000
age.s[53]	1.0001938	1.0009364	45.0970181	44.8949000	44.6933206	18792.2	0.0361286	0.95	35.5319000	54.9122000
age.s[54]	0.9999138	0.9999681	61.7559504	61.5612000	61.2273402	20001.0	0.0443345	0.95	49.7547000	74.0737000
age.s[55]	0.9999552	1.0002093	57.1011481	56.8749000	55.5126731	19564.2	0.0426359	0.95	45.6534000	68.7053000
age.s[56]	1.0000826	1.0003958	73.7079360	73.3115000	71.7685785	20001.0	0.0545977	0.95	58.5788000	88.6668000
age.s[57]	1.0001216	1.0006685	49.2298715	49.1065000	49.0214558	17577.9	0.0387262	0.95	39.6852000	59.7304000
age.s[58]	1.0006633	1.0027366	30.8548714	30.7234000	30.8802194	19588.6	0.0402851	0.95	20.0973000	41.2719000
age.s[59]	0.9998887	0.9999625	80.0595466	79.7999000	79.1419205	20001.0	0.0596284	0.95	64.0463000	96.5733000
age.s[60]	0.9998804	0.9999257	30.6760349	30.4995000	30.3318886	18365.6	0.0398211	0.95	20.3280000	40.9199000

age.s[61]	1.0003968	1.0016535	34.2101954	34.1381000	33.8602476	18081.9	0.0395171	0.95	23.7825000	44.3332000
age.s[62]	1.0004347	1.0013951	61.8443821	61.5876000	60.3937126	18989.2	0.0471129	0.95	49.4446000	74.8933000
age.s[63]	0.9999689	1.0000132	80.2373654	80.0386000	79.0702423	20001.0	0.0584746	0.95	65.0307000	96.8748000
age.s[64]	1.0000655	1.0004072	49.0710085	48.8964000	47.9935315	18443.7	0.0383669	0.95	39.1407000	59.2858000
age.s[65]	1.0000133	1.0003629	72.8444048	72.5294000	70.7671247	20001.0	0.0533704	0.95	58.0805000	87.5460000
age.s[66]	1.0000849	1.0004017	67.9161345	67.6290000	66.8051697	19078.4	0.0490405	0.95	54.8208000	81.2032000
age.s[67]	0.9999632	1.0002568	28.8561428	28.4418000	27.1154272	18901.5	0.0406424	0.95	19.0136000	38.7312000
age.s[68]	1.0000127	1.0004174	24.6091808	23.5693000	20.1544843	19414.2	0.0318261	0.95	19.0003000	33.2479000
age.s[69]	1.0002834	1.0003864	61.7549964	61.5564000	60.8873863	19803.1	0.0450694	0.95	49.2383000	74.1320000
age.s[70]	1.0000306	1.0004535	30.8505446	30.6654000	31.1002613	21060.0	0.0394558	0.95	19.5270000	41.0116000
age.s[71]	0.9998841	0.9999089	45.6938203	45.4867000	45.1974834	18905.3	0.0359239	0.95	36.4718000	55.6517000
age.s[72]	1.0003779	1.0009417	24.6605735	23.6412000	20.1746737	20001.0	0.0314069	0.95	19.0012000	33.3755000
age.s[73]	1.0003938	1.0016487	78.0164223	77.6939000	77.1715967	20001.0	0.0583854	0.95	62.6942000	94.8383000
age.s[74]	0.9999200	1.0000578	56.2765004	56.0855000	55.5722342	20001.0	0.0421262	0.95	44.7392000	67.7797000
age.s[75]	0.9999743	1.0002961	52.9132978	52.7260000	52.5620893	19169.0	0.0401171	0.95	42.3693000	63.9991000
age.s[76]	1.0010864	1.0037024	28.9439862	28.5477000	27.3398390	19295.8	0.0403770	0.95	19.0198000	38.7627000
age.s[77]	1.0000703	1.0001413	37.5441222	37.3986000	36.8841066	19686.9	0.0349566	0.95	28.1217000	47.3118000
age.s[78]	1.0000145	1.0002148	76.8010557	76.4372000	76.2208107	20001.0	0.0575141	0.95	61.3466000	93.2072000
age.s[79]	1.0001673	1.0007906	24.6396038	23.6001000	20.3347227	20001.0	0.0315107	0.95	19.0003000	33.3655000
age.s[80]	1.0001209	1.0006130	34.3253165	34.3393000	35.3107544	19590.7	0.0379212	0.95	23.7181000	44.7061000
age.s[81]	1.0006292	1.0018653	24.5995290	23.5861000	20.2474488	20001.0	0.0310874	0.95	19.0005000	33.2282000
age.s[82]	1.0007547	1.0022928	71.3118095	70.9745000	69.7857147	19565.1	0.0522233	0.95	57.5358000	86.0264000
age.s[83]	1.0006617	1.0022726	30.8304745	30.7263000	30.6298113	20001.0	0.0399874	0.95	19.6018000	40.9109000
age.s[84]	0.9999487	1.0000674	28.9046515	28.5526000	28.6284705	19159.7	0.0404315	0.95	19.0469000	38.7433000
age.s[85]	1.0006043	1.0022205	53.6903486	53.4279000	52.2015290	19184.5	0.0411699	0.95	42.9467000	65.0551000
age.s[86]	1.0001574	1.0006894	60.5515083	60.3991000	59.9747153	20001.0	0.0434643	0.95	48.9656000	73.1853000
age.s[87]	1.0001573	1.0009233	51.1725671	50.9507000	50.0686332	19210.1	0.0393521	0.95	40.9300000	62.0646000
age.s[88]	0.9998522	0.9998545	28.8606762	28.5652000	29.1538277	20001.0	0.0393120	0.95	19.0108000	38.6150000
age.s[89]	1.0000584	1.0002535	53.7184689	53.5014000	53.5167100	19291.9	0.0413593	0.95	42.6786000	64.9799000
age.s[90]	1.0003840	1.0006999	49.5952197	49.3850000	49.5027325	19293.1	0.0387117	0.95	39.2102000	60.1331000
age.s[91]	1.0009617	1.0033189	59.4065675	59.2111000	58.9857676	18750.6	0.0456577	0.95	47.8434000	72.3028000
age.s[92]	0.9999074	0.9999133	56.3051863	56.1050000	55.4363192	20001.0	0.0419168	0.95	44.8133000	67.8514000
age.s[93]	1.0004882	1.0016303	57.8956580	57.6982000	57.6170583	19594.1	0.0429425	0.95	46.3917000	69.8710000
age.s[94]	1.0001634	1.0003686	57.2242714	57.0109000	56.3533609	20001.0	0.0432046	0.95	45.3085000	69.1953000
age.s[95]	0.9999553	1.0001903	63.1298491	62.9665000	62.8998791	19439.5	0.0464136	0.95	50.6779000	75.9112000
age.s[96]	1.0004653	1.0007529	24.6596610	23.6116000	20.2899662	20001.0	0.0315606	0.95	19.0004000	33.3896000
age.s[97]	1.0004732	1.0020543	26.3076781	25.4932000	22.2400306	20001.0	0.0357831	0.95	19.0012000	35.7921000
age.s[98]	1.0002491	1.0009533	24.6187026	23.6232000	20.1966114	20001.0	0.0312833	0.95	19.0012000	33.2482000
age.s[99]	1.0003071	1.0011769	24.6681136	23.6403000	20.2734947	20038.7	0.0316235	0.95	19.0002000	33.4873000

age.s[100]	1.0000341	1.0001116	72.2692734	71.8716000	70.9042010	20001.0	0.0530837	0.95	57.9311000	87.1427000
age.s[101]	1.0005257	1.0022582	62.9455673	62.7987000	63.1104336	19443.0	0.0469417	0.95	49.7228000	75.2282000
age.s[102]	1.0002263	1.0008419	85.5290184	85.8675000	85.3961603	20001.0	0.0568123	0.95	71.5819000	99.9962000
age.s[103]	0.9998977	0.9999640	67.2648851	66.9920000	66.1094007	18496.6	0.0506084	0.95	54.2320000	81.0247000
age.s[104]	1.0000312	1.0001010	42.9904050	42.8620000	42.5613403	18375.5	0.0359820	0.95	33.4918000	52.6622000
age.s[105]	1.0000689	1.0005256	76.7834556	76.5000000	76.5958133	20001.0	0.0574821	0.95	61.0130000	92.6127000
age.s[106]	1.0005536	1.0020166	55.6996580	55.4632000	54.0317027	18485.4	0.0433219	0.95	44.5950000	67.4611000
age.s[107]	1.0002680	1.0009494	56.2867363	56.0511000	55.1319445	19781.5	0.0421057	0.95	45.1492000	68.2672000
age.s[108]	1.0002857	1.0009063	54.7817921	54.5293000	53.8451499	19516.8	0.0417594	0.95	43.8948000	66.6400000
age.s[109]	0.9999709	1.0002496	85.4585703	85.9163000	87.9324658	20001.0	0.0564300	0.95	71.6971000	99.9906000
age.s[110]	1.0002304	1.0011625	81.6404563	81.4943000	81.0817234	20001.0	0.0588204	0.95	66.6738000	98.3301000
age.s[111]	0.9999302	1.0001222	24.6951606	23.7058000	20.1833494	20001.0	0.0312698	0.95	19.0004000	33.3106000
age.s[112]	1.0006847	1.0020761	28.8071529	28.3847000	27.8923047	20001.0	0.0394326	0.95	19.0024000	38.6784000
age.s[113]	1.0001171	1.0004468	28.8704625	28.4962000	28.0514553	19155.1	0.0402981	0.95	19.0006000	38.6228000
age.s[114]	1.0000294	1.0004073	55.0662714	54.8133000	54.2858554	19293.0	0.0416815	0.95	44.2459000	66.7685000
age.s[115]	1.0000097	1.0004170	45.6200893	45.4048000	45.2181744	18075.5	0.0368506	0.95	36.0418000	55.3659000
age.s[116]	1.0000327	1.0001732	64.7275974	64.5567000	64.9629069	20390.0	0.0454762	0.95	52.0737000	77.3223000
age.s[117]	1.0007143	1.0027539	24.7028426	23.6869000	20.1528836	20001.0	0.0316798	0.95	19.0001000	33.5117000
age.s[118]	1.0004711	1.0018913	26.3943914	25.5519000	23.5440624	20001.0	0.0359261	0.95	19.0001000	35.8874000
age.s[119]	0.9999165	0.9999949	66.0101667	65.8413000	65.3463982	20001.0	0.0474487	0.95	53.1071000	79.3160000
age.s[120]	0.9999175	1.0000346	71.8961665	71.5371000	71.0332080	19595.7	0.0526323	0.95	58.0684000	86.9148000
age.s[121]	1.0000836	1.0003347	75.1626545	74.8340000	73.8024475	19678.3	0.0559822	0.95	60.0681000	90.8015000
age.s[122]	1.0003976	1.0015828	69.2374643	68.9174000	68.7136636	20001.0	0.0500192	0.95	55.2053000	82.9045000
age.s[123]	1.0002617	1.0005359	37.5448927	37.4304000	36.5067409	19124.3	0.0354530	0.95	28.2494000	47.3440000
age.s[124]	1.0002236	1.0005770	28.8683060	28.5150000	28.0560228	20001.0	0.0393063	0.95	19.0051000	38.5043000
age.s[125]	1.0002320	1.0010332	45.8565774	45.5778000	44.8983052	18645.0	0.0361628	0.95	36.3340000	55.4661000
age.s[126]	1.0002025	1.0007109	54.8019644	54.6198000	54.2583457	19431.2	0.0416562	0.95	43.5887000	66.0511000
age.s[127]	1.0000606	1.0005794	24.6982414	23.6849000	20.1674795	19400.5	0.0327195	0.95	19.0001000	33.6628000
age.s[128]	1.0001674	1.0005144	47.9790735	47.7451000	46.3698374	20001.0	0.0357766	0.95	38.6631000	58.4189000
age.s[129]	1.0000554	1.0005683	58.5191591	58.3077000	57.5601137	20001.0	0.0428577	0.95	47.1641000	70.7015000
age.s[130]	1.0005861	1.0022355	63.2553511	63.0585000	62.5981960	19147.5	0.0452613	0.95	51.1618000	75.5291000
age.s[131]	1.0001532	1.0009310	28.8780211	28.5031000	27.7469201	20001.0	0.0394360	0.95	19.0019000	38.6350000
age.s[132]	1.0008045	1.0028751	48.3148181	48.0560000	47.5042978	18579.1	0.0376086	0.95	38.7365000	58.6775000
age.s[133]	1.0003044	1.0015182	59.4959398	59.2850000	59.1924580	20001.0	0.0440736	0.95	47.9650000	72.2885000
age.s[134]	1.0004616	1.0020199	30.8630563	30.7506000	32.3068145	18026.6	0.0423627	0.95	19.6861000	41.0000000
age.s[135]	1.0006336	1.0018883	47.0691507	46.8411000	46.2431745	19337.2	0.0363450	0.95	37.2967000	57.0060000
age.s[136]	0.9999439	1.0001533	41.1748008	41.0266000	40.5380031	19607.0	0.0340592	0.95	32.0310000	50.5789000
age.s[137]	1.0010260	1.0037946	56.5540641	56.2854000	55.8700271	19298.9	0.0432244	0.95	45.1199000	68.4745000
age.s[138]	1.0000997	1.0001797	24.6715507	23.6698000	20.1498292	20001.0	0.0314040	0.95	19.0001000	33.2759000

age.s[139]	1.0004659	1.0017811	65.2057201	64.9641000	63.6147107	19502.4	0.0464321	0.95	53.3105000	78.6419000
age.s[140]	0.9999812	1.0002190	30.8809523	30.8183000	31.7927692	20001.0	0.0399883	0.95	19.6264000	40.9161000
age.s[141]	1.0004056	1.0015955	26.3936098	25.5639000	22.2958040	20001.0	0.0358343	0.95	19.0008000	35.9599000
age.s[142]	1.0003368	1.0013580	47.6565511	47.4214000	47.1828657	18672.4	0.0374830	0.95	38.2000000	57.9781000
age.s[143]	0.9999854	1.0002599	24.6375192	23.6203000	20.2219938	19522.7	0.0318395	0.95	19.0003000	33.3692000
age.s[144]	1.0003070	1.0012918	51.7931539	51.6546000	51.7775737	18216.1	0.0390574	0.95	41.5185000	62.0195000
age.s[145]	1.0000848	1.0003872	55.7221590	55.4700000	54.6343520	19127.2	0.0428037	0.95	44.0830000	67.1277000
age.s[146]	1.0000152	1.0001361	30.8508766	30.6758000	30.3161897	19763.0	0.0403396	0.95	20.0349000	41.3539000
age.s[147]	1.0000408	1.0002617	54.4946082	54.2767000	53.7655500	19406.5	0.0415288	0.95	43.2442000	65.6095000
age.s[148]	0.9999194	1.0000159	35.5531371	35.5111000	34.7212757	18677.2	0.0383350	0.95	25.3163000	45.7564000
age.s[149]	1.0003682	1.0015764	60.0068338	59.7864000	59.7320903	19907.6	0.0445382	0.95	48.0392000	72.4948000
age.s[150]	0.9999775	1.0002086	64.5142657	64.2983000	63.9844802	19449.6	0.0473673	0.95	51.7097000	77.5455000
age.s[151]	1.0000245	1.0004792	35.6007507	35.5407000	34.6330245	19195.7	0.0372672	0.95	25.3883000	45.5950000
age.s[152]	1.0004278	1.0018180	34.2599861	34.2089000	33.3750197	19268.2	0.0384427	0.95	23.2317000	44.1731000
age.s[153]	1.0004300	1.0013354	58.2506737	58.0538000	57.2089052	20001.0	0.0431288	0.95	46.0606000	69.8219000
age.s[154]	1.0003487	1.0009947	52.5519188	52.4031000	52.5810551	19134.3	0.0400675	0.95	41.6366000	63.2013000
age.s[155]	1.0001286	1.0006935	26.4441227	25.6904000	22.8196674	20001.0	0.0359583	0.95	19.0011000	35.8853000
age.s[156]	0.9999701	1.0002766	67.0152722	66.7727000	66.2513378	19460.9	0.0490759	0.95	53.6447000	80.4853000
age.s[157]	0.9999188	0.9999685	63.1155549	62.9204000	62.9951796	20001.0	0.0447081	0.95	51.0141000	75.6193000
age.s[158]	1.0002528	1.0010178	69.1235080	68.8498000	68.1051784	20126.6	0.0483260	0.95	55.6456000	82.2701000
age.s[159]	1.0000524	1.0003242	45.3294654	45.1834000	46.0206520	19288.3	0.0359086	0.95	35.8732000	55.2498000
age.s[160]	0.9999900	1.0001384	43.7122832	43.5244000	43.3255473	19490.4	0.0343003	0.95	34.2815000	52.9514000
age.s[161]	0.9999342	1.0000097	30.8458813	30.7252000	31.4861851	18735.1	0.0413932	0.95	19.5152000	40.8501000
age.s[162]	1.0004689	1.0020387	30.8871883	30.7619000	30.9674521	20001.0	0.0404110	0.95	19.2143000	40.5583000
age.s[163]	0.9999949	1.0001419	37.6441125	37.5759000	37.3953455	18446.2	0.0368015	0.95	27.9339000	47.3747000
age.s[164]	1.0006864	1.0022513	26.3316711	25.5492000	23.1047463	20462.7	0.0349812	0.95	19.0009000	35.5658000
age.s[165]	1.0003215	1.0015854	40.1936329	40.0545000	39.8122777	19002.0	0.0348409	0.95	31.1930000	50.1028000
age.s[166]	1.0003683	1.0014931	24.6113918	23.6010000	20.1557239	20001.0	0.0311161	0.95	19.0010000	33.2257000
age.s[167]	1.0000998	1.0004532	81.8129578	81.8145000	82.4206536	20001.0	0.0597396	0.95	66.7025000	98.4632000
age.s[168]	0.9999890	1.0003526	55.9304109	55.7316000	54.5491910	19570.2	0.0419733	0.95	44.5127000	67.3131000
age.s[169]	1.0002193	1.0010097	50.6902935	50.5460000	51.0150375	19013.7	0.0397205	0.95	40.3546000	61.6670000
age.s[170]	0.9999005	0.9999430	37.1183539	37.1033000	37.5351994	18250.8	0.0377066	0.95	27.0625000	46.7928000
age.s[171]	1.0001058	1.0006944	30.8883773	30.7698000	30.5924149	20247.0	0.0399360	0.95	19.5588000	40.9183000
age.s[172]	1.0001283	1.0005230	28.8896423	28.4509000	27.5818402	20001.0	0.0395103	0.95	19.0147000	38.6991000
age.s[173]	1.0004624	1.0014573	61.9475662	61.7742000	61.1481833	19345.2	0.0441837	0.95	50.1846000	74.0331000
age.s[174]	0.9999879	1.0000932	53.5793061	53.3910000	53.0976197	19374.6	0.0403233	0.95	42.6141000	64.5639000
age.s[175]	1.0001867	1.0009382	36.8964955	36.8624000	36.9413521	18664.8	0.0358764	0.95	27.4327000	46.4085000
age.s[176]	1.0004079	1.0013553	67.0134048	66.7196000	66.4772860	20001.0	0.0488688	0.95	53.6912000	80.7798000
age.s[177]	1.0000280	1.0003470	52.3347766	52.0871000	51.5216313	20309.5	0.0390181	0.95	41.4532000	63.0975000

age.s[178]	1.0003908	1.0009849	57.4141287	57.1451000	56.3530685	20001.0	0.0427863	0.95	45.8453000	69.2145000
age.s[179]	1.0001417	1.0004064	24.6843530	23.6502000	20.1424073	20001.0	0.0315573	0.95	19.0009000	33.3871000
age.s[180]	0.9999128	1.0000353	34.2556803	34.2561000	35.0904303	18611.5	0.0391360	0.95	23.6070000	44.4410000
age.s[181]	0.9998682	0.9999137	70.0843305	69.7302000	69.1858464	20001.0	0.0522713	0.95	56.3385000	85.1800000
age.s[182]	0.9999462	1.0000696	61.6217656	61.4234000	60.7348238	19429.1	0.0454966	0.95	49.5517000	74.3206000
age.s[183]	1.0000214	1.0000938	81.5962086	81.4869000	80.1094472	20665.4	0.0574090	0.95	66.9897000	98.3156000
age.s[184]	0.9999678	1.0000481	28.8970521	28.5771000	29.1524654	18689.3	0.0408807	0.95	19.0219000	38.6191000
age.s[185]	0.9999938	1.0002856	83.5812589	83.6656000	81.9777689	20001.0	0.0585314	0.95	69.4413000	99.6943000
age.s[186]	0.9999487	1.0001174	56.5710257	56.3411000	55.9264768	19455.3	0.0426106	0.95	45.1454000	68.3413000
age.s[187]	1.0000408	1.0005184	59.5560585	59.3263000	58.8078321	19514.2	0.0448129	0.95	47.3729000	71.5143000
age.s[188]	1.0007343	1.0017678	73.3440935	72.8874000	71.8387859	19524.1	0.0549557	0.95	58.5239000	88.6327000
age.s[189]	1.0003500	1.0010396	76.3947309	75.9888000	75.9708183	20001.0	0.0570099	0.95	60.9049000	92.2843000
age.s[190]	1.0004272	1.0018446	30.8777806	30.7606000	30.3319428	19324.6	0.0406680	0.95	19.7108000	41.0712000
age.s[191]	1.0003437	1.0016196	52.2030361	52.0360000	51.9435637	18326.9	0.0389842	0.95	42.1739000	62.7965000
age.s[192]	1.0001971	1.0006134	59.4079079	59.2123000	58.8751858	19393.6	0.0450110	0.95	47.2834000	71.6883000
age.s[193]	1.0001742	1.0008860	35.6327607	35.5880000	35.6386567	18788.2	0.0381854	0.95	25.5216000	45.9394000
age.s[194]	1.0001154	1.0005490	64.7421269	64.5002000	64.3379526	19342.9	0.0466648	0.95	52.2843000	77.7573000
age.s[195]	0.9999467	1.0001782	72.2408332	71.8878000	71.3475897	19345.8	0.0533101	0.95	58.1948000	86.9155000
age.s[196]	1.0000119	1.0001634	46.1179068	45.9302000	45.7297158	19065.6	0.0363056	0.95	36.7803000	56.3188000
age.s[197]	1.0002239	1.0011436	57.0684088	56.8957000	56.7681532	19401.6	0.0415059	0.95	45.6293000	68.1564000
age.s[198]	1.0011189	1.0026300	24.6149387	23.5763000	20.2205937	20001.0	0.0313627	0.95	19.0018000	33.3341000
age.s[199]	1.0003392	1.0016418	52.1630857	51.8986000	50.6995213	19223.3	0.0403297	0.95	41.8222000	63.4609000
age.s[200]	0.9999210	1.0000875	24.6574394	23.6261000	20.2986786	20001.0	0.0314662	0.95	19.0003000	33.4248000

2.1.4 15 traits

Table 20: MCMC diagnostic data for CAMSAD dataset (n traits = 15).

	PSRF Point est.	PSRF Upper C.I.	Mean	Median	Mode	ESS	MCSE	HDImass	HDIlow	HDIhigh
b	1.0001915	1.0009402	0.0330737	0.0330425	0.0335476	17898.3	0.0000277	0.95	0.0259620	0.0403974
a	1.0002207	1.0009026	0.0126175	0.0123078	0.0115368	17978.8	0.0000219	0.95	0.0072969	0.0184961
M	1.0002249	1.0008691	47.9326548	48.8875000	50.8948035	18011.2	0.0559272	0.95	32.6203000	60.9423000
beta0[1]	1.0003834	1.0015776	-4.7239787	-4.7040500	-4.6849116	20001.0	0.0068934	0.95	-6.5982300	-2.7901600
beta0[2]	0.9999100	0.9999932	-15.4885810	-15.3100000	-15.0893180	17259.9	0.0198344	0.95	-20.9059000	-10.7852000
beta0[3]	1.0005520	1.0021040	-10.8966164	-10.8246000	-10.7465668	18927.1	0.0116875	0.95	-14.1224000	-7.8753000
beta0[4]	0.9999927	1.0002859	-13.1439610	-13.0398000	-12.7033740	19042.6	0.0133958	0.95	-16.8342000	-9.6666700
beta0[5]	1.0002680	1.0012843	-21.7817205	-21.6128000	-21.4803945	16154.2	0.0224999	0.95	-27.3875000	-16.2698000
beta0[6]	1.0010977	1.0037739	-11.9788056	-11.8512000	-11.4904823	18732.2	0.0148701	0.95	-16.0122000	-8.0316500
beta0[7]	0.9999738	1.0002974	-24.1434016	-23.9729000	-23.8672275	14043.2	0.0267193	0.95	-30.3640000	-18.0849000
beta0[8]	1.0012063	1.0025239	-25.5124138	-25.2987000	-25.1569990	14501.9	0.0287871	0.95	-32.5139000	-19.0487000
beta0[9]	1.0001711	1.0010302	-12.8253038	-12.7305000	-12.7013524	18500.0	0.0137366	0.95	-16.4884000	-9.2819500
beta0[10]	1.0007023	1.0023441	-25.3591019	-25.1746000	-24.8939908	14373.0	0.0272805	0.95	-31.5957000	-19.0575000
beta0[11]	1.0007911	1.0030315	-22.0328424	-21.8358000	-21.1983427	10034.2	0.0330083	0.95	-28.3929000	-15.7076000
beta0[12]	1.0014952	1.0048349	-13.9450003	-13.8248000	-13.5889980	18513.0	0.0148422	0.95	-18.0140000	-10.1562000
beta0[13]	1.0004427	1.0009633	-13.8410608	-13.6908000	-13.3619868	19154.6	0.0171158	0.95	-18.5137000	-9.3136000
beta0[14]	1.0014112	1.0029543	-29.3576194	-29.1154000	-28.1331085	12711.6	0.0343800	0.95	-37.0412000	-22.0233000
beta0[15]	1.0002012	1.0011147	-21.6811834	-21.5123000	-21.0191917	15684.1	0.0227948	0.95	-27.2618000	-16.2454000
beta[1]	1.0003358	1.0012508	1.4265901	1.4214800	1.4054995	20001.0	0.0017707	0.95	0.9319340	1.9132800
beta[2]	0.9999349	1.0000397	3.8464152	3.8061700	3.7616405	17433.1	0.0047331	0.95	2.6230500	5.0456600
beta[3]	1.0004325	1.0017883	3.4030537	3.3811500	3.3319985	19128.1	0.0032306	0.95	2.5824800	4.3159200
beta[4]	1.0000086	1.0003181	3.6130513	3.5879800	3.5127007	19234.4	0.0033966	0.95	2.7072300	4.5385700
beta[5]	1.0002104	1.0010432	5.7519033	5.7133600	5.6158865	16466.8	0.0055113	0.95	4.3789400	7.1409200
beta[6]	1.0011236	1.0038411	2.9865280	2.9587100	2.9265037	18867.9	0.0035639	0.95	2.0671300	3.9824600
beta[7]	0.9999519	1.0002181	6.4339730	6.3951100	6.4168461	13818.0	0.0067625	0.95	4.9115000	7.9865100
beta[8]	1.0011764	1.0024044	6.7001976	6.6496900	6.5841592	14758.8	0.0070644	0.95	5.0498300	8.3800200
beta[9]	1.0001670	1.0010060	3.3867871	3.3631000	3.3473574	18696.3	0.0033735	0.95	2.4984000	4.2845100
beta[10]	1.0006903	1.0022668	6.7642446	6.7172900	6.6547219	14714.7	0.0067589	0.95	5.2025200	8.3421100
beta[11]	1.0008363	1.0032152	6.9659820	6.8998200	6.7992357	10079.9	0.0098792	0.95	5.1386400	8.9330800
beta[12]	1.0016946	1.0053802	3.6825657	3.6568800	3.5783412	18469.1	0.0036750	0.95	2.7441500	4.6910700
beta[13]	1.0003798	1.0008266	3.4308059	3.3975300	3.2642714	19304.5	0.0040931	0.95	2.3278200	4.5415300
beta[14]	1.0012389	1.0026284	7.7791220	7.7225400	7.4547361	12950.9	0.0085645	0.95	5.9616400	9.7410200
beta[15]	1.0001443	1.0008871	5.7412009	5.7041600	5.7106797	16036.9	0.0055857	0.95	4.3515600	7.0771300
thresh[1,1]	NaN	NaN	0.5000000	0.5000000	0.4996355	0.0	NaN	0.95	0.5000000	0.5000000
thresh[2,1]	NaN	NaN	0.5000000	0.5000000	0.4996355	0.0	NaN	0.95	0.5000000	0.5000000

thresh[3,1]	NaN	NaN	0.5000000	0.5000000	0.4996355	0.0	NaN	0.95	0.5000000	0.5000000
thresh[4,1]	NaN	NaN	0.5000000	0.5000000	0.4996355	0.0	NaN	0.95	0.5000000	0.5000000
thresh[5,1]	NaN	NaN	0.5000000	0.5000000	0.4996355	0.0	NaN	0.95	0.5000000	0.5000000
thresh[6,1]	NaN	NaN	0.5000000	0.5000000	0.4996355	0.0	NaN	0.95	0.5000000	0.5000000
thresh[7,1]	NaN	NaN	0.5000000	0.5000000	0.4996355	0.0	NaN	0.95	0.5000000	0.5000000
thresh[8,1]	NaN	NaN	0.5000000	0.5000000	0.4996355	0.0	NaN	0.95	0.5000000	0.5000000
thresh[9,1]	NaN	NaN	0.5000000	0.5000000	0.4996355	0.0	NaN	0.95	0.5000000	0.5000000
thresh[10,1]	NaN	NaN	0.5000000	0.5000000	0.4996355	0.0	NaN	0.95	0.5000000	0.5000000
thresh[11,1]	NaN	NaN	0.5000000	0.5000000	0.4996355	0.0	NaN	0.95	0.5000000	0.5000000
thresh[12,1]	NaN	NaN	0.5000000	0.5000000	0.4996355	0.0	NaN	0.95	0.5000000	0.5000000
thresh[13,1]	NaN	NaN	0.5000000	0.5000000	0.4996355	0.0	NaN	0.95	0.5000000	0.5000000
thresh[14,1]	NaN	NaN	0.5000000	0.5000000	0.4996355	0.0	NaN	0.95	0.5000000	0.5000000
thresh[15,1]	NaN	NaN	0.5000000	0.5000000	0.4996355	0.0	NaN	0.95	0.5000000	0.5000000
thresh[3,2]	1.0002178	1.0010341	4.0807117	4.0659600	4.0858412	19483.9	0.0025812	0.95	3.3656400	4.7729400
thresh[5,2]	1.0000360	1.0003387	2.4450921	2.4355900	2.4259798	19438.2	0.0017364	0.95	1.9824000	2.9267400
thresh[7,2]	0.9999566	1.0000584	2.3362473	2.3221600	2.2938696	18383.2	0.0019670	0.95	1.8253200	2.8560900
thresh[8,2]	1.0001406	1.0007115	3.2626643	3.2456500	3.1820026	17795.7	0.0024869	0.95	2.6268800	3.9161200
thresh[10,2]	1.0002202	1.0005795	3.2631736	3.2428300	3.1950071	18203.9	0.0024842	0.95	2.6350900	3.9349000
thresh[11,2]	1.0008869	1.0035560	5.6307331	5.5745300	5.5153653	10812.3	0.0077030	0.95	4.1086100	7.1696400
thresh[14,2]	1.0000051	1.0001570	2.7072036	2.6900200	2.6270664	17396.3	0.0024268	0.95	2.1219500	3.3672300
thresh[15,2]	1.0000408	1.0001446	2.5175740	2.5035500	2.4940115	19463.2	0.0018262	0.95	2.0366000	3.0216700
thresh_age[1,1]	1.0008258	1.0033506	38.7342303	38.7765000	38.8612630	18815.3	0.0256429	0.95	31.7679000	45.5614000
thresh_age[2,1]	0.9999433	1.0000250	63.8890116	63.9086000	64.4418075	19102.3	0.0244348	0.95	57.3462000	70.5485000
thresh_age[3,1]	1.0004544	1.0016579	28.4098712	28.3665000	27.8783188	17321.1	0.0154658	0.95	24.3661000	32.3268000
thresh_age[4,1]	1.0000103	1.0003499	43.6013942	43.5509000	43.1957313	17709.8	0.0201167	0.95	38.3940000	48.8229000
thresh_age[5,1]	1.0004447	1.0020180	48.0649749	48.0643000	48.0024373	16820.0	0.0210023	0.95	42.8057000	53.3684000
thresh_age[6,1]	1.0003008	1.0011011	65.3525403	65.3055000	65.5732810	19443.6	0.0273389	0.95	57.7182000	72.7764000
thresh_age[7,1]	1.0001222	1.0005469	46.0332554	46.0134000	46.1612692	16869.3	0.0205153	0.95	40.9151000	51.3103000
thresh_age[8,1]	1.0002209	1.0011240	48.4803672	48.4838000	48.6040240	16747.7	0.0211006	0.95	43.1865000	53.8350000
thresh_age[9,1]	1.0000146	1.0002491	51.0767598	51.0800000	51.2834228	18246.9	0.0221017	0.95	45.2712000	56.9173000
thresh_age[10,1]	1.0002293	1.0010717	45.6949386	45.6682000	45.6029670	16078.3	0.0208498	0.95	40.6225000	50.9453000
thresh_age[11,1]	1.0002928	1.0006840	25.3916346	25.3048000	24.9805872	16774.3	0.0115990	0.95	22.5450000	28.3626000
thresh_age[12,1]	1.0001561	1.0007432	50.4688591	50.4830000	50.5934646	17269.8	0.0221871	0.95	44.7829000	56.2372000
thresh_age[13,1]	1.0002296	1.0012256	65.4513654	65.4174000	65.4481056	19517.7	0.0260223	0.95	58.3048000	72.4630000
thresh_age[14,1]	1.0000744	1.0006543	46.4103407	46.4234000	46.5524855	16236.2	0.0207814	0.95	41.1825000	51.4721000
thresh_age[15,1]	1.0004326	1.0018224	47.5729606	47.5790000	47.7169701	16760.0	0.0210552	0.95	42.3699000	53.0150000
thresh_age[3,2]	1.0000015	1.0001317	81.8765092	81.6889000	81.8678607	19531.3	0.0367555	0.95	72.2270000	92.2901000
thresh_age[5,2]	0.9998948	0.9999332	67.5195541	67.5490000	67.9620741	18995.2	0.0237014	0.95	61.1835000	73.9798000
thresh_age[7,2]	0.9999034	0.9999236	61.2950161	61.3091000	61.1232247	18588.4	0.0220615	0.95	55.3410000	67.0709000

thresh_age[8,2]	1.0001862	1.0009837	73.3634370	73.4283000	73.7372941	20001.0	0.0240300	0.95	66.6847000	79.9291000
thresh_age[10,2]	1.0000252	1.0002810	68.8459231	68.9072000	69.1574955	19038.7	0.0234118	0.95	62.5824000	75.1894000
thresh_age[11,2]	1.0000247	1.0004684	53.0267993	53.0529000	53.1888272	16589.3	0.0216725	0.95	47.5110000	58.4654000
thresh_age[14,2]	1.0000182	1.0004142	61.6849725	61.7515000	61.8845749	18181.7	0.0219396	0.95	55.9806000	67.5290000
thresh_age[15,2]	0.9999295	1.0001359	67.7111867	67.7570000	67.8036712	19320.2	0.0234167	0.95	61.0894000	73.8291000
age.s[1]	1.0000058	1.0002960	22.5625020	21.9476000	19.8557187	19598.3	0.0199657	0.95	19.0005000	27.9888000
age.s[2]	1.0009966	1.0038676	45.2727186	45.1823000	45.0807006	17342.9	0.0296529	0.95	37.7901000	52.9625000
age.s[3]	1.0001781	1.0010370	31.8399779	31.7057000	31.8137683	19434.5	0.0340437	0.95	22.4884000	40.8569000
age.s[4]	0.9999168	0.9999436	63.2743740	63.1671000	62.3001372	21071.4	0.0340198	0.95	53.8429000	73.1225000
age.s[5]	1.0004736	1.0017780	85.7377955	85.9117000	86.5164699	20001.0	0.0506773	0.95	73.1944000	99.4742000
age.s[6]	1.0000324	1.0001247	69.4331313	69.2811000	69.6241232	19325.4	0.0410181	0.95	58.3631000	80.7336000
age.s[7]	0.9999794	1.0002079	31.8444853	31.7105000	30.4411416	19000.7	0.0341584	0.95	23.0280000	40.9244000
age.s[8]	0.9998690	0.9998964	63.4579540	63.3472000	63.1397970	19801.9	0.0348992	0.95	54.1415000	73.2608000
age.s[9]	1.0003091	1.0008961	60.6084223	60.4580000	60.3766691	19346.3	0.0344233	0.95	51.6275000	70.2121000
age.s[10]	0.9999676	1.0000486	31.7929186	31.5995000	30.2960742	18402.2	0.0349400	0.95	22.8018000	40.9905000
age.s[11]	0.9999228	1.0000375	31.8229598	31.6764000	31.9276935	19545.6	0.0341923	0.95	22.9274000	41.3420000
age.s[12]	1.0005940	1.0025607	51.3484799	51.2748000	51.1099822	18766.8	0.0300054	0.95	43.3896000	59.4239000
age.s[13]	1.0007692	1.0020597	36.8971045	36.8945000	36.9599690	18413.0	0.0325548	0.95	28.4225000	45.6235000
age.s[14]	0.9998736	0.9999177	29.8146700	29.5567000	28.4449204	18185.4	0.0343002	0.95	21.0240000	38.7596000
age.s[15]	1.0000545	1.0002504	74.2234306	73.9385000	73.1472130	20001.0	0.0452514	0.95	61.7795000	86.7794000
age.s[16]	0.9998998	0.9999078	71.6415835	71.4742000	71.1868426	18562.6	0.0410179	0.95	60.6208000	82.4088000
age.s[17]	0.9999794	1.0000577	82.2798601	82.0416000	80.1435901	19312.6	0.0522977	0.95	68.9394000	96.8895000
age.s[18]	1.0006309	1.0026833	33.1740016	33.1310000	33.6850477	19331.5	0.0335257	0.95	24.2826000	42.1760000
age.s[19]	1.0002528	1.0010600	88.6241311	89.1500000	91.2997545	20867.9	0.0463518	0.95	76.7126000	99.9932000
age.s[20]	1.0002333	1.0008612	67.4269225	67.2826000	67.2800189	18456.2	0.0402657	0.95	56.9618000	78.3296000
age.s[21]	1.0015004	1.0049506	39.6894468	39.6509000	39.5678731	17775.0	0.0303910	0.95	31.9489000	47.7761000
age.s[22]	1.0000473	1.0004722	63.5558589	63.4265000	62.9471180	18493.8	0.0360429	0.95	54.2562000	73.4282000
age.s[23]	1.0001161	1.0008154	33.1929015	33.0804000	33.0892893	18305.6	0.0349427	0.95	24.1559000	42.3956000
age.s[24]	0.9999709	1.0002330	22.5695226	21.9660000	19.7874176	20001.0	0.0197378	0.95	19.0005000	27.9585000
age.s[25]	0.9999796	1.0001847	55.5557528	55.4727000	55.1059729	18855.4	0.0319994	0.95	46.9597000	64.0246000
age.s[26]	1.0002548	1.0011041	88.6419741	89.1501000	91.6140013	20001.0	0.0477538	0.95	76.7389000	99.9959000
age.s[27]	1.0001945	1.0011008	72.2850763	72.1085000	71.9763430	19603.3	0.0423839	0.95	61.0670000	84.0965000
age.s[28]	1.0001549	1.0004059	38.2666500	38.2232000	38.1356642	18320.3	0.0302640	0.95	30.1522000	46.1442000
age.s[29]	1.0003189	1.0011633	54.0700124	53.9370000	53.8570417	19282.7	0.0308384	0.95	45.9675000	62.4915000
age.s[30]	1.0007762	1.0031464	33.2199035	33.1322000	32.5977427	18623.5	0.0348677	0.95	23.9296000	42.2710000
age.s[31]	1.0000141	1.0003207	63.9311885	63.8434000	63.9998422	19332.7	0.0360259	0.95	53.8994000	73.6562000
age.s[32]	1.0002171	1.0011377	69.9071774	69.6984000	69.2821036	20001.0	0.0405918	0.95	59.0677000	81.3833000
age.s[33]	1.0000252	1.0004350	88.6292313	89.1870000	89.9065088	20001.0	0.0475842	0.95	76.7564000	99.9937000
age.s[34]	1.0005324	1.0015961	22.5397547	21.9313000	19.8176286	20001.0	0.0195951	0.95	19.0015000	27.8791000

age.s[35]	1.0000452	1.0004016	36.6228982	36.6317000	37.2555778	17753.1	0.0324615	0.95	27.8592000	44.8515000
age.s[36]	1.0000639	1.0001166	82.0878913	81.9152000	79.6848323	20001.0	0.0503583	0.95	68.8986000	96.5171000
age.s[37]	1.0006989	1.0024031	75.9813881	75.6512000	74.8732117	20001.0	0.0460582	0.95	63.7115000	89.2602000
age.s[38]	0.9999259	1.0000690	22.5954045	21.9731000	19.7687442	20001.0	0.0200304	0.95	19.0000000	28.1073000
age.s[39]	0.9999225	1.0000942	85.7466839	85.8428000	85.2910473	20001.0	0.0507626	0.95	73.7318000	99.9325000
age.s[40]	1.0000411	1.0003515	85.9506802	86.0484000	85.1178804	20001.0	0.0501828	0.95	74.0441000	99.9571000
age.s[41]	1.0001212	1.0007356	52.6217723	52.5397000	52.5752438	18799.4	0.0298747	0.95	44.9997000	61.1039000
age.s[42]	1.0006203	1.0013448	22.5987057	21.9848000	19.7760914	20001.0	0.0197645	0.95	19.0021000	27.9953000
age.s[43]	0.9999910	1.0000631	33.2068368	33.1748000	33.4747937	19514.5	0.0340137	0.95	23.8477000	42.2945000
age.s[44]	1.0006402	1.0024089	51.8125952	51.7011000	51.2501656	18676.2	0.0306162	0.95	43.5070000	59.9381000
age.s[45]	1.0003447	1.0015975	76.0346737	75.7180000	75.0608495	19182.7	0.0475798	0.95	63.4949000	89.2545000
age.s[46]	1.0002911	1.0012017	22.5504102	21.9303000	20.0812719	20001.0	0.0196635	0.95	19.0001000	27.9809000
age.s[47]	1.0012360	1.0045672	31.8547223	31.6987000	31.0454062	19310.8	0.0345645	0.95	22.7354000	41.3745000
age.s[48]	1.0004555	1.0016800	36.6134704	36.5812000	36.1384602	18504.2	0.0324604	0.95	28.2964000	45.3886000
age.s[49]	0.9998798	0.9999581	29.7825418	29.5493000	29.4017654	18952.9	0.0335214	0.95	21.1046000	38.7909000
age.s[50]	1.0008966	1.0020779	22.5789685	21.9625000	19.7962948	20001.0	0.0198460	0.95	19.0004000	28.0019000
age.s[51]	0.9998840	0.9999653	73.5946867	73.3478000	73.4031912	20001.0	0.0441337	0.95	61.4575000	85.7804000
age.s[52]	1.0004177	1.0016271	54.5034984	54.4080000	54.2411935	18585.4	0.0316445	0.95	46.3988000	63.1589000
age.s[53]	1.0001229	1.0007982	49.8917262	49.8367000	49.7606085	18133.2	0.0299983	0.95	42.2006000	57.9943000
age.s[54]	1.0001949	1.0011142	69.8981588	69.6802000	69.0575958	20001.0	0.0395675	0.95	59.4885000	81.3683000
age.s[55]	1.0002588	1.0011285	60.7429794	60.6905000	61.0819519	18988.7	0.0344987	0.95	51.3537000	69.9222000
age.s[56]	0.9999824	1.0001522	72.1328854	71.8664000	71.1909677	20001.0	0.0424523	0.95	60.9453000	84.1119000
age.s[57]	1.0000122	1.0002682	45.8260216	45.7766000	45.2001182	18384.9	0.0295692	0.95	38.0847000	53.7408000
age.s[58]	1.0002440	1.0004915	33.1812709	33.0509000	32.5914069	18872.9	0.0342979	0.95	24.0446000	42.2734000
age.s[59]	1.0003185	1.0015472	84.2527384	84.2193000	82.6722322	19581.7	0.0523282	0.95	71.4927000	99.1255000
age.s[60]	1.0000222	1.0002493	32.5540726	32.4454000	32.1178112	19190.1	0.0327880	0.95	23.7211000	41.2848000
age.s[61]	1.0001490	1.0004731	35.4567003	35.4280000	35.3016387	18889.8	0.0327341	0.95	26.6421000	44.0774000
age.s[62]	1.0004550	1.0016738	62.2014112	62.0817000	62.1871357	18722.2	0.0358073	0.95	52.5146000	71.6887000
age.s[63]	1.0003787	1.0017091	84.7708873	84.7920000	84.0128551	20001.0	0.0506525	0.95	71.8285000	98.7662000
age.s[64]	1.0000637	1.0002470	58.6284235	58.4938000	58.4287813	19230.7	0.0330128	0.95	49.8596000	67.7211000
age.s[65]	1.0001764	1.0010362	76.7438271	76.3782000	75.2406223	20982.8	0.0457639	0.95	64.2481000	90.1851000
age.s[66]	1.0000544	1.0002293	68.6959847	68.4740000	68.1445741	20001.0	0.0387996	0.95	58.1838000	79.6430000
age.s[67]	1.0000652	1.0003891	31.8832474	31.7514000	30.5954384	19242.3	0.0342966	0.95	22.9729000	41.2820000
age.s[68]	1.0001678	1.0006228	22.5569341	21.9581000	19.7365620	20001.0	0.0196467	0.95	19.0003000	27.9469000
age.s[69]	1.0000787	1.0000904	63.5940954	63.4505000	62.9436792	20001.0	0.0354648	0.95	53.9023000	73.5251000
age.s[70]	1.0000593	1.0005353	33.2493087	33.2090000	33.6298511	19584.5	0.0341477	0.95	24.1224000	42.5434000
age.s[71]	1.0002477	1.0011767	45.8925287	45.8352000	45.9761041	17529.4	0.0291927	0.95	38.6455000	53.5892000
age.s[72]	1.0003723	1.0008050	28.4062260	28.0887000	27.7568033	19240.2	0.0322245	0.95	19.9292000	36.8249000
age.s[73]	0.9999915	1.0001955	79.5084066	79.1510000	77.7922194	21665.9	0.0483280	0.95	65.8974000	93.6867000

age.s[74]	1.0003986	1.0014018	57.8294891	57.6613000	57.1792917	18805.3	0.0332878	0.95	48.9235000	66.7477000
age.s[75]	1.0001616	1.0006556	55.3250101	55.2608000	55.6306344	18642.8	0.0319386	0.95	46.9474000	63.9515000
age.s[76]	1.0001000	1.0007672	31.8009351	31.6240000	31.5947049	20001.0	0.0336212	0.95	22.9070000	41.1167000
age.s[77]	1.0004561	1.0018390	41.5781205	41.5294000	41.7483386	17270.7	0.0299257	0.95	33.9257000	49.2944000
age.s[78]	1.0003609	1.0010110	77.5883284	77.2838000	76.4744359	20001.0	0.0479275	0.95	64.1139000	90.5737000
age.s[79]	1.0004490	1.0005965	22.5887302	22.0032000	19.7233135	20001.0	0.0198716	0.95	19.0009000	28.0302000
age.s[80]	1.0001127	1.0003965	35.4584900	35.4180000	35.0698049	18761.5	0.0329472	0.95	26.5218000	44.0209000
age.s[81]	0.9998759	0.9999446	22.5460717	21.9555000	19.8879235	20001.0	0.0195539	0.95	19.0002000	27.9348000
age.s[82]	1.0004205	1.0004354	70.6837245	70.4780000	69.4265476	19438.9	0.0411946	0.95	59.5882000	81.8532000
age.s[83]	1.0004827	1.0020971	33.1910503	33.0975000	32.3230345	19095.7	0.0341386	0.95	24.2407000	42.4319000
age.s[84]	1.0001675	1.0008087	31.8549003	31.6970000	30.5513660	19913.1	0.0336965	0.95	22.9285000	41.2034000
age.s[85]	1.0002948	1.0014374	56.9848522	56.8498000	56.4934721	19093.5	0.0325871	0.95	48.4579000	66.0117000
age.s[86]	1.0000266	1.0002791	61.0605442	60.9475000	60.6049371	20001.0	0.0334131	0.95	51.8603000	70.2472000
age.s[87]	0.9998901	0.9999901	54.2063022	54.1237000	54.2401750	18559.5	0.0311979	0.95	45.9578000	62.4483000
age.s[88]	1.0002060	1.0009045	31.8508563	31.7091000	30.9230656	19328.1	0.0340462	0.95	22.7291000	40.9215000
age.s[89]	0.9999097	0.9999557	53.1024671	53.0176000	52.9669499	18873.4	0.0308167	0.95	44.9225000	61.3984000
age.s[90]	1.0001812	1.0009358	46.1643268	46.0990000	46.3433141	18660.0	0.0287698	0.95	38.1854000	53.6359000
age.s[91]	0.9999940	1.0000454	60.5328835	60.3704000	59.6177188	18689.0	0.0351105	0.95	51.0837000	69.8435000
age.s[92]	1.0003001	1.0010146	58.5421670	58.4763000	58.5222225	19356.6	0.0332994	0.95	49.7373000	67.9110000
age.s[93]	1.0002496	1.0011280	61.8895809	61.8187000	61.6267165	20001.0	0.0342293	0.95	52.3627000	71.3502000
age.s[94]	0.9999766	1.0001642	61.3575767	61.2276000	60.4213392	19277.1	0.0347352	0.95	52.0741000	71.0454000
age.s[95]	1.0003100	1.0015423	71.6145210	71.4172000	70.1366920	19482.4	0.0423840	0.95	60.3274000	83.4079000
age.s[96]	1.0012719	1.0041817	22.6088329	22.0155000	19.9456477	20001.0	0.0197686	0.95	19.0000000	28.0337000
age.s[97]	1.0000235	1.0002989	29.8206071	29.5976000	29.2687528	19308.9	0.0332423	0.95	21.0894000	38.7302000
age.s[98]	1.0001761	1.0006883	22.5704869	21.9632000	19.7276864	20001.0	0.0197474	0.95	19.0001000	27.9535000
age.s[99]	1.0008415	1.0022133	22.5773868	21.9713000	19.8196522	20001.0	0.0197744	0.95	19.0001000	28.0040000
age.s[100]	1.0000839	1.0002590	77.3360386	77.0027000	76.4552042	20001.0	0.0477677	0.95	64.4345000	90.8854000
age.s[101]	1.0001169	1.0003161	71.3987741	71.2130000	71.0579890	19524.6	0.0422328	0.95	59.8891000	83.0428000
age.s[102]	0.9999631	1.0001556	82.6331221	82.4423000	82.1316573	20001.0	0.0503893	0.95	69.2774000	97.0121000
age.s[103]	1.0002698	1.0011404	75.0981023	74.8576000	74.1362800	20001.0	0.0450625	0.95	63.3829000	88.3322000
age.s[104]	0.9999771	0.9999850	53.5519581	53.4485000	53.0607704	18577.2	0.0305119	0.95	45.6351000	61.9796000
age.s[105]	0.9999220	0.9999984	71.8397319	71.6594000	71.4010301	20001.0	0.0407513	0.95	60.6631000	83.1655000
age.s[106]	0.9999308	0.9999520	58.2031964	58.0248000	57.5385892	18459.9	0.0335010	0.95	49.6952000	67.3699000
age.s[107]	1.0002934	1.0005220	61.5678324	61.4209000	60.9765514	19432.5	0.0347788	0.95	52.1331000	71.0732000
age.s[108]	1.0004673	1.0011515	54.9380356	54.8348000	54.7670127	18901.4	0.0318263	0.95	46.4883000	63.6266000
age.s[109]	1.0008748	1.0033524	88.5832169	89.0774000	89.1103376	20001.0	0.0477173	0.95	76.7170000	99.9954000
age.s[110]	1.0010197	1.0041340	85.8714046	85.9963000	86.6211587	20001.0	0.0504450	0.95	73.6238000	99.7709000
age.s[111]	1.0000207	1.0002506	22.5390118	21.9268000	19.7675063	19324.6	0.0199483	0.95	19.0001000	27.9042000
age.s[112]	0.9999832	1.0002205	31.8337914	31.6408000	31.0905999	18718.8	0.0347107	0.95	22.5684000	40.8666000

age.s[113]	1.0003401	1.0013639	31.8142836	31.6526000	30.5054345	20001.0	0.0335521	0.95	22.7965000	40.9725000
age.s[114]	0.9999071	1.0000451	55.9183737	55.8231000	56.1853653	18024.6	0.0329911	0.95	47.3156000	64.5652000
age.s[115]	1.0005576	1.0023479	47.5258902	47.4360000	47.3999869	18017.3	0.0289128	0.95	39.8293000	55.0415000
age.s[116]	0.9998873	0.9999435	66.9069511	66.7764000	66.9754397	20001.0	0.0376660	0.95	56.4883000	77.3330000
age.s[117]	1.0007128	1.0017229	22.5925333	21.9967000	19.8178593	20001.0	0.0198773	0.95	19.0001000	28.0948000
age.s[118]	1.0006787	1.0012410	23.4087651	22.8848000	20.2848414	19780.2	0.0222726	0.95	19.0009000	29.2731000
age.s[119]	1.0002310	1.0012204	65.8625500	65.6388000	64.3055046	20001.0	0.0372338	0.95	56.1786000	76.7886000
age.s[120]	1.0001574	1.0009744	78.6533390	78.3319000	77.2710152	19529.2	0.0489581	0.95	65.6637000	92.3392000
age.s[121]	0.9999939	1.0003707	73.0080180	72.7686000	72.7156277	20001.0	0.0427545	0.95	61.5133000	85.1828000
age.s[122]	1.0001591	1.0005418	73.7049368	73.5144000	73.5274660	20001.0	0.0436687	0.95	61.5937000	85.5769000
age.s[123]	1.0002483	1.0010426	38.6541406	38.6608000	38.6690162	18521.6	0.0305131	0.95	30.4296000	46.6739000
age.s[124]	1.0001644	1.0002930	31.8426410	31.6373000	31.0404015	19056.0	0.0346372	0.95	22.7619000	41.1136000
age.s[125]	1.0001240	1.0008427	47.9019530	47.8426000	47.9885516	18779.9	0.0289056	0.95	40.3063000	55.7659000
age.s[126]	1.0003925	1.0017625	57.6273091	57.4965000	57.5675818	19361.1	0.0329832	0.95	48.8988000	66.7900000
age.s[127]	1.0010026	1.0032590	22.6025284	22.0032000	19.8204466	18813.9	0.0203773	0.95	19.0000000	28.0354000
age.s[128]	1.0001086	1.0005021	53.6277227	53.5547000	53.9675142	19078.2	0.0302364	0.95	45.4521000	61.7369000
age.s[129]	0.9999140	1.0000230	65.6926599	65.4919000	64.6654090	20001.0	0.0370867	0.95	55.6511000	76.1453000
age.s[130]	1.0005309	1.0019676	65.7749686	65.6291000	64.8937561	19468.1	0.0365905	0.95	55.8300000	75.7783000
age.s[131]	1.0000440	1.0003354	31.8367067	31.7258000	31.6635053	19030.4	0.0343656	0.95	22.7845000	41.0510000
age.s[132]	1.0001286	1.0008670	49.8634268	49.7437000	48.9036350	17987.7	0.0302369	0.95	42.0905000	57.9879000
age.s[133]	1.0009006	1.0035956	65.4128835	65.2346000	64.7824348	20220.6	0.0361731	0.95	55.4842000	75.6096000
age.s[134]	1.0004086	1.0016964	36.8284696	36.8066000	36.6290361	18392.2	0.0322684	0.95	28.0532000	45.0243000
age.s[135]	1.0001130	1.0006465	49.1577378	49.0876000	49.2196302	19081.2	0.0288657	0.95	41.4441000	56.9980000
age.s[136]	1.0002630	1.0013475	42.9316464	42.8456000	42.4044586	18679.5	0.0285960	0.95	35.6189000	50.9557000
age.s[137]	1.0001572	1.0007827	57.4429985	57.3192000	56.8217293	18707.1	0.0332409	0.95	49.0757000	66.8032000
age.s[138]	1.0000112	1.0000675	22.5853709	21.9673000	19.8034254	20001.0	0.0198581	0.95	19.0000000	28.0423000
age.s[139]	1.0001289	1.0005117	67.5277252	67.3683000	67.1128822	20001.0	0.0376376	0.95	57.2639000	78.0151000
age.s[140]	1.0006422	1.0027208	33.2415818	33.1415000	32.8178279	19237.2	0.0343420	0.95	23.9024000	42.2920000
age.s[141]	0.9999666	0.9999867	34.3641846	34.3493000	34.7086793	18674.6	0.0328814	0.95	25.5701000	42.9029000
age.s[142]	1.0000508	1.0002597	47.8527690	47.7889000	47.1424206	18330.9	0.0291087	0.95	40.0145000	55.3518000
age.s[143]	1.0000646	1.0005456	28.3311324	28.0182000	27.2370269	19582.6	0.0316733	0.95	20.2250000	37.1065000
age.s[144]	0.9999770	1.0000778	57.4483242	57.3736000	57.9215668	19047.7	0.0317047	0.95	49.0279000	66.0983000
age.s[145]	1.0003297	1.0007892	61.0629060	60.9373000	60.1472665	19285.0	0.0343545	0.95	52.0054000	70.5643000
age.s[146]	1.0005062	1.0019075	33.1990945	33.1362000	33.5090691	19487.6	0.0338514	0.95	24.1750000	42.4396000
age.s[147]	1.0002329	1.0008762	61.2409105	61.1096000	60.5952636	19792.8	0.0340604	0.95	52.0673000	70.6134000
age.s[148]	1.0002226	1.0005849	36.4638799	36.4351000	36.6586764	18621.0	0.0328283	0.95	27.7101000	45.1074000
age.s[149]	1.0001713	1.0006812	60.7865522	60.7027000	60.9046387	18980.3	0.0343304	0.95	51.8188000	70.3366000
age.s[150]	1.0001428	1.0001991	67.1055365	66.8982000	66.1636616	20001.0	0.0373951	0.95	57.4099000	78.0459000
age.s[151]	1.0001736	1.0010400	44.5972408	44.5575000	44.5820582	18095.1	0.0280037	0.95	37.2341000	52.0250000

age.s[152]	0.9999193	1.0000914	35.4889338	35.5001000	35.7495887	19522.8	0.0324402	0.95	26.4438000	44.0347000
age.s[153]	1.0000404	1.0005245	61.0915049	60.9738000	59.7131442	19434.9	0.0345367	0.95	52.1736000	70.9563000
age.s[154]	0.9999940	1.0001833	55.0421081	54.9555000	55.0022792	18987.4	0.0316772	0.95	46.7966000	63.7757000
age.s[155]	1.0000700	1.0002987	29.8664548	29.6224000	28.5446208	18850.0	0.0337963	0.95	20.8916000	38.6402000
age.s[156]	0.9999498	1.0001810	68.3549539	68.1287000	67.3524377	19407.7	0.0394866	0.95	57.6230000	79.2092000
age.s[157]	1.0001495	1.0007513	62.2372046	62.1528000	62.1872241	19189.8	0.0344936	0.95	52.8748000	71.6067000
age.s[158]	1.0000310	1.0000540	70.8472977	70.6457000	69.7095143	19549.5	0.0404999	0.95	60.0186000	82.1807000
age.s[159]	0.9999977	1.0000524	46.4684021	46.3936000	46.1566678	18530.8	0.0288789	0.95	38.5396000	53.9100000
age.s[160]	1.0000843	1.0002432	46.8488935	46.7699000	46.4026892	17802.6	0.0290812	0.95	39.2582000	54.5034000
age.s[161]	1.0004473	1.0014115	38.6094410	38.5848000	38.8284381	18625.9	0.0305106	0.95	30.5271000	46.8458000
age.s[162]	1.0003583	1.0016959	33.1510397	33.0743000	33.1514759	19446.5	0.0338280	0.95	24.0556000	42.2720000
age.s[163]	1.0003129	1.0011584	38.1083053	38.1187000	38.2050259	19171.2	0.0307591	0.95	29.9788000	46.5610000
age.s[164]	1.0000232	1.0000294	23.4113667	22.8351000	20.5065194	19522.8	0.0226865	0.95	19.0001000	29.4214000
age.s[165]	1.0000506	1.0002656	44.3722244	44.3188000	44.4686121	17590.0	0.0290872	0.95	36.6925000	51.6045000
age.s[166]	1.0006674	1.0018226	22.6001082	21.9989000	19.7994962	20001.0	0.0199531	0.95	19.0004000	28.0940000
age.s[167]	0.9999571	1.0001360	72.7346343	72.5083000	71.5162166	20001.0	0.0412645	0.95	61.3686000	84.0683000
age.s[168]	1.0000620	1.0005581	56.9394788	56.8150000	55.8314898	19176.6	0.0321152	0.95	48.6455000	66.0798000
age.s[169]	1.0000202	1.0004232	50.6426415	50.5779000	49.8197993	18657.2	0.0300747	0.95	42.5775000	58.6413000
age.s[170]	1.0000579	1.0006164	37.9319265	37.9312000	37.2586333	18474.7	0.0316587	0.95	29.7540000	46.5765000
age.s[171]	1.0001038	1.0006514	33.2780755	33.2188000	33.5707578	19214.9	0.0344210	0.95	23.7967000	42.3206000
age.s[172]	0.9999459	0.9999739	31.7983616	31.6301000	31.4731690	18865.0	0.0346115	0.95	22.6412000	40.8898000
age.s[173]	0.9998997	0.9999485	68.2681347	68.0688000	67.8389047	19252.5	0.0396222	0.95	57.6381000	79.0582000
age.s[174]	0.9999002	0.9999207	57.6591090	57.5380000	56.9154749	17445.4	0.0340033	0.95	48.9807000	66.5379000
age.s[175]	1.0000761	1.0003781	38.2796537	38.2794000	38.9699132	17725.8	0.0308208	0.95	30.1720000	46.2202000
age.s[176]	1.0001073	1.0002116	73.6141541	73.3316000	72.1894084	20001.0	0.0442052	0.95	61.9211000	86.2903000
age.s[177]	1.0003621	1.0017150	55.9360163	55.8267000	55.9725010	19565.1	0.0313664	0.95	47.5658000	64.5892000
age.s[178]	1.0003468	1.0013650	56.6891933	56.5229000	55.8918702	18287.0	0.0328540	0.95	47.8030000	65.2874000
age.s[179]	0.9998947	0.9999708	22.5945480	21.9429000	19.7709213	20608.9	0.0195920	0.95	19.0000000	28.0201000
age.s[180]	1.0000287	1.0000951	35.4284257	35.4297000	35.8981520	19038.9	0.0326879	0.95	26.6302000	44.1373000
age.s[181]	0.9998924	0.9999347	76.7595655	76.5218000	76.8965307	20514.7	0.0466984	0.95	64.3641000	90.3431000
age.s[182]	1.0001434	1.0008157	69.6107691	69.4579000	69.4861026	20001.0	0.0401771	0.95	58.9011000	80.9913000
age.s[183]	1.0005007	1.0019962	77.2411894	76.9277000	75.7533754	20001.0	0.0469781	0.95	64.3026000	90.4755000
age.s[184]	1.0002725	1.0009591	31.8085838	31.6296000	31.0679297	18338.3	0.0352301	0.95	22.5911000	40.9588000
age.s[185]	1.0003906	1.0018032	87.2421317	87.5754000	87.8433351	20001.0	0.0496031	0.95	75.1912000	99.9957000
age.s[186]	0.9999554	1.0000903	62.5802029	62.4623000	62.2672580	19535.3	0.0348474	0.95	53.1835000	72.2022000
age.s[187]	1.0002389	1.0007608	64.3380267	64.1845000	63.6253768	17726.0	0.0377145	0.95	54.6032000	74.1863000
age.s[188]	1.0000116	1.0002250	72.8784777	72.6227000	72.4624478	20001.0	0.0424591	0.95	61.1623000	84.7113000
age.s[189]	1.0002837	1.0003189	81.7784540	81.5391000	81.1598068	20001.0	0.0503517	0.95	68.6395000	96.3370000
age.s[190]	1.0006925	1.0010105	33.2502256	33.1779000	33.1935758	19081.8	0.0347591	0.95	24.1256000	42.7785000

age.s[191]	0.9999604	1.0001776	56.7880956	56.7001000	56.7916619	17740.7	0.0327733	0.95	48.5952000	65.7001000
age.s[192]	1.0000659	1.0005520	62.3561172	62.2088000	61.8478932	18964.1	0.0352783	0.95	53.0832000	72.1441000
age.s[193]	1.0004107	1.0014421	36.5979633	36.6224000	36.6431909	19120.7	0.0320155	0.95	27.8565000	45.0055000
age.s[194]	1.0004989	1.0010114	71.5925753	71.3973000	71.1446524	20094.3	0.0412828	0.95	60.3126000	83.0980000
age.s[195]	0.9999474	1.0001026	78.7137610	78.4795000	78.6486078	20001.0	0.0480581	0.95	65.2779000	91.9657000
age.s[196]	1.0000784	1.0003862	44.5365966	44.4879000	44.4805172	18770.9	0.0283884	0.95	37.0040000	52.1926000
age.s[197]	0.9999742	1.0002009	56.0073757	55.9001000	55.3777073	17849.5	0.0325830	0.95	47.6662000	64.7494000
age.s[198]	0.9999590	0.9999751	22.5735711	21.9597000	19.7319167	19485.0	0.0200668	0.95	19.0001000	28.0242000
age.s[199]	1.0001464	1.0003995	54.3826434	54.2726000	54.9347713	18622.5	0.0316644	0.95	45.9681000	62.7878000
age.s[200]	1.0000930	1.0002197	22.5526099	21.9537000	19.8178780	20483.8	0.0193989	0.95	19.0006000	27.9610000

2.1.5 25 traits

Table 21: MCMC diagnostic data for CAMSAD dataset (n traits = 25).

	PSRF Point est.	PSRF Upper C.I.	Mean	Median	Mode	ESS	MCSE	HDImass	HDIlow	HDIhigh
b	1.0002299	1.0008256	0.0329531	0.0328901	0.0321188	19178.9	0.0000238	0.95	0.0264720	0.0393057
a	1.0003260	1.0010093	0.0126196	0.0124022	0.0116105	19263.7	0.0000189	0.95	0.0077094	0.0177595
M	1.0003735	1.0010635	47.9648642	48.6532000	50.7034167	19288.0	0.0479334	0.95	34.6496000	59.9739000
beta0[1]	1.0000289	1.0003264	-4.6238942	-4.6109800	-4.5701180	20001.0	0.0066879	0.95	-6.5644600	-2.8420300
beta0[2]	1.0003830	1.0014402	-15.5987623	-15.4671000	-14.7510207	18865.8	0.0183956	0.95	-20.4954000	-10.7253000
beta0[3]	1.0004537	1.0015542	-10.8914857	-10.8391000	-10.8698791	19214.6	0.0111970	0.95	-13.9399000	-7.9186600
beta0[4]	1.0004997	1.0012746	-12.9223600	-12.8370000	-12.6722205	20001.0	0.0122835	0.95	-16.4611000	-9.6560100
beta0[5]	1.0006454	1.0020676	-21.7723441	-21.6336000	-21.3515905	17414.1	0.0205858	0.95	-27.2366000	-16.6540000
beta0[6]	1.0001245	1.0006764	-12.3061878	-12.1870000	-11.7972555	20001.0	0.0143524	0.95	-16.5292000	-8.6702300
beta0[7]	0.9999007	0.9999648	-22.6801474	-22.5408000	-22.2082589	17329.6	0.0212381	0.95	-28.1993000	-17.3439000
beta0[8]	1.0000913	1.0005643	-22.5728013	-22.4112000	-21.8544978	17658.9	0.0218052	0.95	-28.5269000	-17.2350000
beta0[9]	1.0001312	1.0005024	-12.8652737	-12.7775000	-12.6911729	18587.0	0.0132534	0.95	-16.5838000	-9.5210500
beta0[10]	1.0010033	1.0031587	-22.1047250	-21.9644000	-21.8141072	16352.3	0.0209335	0.95	-27.3743000	-16.8994000
beta0[11]	0.9999523	1.0002106	-22.9850141	-22.7747000	-22.4826424	11858.3	0.0300335	0.95	-29.6093000	-16.9456000
beta0[12]	1.0004939	1.0020915	-13.6391461	-13.5452000	-13.4424816	19204.7	0.0136298	0.95	-17.3047000	-9.9769700
beta0[13]	0.9999848	1.0003236	-14.8573391	-14.7202000	-14.1959982	19029.3	0.0177021	0.95	-19.6828000	-10.2174000
beta0[14]	1.0003697	1.0016618	-25.7505473	-25.5922000	-25.3870476	14934.1	0.0261173	0.95	-31.9599000	-19.5808000
beta0[15]	1.0006001	1.0019340	-23.0199212	-22.8623000	-22.8556879	16163.1	0.0227555	0.95	-28.8941000	-17.6818000
beta0[16]	1.0003950	1.0009800	-18.6364067	-18.4213000	-17.7643762	11356.3	0.0273241	0.95	-24.3255000	-13.0727000
beta0[17]	1.0001398	1.0005484	-24.8047560	-24.6295000	-24.3735144	15688.1	0.0249566	0.95	-31.0219000	-18.8411000
beta0[18]	1.0001683	1.0008405	-4.2717808	-4.2511900	-4.0632685	19568.3	0.0068955	0.95	-6.2198400	-2.4456100
beta0[19]	1.0005183	1.0020387	-4.8672855	-4.8475700	-4.8284668	19603.7	0.0070124	0.95	-6.8625700	-3.0246900
beta0[20]	0.9999737	1.0002345	-19.6428629	-19.5170000	-19.0800325	19484.5	0.0173624	0.95	-24.4430000	-15.0486000
beta0[21]	1.0002504	1.0013216	-11.4087012	-11.2509000	-11.0943573	19247.3	0.0166710	0.95	-16.2644000	-7.2244000
beta0[22]	0.9999739	1.0002972	-13.5402269	-13.4129000	-13.0744288	19171.0	0.0146841	0.95	-17.4927000	-9.6908300
beta0[23]	0.9999429	1.0000233	-4.7795675	-4.7631300	-4.7978141	20001.0	0.0068717	0.95	-6.7143900	-2.9317900
beta0[24]	1.0003234	1.0013411	-10.7198326	-10.6462000	-10.5310127	19560.0	0.0118551	0.95	-14.0823000	-7.5632500
beta0[25]	1.0000156	1.0003453	-17.6714885	-17.5114000	-17.2766499	18315.8	0.0185557	0.95	-22.6956000	-12.9321000
beta[1]	0.9999738	1.0002005	1.4012081	1.3968900	1.3585140	20001.0	0.0017259	0.95	0.9341130	1.8944200
beta[2]	1.0003288	1.0012585	3.8709046	3.8399200	3.6870439	18979.5	0.0044122	0.95	2.7042200	5.0547800
beta[3]	1.0003811	1.0013848	3.4100807	3.3948100	3.3763053	19392.0	0.0031307	0.95	2.5766700	4.2713500
beta[4]	1.0003563	1.0009853	3.5586062	3.5396300	3.5384865	20001.0	0.0031504	0.95	2.7123500	4.4529000
beta[5]	1.0005328	1.0016996	5.7572153	5.7198800	5.5919464	17579.7	0.0051184	0.95	4.5047100	7.1535000
beta[6]	1.0000813	1.0005219	3.0657695	3.0388200	2.9546417	20001.0	0.0034659	0.95	2.1347900	4.0300700
beta[7]	0.9998765	0.9999001	6.0619203	6.0299800	6.0472593	17529.9	0.0053481	0.95	4.7247700	7.4695600

beta[8]	1.0000311	1.0003330	5.9529681	5.9127300	5.8188712	17900.0	0.0054026	0.95	4.6107200	7.4303000
beta[9]	1.0001038	1.0005129	3.3974487	3.3776700	3.3631203	18683.4	0.0032807	0.95	2.5592500	4.3047600
beta[10]	1.0008514	1.0027523	5.9237377	5.8878300	5.8505713	16611.4	0.0052461	0.95	4.6375300	7.2741400
beta[11]	0.9999246	1.0001110	7.2817564	7.2157500	7.1344397	11808.1	0.0091308	0.95	5.4007300	9.2467700
beta[12]	1.0004744	1.0019985	3.6062525	3.5850700	3.5655150	19346.9	0.0033830	0.95	2.6904100	4.5148900
beta[13]	0.9999774	1.0003119	3.6756811	3.6431900	3.4952693	19068.4	0.0042543	0.95	2.5775700	4.8561100
beta[14]	1.0004248	1.0019024	6.8512443	6.8113800	6.7563824	15057.1	0.0065756	0.95	5.2774900	8.4110200
beta[15]	1.0005802	1.0017921	6.0858064	6.0475400	6.0406815	16336.4	0.0056597	0.95	4.7476600	7.5442800
beta[16]	1.0004236	1.0011345	5.8962101	5.8316200	5.6398863	11139.7	0.0083328	0.95	4.2306700	7.6179100
beta[17]	1.0000989	1.0003754	6.5945674	6.5533700	6.5098208	15585.6	0.0063172	0.95	5.0580700	8.1302600
beta[18]	1.0001580	1.0008156	1.1680803	1.1638200	1.1263729	19922.2	0.0017328	0.95	0.6880380	1.6427600
beta[19]	1.0003789	1.0015906	1.3309803	1.3269300	1.3150684	20001.0	0.0017623	0.95	0.8474910	1.8249400
beta[20]	0.9999551	1.0001460	5.3436150	5.3127900	5.2110848	19484.6	0.0044447	0.95	4.1531000	6.5519800
beta[21]	1.0002215	1.0012027	2.7038159	2.6665200	2.6200873	20001.0	0.0039055	0.95	1.6522600	3.8138600
beta[22]	0.9999269	1.0001173	3.4543961	3.4267400	3.3745086	19296.7	0.0035743	0.95	2.5287000	4.4354400
beta[23]	0.9999506	1.0001111	1.3687105	1.3642400	1.3620065	20001.0	0.0017565	0.95	0.8765980	1.8420300
beta[24]	1.0003089	1.0012889	2.7488773	2.7336000	2.7316315	20001.0	0.0028668	0.95	1.9849800	3.5801400
beta[25]	1.0000450	1.0004133	4.7645960	4.7232000	4.6374864	18502.0	0.0047228	0.95	3.5495200	6.0487700
thresh[1,1]	NaN	NaN	0.5000000	0.5000000	0.4996355	0.0	NaN	0.95	0.5000000	0.5000000
thresh[2,1]	NaN	NaN	0.5000000	0.5000000	0.4996355	0.0	NaN	0.95	0.5000000	0.5000000
thresh[3,1]	NaN	NaN	0.5000000	0.5000000	0.4996355	0.0	NaN	0.95	0.5000000	0.5000000
thresh[4,1]	NaN	NaN	0.5000000	0.5000000	0.4996355	0.0	NaN	0.95	0.5000000	0.5000000
thresh[5,1]	NaN	NaN	0.5000000	0.5000000	0.4996355	0.0	NaN	0.95	0.5000000	0.5000000
thresh[6,1]	NaN	NaN	0.5000000	0.5000000	0.4996355	0.0	NaN	0.95	0.5000000	0.5000000
thresh[7,1]	NaN	NaN	0.5000000	0.5000000	0.4996355	0.0	NaN	0.95	0.5000000	0.5000000
thresh[8,1]	NaN	NaN	0.5000000	0.5000000	0.4996355	0.0	NaN	0.95	0.5000000	0.5000000
thresh[9,1]	NaN	NaN	0.5000000	0.5000000	0.4996355	0.0	NaN	0.95	0.5000000	0.5000000
thresh[10,1]	NaN	NaN	0.5000000	0.5000000	0.4996355	0.0	NaN	0.95	0.5000000	0.5000000
thresh[11,1]	NaN	NaN	0.5000000	0.5000000	0.4996355	0.0	NaN	0.95	0.5000000	0.5000000
thresh[12,1]	NaN	NaN	0.5000000	0.5000000	0.4996355	0.0	NaN	0.95	0.5000000	0.5000000
thresh[13,1]	NaN	NaN	0.5000000	0.5000000	0.4996355	0.0	NaN	0.95	0.5000000	0.5000000
thresh[14,1]	NaN	NaN	0.5000000	0.5000000	0.4996355	0.0	NaN	0.95	0.5000000	0.5000000
thresh[15,1]	NaN	NaN	0.5000000	0.5000000	0.4996355	0.0	NaN	0.95	0.5000000	0.5000000
thresh[16,1]	NaN	NaN	0.5000000	0.5000000	0.4996355	0.0	NaN	0.95	0.5000000	0.5000000
thresh[17,1]	NaN	NaN	0.5000000	0.5000000	0.4996355	0.0	NaN	0.95	0.5000000	0.5000000
thresh[18,1]	NaN	NaN	0.5000000	0.5000000	0.4996355	0.0	NaN	0.95	0.5000000	0.5000000
thresh[19,1]	NaN	NaN	0.5000000	0.5000000	0.4996355	0.0	NaN	0.95	0.5000000	0.5000000
thresh[20,1]	NaN	NaN	0.5000000	0.5000000	0.4996355	0.0	NaN	0.95	0.5000000	0.5000000
thresh[21,1]	NaN	NaN	0.5000000	0.5000000	0.4996355	0.0	NaN	0.95	0.5000000	0.5000000

thresh[22,1]	NaN	NaN	0.5000000	0.5000000	0.4996355	0.0	NaN	0.95	0.5000000	0.5000000
thresh[23,1]	NaN	NaN	0.5000000	0.5000000	0.4996355	0.0	NaN	0.95	0.5000000	0.5000000
thresh[24,1]	NaN	NaN	0.5000000	0.5000000	0.4996355	0.0	NaN	0.95	0.5000000	0.5000000
thresh[25,1]	NaN	NaN	0.5000000	0.5000000	0.4996355	0.0	NaN	0.95	0.5000000	0.5000000
thresh[3,2]	1.0002970	1.0013671	4.1276554	4.1114900	4.0559678	20001.0	0.0025579	0.95	3.4478100	4.8585000
thresh[5,2]	0.9999417	1.0000774	2.4964205	2.4856100	2.4505959	20001.0	0.0017468	0.95	2.0213200	2.9831000
thresh[7,2]	1.0003997	1.0006867	2.2977921	2.2845000	2.2602229	19011.9	0.0018603	0.95	1.8175100	2.8189800
thresh[8,2]	0.9999326	1.0000271	3.0441103	3.0306600	3.0202385	19578.7	0.0020698	0.95	2.5025100	3.6251900
thresh[10,2]	1.0001284	1.0005728	2.9995384	2.9858100	2.9283215	19002.0	0.0020812	0.95	2.4555500	3.5697400
thresh[11,2]	0.9998844	0.9999688	5.9490668	5.8988900	5.8803702	12352.1	0.0073359	0.95	4.4031400	7.5634500
thresh[14,2]	1.0003849	1.0016707	2.5272492	2.5114400	2.4421768	18484.6	0.0021066	0.95	2.0044600	3.1086000
thresh[15,2]	1.0001153	1.0002897	2.6448832	2.6307100	2.5683463	19446.5	0.0019414	0.95	2.1287400	3.1777500
thresh[16,2]	1.0003302	1.0010627	6.3203749	6.2562400	6.2070338	10972.6	0.0080679	0.95	4.7299800	7.9985000
thresh[17,2]	0.9999338	1.0000951	3.1492045	3.1341000	3.1431358	18042.6	0.0024436	0.95	2.5542500	3.8265100
thresh[20,2]	0.9999859	1.0000455	2.8763544	2.8628300	2.8120254	19013.7	0.0020952	0.95	2.3537700	3.4791400
thresh_age[1,1]	1.0001074	1.0006599	38.5381092	38.5733000	38.6344888	19144.5	0.0243186	0.95	31.7458000	45.0603000
thresh_age[2,1]	1.0002064	1.0010426	64.0345599	64.0015000	64.0951074	20001.0	0.0219801	0.95	57.9849000	70.2174000
thresh_age[3,1]	1.0002501	1.0011914	28.1777687	28.1431000	28.1505005	18762.1	0.0134605	0.95	24.5894000	31.7989000
thresh_age[4,1]	1.0004894	1.0019846	43.4063132	43.3790000	43.3209684	18617.8	0.0173421	0.95	38.9568000	48.2432000
thresh_age[5,1]	1.0005518	1.0023254	47.8252665	47.7874000	47.6807536	18794.4	0.0171412	0.95	43.2868000	52.4362000
thresh_age[6,1]	1.0001325	1.0008612	65.2726271	65.1964000	64.9801581	20001.0	0.0246537	0.95	58.5616000	72.3006000
thresh_age[7,1]	1.0006079	1.0022345	45.7483612	45.7367000	45.6002943	18274.3	0.0171139	0.95	41.3468000	50.3842000
thresh_age[8,1]	1.0009783	1.0039355	48.1701329	48.1482000	47.9279159	18684.7	0.0174023	0.95	43.5233000	52.8126000
thresh_age[9,1]	1.0002443	1.0005256	51.0497848	51.0427000	51.2442460	19402.6	0.0192126	0.95	45.8507000	56.2802000
thresh_age[10,1]	1.0004344	1.0019417	45.3785287	45.3773000	45.5698249	18093.3	0.0170454	0.95	40.8248000	49.7913000
thresh_age[11,1]	1.0001936	1.0009862	25.1579854	25.0688000	24.8253803	17734.4	0.0099303	0.95	22.5942000	27.7619000
thresh_age[12,1]	1.0000672	1.0004944	50.3878103	50.3887000	50.6236043	19861.3	0.0185777	0.95	45.0976000	55.3819000
thresh_age[13,1]	1.0001641	1.0008060	65.3090687	65.2610000	65.1706692	20001.0	0.0230028	0.95	59.0182000	71.7933000
thresh_age[14,1]	1.0000294	1.0003605	46.0975828	46.0685000	45.8968235	17778.1	0.0172021	0.95	41.7119000	50.6352000
thresh_age[15,1]	1.0005441	1.0019996	47.6417306	47.6394000	47.5147845	18454.4	0.0173935	0.95	43.0817000	52.2965000
thresh_age[16,1]	1.0001562	1.0008090	25.6734767	25.6117000	25.6197990	17783.3	0.0105923	0.95	23.0501000	28.5610000
thresh_age[17,1]	1.0004221	1.0018734	46.3561269	46.3683000	46.5749050	18198.3	0.0171397	0.95	41.8879000	50.8656000
thresh_age[18,1]	1.0002860	1.0004664	60.0491246	59.3230000	58.0814958	20001.0	0.0451121	0.95	48.7189000	72.7105000
thresh_age[19,1]	1.0007251	1.0021256	56.7004228	56.3022000	55.5775208	20001.0	0.0354193	0.95	47.3565000	66.6009000
thresh_age[20,1]	1.0001676	1.0008150	43.3229099	43.2962000	43.3221809	18112.5	0.0168637	0.95	38.9551000	47.8496000
thresh_age[21,1]	1.0007971	1.0007985	82.7892399	81.8763000	81.1351988	18674.6	0.0509326	0.95	70.7940000	96.4653000
thresh_age[22,1]	1.0009121	1.0037529	58.2232353	58.2068000	58.4251907	19450.9	0.0210869	0.95	52.6057000	64.0865000
thresh_age[23,1]	1.0004964	1.0021193	47.3073584	47.1885000	46.5788250	20001.0	0.0270971	0.95	39.9161000	54.9244000
thresh_age[24,1]	0.9999886	1.0003332	59.2576595	59.1868000	59.1512273	19210.2	0.0233665	0.95	53.0049000	65.7509000

thresh_age[25,1]	1.0006070	1.0020165	45.2858925	45.2797000	45.4468048	18771.9	0.0170617	0.95	40.7913000	49.9550000
thresh_age[3,2]	1.0003582	1.0012805	82.1294538	81.8886000	81.6964597	20001.0	0.0349500	0.95	72.5862000	91.9375000
thresh_age[5,2]	1.0001806	1.0009007	67.7525703	67.7803000	67.8445357	20001.0	0.0207630	0.95	62.1078000	73.5902000
thresh_age[7,2]	1.0001894	1.0010784	61.5913454	61.5813000	61.3911391	19056.0	0.0193335	0.95	56.2858000	66.6800000
thresh_age[8,2]	1.0006400	1.0019171	74.0096699	74.0224000	74.0351913	20001.0	0.0225139	0.95	67.8823000	80.3463000
thresh_age[10,2]	1.0000024	1.0001485	69.3070421	69.3222000	69.7946373	20001.0	0.0210876	0.95	63.4093000	75.0446000
thresh_age[11,2]	1.0002633	1.0013693	53.1544875	53.1686000	53.2879687	17987.4	0.0180171	0.95	48.3326000	57.8038000
thresh_age[14,2]	1.0000202	1.0003710	62.0198816	62.0603000	62.3992206	19372.6	0.0189737	0.95	56.7523000	67.0389000
thresh_age[15,2]	1.0000402	1.0005099	67.8646728	67.8708000	67.7966679	20001.0	0.0203633	0.95	62.4086000	73.6878000
thresh_age[16,2]	1.0001320	1.0007968	68.9916620	68.9713000	68.6413265	19445.1	0.0217017	0.95	63.0912000	74.9601000
thresh_age[17,2]	1.0000108	1.0003232	69.3599232	69.3926000	69.6245400	20001.0	0.0204695	0.95	63.6989000	74.9891000
thresh_age[20,2]	1.0001989	1.0010469	67.6724548	67.6800000	67.8412685	19476.3	0.0212477	0.95	61.8253000	73.4316000
age.s[1]	0.9999657	1.0001538	21.5681873	21.0723000	19.5744553	20001.0	0.0146505	0.95	19.0002000	25.6467000
age.s[2]	1.0003803	1.0014825	41.6809785	41.6414000	41.1032385	18893.3	0.0241010	0.95	35.0273000	47.9551000
age.s[3]	0.9999711	1.0001532	33.4008690	33.3043000	32.6141726	19099.0	0.0278082	0.95	26.1177000	41.2261000
age.s[4]	1.0000324	1.0001740	64.4410164	64.2999000	63.9635438	20001.0	0.0307451	0.95	56.1455000	73.1761000
age.s[5]	0.9998910	0.9999322	88.1334296	88.4399000	88.6992835	20001.0	0.0445258	0.95	77.2853000	99.9999000
age.s[6]	1.0002898	1.0005929	68.6419117	68.4803000	68.3157766	20001.0	0.0340125	0.95	59.4441000	78.0093000
age.s[7]	0.9999135	1.0000793	30.7872503	30.6718000	30.8385562	20620.7	0.0267369	0.95	23.6537000	38.3963000
age.s[8]	1.0000334	1.0003920	64.3147853	64.2105000	64.8596966	20001.0	0.0304808	0.95	56.0800000	72.9504000
age.s[9]	1.0004775	1.0020076	63.6977340	63.5783000	63.4091998	19497.1	0.0308124	0.95	55.2528000	72.0691000
age.s[10]	1.0000681	1.0005964	39.9610163	39.9312000	40.2955273	21436.8	0.0233679	0.95	33.4839000	46.8288000
age.s[11]	0.9999127	0.9999993	30.8285630	30.6322000	29.7779045	20001.0	0.0271276	0.95	23.5683000	38.3553000
age.s[12]	1.0001423	1.0003387	49.8088735	49.7419000	50.0802517	19550.5	0.0246735	0.95	43.1395000	56.6298000
age.s[13]	1.0002250	1.0011447	36.5457381	36.5072000	36.1141394	19532.3	0.0262111	0.95	29.3452000	43.5779000
age.s[14]	1.0000935	1.0004783	29.4581772	29.2447000	28.9582375	19428.0	0.0268278	0.95	22.4978000	37.0006000
age.s[15]	0.9999508	1.0000379	80.4824091	80.2004000	79.7102857	20001.0	0.0443585	0.95	68.2107000	92.7039000
age.s[16]	1.0003056	1.0013109	73.0042551	72.8365000	72.9255730	20001.0	0.0353087	0.95	63.4100000	82.9876000
age.s[17]	1.0000310	1.0002873	77.7677827	77.4728000	77.1306878	19602.0	0.0420857	0.95	66.2102000	89.3144000
age.s[18]	1.0001261	1.0004674	36.1556086	36.1377000	36.8989524	19176.3	0.0261072	0.95	29.5010000	43.6467000
age.s[19]	1.0004057	1.0013737	91.7401990	92.4413000	95.1626206	20001.0	0.0387319	0.95	81.5797000	99.9997000
age.s[20]	1.0001122	1.0004038	68.0707217	67.9203000	67.3717195	20001.0	0.0331063	0.95	58.9565000	77.0947000
age.s[21]	1.0006378	1.0019089	41.6954000	41.6450000	41.4589850	18859.1	0.0243969	0.95	35.4465000	48.6131000
age.s[22]	1.0001708	1.0005107	65.1158817	64.9694000	64.7421169	19495.7	0.0316587	0.95	56.6619000	74.0524000
age.s[23]	1.0000266	1.0003358	27.6459960	27.4551000	26.6267645	19178.6	0.0249861	0.95	21.0933000	34.4018000
age.s[24]	1.0001685	1.0004661	21.5920316	21.1277000	19.5414592	20001.0	0.0145171	0.95	19.0002000	25.5975000
age.s[25]	1.0001626	1.0008925	57.4591200	57.3735000	57.3645842	19018.9	0.0283962	0.95	49.9780000	65.2581000
age.s[26]	0.9999953	1.0000671	86.4731176	86.5409000	86.9904824	20001.0	0.0447669	0.95	75.6648000	99.5689000
age.s[27]	1.0005708	1.0021418	68.1616004	68.0390000	67.9788062	20206.3	0.0330677	0.95	58.8785000	77.2818000

age.s[28]	1.0001478	1.0008795	36.2908474	36.2525000	36.2097456	18160.8	0.0264311	0.95	29.4122000	43.3348000
age.s[29]	1.0003769	1.0017475	54.8686964	54.7742000	55.1593696	19492.0	0.0269344	0.95	47.8165000	62.3442000
age.s[30]	1.0006742	1.0021464	32.5968365	32.4921000	32.1191740	19152.0	0.0278790	0.95	25.1731000	40.1777000
age.s[31]	0.9999811	1.0002311	66.9536465	66.8530000	66.8071919	20634.6	0.0314355	0.95	58.1757000	75.7489000
age.s[32]	1.0001496	1.0007971	69.0995744	68.9721000	69.4712554	20001.0	0.0339545	0.95	59.7912000	78.5995000
age.s[33]	1.0000999	1.0002025	91.7657967	92.4876000	95.1717148	21912.0	0.0371010	0.95	81.5533000	99.9984000
age.s[34]	0.9999342	1.0001092	22.6340825	22.2134000	21.1714099	20845.4	0.0174932	0.95	19.0004000	27.3518000
age.s[35]	1.0011042	1.0040619	34.8573992	34.8137000	34.8703544	19076.2	0.0266829	0.95	27.9193000	42.2476000
age.s[36]	0.9999973	1.0003559	85.8818283	85.8721000	85.6168076	20001.0	0.0453131	0.95	74.5033000	98.7856000
age.s[37]	0.9999652	1.0002106	77.0481878	76.8403000	75.6044224	21285.4	0.0392195	0.95	66.0522000	88.4141000
age.s[38]	0.9998806	0.9999400	21.5657473	21.0993000	19.6011316	20001.0	0.0144781	0.95	19.0003000	25.6102000
age.s[39]	1.0000723	1.0004579	87.0790013	87.1781000	86.8239330	20494.4	0.0450494	0.95	76.2152000	99.9908000
age.s[40]	1.0000222	1.0003180	88.0739141	88.3155000	88.8329084	20001.0	0.0446426	0.95	77.2663000	99.9849000
age.s[41]	1.0001831	1.0008392	53.6588729	53.5685000	53.2059755	20001.0	0.0257535	0.95	46.5056000	60.6548000
age.s[42]	1.0000888	1.0001792	21.5638267	21.0923000	19.6040671	20001.0	0.0144704	0.95	19.0006000	25.6152000
age.s[43]	1.0001125	1.0003788	32.6486337	32.5732000	32.6337466	20001.0	0.0273429	0.95	25.2239000	40.1876000
age.s[44]	1.0012482	1.0049379	51.2089846	51.1504000	51.5513009	19722.1	0.0256243	0.95	43.8004000	57.9164000
age.s[45]	1.0002823	1.0004954	77.8727209	77.6437000	77.0348293	20001.0	0.0410368	0.95	66.6239000	89.4362000
age.s[46]	1.0001488	1.0009098	23.2917385	22.9652000	22.8246533	19549.2	0.0195033	0.95	19.0002000	28.2775000
age.s[47]	1.0002489	1.0007378	35.8415391	35.8254000	36.4495723	19805.8	0.0257869	0.95	28.8167000	42.9402000
age.s[48]	1.0002374	1.0008188	36.4146941	36.3995000	35.8870577	19508.5	0.0261377	0.95	29.1873000	43.3553000
age.s[49]	1.0008965	1.0028223	25.6939630	25.4582000	24.9072642	19600.3	0.0228778	0.95	19.5067000	31.7547000
age.s[50]	0.9999836	1.0000559	21.5835585	21.1295000	19.5314665	20001.0	0.0144728	0.95	19.0009000	25.6020000
age.s[51]	1.0002836	1.0014279	79.6235357	79.3186000	78.4514225	20001.0	0.0425161	0.95	68.1943000	91.7254000
age.s[52]	1.0003508	1.0005040	54.0306513	53.9434000	54.0542750	19410.3	0.0264062	0.95	46.7287000	61.0606000
age.s[53]	1.0006222	1.0023443	48.7247471	48.6353000	48.3804273	19147.3	0.0250164	0.95	42.2778000	55.7726000
age.s[54]	1.0007997	1.0031542	69.4311860	69.2894000	67.8424637	20001.0	0.0339890	0.95	60.2483000	78.9835000
age.s[55]	0.9999987	1.0002555	62.5280946	62.4096000	62.3275315	20324.5	0.0295648	0.95	54.3554000	70.8383000
age.s[56]	0.9999304	1.0001190	72.2983909	72.1096000	71.9481258	20001.0	0.0361639	0.95	62.2947000	82.2316000
age.s[57]	1.0002796	1.0012774	42.9181603	42.8494000	42.5395103	20145.6	0.0238804	0.95	36.3077000	49.5163000
age.s[58]	1.0005527	1.0023939	32.6947920	32.6272000	32.1282854	18974.5	0.0281414	0.95	25.2508000	40.3207000
age.s[59]	1.0001015	1.0006060	76.8374348	76.6152000	76.1162722	18014.6	0.0427517	0.95	65.7232000	88.0628000
age.s[60]	0.9999822	1.0002490	31.3324937	31.1942000	30.9411035	17959.0	0.0277714	0.95	24.2623000	38.6367000
age.s[61]	1.0001209	1.0005874	33.4130223	33.3372000	33.6919515	18321.2	0.0282018	0.95	25.7982000	40.7233000
age.s[62]	1.0002758	1.0014135	61.1301259	61.0444000	60.1070558	20001.0	0.0299598	0.95	52.6546000	69.2495000
age.s[63]	1.0002584	1.0011965	87.2285623	87.3398000	87.0288480	20001.0	0.0451199	0.95	76.3954000	99.9519000
age.s[64]	1.0002226	1.0011788	59.2622033	59.1889000	59.3543810	19291.2	0.0286165	0.95	51.5706000	67.0983000
age.s[65]	1.0001855	1.0010710	75.3669106	75.1682000	74.3944140	20659.5	0.0388246	0.95	65.0099000	86.6900000
age.s[66]	1.0003415	1.0009804	62.7681188	62.6740000	62.3940760	20001.0	0.0296358	0.95	54.4470000	70.8280000

age.s[67]	1.0003896	1.0017434	30.8132131	30.6796000	30.7573330	20001.0	0.0269384	0.95	23.5875000	38.3961000
age.s[68]	1.0001974	1.0009372	24.1412181	23.8670000	23.0337988	19384.1	0.0208101	0.95	19.0029000	29.2957000
age.s[69]	0.9998969	0.9999939	68.2926190	68.1779000	68.1653849	20001.0	0.0326679	0.95	59.5345000	77.4769000
age.s[70]	1.0001595	1.0007885	33.4151218	33.3615000	33.7812106	20001.0	0.0271621	0.95	25.9819000	40.8841000
age.s[71]	1.0001359	1.0008855	44.3353981	44.2740000	44.3528171	18623.0	0.0240088	0.95	37.9739000	50.8419000
age.s[72]	1.0004601	1.0018022	29.4678105	29.3305000	29.6767090	18852.7	0.0268586	0.95	22.6143000	36.8635000
age.s[73]	1.0001636	1.0003819	82.8456555	82.5706000	82.1978547	21565.4	0.0440832	0.95	70.6350000	95.8297000
age.s[74]	1.0001118	1.0007324	57.0732908	56.9799000	56.3067394	19571.9	0.0273435	0.95	49.5591000	64.5632000
age.s[75]	1.0003063	1.0010737	54.0777277	54.0049000	53.9429361	19403.2	0.0265778	0.95	46.9358000	61.4500000
age.s[76]	1.0000129	1.0003911	30.8088803	30.6686000	30.4375553	20001.0	0.0268120	0.95	23.7882000	38.4660000
age.s[77]	1.0005470	1.0020637	41.4449960	41.4002000	41.3438422	18752.3	0.0240802	0.95	35.0338000	47.8963000
age.s[78]	1.0003670	1.0011862	75.9454762	75.6823000	75.3038690	19115.1	0.0410452	0.95	65.0042000	87.2304000
age.s[79]	1.0001187	1.0003176	21.5851635	21.0984000	19.5512227	20001.0	0.0145872	0.95	19.0001000	25.5954000
age.s[80]	0.9999734	1.0002353	37.4694032	37.4693000	37.9992518	19392.3	0.0259472	0.95	30.5095000	44.6361000
age.s[81]	1.0001211	1.0007994	24.1162505	23.8543000	23.6208209	20001.0	0.0203831	0.95	19.0041000	29.2544000
age.s[82]	1.0000316	1.0000631	67.4166187	67.3123000	67.8109154	20001.0	0.0325030	0.95	58.6022000	76.3913000
age.s[83]	0.9998966	0.9999019	34.2371679	34.1839000	33.8252582	20001.0	0.0268968	0.95	26.6386000	41.4955000
age.s[84]	1.0000127	1.0002742	30.8306516	30.6314000	29.9839607	18969.7	0.0278440	0.95	23.3862000	38.1449000
age.s[85]	1.0000065	1.0003994	58.4474884	58.3276000	57.9496558	20001.0	0.0281637	0.95	50.8636000	66.4043000
age.s[86]	0.9999523	1.0002205	66.2771948	66.1894000	66.1181729	19524.9	0.0318332	0.95	57.6980000	75.1829000
age.s[87]	1.0003402	1.0014062	53.5478315	53.4474000	53.0486298	19071.1	0.0267412	0.95	46.2837000	60.6823000
age.s[88]	1.0001548	1.0009511	30.7945598	30.6784000	30.6548830	20001.0	0.0269653	0.95	23.3553000	38.0774000
age.s[89]	1.0002429	1.0012445	51.1154219	51.0527000	50.9497266	19273.7	0.0254422	0.95	44.2872000	58.0682000
age.s[90]	1.0004228	1.0015584	48.3585331	48.2875000	48.1027150	18999.6	0.0249350	0.95	41.7820000	55.1699000
age.s[91]	1.0000887	1.0005814	63.0206940	62.8651000	61.9302165	20001.0	0.0301813	0.95	54.6740000	71.3321000
age.s[92]	1.0001062	1.0002343	63.9814064	63.8404000	63.0790827	20001.0	0.0303935	0.95	55.4183000	72.3059000
age.s[93]	1.0000610	1.0004250	63.8029461	63.6597000	63.3727607	19591.2	0.0308076	0.95	55.4040000	72.2935000
age.s[94]	0.9998521	0.9998550	66.0480494	65.9239000	65.7542093	19563.9	0.0318816	0.95	57.3749000	74.7514000
age.s[95]	1.0002755	1.0008628	70.2397217	70.0625000	68.7904081	20001.0	0.0348836	0.95	60.8250000	80.1342000
age.s[96]	1.0002935	1.0009664	21.5704982	21.0824000	19.5795118	20001.0	0.0145054	0.95	19.0002000	25.5303000
age.s[97]	1.0000756	1.0006329	32.1070582	32.0049000	31.8024745	20001.0	0.0270056	0.95	24.6849000	39.5330000
age.s[98]	1.0004902	1.0020027	21.6099676	21.1265000	19.5496616	19121.5	0.0150811	0.95	19.0001000	25.6597000
age.s[99]	1.0001906	1.0006175	21.5471996	21.0624000	19.5832930	20001.0	0.0144876	0.95	19.0003000	25.5417000
age.s[100]	1.0000981	1.0003974	68.9275894	68.7546000	68.0121255	20001.0	0.0337039	0.95	59.9572000	78.5296000
age.s[101]	1.0002933	1.0004803	73.3198027	73.1416000	73.0822633	20338.5	0.0368072	0.95	63.3081000	83.9972000
age.s[102]	1.0002254	1.0005902	84.8830830	84.7838000	84.1624917	20001.0	0.0450697	0.95	73.0954000	97.6979000
age.s[103]	1.0001922	1.0007430	76.3531392	76.1156000	75.6271539	20001.0	0.0399685	0.95	65.3178000	87.4087000
age.s[104]	1.0001827	1.0007913	55.5539688	55.4482000	54.9476439	19524.9	0.0269171	0.95	48.1424000	62.8606000
age.s[105]	0.9998985	0.9999258	73.1519206	72.9879000	72.6154401	19485.1	0.0371470	0.95	63.3277000	83.5665000

age.s[106]	1.0001260	1.0004997	56.7274346	56.6400000	56.4600740	20001.0	0.0275117	0.95	49.1460000	64.4226000
age.s[107]	0.9999402	1.0000794	63.2708212	63.1763000	62.3664900	20001.0	0.0305419	0.95	54.8247000	71.6446000
age.s[108]	1.0002682	1.0013062	57.2620412	57.1911000	57.1139606	20001.0	0.0273393	0.95	49.9173000	65.1640000
age.s[109]	1.0003212	1.0010268	88.3067604	88.5965000	89.0358584	20001.0	0.0443847	0.95	77.4051000	99.9984000
age.s[110]	0.9998878	0.9999222	80.6825553	80.3716000	79.4101638	20001.0	0.0437636	0.95	68.8032000	93.0716000
age.s[111]	1.0006946	1.0017780	21.5882246	21.1115000	19.5782162	20001.0	0.0146288	0.95	19.0003000	25.5888000
age.s[112]	1.0001430	1.0007870	27.7043067	27.5004000	27.0853367	19438.8	0.0247720	0.95	21.0840000	34.4731000
age.s[113]	0.9999089	0.9999744	30.8198941	30.6754000	30.4966960	20001.0	0.0269593	0.95	23.7070000	38.4628000
age.s[114]	1.0006912	1.0028576	55.3577890	55.2401000	54.4145861	19414.4	0.0270876	0.95	48.0757000	62.8909000
age.s[115]	1.0002500	1.0011907	44.1512513	44.1089000	44.3325692	18988.0	0.0233727	0.95	37.7291000	50.3181000
age.s[116]	1.0000284	1.0003261	65.9098863	65.8236000	65.7453756	20001.0	0.0319123	0.95	56.9795000	74.6672000
age.s[117]	1.0000195	1.0004612	21.5606390	21.0867000	19.6435943	20085.5	0.0144773	0.95	19.0000000	25.5878000
age.s[118]	1.0001103	1.0007256	23.9828994	23.7109000	23.0921902	19483.7	0.0207867	0.95	19.0001000	29.1431000
age.s[119]	1.0000393	1.0002349	63.1271102	63.0008000	63.2008334	20001.0	0.0303303	0.95	54.7232000	71.4870000
age.s[120]	1.0001879	1.0009769	73.7577474	73.5372000	73.5867760	20001.0	0.0376038	0.95	63.4163000	84.0877000
age.s[121]	1.0002823	1.0012406	66.8457285	66.6956000	66.3420688	20181.7	0.0323067	0.95	58.1115000	75.9855000
age.s[122]	1.0001640	1.0006046	75.3129805	75.0682000	74.0745337	20001.0	0.0392101	0.95	64.8170000	86.3956000
age.s[123]	1.0011616	1.0043807	32.8919993	32.8010000	32.5276239	18038.6	0.0258476	0.95	25.9575000	39.5583000
age.s[124]	1.0002352	1.0010003	32.5541055	32.4772000	32.7402758	20001.0	0.0273826	0.95	25.1890000	40.2852000
age.s[125]	1.0002884	1.0005325	46.6114201	46.5474000	46.6511383	19378.5	0.0240759	0.95	40.3024000	53.3439000
age.s[126]	1.0002175	1.0007044	56.6050391	56.5373000	55.8103952	20001.0	0.0274136	0.95	49.1868000	64.2300000
age.s[127]	1.0009855	1.0029565	21.5718444	21.1067000	19.5611454	21093.4	0.0142024	0.95	19.0002000	25.5939000
age.s[128]	1.0001021	1.0006670	55.6330081	55.5186000	55.4353051	20001.0	0.0265969	0.95	48.5817000	63.3140000
age.s[129]	1.0003728	1.0014324	64.5931344	64.4763000	63.9171991	19583.7	0.0315558	0.95	56.0664000	73.1934000
age.s[130]	0.9999730	1.0002227	66.6487891	66.5698000	67.2711643	20001.0	0.0316351	0.95	57.6972000	75.1272000
age.s[131]	0.9999576	1.0001644	26.8492845	26.6478000	26.3703154	19092.7	0.0243275	0.95	20.4100000	33.4594000
age.s[132]	1.0000151	1.0004388	48.7163343	48.6633000	48.5838389	18690.6	0.0252230	0.95	41.9456000	55.3709000
age.s[133]	1.0000682	1.0002809	67.1547993	66.9865000	65.9263333	18927.3	0.0333090	0.95	58.3805000	76.3300000
age.s[134]	1.0000496	1.0003789	35.2781034	35.2652000	35.7460058	19587.4	0.0266937	0.95	28.1427000	42.7362000
age.s[135]	1.0002167	1.0010706	47.9070286	47.8024000	47.4261051	20001.0	0.0241391	0.95	41.1665000	54.5068000
age.s[136]	1.0002244	1.0011038	45.2036699	45.1443000	45.1440522	19076.5	0.0242769	0.95	38.8666000	51.8941000
age.s[137]	1.0003148	1.0015181	59.1104840	58.9716000	58.4720182	20001.0	0.0285302	0.95	51.5442000	67.3223000
age.s[138]	1.0000791	1.0006850	24.1426993	23.8668000	23.5877680	19385.9	0.0208904	0.95	19.0006000	29.3239000
age.s[139]	1.0000602	1.0005177	65.4667180	65.3592000	64.6591247	20001.0	0.0309094	0.95	57.0380000	73.9292000
age.s[140]	1.0003603	1.0017138	27.6254880	27.4316000	27.1071070	18452.0	0.0251446	0.95	21.2822000	34.5282000
age.s[141]	1.0001825	1.0010067	34.0744853	34.0166000	34.2773753	19574.5	0.0265417	0.95	27.0302000	41.4208000
age.s[142]	1.0003034	1.0014412	46.9678656	46.8909000	46.4287541	20001.0	0.0241749	0.95	40.3159000	53.6622000
age.s[143]	0.9998767	0.9999179	33.0952643	33.0172000	33.1108330	18661.8	0.0264890	0.95	26.0991000	40.2045000
age.s[144]	0.9999901	1.0001564	63.2378522	63.1358000	62.5510429	20001.0	0.0297766	0.95	54.9606000	71.4752000

age.s[145]	0.9999573	1.0001418	60.2162337	60.1168000	60.0157716	20001.0	0.0289939	0.95	52.5041000	68.5509000
age.s[146]	0.9999909	1.0003574	31.7305279	31.5697000	31.0109993	19559.6	0.0274399	0.95	24.7272000	39.4927000
age.s[147]	0.9999901	1.0002048	63.9563726	63.8336000	62.8653333	20001.0	0.0307323	0.95	55.6218000	72.4849000
age.s[148]	1.0004426	1.0017469	34.9778878	34.9342000	34.8256204	19066.6	0.0271763	0.95	27.4449000	42.1295000
age.s[149]	0.9998742	0.9999059	55.8488889	55.7690000	55.7547074	19398.3	0.0269631	0.95	48.4069000	63.1105000
age.s[150]	0.9999632	1.0001288	68.3510659	68.2311000	67.9667341	20001.0	0.0331324	0.95	59.2156000	77.5316000
age.s[151]	1.0003787	1.0014749	46.9073957	46.8499000	46.9355246	19319.0	0.0237613	0.95	40.5177000	53.3387000
age.s[152]	0.9999905	1.0003003	34.0947790	34.0163000	33.8351511	19327.5	0.0269763	0.95	26.6273000	41.1096000
age.s[153]	1.0004138	1.0017538	64.5004554	64.3947000	64.5781100	19515.0	0.0307497	0.95	56.1380000	72.7884000
age.s[154]	1.0001293	1.0005169	54.5081342	54.4628000	54.7739226	19597.9	0.0267969	0.95	47.3240000	62.0293000
age.s[155]	0.9999856	1.0002504	26.5107584	26.3102000	25.7254033	19426.6	0.0239602	0.95	20.1638000	32.9585000
age.s[156]	1.0000968	1.0006838	67.0301379	66.8710000	66.7042351	20001.0	0.0328999	0.95	58.3391000	76.4231000
age.s[157]	1.0000646	1.0000928	61.2773895	61.1901000	61.2491926	20001.0	0.0290633	0.95	53.0108000	69.2613000
age.s[158]	1.0002821	1.0011587	71.5843333	71.4055000	70.9013887	19548.9	0.0353951	0.95	62.3111000	81.6066000
age.s[159]	1.0010448	1.0038132	48.0102629	47.9421000	47.8052264	20089.8	0.0241893	0.95	41.4106000	54.7778000
age.s[160]	0.9999719	1.0001046	46.5488378	46.4807000	46.3399333	19347.5	0.0240596	0.95	40.0665000	53.2127000
age.s[161]	0.9999363	0.9999391	42.6469329	42.6053000	42.6547851	18895.9	0.0245021	0.95	36.1159000	49.2744000
age.s[162]	1.0000388	1.0004185	35.2826358	35.2215000	35.2247399	19085.3	0.0267101	0.95	28.0816000	42.4245000
age.s[163]	1.0002278	1.0009240	37.7649924	37.7203000	37.6916532	18906.7	0.0258204	0.95	30.9411000	44.7431000
age.s[164]	1.0000948	1.0006397	24.8638365	24.6357000	23.9482373	19500.8	0.0217416	0.95	19.1941000	30.3750000
age.s[165]	1.0008243	1.0033241	47.0463796	46.9894000	46.9161430	19717.3	0.0242468	0.95	40.2116000	53.5239000
age.s[166]	1.0001549	1.0009039	21.5624687	21.0711000	19.5604652	20001.0	0.0146236	0.95	19.0001000	25.5779000
age.s[167]	0.9998918	0.9999259	78.6310525	78.4165000	78.1930535	20001.0	0.0405328	0.95	67.2433000	89.8918000
age.s[168]	1.0002352	1.0006617	58.3362843	58.2426000	58.2163777	20001.0	0.0275031	0.95	50.8373000	66.0828000
age.s[169]	1.0001656	1.0007571	48.4776556	48.3880000	47.9689343	19201.9	0.0245939	0.95	41.8242000	55.1230000
age.s[170]	1.0007806	1.0031436	37.3783494	37.3567000	37.4146408	20001.0	0.0256152	0.95	30.2872000	44.5410000
age.s[171]	1.0003963	1.0016434	36.4753148	36.4451000	36.5107793	19359.6	0.0260779	0.95	29.5840000	43.7041000
age.s[172]	0.9999909	1.0002369	33.4314505	33.3578000	32.6000360	19028.0	0.0278609	0.95	26.0994000	40.9687000
age.s[173]	1.0000080	1.0000927	68.3445517	68.1728000	67.5636825	19200.5	0.0339345	0.95	59.3777000	77.6464000
age.s[174]	1.0001739	1.0003330	58.2117206	58.1016000	57.7754503	20001.0	0.0282973	0.95	50.4663000	66.0758000
age.s[175]	1.0000427	1.0005138	37.9552393	37.9342000	37.6404461	18953.1	0.0251031	0.95	31.2312000	44.8833000
age.s[176]	0.9999813	1.0002506	77.7067822	77.5215000	77.6219323	20001.0	0.0412169	0.95	66.7215000	89.4820000
age.s[177]	0.9999927	1.0002218	55.2745830	55.1942000	55.7045997	20001.0	0.0268939	0.95	47.7680000	62.7297000
age.s[178]	1.0000411	1.0004135	53.4039443	53.3308000	52.9791324	19526.9	0.0262393	0.95	46.2567000	60.6497000
age.s[179]	1.0011814	1.0029436	21.5890395	21.1193000	19.5733318	19512.6	0.0149198	0.95	19.0007000	25.6879000
age.s[180]	1.0004488	1.0017612	36.6043154	36.5658000	36.5478425	19048.2	0.0259700	0.95	29.5574000	43.5192000
age.s[181]	1.0002555	1.0006519	73.0234955	72.8279000	72.4346154	20001.0	0.0372870	0.95	62.9824000	83.4524000
age.s[182]	1.0000103	1.0003599	76.5094273	76.3107000	76.1223380	20001.0	0.0395813	0.95	65.8139000	87.5665000
age.s[183]	0.9999497	1.0001934	68.7074051	68.5268000	67.7826546	20001.0	0.0338804	0.95	59.5099000	78.1785000

age.s[184]	1.0000098	1.0002652	30.8092962	30.6645000	30.8848698	20710.0	0.0266352	0.95	23.4897000	38.2578000
age.s[185]	0.9999363	1.0000826	84.8740212	84.7538000	84.3934351	20001.0	0.0454232	0.95	73.2235000	97.9677000
age.s[186]	1.0002594	1.0012594	60.2243794	60.1784000	60.2648730	18897.4	0.0298899	0.95	52.0305000	68.1249000
age.s[187]	1.0002962	1.0010260	66.1627219	66.0514000	65.2415768	18994.6	0.0325229	0.95	57.7334000	75.3117000
age.s[188]	0.9999987	1.0002282	72.8099460	72.6306000	72.2293202	20001.0	0.0368876	0.95	62.9630000	83.4027000
age.s[189]	1.0004794	1.0021564	81.9206754	81.7197000	81.8654416	20001.0	0.0442504	0.95	70.1850000	94.6312000
age.s[190]	1.0001627	1.0008107	32.6340707	32.5747000	32.6053822	19133.7	0.0279625	0.95	25.2378000	40.2499000
age.s[191]	1.0006277	1.0024866	54.5362780	54.4699000	54.0437926	19081.3	0.0263775	0.95	47.3258000	61.4991000
age.s[192]	1.0003583	1.0009113	60.2179408	60.1215000	60.1547174	18893.9	0.0295850	0.95	52.1865000	68.0870000
age.s[193]	1.0004968	1.0022262	35.7437148	35.7484000	36.0961061	19602.6	0.0264937	0.95	28.8198000	43.3435000
age.s[194]	1.0001989	1.0003518	75.1971858	75.0375000	75.0556960	20001.0	0.0389759	0.95	64.6536000	86.0541000
age.s[195]	0.9999114	0.9999487	80.0376451	79.7449000	79.2173009	20203.3	0.0420575	0.95	68.8221000	92.0758000
age.s[196]	1.0005066	1.0021621	44.5125252	44.4770000	43.9874500	18113.4	0.0247257	0.95	38.0222000	51.0073000
age.s[197]	1.0008155	1.0032826	52.9308887	52.8470000	52.7586732	20001.0	0.0258344	0.95	45.8454000	60.2041000
age.s[198]	1.0008468	1.0014552	21.5629154	21.0661000	19.5644011	20001.0	0.0146486	0.95	19.0003000	25.6000000
age.s[199]	1.0000595	1.0005964	54.9932186	54.9144000	54.8601653	19053.0	0.0274707	0.95	47.7008000	62.5874000
age.s[200]	1.0000481	1.0001266	24.1149627	23.8399000	23.4042374	19600.8	0.0206354	0.95	19.0016000	29.2789000

2.1.6 40 traits

Table 22: MCMC diagnostic data for CAMSAD dataset (n traits = 40).

	PSRF Point est.	PSRF Upper C.I.	Mean	Median	Mode	ESS	MCSE	HDImass	HDIlow	HDIhigh
b	1.0005987	1.0014396	0.0266824	0.0257804	0.0221034	18250.3	0.0000358	0.95	0.0200004	0.0358798
a	1.0002057	1.0007437	0.0191188	0.0194030	0.0197430	18581.4	0.0000376	0.95	0.0102840	0.0278988
M	1.0001383	1.0005915	29.9135982	30.0233000	30.4551942	18284.4	0.1085904	0.95	2.3797300	53.8417000
beta0[1]	0.9999616	0.9999770	-4.6156700	-4.5805300	-4.5549830	20001.0	0.0078355	0.95	-6.8268400	-2.4927600
beta0[2]	1.0007071	1.0027283	-15.8736286	-15.4389000	-14.7296581	14761.1	0.0285509	0.95	-22.7674000	-9.6091600
beta0[3]	1.0001097	1.0001368	-11.5024993	-11.2703000	-10.9849089	17031.9	0.0177635	0.95	-16.0781000	-7.2352200
beta0[4]	0.9999289	0.9999554	-17.1878076	-16.7305000	-15.6424285	13210.6	0.0315651	0.95	-24.6337000	-10.8712000
beta0[5]	1.0000036	1.0003567	-17.8310014	-17.3878000	-16.8716646	13667.2	0.0302098	0.95	-25.0428000	-11.7310000
beta[1]	1.0000025	1.0000524	1.4334775	1.4250200	1.4208944	20001.0	0.0020647	0.95	0.8765640	2.0197200
beta[2]	1.0006263	1.0025619	4.0512406	3.9516500	3.8572764	14819.3	0.0069188	0.95	2.5262100	5.7345300
beta[3]	1.0000355	1.0000458	3.6530290	3.5859000	3.4952487	16885.8	0.0051284	0.95	2.4302800	4.9615800
beta[4]	0.9999466	1.0000331	4.8175629	4.6949100	4.3647381	13251.0	0.0084041	0.95	3.0767500	6.7329000
beta[5]	0.9999831	1.0003200	4.8958519	4.7895100	4.6678375	13664.8	0.0077336	0.95	3.2749900	6.6935800
thresh[1,1]	NaN	NaN	0.5000000	0.5000000	0.4996355	0.0	NaN	0.95	0.5000000	0.5000000
thresh[2,1]	NaN	NaN	0.5000000	0.5000000	0.4996355	0.0	NaN	0.95	0.5000000	0.5000000
thresh[3,1]	NaN	NaN	0.5000000	0.5000000	0.4996355	0.0	NaN	0.95	0.5000000	0.5000000
thresh[4,1]	NaN	NaN	0.5000000	0.5000000	0.4996355	0.0	NaN	0.95	0.5000000	0.5000000
thresh[5,1]	NaN	NaN	0.5000000	0.5000000	0.4996355	0.0	NaN	0.95	0.5000000	0.5000000
thresh[3,2]	1.0001249	1.0002356	4.1838714	4.1239700	3.9989827	16752.3	0.0039747	0.95	3.2825800	5.2214100
thresh[5,2]	1.0001205	1.0008071	2.2692257	2.2347800	2.2051877	15020.5	0.0024751	0.95	1.7118500	2.8648300
thresh_age[1,1]	1.0001014	1.0006277	35.2968162	35.0517000	34.6217675	19056.0	0.0289958	0.95	27.4338000	43.2545000
thresh_age[2,1]	1.0003979	1.0016196	56.9385101	56.5019000	55.2024311	19292.9	0.0335480	0.95	48.5345000	66.1306000
thresh_age[3,1]	1.0001254	1.0004166	26.6809075	26.3114000	25.8743158	18138.7	0.0191536	0.95	22.2392000	32.1545000
thresh_age[4,1]	1.0004793	1.0012922	39.3769474	38.7832000	37.9660554	18140.1	0.0277368	0.95	33.1440000	47.0806000
thresh_age[5,1]	1.0002770	1.0008229	42.2491550	41.6725000	40.5524560	18103.3	0.0293820	0.95	35.6755000	50.5430000
thresh_age[3,2]	1.0002782	1.0011676	73.8563464	73.2947000	72.2792144	19384.5	0.0455717	0.95	61.9669000	86.1441000
thresh_age[5,2]	1.0001025	1.0005009	60.7997742	60.3701000	59.2686426	17962.4	0.0358762	0.95	51.9928000	70.1634000
age.s[1]	0.9999207	1.0000613	24.5385293	23.4096000	20.2682098	19070.6	0.0332201	0.95	19.0004000	33.4815000
age.s[2]	1.0001089	1.0006114	42.5122217	41.8597000	40.0419495	20001.0	0.0556096	0.95	27.6352000	58.0200000
age.s[3]	1.0002843	1.0007323	30.6369560	29.9289000	27.5384663	20001.0	0.0474045	0.95	19.0006000	42.8265000
age.s[4]	1.0000481	1.0005004	55.9006936	55.2031000	52.9337078	19034.6	0.0702890	0.95	38.0980000	75.5433000
age.s[5]	1.0003264	1.0011456	68.9327400	68.4588000	68.0661370	19580.6	0.0791705	0.95	48.2562000	90.8355000
age.s[6]	1.0000473	1.0001487	58.9407287	58.1956000	55.5103666	20001.0	0.0708717	0.95	40.0226000	78.8342000
age.s[7]	0.9999265	0.9999549	30.6883477	29.9731000	29.1625651	19257.5	0.0482091	0.95	19.0171000	42.7948000
age.s[8]	1.0006945	1.0015881	65.0337846	64.3058000	62.1982988	18313.7	0.0800268	0.95	44.2037000	86.1023000

age.s[9]	1.0000785	1.0004343	58.2918607	57.5874000	54.9218555	20001.0	0.0685985	0.95	40.0116000	77.6717000
age.s[10]	1.0002565	1.0005459	30.6465588	29.8776000	28.1105046	19411.2	0.0481577	0.95	19.0032000	42.8559000
age.s[11]	1.0005028	1.0012177	30.6368328	29.9833000	28.9412717	20001.0	0.0470991	0.95	19.0008000	42.5588000
age.s[12]	0.9999225	0.9999904	30.6875749	29.9494000	29.5954353	19582.0	0.0479380	0.95	19.0091000	42.8699000
age.s[13]	1.0004914	1.0017005	33.4941707	32.9313000	32.5536209	20001.0	0.0499707	0.95	19.6332000	46.5468000
age.s[14]	1.0002431	1.0009162	26.5925235	25.5517000	23.0186294	20001.0	0.0389221	0.95	19.0001000	37.0662000
age.s[15]	1.0000103	1.0004150	68.8022313	68.1845000	65.9578417	20001.0	0.0785285	0.95	47.9965000	90.8938000
age.s[16]	1.0001212	1.0005206	78.0955246	78.3097000	78.8556621	20001.0	0.0763335	0.95	59.2915000	99.2597000
age.s[17]	1.0000468	1.0003568	68.8649290	68.2889000	67.7521515	20001.0	0.0789680	0.95	47.8566000	91.2958000
age.s[18]	1.0001464	1.0008151	33.3800872	32.8471000	30.5830368	19312.0	0.0501127	0.95	19.7748000	46.2554000
age.s[19]	0.9999828	1.0002997	77.9503027	78.1347000	75.8629217	20001.0	0.0763108	0.95	59.6071000	99.6936000
age.s[20]	1.0005710	1.0016830	58.3574386	57.6500000	56.9415401	20001.0	0.0688796	0.95	40.0396000	77.7680000
age.s[21]	0.9999219	1.0000934	39.9453485	39.3042000	37.9245519	19223.2	0.0547462	0.95	25.7687000	55.0353000
age.s[22]	1.0000827	1.0006169	41.5382585	40.9964000	40.0089198	17588.0	0.0574799	0.95	27.6460000	56.9736000
age.s[23]	0.9999404	1.0001301	33.3967431	32.8428000	32.2866082	20001.0	0.0500347	0.95	19.7050000	46.4906000
age.s[24]	1.0001517	1.0002316	24.6262574	23.5047000	20.2427503	20001.0	0.0327307	0.95	19.0006000	33.6404000
age.s[25]	0.9999432	0.9999897	47.6389699	46.9445000	44.9564600	20001.0	0.0584678	0.95	32.8051000	64.7431000
age.s[26]	0.9998745	0.9999105	78.1815538	78.3552000	78.1147985	20001.0	0.0766784	0.95	59.8668000	99.7998000
age.s[27]	1.0000631	1.0005358	55.2346127	54.4944000	52.5014124	19238.0	0.0673841	0.95	38.0618000	73.9805000
age.s[28]	0.9998866	0.9999692	30.6146442	29.9035000	29.4808903	20001.0	0.0473051	0.95	19.0016000	42.7511000
age.s[29]	0.9999974	1.0000997	41.8254698	41.2265000	39.8450109	18485.9	0.0547264	0.95	28.3595000	56.9332000
age.s[30]	1.0000149	1.0002496	33.4293936	32.8999000	32.5155749	20001.0	0.0501118	0.95	20.0657000	46.8283000
age.s[31]	1.0001627	1.0007565	59.1146016	58.4549000	58.5776920	20001.0	0.0707416	0.95	40.4019000	79.1038000
age.s[32]	0.9999718	1.0000923	50.2897394	49.5307000	48.7860896	20001.0	0.0627192	0.95	33.5142000	67.3727000
age.s[33]	1.0000551	1.0005316	78.0490417	78.1826000	78.1634706	20001.0	0.0765973	0.95	59.5226000	99.3364000
age.s[34]	0.9999357	1.0000590	24.6249660	23.5564000	20.2920120	19537.3	0.0330151	0.95	19.0003000	33.6343000
age.s[35]	0.9999157	1.0000035	33.5681854	33.1182000	33.2883043	18908.9	0.0478881	0.95	21.5526000	46.7586000
age.s[36]	1.0001962	1.0010665	78.0880563	78.1926000	77.8662072	20001.0	0.0763914	0.95	59.2226000	99.3152000
age.s[37]	1.0002063	1.0011344	54.7201950	53.9963000	52.0856624	18846.0	0.0645183	0.95	38.0616000	72.2066000
age.s[38]	1.0000911	1.0003784	24.6188379	23.4627000	20.3022316	20384.2	0.0323470	0.95	19.0004000	33.7773000
age.s[39]	0.9999108	0.9999544	69.0003272	68.3666000	66.8350876	20001.0	0.0785446	0.95	47.8473000	90.7243000
age.s[40]	1.0003113	1.0015081	78.0869257	78.3746000	79.7781423	20001.0	0.0770857	0.95	59.7053000	99.7182000
age.s[41]	1.0002964	1.0010077	41.7773990	41.1707000	40.5349782	18000.9	0.0559008	0.95	28.0652000	56.9164000
age.s[42]	0.9999348	1.0000840	24.5972146	23.5020000	20.1593032	20001.0	0.0322894	0.95	19.0003000	33.5538000
age.s[43]	1.0001321	1.0006672	33.4688369	32.8897000	31.2610318	19496.8	0.0505568	0.95	20.1526000	46.9133000
age.s[44]	0.9999458	0.9999947	41.8203828	41.2487000	40.7542821	19428.1	0.0535781	0.95	27.7574000	56.7215000
age.s[45]	0.9999114	0.9999162	59.1983420	58.4081000	56.1544762	20001.0	0.0717036	0.95	41.1650000	80.1829000
age.s[46]	0.9999794	1.0000131	24.6224860	23.5008000	20.2907131	20001.0	0.0325481	0.95	19.0014000	33.6342000
age.s[47]	1.0005929	1.0021449	30.7347333	30.0191000	28.6061561	20001.0	0.0472350	0.95	19.1325000	42.8813000

age.s[48]	1.0001866	1.0010277	33.3859595	32.8240000	31.3671711	20001.0	0.0498417	0.95	20.5139000	47.2945000
age.s[49]	1.0004493	1.0010944	26.6168572	25.6054000	22.1203467	20001.0	0.0387587	0.95	19.0001000	36.9708000
age.s[50]	0.9999733	1.0002818	24.6266881	23.5171000	20.2377273	20001.0	0.0328291	0.95	19.0033000	33.7800000
age.s[51]	1.0000478	1.0000925	65.2445992	64.5362000	62.6544913	20001.0	0.0763100	0.95	44.6839000	86.3694000
age.s[52]	1.0001878	1.0004631	50.1750796	49.4108000	47.5121771	18735.9	0.0648884	0.95	34.2415000	68.3983000
age.s[53]	1.0001846	1.0010765	39.9602705	39.2768000	38.0435637	19491.9	0.0540663	0.95	26.0030000	55.0475000
age.s[54]	1.0003942	1.0010088	78.0016961	78.1287000	77.3413235	20001.0	0.0765003	0.95	59.0666000	99.2532000
age.s[55]	1.0001426	1.0007795	68.9040062	68.4461000	68.3824764	18927.3	0.0808259	0.95	48.0268000	90.6028000
age.s[56]	1.0004821	1.0012969	55.4219174	54.6505000	53.1996063	19203.6	0.0674535	0.95	38.2325000	74.1531000
age.s[57]	1.0000227	1.0001651	42.4092857	41.7792000	41.2432941	19437.9	0.0564280	0.95	27.9755000	58.4180000
age.s[58]	1.0000150	1.0004319	33.4410917	32.8660000	31.4659595	20001.0	0.0502039	0.95	20.0151000	46.7079000
age.s[59]	1.0001995	1.0009463	64.9353064	64.2199000	62.1809620	20001.0	0.0771254	0.95	44.6246000	86.7367000
age.s[60]	0.9999679	1.0000329	33.5513370	33.0338000	32.7318409	19226.6	0.0470315	0.95	21.0079000	46.0460000
age.s[61]	0.9999865	1.0002654	40.0175281	39.3487000	37.9921464	19568.0	0.0541116	0.95	26.2034000	55.1938000
age.s[62]	1.0001281	1.0008257	55.9604056	55.1697000	53.0875578	18656.1	0.0707402	0.95	37.7464000	74.8948000
age.s[63]	0.9999996	1.0002826	67.3871959	66.7813000	66.3064040	19344.1	0.0763019	0.95	48.1316000	89.3759000
age.s[64]	1.0000345	1.0005059	42.4824403	41.8418000	40.2796486	19603.9	0.0562960	0.95	27.6647000	58.1575000
age.s[65]	1.0004912	1.0021669	78.1447702	78.2705000	77.3350876	20001.0	0.0764899	0.95	59.6219000	99.3744000
age.s[66]	1.0002820	1.0008986	50.3017181	49.6018000	47.1693210	17875.5	0.0652378	0.95	34.3141000	67.7345000
age.s[67]	1.0003412	1.0014021	30.6515681	29.9102000	27.9038256	20457.3	0.0467622	0.95	19.0222000	43.0042000
age.s[68]	1.0003195	1.0008800	24.6165964	23.4928000	20.1017966	20001.0	0.0327006	0.95	19.0002000	33.6857000
age.s[69]	0.9999867	1.0001397	58.0545211	57.4939000	56.8862898	20001.0	0.0650678	0.95	41.5364000	77.0654000
age.s[70]	1.0004711	1.0014752	33.4596860	32.8918000	31.6304708	19593.0	0.0505746	0.95	20.7130000	47.4420000
age.s[71]	1.0003629	1.0006275	39.7526007	39.2470000	38.2183386	18991.6	0.0524141	0.95	26.8616000	54.9061000
age.s[72]	1.0005957	1.0010744	24.5976736	23.4698000	20.2062302	20001.0	0.0325413	0.95	19.0001000	33.6617000
age.s[73]	1.0007759	1.0029645	69.0391328	68.5962000	68.6454954	19302.7	0.0802210	0.95	48.1506000	91.2201000
age.s[74]	1.0007633	1.0027534	58.3270277	57.5684000	56.7561357	20001.0	0.0692006	0.95	40.2917000	78.0143000
age.s[75]	1.0002877	1.0011641	55.3940150	54.7935000	53.3423502	20001.0	0.0623225	0.95	39.0430000	73.0125000
age.s[76]	1.0000763	1.0005323	30.6862035	29.9950000	28.1727468	20001.0	0.0473091	0.95	19.0080000	42.8293000
age.s[77]	1.0003594	1.0015833	41.8631060	41.2915000	39.9452496	18107.3	0.0558573	0.95	28.1621000	57.4588000
age.s[78]	1.0001806	1.0009722	59.1284625	58.4836000	57.9138542	18782.2	0.0729807	0.95	40.6327000	79.1020000
age.s[79]	0.9999172	0.9999862	24.6179084	23.5195000	20.2119070	20001.0	0.0326744	0.95	19.0002000	33.6080000
age.s[80]	0.9999243	0.9999811	40.0388877	39.4242000	38.3152732	19467.9	0.0540630	0.95	25.9050000	55.0620000
age.s[81]	0.9999280	1.0001293	24.6185888	23.5365000	20.2800728	20001.0	0.0325140	0.95	19.0002000	33.7462000
age.s[82]	1.0001101	1.0003698	50.2608815	49.5842000	47.4437286	18808.8	0.0631376	0.95	34.3528000	67.8591000
age.s[83]	1.0000955	1.0004994	33.4191028	32.8940000	32.5423399	20009.4	0.0499384	0.95	19.8308000	46.3099000
age.s[84]	0.9999916	1.0002109	30.6637704	29.9433000	28.3581839	20001.0	0.0472152	0.95	19.0179000	42.7297000
age.s[85]	1.0002239	1.0010293	50.2673217	49.5562000	48.1650934	20062.2	0.0614489	0.95	34.3326000	67.8266000
age.s[86]	1.0007529	1.0024741	66.1543879	65.4782000	63.8322868	19915.2	0.0730563	0.95	46.9277000	86.9717000

age.s[87]	0.9999072	0.9999725	41.8540003	41.2997000	41.2300305	19402.1	0.0540523	0.95	28.0507000	57.0931000
age.s[88]	1.0000587	1.0004289	30.6007578	29.9073000	28.5413189	20001.0	0.0469735	0.95	19.0026000	42.6644000
age.s[89]	1.0004416	1.0018782	50.3289906	49.6395000	48.3784775	20001.0	0.0617013	0.95	34.3678000	68.0262000
age.s[90]	1.0002490	1.0012049	42.4736338	41.8263000	40.9675501	18922.8	0.0578324	0.95	27.5223000	58.2037000
age.s[91]	1.0000955	1.0001239	50.2850582	49.5978000	48.7788523	20001.0	0.0615534	0.95	34.7480000	68.0698000
age.s[92]	1.0004426	1.0013597	58.3287727	57.6509000	55.8428983	19105.4	0.0708392	0.95	40.3887000	78.1353000
age.s[93]	1.0002014	1.0002125	55.9184196	55.2323000	55.0447607	19223.2	0.0694950	0.95	38.4186000	75.3795000
age.s[94]	1.0002870	1.0010108	50.3433179	49.6664000	47.9411314	20001.0	0.0613315	0.95	34.3231000	67.7206000
age.s[95]	1.0001129	1.0007729	67.3389003	66.6640000	65.1878483	20001.0	0.0753532	0.95	47.4228000	88.8713000
age.s[96]	1.0000160	1.0001060	24.6099088	23.4492000	20.3690170	20001.0	0.0325935	0.95	19.0006000	33.6739000
age.s[97]	1.0006158	1.0020379	26.6656235	25.6663000	23.3105805	20001.0	0.0389469	0.95	19.0007000	37.0271000
age.s[98]	1.0010844	1.0026141	24.6173885	23.5157000	20.3269831	19500.6	0.0327385	0.95	19.0003000	33.5455000
age.s[99]	1.0003729	1.0013138	24.6311969	23.5281000	20.1102905	20001.0	0.0329061	0.95	19.0007000	33.7014000
age.s[100]	1.0001870	1.0009643	58.3255973	57.6218000	56.2582177	19308.3	0.0708663	0.95	39.8585000	77.3997000
age.s[101]	1.0005932	1.0016001	59.0956878	58.4370000	56.2998805	20001.0	0.0706654	0.95	39.8197000	78.3853000
age.s[102]	0.9999228	0.9999251	78.1843023	78.3693000	78.6516386	20001.0	0.0773507	0.95	59.7863000	99.9255000
age.s[103]	1.0009369	1.0029980	74.1393377	73.8452000	71.7483902	20001.0	0.0781517	0.95	54.1341000	96.3525000
age.s[104]	1.0002028	1.0008862	42.3738116	41.7581000	41.5133720	19492.9	0.0558521	0.95	28.0605000	58.0365000
age.s[105]	0.9999729	1.0001594	59.0221159	58.2734000	56.3072520	18931.4	0.0728907	0.95	39.8976000	78.6446000
age.s[106]	1.0004460	1.0010406	50.1771780	49.4175000	48.5447972	19487.5	0.0617512	0.95	33.8951000	67.1291000
age.s[107]	0.9999920	1.0003730	58.3325244	57.6351000	56.3958984	20001.0	0.0684831	0.95	40.5732000	77.5609000
age.s[108]	1.0000302	1.0004390	47.7118704	47.0019000	44.2580563	19505.5	0.0597379	0.95	32.1578000	64.3131000
age.s[109]	1.0001859	1.0010387	78.0141225	78.3157000	79.1173936	20001.0	0.0766519	0.95	59.4697000	99.5783000
age.s[110]	0.9999076	1.0000393	78.0113525	78.2583000	79.7179510	20001.0	0.0769540	0.95	58.9216000	99.1754000
age.s[111]	1.0006816	1.0017329	24.6078252	23.4819000	20.2202594	20001.0	0.0325938	0.95	19.0002000	33.6620000
age.s[112]	1.0000974	1.0002831	30.6580259	30.0288000	29.7319931	20001.0	0.0469278	0.95	19.0039000	42.7681000
age.s[113]	1.0001468	1.0005893	30.6092342	29.9170000	27.3219879	19273.0	0.0477482	0.95	19.0007000	42.6426000
age.s[114]	1.0000821	1.0006457	55.3623518	54.7567000	52.8470631	19298.2	0.0644810	0.95	38.7035000	73.0531000
age.s[115]	1.0007781	1.0029213	33.3409371	32.8243000	30.7364764	17868.8	0.0526856	0.95	20.2484000	46.6623000
age.s[116]	1.0008693	1.0027964	55.3184282	54.5738000	53.5750460	18342.7	0.0693115	0.95	37.9903000	74.2360000
age.s[117]	1.0002814	1.0006294	24.6455787	23.5054000	20.1852199	19079.4	0.0339133	0.95	19.0001000	33.7684000
age.s[118]	1.0000880	1.0004436	26.6203771	25.6245000	21.0574049	20001.0	0.0389819	0.95	19.0010000	37.0133000
age.s[119]	1.0000148	1.0003531	78.1776253	78.3136000	77.1753774	20001.0	0.0765567	0.95	59.3215000	99.2349000
age.s[120]	1.0001164	1.0007018	67.5593473	66.9024000	64.9233752	20001.0	0.0750981	0.95	47.7931000	88.9907000
age.s[121]	1.0006069	1.0024796	58.2739156	57.5532000	55.3051170	19568.6	0.0696828	0.95	40.4289000	77.8602000
age.s[122]	1.0009879	1.0038831	78.0585666	78.1818000	76.6404060	20001.0	0.0769784	0.95	58.8725000	99.1104000
age.s[123]	1.0001025	1.0001832	41.7072778	41.0815000	39.3065248	19336.6	0.0530520	0.95	28.1306000	56.5078000
age.s[124]	1.0000771	1.0005336	30.7305920	30.0642000	29.7414193	20001.0	0.0471541	0.95	19.0126000	42.6950000
age.s[125]	1.0001266	1.0004754	30.6219631	29.9011000	28.1425195	20001.0	0.0473285	0.95	19.0504000	42.8100000

age.s[126]	1.0001271	1.0006312	47.7042611	46.9954000	45.6538884	20001.0	0.0587422	0.95	32.1280000	64.2402000
age.s[127]	1.0003012	1.0010908	24.5810871	23.4424000	20.1951041	19383.5	0.0331453	0.95	19.0009000	33.6303000
age.s[128]	0.9999906	1.0002467	58.1068327	57.4611000	54.0153747	20001.0	0.0657819	0.95	40.8175000	76.5242000
age.s[129]	1.0004556	1.0018510	65.0654172	64.3117000	63.5582610	20001.0	0.0773587	0.95	44.7466000	87.1212000
age.s[130]	0.9998713	0.9999141	58.1281801	57.5046000	57.1173474	20001.0	0.0654761	0.95	40.6930000	76.1966000
age.s[131]	1.0000193	1.0002689	30.6680486	29.9723000	28.3928169	20001.0	0.0472452	0.95	19.0146000	42.6457000
age.s[132]	1.0001993	1.0010242	40.2777274	39.6075000	39.1226501	18564.1	0.0519985	0.95	26.8636000	54.2855000
age.s[133]	1.0001955	1.0010090	69.0996719	68.6514000	69.0555176	20001.0	0.0790614	0.95	48.0757000	91.3064000
age.s[134]	1.0002462	1.0005778	33.3766490	32.7961000	31.2128828	17994.6	0.0529378	0.95	19.9900000	46.7037000
age.s[135]	1.0001951	1.0010313	50.2999340	49.6184000	48.6285653	18134.1	0.0646938	0.95	34.0508000	67.2484000
age.s[136]	1.0003004	1.0011225	47.7221727	47.0039000	45.6067916	20001.0	0.0586734	0.95	32.2000000	64.1045000
age.s[137]	1.0006844	1.0019557	55.3458669	54.6385000	53.0959813	19630.7	0.0672236	0.95	37.8738000	74.1419000
age.s[138]	1.0005538	1.0019950	24.6063594	23.4682000	20.1882623	20001.0	0.0327390	0.95	19.0003000	33.7237000
age.s[139]	0.9999640	1.0000225	66.2225743	65.7044000	64.9328688	19314.6	0.0737958	0.95	46.5064000	86.2385000
age.s[140]	1.0009806	1.0026865	33.4205723	32.8289000	32.3274270	19436.7	0.0508515	0.95	19.8380000	46.7060000
age.s[141]	0.9999359	1.0000505	26.6281164	25.5862000	23.7560728	19206.0	0.0399156	0.95	19.0023000	37.1836000
age.s[142]	1.0000400	1.0004578	30.6540029	29.9741000	29.2796775	19565.7	0.0476454	0.95	19.0026000	42.7474000
age.s[143]	0.9999905	1.0002271	24.6484291	23.5458000	20.1590726	20001.0	0.0326572	0.95	19.0002000	33.7518000
age.s[144]	1.0001319	1.0007015	78.1213636	78.2112000	76.8621932	20001.0	0.0761898	0.95	59.8432000	99.9115000
age.s[145]	1.0000924	1.0007006	50.2795002	49.6047000	49.0577974	19602.0	0.0621954	0.95	34.0745000	67.4797000
age.s[146]	0.9999730	1.0000954	33.4597130	32.9452000	32.8145092	19466.5	0.0508368	0.95	19.7363000	46.4881000
age.s[147]	1.0000046	1.0002846	59.0091134	58.3077000	55.3549081	19476.5	0.0723024	0.95	40.0593000	78.9820000
age.s[148]	1.0005170	1.0021689	42.4182184	41.7032000	40.5780103	20001.0	0.0555097	0.95	28.0717000	58.3092000
age.s[149]	1.0000611	1.0003274	58.3316982	57.6285000	56.9132824	19122.2	0.0707456	0.95	40.1861000	77.5291000
age.s[150]	1.0000220	1.0002773	66.1889184	65.5356000	64.7615721	19583.2	0.0742384	0.95	46.8041000	86.8185000
age.s[151]	0.9998747	0.9999269	33.4574725	32.9412000	33.1947157	20001.0	0.0497477	0.95	20.1850000	46.8312000
age.s[152]	0.9998786	0.9999183	40.0246698	39.3505000	39.0160967	19473.9	0.0543203	0.95	25.7045000	54.8076000
age.s[153]	1.0001294	1.0007854	58.5042884	57.7635000	55.8673196	20001.0	0.0699836	0.95	40.1866000	78.3360000
age.s[154]	0.9999296	0.9999997	47.6853063	47.0266000	44.6440103	19388.7	0.0592835	0.95	32.1541000	64.0067000
age.s[155]	0.9999115	1.0000178	26.6284844	25.6375000	21.3142930	20001.0	0.0388936	0.95	19.0045000	37.0102000
age.s[156]	1.0012974	1.0045542	55.3669333	54.6306000	52.6622376	19224.5	0.0678529	0.95	37.2873000	73.6152000
age.s[157]	1.0010915	1.0039591	58.4259747	57.7455000	55.0231527	18350.0	0.0717773	0.95	40.7229000	78.3674000
age.s[158]	1.0000125	1.0003876	58.2936624	57.5560000	55.8384148	18540.7	0.0719746	0.95	40.4226000	77.9234000
age.s[159]	1.0006017	1.0016234	42.4618991	41.7489000	39.8906224	18966.5	0.0576798	0.95	27.9948000	58.6598000
age.s[160]	1.0000411	1.0005408	42.1956611	41.5027000	39.2422403	19352.7	0.0531367	0.95	28.9832000	57.1199000
age.s[161]	0.9999797	1.0002616	33.4213229	32.8489000	31.5568861	19358.2	0.0508203	0.95	19.8396000	46.5011000
age.s[162]	1.0002882	1.0013334	33.4251106	32.8540000	31.0468229	20001.0	0.0495617	0.95	20.5665000	47.1370000
age.s[163]	1.0001710	1.0003828	39.9886003	39.3712000	38.9470278	20001.0	0.0532363	0.95	26.2764000	55.3636000
age.s[164]	1.0002323	1.0010424	26.6139560	25.6171000	22.2581277	20001.0	0.0387304	0.95	19.0014000	37.0124000

age.s[165]	1.0000482	1.0003691	42.3847258	41.7250000	40.3709773	19552.9	0.0562656	0.95	27.9796000	58.3661000
age.s[166]	1.0001026	1.0002624	24.6156085	23.5423000	20.1425762	20001.0	0.0325826	0.95	19.0003000	33.5904000
age.s[167]	1.0000019	1.0000827	68.9293757	68.3486000	67.9827734	19320.7	0.0797176	0.95	48.1750000	90.9045000
age.s[168]	1.0001553	1.0006454	58.2719012	57.5862000	56.4422540	19272.9	0.0701193	0.95	39.9647000	77.3728000
age.s[169]	1.0002119	1.0011830	42.4666152	41.8618000	40.1810835	20001.0	0.0550749	0.95	27.8082000	57.9580000
age.s[170]	1.0000704	1.0004466	42.1785061	41.5668000	40.5348092	19073.6	0.0542991	0.95	28.1772000	57.1703000
age.s[171]	0.9999617	1.0000336	33.4112764	32.9084000	32.4934893	20001.0	0.0497288	0.95	20.1862000	46.6792000
age.s[172]	0.9999497	1.0000677	30.6328756	29.8923000	28.3415438	20633.3	0.0462162	0.95	19.0764000	42.7005000
age.s[173]	1.0002796	1.0010136	33.3693224	32.8353000	31.6133259	20001.0	0.0498695	0.95	20.0935000	46.7006000
age.s[174]	1.0006187	1.0024151	50.4425391	49.7864000	48.5918254	20001.0	0.0617226	0.95	33.7503000	67.3546000
age.s[175]	1.0001080	1.0006862	30.6528473	29.9752000	29.1015453	19389.8	0.0476220	0.95	19.0101000	42.6325000
age.s[176]	1.0004937	1.0019660	55.3028068	54.6381000	54.4834802	20001.0	0.0658275	0.95	38.1919000	73.9045000
age.s[177]	1.0009461	1.0030687	47.7846878	47.0976000	44.9419367	19458.9	0.0609135	0.95	31.8796000	64.3080000
age.s[178]	1.0000336	1.0003463	58.3067267	57.5407000	56.0393367	19319.7	0.0706843	0.95	40.4976000	78.3080000
age.s[179]	1.0004393	1.0010105	24.5961772	23.4699000	20.1726576	20412.7	0.0324060	0.95	19.0000000	33.6788000
age.s[180]	1.0000228	1.0000481	40.0122969	39.3936000	37.9643035	19325.5	0.0545289	0.95	26.3054000	55.4860000
age.s[181]	1.0003797	1.0015707	69.0464686	68.4368000	67.6967566	20001.0	0.0788431	0.95	48.6363000	91.6428000
age.s[182]	1.0003873	1.0012289	68.9999439	68.3764000	65.9070980	18857.5	0.0818518	0.95	49.0010000	92.3309000
age.s[183]	1.0001414	1.0006124	77.9488152	78.0520000	77.7049225	20001.0	0.0764277	0.95	58.9091000	99.2363000
age.s[184]	1.0001719	1.0009453	30.6281748	29.9135000	29.0759014	20001.0	0.0474882	0.95	19.0355000	42.9650000
age.s[185]	1.0002232	1.0007625	74.0891669	73.7868000	72.7674284	18902.2	0.0804211	0.95	53.6143000	96.0206000
age.s[186]	1.0002916	1.0009738	59.0942213	58.2847000	55.6765548	18879.8	0.0735564	0.95	40.3207000	78.9743000
age.s[187]	1.0002861	1.0013908	48.3455800	47.7323000	47.5269538	18083.4	0.0631196	0.95	32.0556000	64.8565000
age.s[188]	1.0003679	1.0012095	68.8727525	68.3161000	66.6299506	20001.0	0.0789917	0.95	47.0036000	90.4730000
age.s[189]	0.9999018	1.0000329	78.0226224	78.1862000	78.7751879	20001.0	0.0765503	0.95	58.9178000	98.9985000
age.s[190]	0.9999040	1.0000381	33.4971812	32.9154000	32.3699462	20001.0	0.0503150	0.95	20.0733000	46.8411000
age.s[191]	1.0003152	1.0008081	39.7489610	39.1250000	38.0548774	18313.7	0.0532803	0.95	26.4734000	54.3923000
age.s[192]	1.0001257	1.0008631	50.2795392	49.5805000	48.2047037	20001.0	0.0611484	0.95	34.5783000	67.8707000
age.s[193]	1.0001212	1.0004147	33.4063142	32.8042000	31.2434630	20001.0	0.0502213	0.95	20.3651000	47.2150000
age.s[194]	1.0000850	1.0006511	55.3870954	54.6729000	53.6655816	20001.0	0.0666544	0.95	37.4014000	73.5353000
age.s[195]	0.9999159	0.9999498	68.8831884	68.3154000	67.6299892	20001.0	0.0785990	0.95	48.0563000	91.0205000
age.s[196]	1.0003389	1.0013826	50.3210786	49.5861000	47.8311950	18763.6	0.0639359	0.95	34.2556000	67.7831000
age.s[197]	0.9999083	0.9999814	50.2557196	49.5343000	48.2877193	20001.0	0.0611701	0.95	34.4425000	67.8776000
age.s[198]	1.0004359	1.0010750	24.6335759	23.5103000	20.1525170	19945.9	0.0331445	0.95	19.0000000	33.7951000
age.s[199]	1.0000609	1.0005248	55.4098807	54.7161000	52.8045473	19522.9	0.0673160	0.95	37.6692000	73.8569000
age.s[200]	1.0008502	1.0028729	24.6392472	23.5197000	20.2615773	18142.6	0.0342143	0.95	19.0002000	33.6576000

2.1.7 64 traits

Table 23: MCMC diagnostic data for CAMSAD dataset (n traits = 64).

	PSRF Point est.	PSRF Upper C.I.	Mean	Median	Mode	ESS	MCSE	HDImass	HDIlow	HDIhigh
b	1.0002437	1.0011291	3.583290e-02	0.0358072	0.0358775	19372.6	1.990000e-05	0.95	0.0306027	0.0414121
a	1.0002219	1.0010520	1.048680e-02	0.0103466	0.0099868	19408.8	1.310000e-05	0.95	0.0070860	0.0141021
M	1.0002279	1.0010518	5.328682e+01	53.6716000	54.5656060	19415.2	3.195270e-02	0.95	44.4202000	61.4813000
beta0[1]	1.0000789	1.0001453	-4.670462e+00	-4.6611200	-4.7549518	21812.2	6.282900e-03	0.95	-6.4314500	-2.8268700
beta0[2]	0.9999824	1.0000572	-3.907380e+00	-3.8958300	-3.9999648	20530.3	7.132300e-03	0.95	-5.8539000	-1.8630800
beta0[3]	0.9999371	1.0000268	-3.488227e+00	-3.4591900	-3.3421702	19275.4	7.171400e-03	0.95	-5.4675200	-1.5544100
beta0[4]	0.9999366	1.0000830	-4.528632e+00	-4.5239800	-4.6172450	20001.0	6.501400e-03	0.95	-6.3096400	-2.7144700
beta0[5]	1.0000622	1.0005251	-4.480313e+00	-4.4685800	-4.4504540	20001.0	6.374700e-03	0.95	-6.2318700	-2.6974200
beta0[6]	0.9999664	1.0000651	-4.875842e+00	-4.8631800	-4.8705868	19569.8	6.630100e-03	0.95	-6.6731700	-3.0452000
beta0[7]	1.0000732	1.0005556	-3.963942e+00	-3.9547000	-3.8984904	20001.0	6.231700e-03	0.95	-5.7010200	-2.2326800
beta0[8]	0.9999698	1.0000385	-4.588317e+00	-4.5709000	-4.4318231	20001.0	6.632400e-03	0.95	-6.4781500	-2.8250600
beta0[9]	1.0005287	1.0021316	-4.091087e+00	-4.0788100	-4.1122854	20050.7	6.488200e-03	0.95	-5.8914900	-2.3154700
beta0[10]	1.0000628	1.0003937	-2.446775e+01	-24.3363000	-24.2055947	16654.1	2.301410e-02	0.95	-30.5928000	-19.0353000
beta0[11]	0.9999851	1.0000626	-2.436955e+01	-24.2487000	-23.9007198	16276.2	2.324640e-02	0.95	-30.0999000	-18.6254000
beta0[12]	0.9999568	0.9999980	-2.109315e+01	-21.0155000	-21.0895526	18645.8	1.760320e-02	0.95	-25.7926000	-16.4768000
beta0[13]	1.0001149	1.0005120	-2.460907e+01	-24.4823000	-24.2469989	17162.9	2.174990e-02	0.95	-30.3791000	-19.2585000
beta0[14]	1.0000718	1.0005079	-2.336051e+01	-23.2493000	-23.2872663	15748.0	2.132970e-02	0.95	-28.6841000	-18.2005000
beta0[15]	1.0007272	1.0021648	-2.578404e+01	-25.6660000	-25.6519312	16300.1	2.343580e-02	0.95	-31.5396000	-19.8796000
beta0[16]	0.9998999	0.9999870	-2.278195e+01	-22.6800000	-22.1163485	17815.1	1.964450e-02	0.95	-27.9021000	-17.6413000
beta0[17]	0.9999334	1.0000959	-2.121706e+01	-21.1187000	-20.6790650	18311.8	1.769870e-02	0.95	-25.8687000	-16.6374000
beta0[18]	0.9999253	1.0001108	-2.204944e+01	-21.9491000	-21.5376413	17522.6	1.974660e-02	0.95	-27.1539000	-16.9582000
beta0[19]	1.0001139	1.0007061	-2.277554e+01	-22.6387000	-22.1544729	17537.6	1.993370e-02	0.95	-27.8981000	-17.6199000
beta0[20]	1.0002533	1.0011555	-2.328482e+01	-23.1913000	-23.0715568	17170.3	2.083890e-02	0.95	-28.8581000	-18.2077000
beta0[21]	1.0004122	1.0004920	-2.216579e+01	-22.0672000	-21.7646620	17910.3	1.851260e-02	0.95	-27.1467000	-17.4855000
beta0[22]	1.0000109	1.0002452	-2.639080e+01	-26.2727000	-26.1826962	15955.4	2.495710e-02	0.95	-32.6540000	-20.3319000
beta0[23]	1.0000531	1.0004990	-2.075637e+01	-20.6810000	-20.2929143	18404.9	1.666020e-02	0.95	-25.4163000	-16.5274000
beta0[24]	1.0003808	1.0011989	-1.886492e+01	-18.7899000	-18.5880654	18751.6	1.544540e-02	0.95	-22.9417000	-14.7323000
beta0[25]	1.0012173	1.0048605	-1.921495e+01	-19.1244000	-18.9204474	18633.8	1.534740e-02	0.95	-23.3325000	-15.1649000
beta0[26]	1.0001984	1.0006789	-1.920472e+01	-19.1013000	-18.7614477	19137.9	1.572690e-02	0.95	-23.5120000	-15.0043000
beta0[27]	1.0002001	1.0006377	-8.364917e+00	-8.2736900	-8.0919263	20001.0	1.214890e-02	0.95	-11.7717000	-5.1282800
beta0[28]	1.0004164	1.0013431	-1.587136e+01	-15.7857000	-15.3184136	18991.7	1.563150e-02	0.95	-20.1389000	-11.7567000
beta0[29]	1.0000678	1.0005527	-1.342270e+01	-13.3144000	-13.1224208	19501.2	1.341780e-02	0.95	-17.1818000	-9.8736200
beta0[30]	1.0000212	1.0003206	-1.313879e+01	-13.0645000	-13.1023959	20001.0	1.293960e-02	0.95	-16.7779000	-9.6700300
beta0[31]	1.0001277	1.0004807	-7.966706e+00	-7.9195700	-7.8497613	20001.0	9.222700e-03	0.95	-10.5725000	-5.4782400
beta0[32]	1.0001584	1.0008933	-1.230900e+01	-12.1752000	-11.3014329	19052.3	1.767100e-02	0.95	-17.1307000	-7.6336600

beta0[33]	1.0001284	1.0005991	-1.716147e+01	-17.0315000	-16.8367953	18435.9	2.020530e-02	0.95	-22.5968000	-11.8986000
beta0[34]	1.0005771	1.0024033	-1.947537e+01	-19.3169000	-19.0944734	17359.4	2.275020e-02	0.95	-25.4423000	-13.7687000
beta0[35]	1.0000133	1.0002331	-1.376228e+01	-13.6596000	-13.6576544	19534.3	1.601060e-02	0.95	-18.2335000	-9.4919800
beta0[36]	0.9999843	1.0000338	-1.222789e+01	-12.1450000	-12.0407332	18862.3	1.331340e-02	0.95	-15.8303000	-8.7212000
beta0[37]	1.0004272	1.0012255	-1.066603e+01	-10.5443000	-10.5526060	20649.1	1.562660e-02	0.95	-15.1095000	-6.4354800
beta0[38]	1.0001900	1.0004275	-9.700127e+00	-9.6459000	-9.5420587	19185.8	9.488700e-03	0.95	-12.2925000	-7.1839300
beta0[39]	1.0000905	1.0007113	-1.356371e+01	-13.4895000	-13.0082364	19342.6	1.317440e-02	0.95	-17.1544000	-10.0218000
beta0[40]	1.0001713	1.0004527	-1.177958e+01	-11.7144000	-11.6143597	19493.8	1.124730e-02	0.95	-14.9090000	-8.7857600
beta0[41]	1.0003462	1.0015663	-1.970982e+01	-19.5836000	-19.5257936	18093.5	2.052180e-02	0.95	-25.2126000	-14.5263000
beta0[42]	1.0000176	1.0003267	-1.410451e+01	-14.0199000	-13.4115736	19143.0	1.655180e-02	0.95	-18.6698000	-9.7892200
beta0[43]	1.0002784	1.0011722	-1.272991e+01	-12.6364000	-12.0500299	20001.0	1.501490e-02	0.95	-16.8951000	-8.6558300
beta0[44]	1.0008487	1.0035160	-1.984814e+01	-19.7228000	-19.1641946	16783.2	2.171260e-02	0.95	-25.4828000	-14.4661000
beta0[45]	1.0001117	1.0008027	-2.115972e+01	-21.0062000	-20.7777730	16903.1	2.319510e-02	0.95	-27.1695000	-15.4541000
beta0[46]	1.0001415	1.0009073	-1.306813e+01	-12.9871000	-12.8804890	19352.4	1.379410e-02	0.95	-16.9180000	-9.4307000
beta0[47]	1.0001124	1.0006868	-1.124384e+01	-11.1727000	-10.9710672	20001.0	1.203260e-02	0.95	-14.6637000	-8.0814500
beta0[48]	0.9999316	1.0000422	-1.445744e+01	-14.3574000	-14.2109248	20001.0	1.496800e-02	0.95	-18.7162000	-10.5547000
beta0[49]	1.0002406	1.0008908	-1.324072e+01	-13.1709000	-12.8832231	19265.1	1.296860e-02	0.95	-16.8197000	-9.8177700
beta0[50]	1.0000030	1.0003322	-1.360181e+01	-13.5255000	-13.0830182	19175.0	1.327640e-02	0.95	-17.2538000	-10.1389000
beta0[51]	0.9999326	0.9999940	-2.406645e+01	-23.8994000	-23.4307712	12053.4	2.871010e-02	0.95	-30.4173000	-18.2402000
beta0[52]	1.0001021	1.0005755	-9.818300e+00	-9.7770200	-9.7400400	19544.7	9.094800e-03	0.95	-12.3912000	-7.3878000
beta0[53]	1.0000302	1.0003963	-1.764821e+01	-17.5108000	-17.2246405	13180.0	2.160190e-02	0.95	-22.5594000	-12.9604000
beta0[54]	1.0000574	1.0004642	-1.689301e+01	-16.7802000	-16.4350985	18973.2	1.656540e-02	0.95	-21.5041000	-12.6093000
beta0[55]	1.0007169	1.0028304	-1.651726e+01	-16.4078000	-16.1892548	14055.9	1.973350e-02	0.95	-21.0868000	-11.9855000
beta0[56]	1.0003783	1.0015792	-1.147931e+01	-11.4206000	-11.3167732	19239.3	1.104690e-02	0.95	-14.5609000	-8.5737100
beta0[57]	0.9999919	1.0000717	-9.250798e+00	-9.2259200	-9.1061014	20001.0	8.089200e-03	0.95	-11.4780000	-7.0309500
beta0[58]	1.0001312	1.0006779	-1.252840e+01	-12.4912000	-12.4517791	20001.0	9.627200e-03	0.95	-15.2776000	-9.9317300
beta0[59]	0.9999009	1.0000325	-1.295699e+01	-12.8915000	-12.9013057	18582.9	1.231980e-02	0.95	-16.4154000	-9.8547800
beta0[60]	0.9998938	0.9999858	-1.587912e+01	-15.7630000	-15.3290889	18292.2	1.860160e-02	0.95	-20.7240000	-10.9269000
beta0[61]	0.9999632	1.0001620	-1.151797e+01	-11.4579000	-11.3586730	19385.8	1.115280e-02	0.95	-14.6054000	-8.5582500
beta0[62]	1.0005652	1.0017211	-1.647621e+01	-16.3959000	-16.3210472	18931.2	1.449730e-02	0.95	-20.4543000	-12.7339000
beta0[63]	1.0003746	1.0017667	-1.289460e+01	-12.8501000	-12.8491599	19519.8	1.008060e-02	0.95	-15.5923000	-10.0652000
beta0[64]	1.0001979	1.0008828	-1.559050e+01	-15.5280000	-15.6250226	19614.5	1.238290e-02	0.95	-18.9909000	-12.3265000
beta[1]	1.0000668	1.0001190	1.513974e+00	1.5114800	1.5205348	21724.2	1.652700e-03	0.95	1.0307400	1.9774700
beta[2]	1.0000066	1.0000806	9.341551e-01	0.9299350	0.9431312	20485.1	1.777300e-03	0.95	0.4332820	1.4286700
beta[3]	0.9999609	1.0000701	8.297890e-01	0.8244210	0.8183588	19377.5	1.786000e-03	0.95	0.3401960	1.3157000
beta[4]	0.9999213	1.0000235	1.292644e+00	1.2913000	1.3152501	20001.0	1.651400e-03	0.95	0.8301440	1.7430100
beta[5]	1.0000618	1.0004661	1.351957e+00	1.3503300	1.3614312	20001.0	1.633400e-03	0.95	0.8943980	1.7991700
beta[6]	0.9999274	1.0000129	1.462468e+00	1.4588100	1.4471899	19593.0	1.698500e-03	0.95	1.0010100	1.9280100
beta[7]	1.0000505	1.0005385	1.206497e+00	1.2047900	1.2026799	20001.0	1.594800e-03	0.95	0.7490150	1.6362400

beta[8]	0.9999174	0.9999526	1.248305e+00	1.2439600	1.2362896	20001.0	1.670800e-03	0.95	0.7881530	1.7107200
beta[9]	1.0005629	1.0022708	1.112530e+00	1.1098300	1.1096605	19600.7	1.650400e-03	0.95	0.6721490	1.5750900
beta[10]	1.0000765	1.0004299	6.293893e+00	6.2633000	6.2251229	16703.6	5.647600e-03	0.95	4.8641400	7.7122400
beta[11]	0.9999655	1.0000274	6.325073e+00	6.2950800	6.1809312	16335.2	5.757800e-03	0.95	4.9008100	7.7509200
beta[12]	0.9999349	0.9999816	5.551541e+00	5.5324400	5.5562870	18826.9	4.372100e-03	0.95	4.4223500	6.7538800
beta[13]	1.0001419	1.0005614	6.486567e+00	6.4547000	6.4040574	17256.6	5.464300e-03	0.95	5.1439000	7.9435600
beta[14]	1.0000426	1.0004383	6.187718e+00	6.1586200	6.1323587	15911.3	5.357100e-03	0.95	4.8672600	7.5094500
beta[15]	1.0007532	1.0022657	6.765743e+00	6.7321000	6.6552594	16365.3	5.871800e-03	0.95	5.3085600	8.2364100
beta[16]	0.9998749	0.9999185	6.004864e+00	5.9771400	5.8378836	17943.4	4.923500e-03	0.95	4.7285400	7.3088600
beta[17]	0.9999362	1.0000597	5.596921e+00	5.5720000	5.4908218	18417.9	4.411300e-03	0.95	4.4338300	6.7462900
beta[18]	0.9999332	1.0001331	5.662865e+00	5.6357700	5.5538499	17643.0	4.817400e-03	0.95	4.4278900	6.9285900
beta[19]	1.0000474	1.0004996	5.934032e+00	5.8994000	5.8071487	17586.0	4.941700e-03	0.95	4.6870800	7.2408600
beta[20]	1.0002022	1.0009824	6.069972e+00	6.0470200	6.0343594	17249.4	5.172400e-03	0.95	4.7684000	7.4263800
beta[21]	1.0003458	1.0003985	5.988763e+00	5.9623800	5.9186866	18080.3	4.738900e-03	0.95	4.7295700	7.2170400
beta[22]	1.0000188	1.0002710	6.910568e+00	6.8813700	6.8653244	16058.8	6.249700e-03	0.95	5.4392900	8.5343600
beta[23]	1.0000862	1.0005580	5.675828e+00	5.6559200	5.5834353	18649.6	4.300000e-03	0.95	4.5475200	6.8561800
beta[24]	1.0003938	1.0011888	4.988496e+00	4.9705500	4.9451108	18879.6	3.837200e-03	0.95	3.9819300	6.0316600
beta[25]	1.0012522	1.0049613	5.211679e+00	5.1899400	5.1290257	18844.8	3.897000e-03	0.95	4.1989900	6.2779000
beta[26]	1.0002414	1.0008484	5.166291e+00	5.1403400	5.0590686	19314.2	3.995400e-03	0.95	4.0646000	6.2481800
beta[27]	1.0002347	1.0006938	2.805583e+00	2.7763300	2.7212022	20001.0	3.569000e-03	0.95	1.8673000	3.8171800
beta[28]	1.0004257	1.0013932	4.179477e+00	4.1586900	4.0582418	18974.7	3.906700e-03	0.95	3.1213400	5.2133500
beta[29]	1.0000936	1.0005749	3.472293e+00	3.4472500	3.3899149	19594.0	3.284700e-03	0.95	2.6077700	4.4047500
beta[30]	1.0000066	1.0002837	3.389028e+00	3.3703100	3.3661671	20001.0	3.180100e-03	0.95	2.5182600	4.2705000
beta[31]	1.0001415	1.0005479	2.030067e+00	2.0197200	1.9994381	20001.0	2.260900e-03	0.95	1.4233500	2.6726900
beta[32]	1.0001389	1.0008539	2.891036e+00	2.8595900	2.6583750	19071.2	4.181100e-03	0.95	1.7794900	4.0324400
beta[33]	1.0001054	1.0005250	4.187727e+00	4.1566400	4.0280872	18452.2	4.816600e-03	0.95	2.9876800	5.5425500
beta[34]	1.0006074	1.0025133	4.788366e+00	4.7515100	4.6637448	17374.7	5.433400e-03	0.95	3.4224800	6.2102900
beta[35]	1.0000365	1.0002843	3.382622e+00	3.3597500	3.2312759	19154.0	3.863800e-03	0.95	2.3497700	4.4386900
beta[36]	1.0000125	1.0001266	3.095911e+00	3.0772400	3.0472318	18906.7	3.219700e-03	0.95	2.2419500	3.9704000
beta[37]	1.0003740	1.0011269	2.489322e+00	2.4621000	2.4573789	20641.7	3.715100e-03	0.95	1.4487900	3.5046700
beta[38]	1.0001721	1.0004564	2.626280e+00	2.6145900	2.5832756	19222.2	2.373900e-03	0.95	1.9835600	3.2684100
beta[39]	1.0001078	1.0007878	3.546026e+00	3.5269500	3.4515187	19466.7	3.241600e-03	0.95	2.6700100	4.4286700
beta[40]	1.0001358	1.0003357	3.249401e+00	3.2317800	3.2001724	19534.9	2.868900e-03	0.95	2.4769200	4.0382000
beta[41]	1.0003194	1.0014887	5.074862e+00	5.0401200	4.9755607	18192.2	5.050500e-03	0.95	3.7998700	6.4361800
beta[42]	1.0000260	1.0003555	3.462126e+00	3.4410100	3.3299612	19181.2	3.941800e-03	0.95	2.4435100	4.5590700
beta[43]	1.0002822	1.0012142	3.114910e+00	3.0950500	2.9413903	20001.0	3.590400e-03	0.95	2.1176800	4.0959300
beta[44]	1.0008510	1.0035067	5.021479e+00	4.9900200	4.8711039	16880.4	5.268500e-03	0.95	3.6848000	6.3657100
beta[45]	1.0000835	1.0006975	5.407239e+00	5.3693200	5.1373123	16978.5	5.671300e-03	0.95	3.9948700	6.8613900
beta[46]	1.0001354	1.0008819	3.322828e+00	3.3031200	3.2863129	19373.8	3.346400e-03	0.95	2.4507100	4.2675300

beta[47]	1.0001290	1.0007363	2.849271e+00	2.8329800	2.7781319	20001.0	2.925300e-03	0.95	2.0759300	3.6737600
beta[48]	0.9999179	1.0000297	3.640178e+00	3.6151300	3.6007728	20001.0	3.629800e-03	0.95	2.6849600	4.6677500
beta[49]	1.0003009	1.0010418	3.453190e+00	3.4352100	3.3821695	19333.5	3.189500e-03	0.95	2.5972900	4.3238600
beta[50]	0.9999801	1.0002731	3.559468e+00	3.5431100	3.4643390	19237.7	3.275300e-03	0.95	2.6962300	4.4544300
beta[51]	0.9999078	0.9999194	7.637167e+00	7.5801400	7.4394373	11894.5	8.888800e-03	0.95	5.7675000	9.5177200
beta[52]	1.0001340	1.0007035	2.686256e+00	2.6759200	2.6647228	20001.0	2.271900e-03	0.95	2.0532800	3.3137800
beta[53]	1.0000796	1.0005813	5.595808e+00	5.5552300	5.4581969	12982.7	6.579800e-03	0.95	4.1855200	7.0989600
beta[54]	1.0000573	1.0005255	4.508470e+00	4.4782800	4.3797049	19117.5	4.182600e-03	0.95	3.3991300	5.6585700
beta[55]	1.0008113	1.0030426	5.173986e+00	5.1432900	5.1181081	13814.8	5.906600e-03	0.95	3.8348900	6.5344500
beta[56]	1.0003566	1.0014922	3.558453e+00	3.5403500	3.5032527	19322.5	3.103800e-03	0.95	2.7236800	4.4042400
beta[57]	0.9999688	1.0000151	3.011195e+00	3.0044600	3.0011850	20001.0	2.295100e-03	0.95	2.3637300	3.6248000
beta[58]	1.0001935	1.0008025	3.756001e+00	3.7468800	3.7356272	20001.0	2.644800e-03	0.95	3.0071400	4.4794600
beta[59]	0.9999016	1.0000347	3.527248e+00	3.5097900	3.5039809	18750.0	3.119200e-03	0.95	2.7280700	4.3962800
beta[60]	0.9998984	0.9999877	3.896715e+00	3.8704400	3.7850270	18366.4	4.432600e-03	0.95	2.7419000	5.0799500
beta[61]	0.9999822	1.0002000	3.059470e+00	3.0451700	2.9965989	19447.9	2.774400e-03	0.95	2.3405500	3.8503500
beta[62]	1.0005707	1.0016630	4.334949e+00	4.3167000	4.3365481	19048.2	3.585500e-03	0.95	3.3828600	5.2964900
beta[63]	1.0003409	1.0016384	3.710967e+00	3.7005700	3.7103940	19583.7	2.669400e-03	0.95	2.9972200	4.4641400
beta[64]	1.0002563	1.0010563	4.336675e+00	4.3196600	4.3101260	19788.7	3.203100e-03	0.95	3.4731400	5.2127700
thresh[1,1]	NaN	NaN	5.000000e-01	0.5000000	0.4996355	0.0	NaN	0.95	0.5000000	0.5000000
thresh[2,1]	NaN	NaN	5.000000e-01	0.5000000	0.4996355	0.0	NaN	0.95	0.5000000	0.5000000
thresh[3,1]	NaN	NaN	5.000000e-01	0.5000000	0.4996355	0.0	NaN	0.95	0.5000000	0.5000000
thresh[4,1]	NaN	NaN	5.000000e-01	0.5000000	0.4996355	0.0	NaN	0.95	0.5000000	0.5000000
thresh[5,1]	NaN	NaN	5.000000e-01	0.5000000	0.4996355	0.0	NaN	0.95	0.5000000	0.5000000
thresh[6,1]	NaN	NaN	5.000000e-01	0.5000000	0.4996355	0.0	NaN	0.95	0.5000000	0.5000000
thresh[7,1]	NaN	NaN	5.000000e-01	0.5000000	0.4996355	0.0	NaN	0.95	0.5000000	0.5000000
thresh[8,1]	NaN	NaN	5.000000e-01	0.5000000	0.4996355	0.0	NaN	0.95	0.5000000	0.5000000
thresh[9,1]	NaN	NaN	5.000000e-01	0.5000000	0.4996355	0.0	NaN	0.95	0.5000000	0.5000000
thresh[10,1]	NaN	NaN	5.000000e-01	0.5000000	0.4996355	0.0	NaN	0.95	0.5000000	0.5000000
thresh[11,1]	NaN	NaN	5.000000e-01	0.5000000	0.4996355	0.0	NaN	0.95	0.5000000	0.5000000
thresh[12,1]	NaN	NaN	5.000000e-01	0.5000000	0.4996355	0.0	NaN	0.95	0.5000000	0.5000000
thresh[13,1]	NaN	NaN	5.000000e-01	0.5000000	0.4996355	0.0	NaN	0.95	0.5000000	0.5000000
thresh[14,1]	NaN	NaN	5.000000e-01	0.5000000	0.4996355	0.0	NaN	0.95	0.5000000	0.5000000
thresh[15,1]	NaN	NaN	5.000000e-01	0.5000000	0.4996355	0.0	NaN	0.95	0.5000000	0.5000000
thresh[16,1]	NaN	NaN	5.000000e-01	0.5000000	0.4996355	0.0	NaN	0.95	0.5000000	0.5000000
thresh[17,1]	NaN	NaN	5.000000e-01	0.5000000	0.4996355	0.0	NaN	0.95	0.5000000	0.5000000
thresh[18,1]	NaN	NaN	5.000000e-01	0.5000000	0.4996355	0.0	NaN	0.95	0.5000000	0.5000000
thresh[19,1]	NaN	NaN	5.000000e-01	0.5000000	0.4996355	0.0	NaN	0.95	0.5000000	0.5000000
thresh[20,1]	NaN	NaN	5.000000e-01	0.5000000	0.4996355	0.0	NaN	0.95	0.5000000	0.5000000
thresh[21,1]	NaN	NaN	5.000000e-01	0.5000000	0.4996355	0.0	NaN	0.95	0.5000000	0.5000000

thresh[22,1]	NaN	NaN	5.000000e-01	0.5000000	0.4996355	0.0	NaN	0.95	0.5000000	0.5000000
thresh[23,1]	NaN	NaN	5.000000e-01	0.5000000	0.4996355	0.0	NaN	0.95	0.5000000	0.5000000
thresh[24,1]	NaN	NaN	5.000000e-01	0.5000000	0.4996355	0.0	NaN	0.95	0.5000000	0.5000000
thresh[25,1]	NaN	NaN	5.000000e-01	0.5000000	0.4996355	0.0	NaN	0.95	0.5000000	0.5000000
thresh[26,1]	NaN	NaN	5.000000e-01	0.5000000	0.4996355	0.0	NaN	0.95	0.5000000	0.5000000
thresh[27,1]	NaN	NaN	5.000000e-01	0.5000000	0.4996355	0.0	NaN	0.95	0.5000000	0.5000000
thresh[28,1]	NaN	NaN	5.000000e-01	0.5000000	0.4996355	0.0	NaN	0.95	0.5000000	0.5000000
thresh[29,1]	NaN	NaN	5.000000e-01	0.5000000	0.4996355	0.0	NaN	0.95	0.5000000	0.5000000
thresh[30,1]	NaN	NaN	5.000000e-01	0.5000000	0.4996355	0.0	NaN	0.95	0.5000000	0.5000000
thresh[31,1]	NaN	NaN	5.000000e-01	0.5000000	0.4996355	0.0	NaN	0.95	0.5000000	0.5000000
thresh[32,1]	NaN	NaN	5.000000e-01	0.5000000	0.4996355	0.0	NaN	0.95	0.5000000	0.5000000
thresh[33,1]	NaN	NaN	5.000000e-01	0.5000000	0.4996355	0.0	NaN	0.95	0.5000000	0.5000000
thresh[34,1]	NaN	NaN	5.000000e-01	0.5000000	0.4996355	0.0	NaN	0.95	0.5000000	0.5000000
thresh[35,1]	NaN	NaN	5.000000e-01	0.5000000	0.4996355	0.0	NaN	0.95	0.5000000	0.5000000
thresh[36,1]	NaN	NaN	5.000000e-01	0.5000000	0.4996355	0.0	NaN	0.95	0.5000000	0.5000000
thresh[37,1]	NaN	NaN	5.000000e-01	0.5000000	0.4996355	0.0	NaN	0.95	0.5000000	0.5000000
thresh[38,1]	NaN	NaN	5.000000e-01	0.5000000	0.4996355	0.0	NaN	0.95	0.5000000	0.5000000
thresh[39,1]	NaN	NaN	5.000000e-01	0.5000000	0.4996355	0.0	NaN	0.95	0.5000000	0.5000000
thresh[40,1]	NaN	NaN	5.000000e-01	0.5000000	0.4996355	0.0	NaN	0.95	0.5000000	0.5000000
thresh[41,1]	NaN	NaN	5.000000e-01	0.5000000	0.4996355	0.0	NaN	0.95	0.5000000	0.5000000
thresh[42,1]	NaN	NaN	5.000000e-01	0.5000000	0.4996355	0.0	NaN	0.95	0.5000000	0.5000000
thresh[43,1]	NaN	NaN	5.000000e-01	0.5000000	0.4996355	0.0	NaN	0.95	0.5000000	0.5000000
thresh[44,1]	NaN	NaN	5.000000e-01	0.5000000	0.4996355	0.0	NaN	0.95	0.5000000	0.5000000
thresh[45,1]	NaN	NaN	5.000000e-01	0.5000000	0.4996355	0.0	NaN	0.95	0.5000000	0.5000000
thresh[46,1]	NaN	NaN	5.000000e-01	0.5000000	0.4996355	0.0	NaN	0.95	0.5000000	0.5000000
thresh[47,1]	NaN	NaN	5.000000e-01	0.5000000	0.4996355	0.0	NaN	0.95	0.5000000	0.5000000
thresh[48,1]	NaN	NaN	5.000000e-01	0.5000000	0.4996355	0.0	NaN	0.95	0.5000000	0.5000000
thresh[49,1]	NaN	NaN	5.000000e-01	0.5000000	0.4996355	0.0	NaN	0.95	0.5000000	0.5000000
thresh[50,1]	NaN	NaN	5.000000e-01	0.5000000	0.4996355	0.0	NaN	0.95	0.5000000	0.5000000
thresh[51,1]	NaN	NaN	5.000000e-01	0.5000000	0.4996355	0.0	NaN	0.95	0.5000000	0.5000000
thresh[52,1]	NaN	NaN	5.000000e-01	0.5000000	0.4996355	0.0	NaN	0.95	0.5000000	0.5000000
thresh[53,1]	NaN	NaN	5.000000e-01	0.5000000	0.4996355	0.0	NaN	0.95	0.5000000	0.5000000
thresh[54,1]	NaN	NaN	5.000000e-01	0.5000000	0.4996355	0.0	NaN	0.95	0.5000000	0.5000000
thresh[55,1]	NaN	NaN	5.000000e-01	0.5000000	0.4996355	0.0	NaN	0.95	0.5000000	0.5000000
thresh[56,1]	NaN	NaN	5.000000e-01	0.5000000	0.4996355	0.0	NaN	0.95	0.5000000	0.5000000
thresh[57,1]	NaN	NaN	5.000000e-01	0.5000000	0.4996355	0.0	NaN	0.95	0.5000000	0.5000000
thresh[58,1]	NaN	NaN	5.000000e-01	0.5000000	0.4996355	0.0	NaN	0.95	0.5000000	0.5000000
thresh[59,1]	NaN	NaN	5.000000e-01	0.5000000	0.4996355	0.0	NaN	0.95	0.5000000	0.5000000
thresh[60,1]	NaN	NaN	5.000000e-01	0.5000000	0.4996355	0.0	NaN	0.95	0.5000000	0.5000000

thresh[61,1]	NaN	NaN	5.000000e-01	0.5000000	0.4996355	0.0	NaN	0.95	0.5000000	0.5000000
thresh[62,1]	NaN	NaN	5.000000e-01	0.5000000	0.4996355	0.0	NaN	0.95	0.5000000	0.5000000
thresh[63,1]	NaN	NaN	5.000000e-01	0.5000000	0.4996355	0.0	NaN	0.95	0.5000000	0.5000000
thresh[64,1]	NaN	NaN	5.000000e-01	0.5000000	0.4996355	0.0	NaN	0.95	0.5000000	0.5000000
thresh[10,2]	1.0000566	1.0004797	3.055947e+00	3.0480100	3.0665420	18328.8	2.045500e-03	0.95	2.5151700	3.5968300
thresh[11,2]	1.0000283	1.0001678	3.062217e+00	3.0497400	3.0089604	17903.1	2.129000e-03	0.95	2.5148000	3.6263300
thresh[12,2]	0.9999334	1.0000437	2.935483e+00	2.9268800	2.8822972	20001.0	1.816900e-03	0.95	2.4374500	3.4395800
thresh[13,2]	1.0002394	1.0008037	3.125945e+00	3.1137500	3.1225171	19319.6	2.162500e-03	0.95	2.5426400	3.7141900
thresh[14,2]	1.0004849	1.0017269	2.553061e+00	2.5403400	2.5272613	19375.2	1.930200e-03	0.95	2.0602000	3.1067700
thresh[15,2]	1.0002769	1.0010518	2.452640e+00	2.4423000	2.4485530	18445.1	1.960800e-03	0.95	1.9508000	2.9868700
thresh[16,2]	1.0004175	1.0013457	2.237705e+00	2.2288700	2.2051002	20001.0	1.695900e-03	0.95	1.7835100	2.7166900
thresh[17,2]	1.0003767	1.0006264	2.234608e+00	2.2277100	2.2271369	19602.2	1.566900e-03	0.95	1.8255900	2.6785300
thresh[18,2]	1.0000989	1.0007524	2.373481e+00	2.3665500	2.3658980	19135.3	1.544500e-03	0.95	1.9614100	2.7969000
thresh[19,2]	0.9999632	1.0002242	2.482046e+00	2.4732800	2.4964434	19876.1	1.692000e-03	0.95	2.0344900	2.9560400
thresh[20,2]	1.0000871	1.0003932	2.566053e+00	2.5597800	2.5657388	20215.8	1.759000e-03	0.95	2.1065500	3.0781900
thresh[21,2]	1.0000621	1.0000719	3.075506e+00	3.0614300	3.0474105	19336.3	2.204600e-03	0.95	2.4706100	3.6663000
thresh[22,2]	1.0000545	1.0003798	3.173775e+00	3.1611900	3.1288038	18461.7	2.374600e-03	0.95	2.5560400	3.8155200
thresh[23,2]	1.0013751	1.0040512	2.928511e+00	2.9163200	2.8415695	20001.0	2.114600e-03	0.95	2.3637400	3.5418800
thresh[24,2]	1.0002199	1.0005512	2.172469e+00	2.1671800	2.2018648	20001.0	1.401200e-03	0.95	1.8010400	2.5738900
thresh[25,2]	1.0011183	1.0030671	2.308898e+00	2.2975500	2.2655999	20001.0	1.627800e-03	0.95	1.8803400	2.7766600
thresh[26,2]	1.0003225	1.0009514	2.790907e+00	2.7793300	2.7582830	20001.0	1.868500e-03	0.95	2.2966400	3.3271900
thresh[51,2]	0.9999576	1.0000983	6.693479e+00	6.6453400	6.5291125	12213.8	8.000200e-03	0.95	5.0091600	8.4325600
thresh[53,2]	1.0002700	1.0012870	6.280984e+00	6.2432000	6.1882203	12610.0	6.727500e-03	0.95	4.8581400	7.8089800
thresh[55,2]	1.0008971	1.0031224	5.903707e+00	5.8710000	5.8675703	13567.1	5.875600e-03	0.95	4.6389200	7.3026800
thresh[56,2]	1.0002524	1.0012962	4.307170e+00	4.2941000	4.2452258	20001.0	2.658800e-03	0.95	3.5953800	5.0655500
thresh[57,2]	0.9998865	0.9999439	3.170123e+00	3.1566800	3.1081899	21914.4	1.873100e-03	0.95	2.6529200	3.7249900
thresh[58,2]	1.0000098	1.0004272	2.653556e+00	2.6429000	2.6102906	20001.0	1.788300e-03	0.95	2.1731000	3.1629700
thresh[62,2]	1.0002603	1.0005868	2.774795e+00	2.7685100	2.7719706	20001.0	1.580300e-03	0.95	2.3539400	3.2267400
thresh[63,2]	1.0007560	1.0027213	2.378135e+00	2.3682800	2.3647068	19792.9	1.532000e-03	0.95	1.9603000	2.7988900
thresh[64,2]	1.0004014	1.0013948	2.909417e+00	2.9015700	2.8369815	20001.0	1.801500e-03	0.95	2.4205000	3.4126800
thresh_age[1,1]	0.9999224	1.0000842	3.018944e+01	30.3073000	30.4620597	20001.0	2.065120e-02	0.95	24.5262000	36.0143000
thresh_age[2,1]	1.2909672	1.3007386	3.116654e+17	111.4830000	53.7027823	20001.0	3.116654e+17	0.95	73.6210000	208.0360000
thresh_age[3,1]	1.2909672	1.3007386	6.959522e+21	121.7310000	53.5497351	20001.0	6.959502e+21	0.95	76.3424000	282.4520000
thresh_age[4,1]	1.0002650	1.0012815	4.888649e+01	48.7612000	48.8592990	20001.0	2.770920e-02	0.95	41.2355000	56.6068000
thresh_age[5,1]	1.0001640	1.0007573	3.958382e+01	39.6770000	39.8921378	20001.0	2.367320e-02	0.95	32.9894000	46.1337000
thresh_age[6,1]	1.0004508	1.0006017	3.928772e+01	39.3328000	39.6239644	20001.0	2.230060e-02	0.95	33.0524000	45.4411000
thresh_age[7,1]	1.0001014	1.0003406	4.020975e+01	40.2989000	40.5968381	20001.0	2.633280e-02	0.95	32.8466000	47.5525000
thresh_age[8,1]	1.0003835	1.0010474	5.926011e+01	58.8156000	57.9679985	20652.4	3.712190e-02	0.95	49.7105000	70.0704000
thresh_age[9,1]	1.0196127	1.0211623	6.261795e+01	61.8048000	61.1975403	20001.0	5.010120e-02	0.95	50.6058000	75.6365000

thresh_age[10,1]	1.0003440	1.0007883	5.276479e+01	52.7522000	52.5736844	18851.1	1.449810e-02	0.95	48.9282000	56.7125000
thresh_age[11,1]	1.0004335	1.0010324	5.094880e+01	50.9429000	51.1073905	18849.1	1.408000e-02	0.95	47.2358000	54.8265000
thresh_age[12,1]	1.0002383	1.0008662	4.883647e+01	48.8205000	48.6284854	18329.5	1.437740e-02	0.95	45.0388000	52.6239000
thresh_age[13,1]	0.9999437	0.9999489	4.794512e+01	47.9339000	47.8909080	18892.2	1.361870e-02	0.95	44.2984000	51.5895000
thresh_age[14,1]	1.0007371	1.0030784	4.723920e+01	47.2279000	47.1334060	18259.2	1.400520e-02	0.95	43.5662000	50.9503000
thresh_age[15,1]	1.0000552	1.0003386	4.861709e+01	48.6204000	48.5882153	19140.9	1.344970e-02	0.95	44.9959000	52.2513000
thresh_age[16,1]	1.0004997	1.0019914	4.824530e+01	48.2561000	48.2885487	19113.3	1.377270e-02	0.95	44.5793000	52.0294000
thresh_age[17,1]	1.0000696	1.0005711	4.838029e+01	48.3653000	48.0893252	18431.0	1.428330e-02	0.95	44.6191000	52.1956000
thresh_age[18,1]	0.9999234	0.9999432	5.356107e+01	53.5660000	53.4370635	18253.3	1.488730e-02	0.95	49.6573000	57.5174000
thresh_age[19,1]	1.0008388	1.0026484	5.046696e+01	50.4805000	50.7911071	19597.9	1.376320e-02	0.95	46.6955000	54.2430000
thresh_age[20,1]	1.0003912	1.0016155	5.027011e+01	50.2825000	50.3387307	18427.1	1.416580e-02	0.95	46.5090000	54.0885000
thresh_age[21,1]	1.0002239	1.0006961	4.399003e+01	43.9882000	44.1232693	17770.7	1.362270e-02	0.95	40.3423000	47.4502000
thresh_age[22,1]	1.0003088	1.0010171	4.893121e+01	48.9345000	49.0529048	18839.0	1.363130e-02	0.95	45.3105000	52.7096000
thresh_age[23,1]	1.0010619	1.0036310	4.228343e+01	42.2639000	42.2464667	18215.1	1.322070e-02	0.95	38.8426000	45.7888000
thresh_age[24,1]	0.9999770	1.0001492	4.845789e+01	48.4616000	48.5593128	19390.1	1.411340e-02	0.95	44.5332000	52.1973000
thresh_age[25,1]	1.0000523	1.0003952	4.389607e+01	43.8919000	44.1562209	19191.1	1.349930e-02	0.95	40.2181000	47.4793000
thresh_age[26,1]	1.0002074	1.0009516	4.529506e+01	45.2709000	45.0493829	18942.3	1.357790e-02	0.95	41.6963000	48.9025000
thresh_age[27,1]	1.0000440	1.0003032	2.342390e+01	23.5038000	23.4998110	19983.7	1.277120e-02	0.95	19.7446000	26.8224000
thresh_age[28,1]	1.0003200	1.0014995	5.018811e+01	50.1846000	50.1382149	19536.1	1.492390e-02	0.95	46.1683000	54.2545000
thresh_age[29,1]	0.9998837	0.9998886	5.506441e+01	55.0407000	54.9608098	19167.0	1.707730e-02	0.95	50.2939000	59.5699000
thresh_age[30,1]	1.0001269	1.0005984	5.590004e+01	55.8604000	55.8182721	19317.0	1.723950e-02	0.95	51.2014000	60.6265000
thresh_age[31,1]	1.0001287	1.0007258	6.492477e+01	64.6257000	63.8344857	20001.0	2.795980e-02	0.95	57.3892000	72.7560000
thresh_age[32,1]	1.0002746	1.0005552	8.485748e+01	83.9582000	82.9557799	20001.0	4.458270e-02	0.95	74.2284000	97.5935000
thresh_age[33,1]	1.0000561	1.0004609	6.789721e+01	67.8243000	67.7674192	20001.0	1.882920e-02	0.95	62.8870000	73.3462000
thresh_age[34,1]	1.0002457	1.0004102	6.480012e+01	64.7890000	64.7710498	20001.0	1.681080e-02	0.95	60.0016000	69.3933000
thresh_age[35,1]	1.0004665	1.0021180	6.783662e+01	67.7290000	67.4503477	20001.0	2.080180e-02	0.95	62.3306000	73.8118000
thresh_age[36,1]	1.0009623	1.0035763	6.099070e+01	60.9379000	60.8390006	20001.0	1.909220e-02	0.95	55.9003000	66.3987000
thresh_age[37,1]	1.0000880	1.0001406	9.025647e+01	88.7286000	86.8561033	20409.3	6.205550e-02	0.95	76.3358000	108.0720000
thresh_age[38,1]	1.0000216	1.0003025	4.853091e+01	48.5200000	48.5567192	18544.8	1.783630e-02	0.95	43.7890000	53.3176000
thresh_age[39,1]	1.0002039	1.0010951	5.270489e+01	52.6911000	52.7446364	19118.7	1.670130e-02	0.95	48.2447000	57.2389000
thresh_age[40,1]	1.0006699	1.0019218	4.369274e+01	43.6842000	43.6601736	19503.2	1.515720e-02	0.95	39.5686000	47.8665000
thresh_age[41,1]	1.0000618	1.0005721	5.358162e+01	53.5753000	53.5216868	19121.9	1.496160e-02	0.95	49.4225000	57.5017000
thresh_age[42,1]	1.0000342	1.0004940	6.795995e+01	67.8799000	67.9987915	20001.0	2.050730e-02	0.95	62.3178000	73.7567000
thresh_age[43,1]	1.0000959	1.0007465	7.004534e+01	69.9128000	69.1855822	20001.0	2.359620e-02	0.95	63.4508000	76.4545000
thresh_age[44,1]	0.9999356	1.0000787	5.746670e+01	57.4611000	57.5622001	20878.2	1.484660e-02	0.95	53.0148000	61.4882000
thresh_age[45,1]	1.0001899	1.0010941	5.483906e+01	54.8570000	54.9174238	18809.3	1.509170e-02	0.95	50.8015000	58.9645000
thresh_age[46,1]	0.9999898	1.0000696	5.929565e+01	59.2837000	59.5224150	20001.0	1.806470e-02	0.95	54.3598000	64.3775000
thresh_age[47,1]	1.0001124	1.0002867	6.167133e+01	61.5993000	61.5473912	19911.0	2.037480e-02	0.95	56.0063000	67.2961000
thresh_age[48,1]	1.0003594	1.0006893	6.086436e+01	60.8525000	60.9089830	20001.0	1.783370e-02	0.95	56.0640000	65.9699000

thresh_age[49,1]	1.0001850	1.0004982	5.340098e+01	53.3877000	53.3923221	19065.8	1.684970e-02	0.95	48.9555000	58.0727000
thresh_age[50,1]	1.0001592	1.0007909	5.247983e+01	52.4769000	52.6448466	19539.7	1.627530e-02	0.95	48.0582000	56.9683000
thresh_age[51,1]	1.0007856	1.0029400	2.495308e+01	24.9159000	24.8188060	18750.0	8.176500e-03	0.95	22.7893000	27.1554000
thresh_age[52,1]	1.0000267	1.0001955	4.650559e+01	46.4771000	46.1210726	19559.4	1.659840e-02	0.95	41.9555000	51.0611000
thresh_age[53,1]	1.0004845	1.0020521	2.560582e+01	25.5855000	25.6361677	18375.2	8.879300e-03	0.95	23.1938000	27.9307000
thresh_age[54,1]	1.0002196	1.0010404	4.730472e+01	47.2828000	46.9195862	18880.3	1.434940e-02	0.95	43.4071000	51.1478000
thresh_age[55,1]	1.0000568	1.0002217	2.680109e+01	26.7707000	26.6654794	18388.1	9.776400e-03	0.95	24.2928000	29.4090000
thresh_age[56,1]	1.0002118	1.0011058	2.892328e+01	28.8971000	28.7614244	18586.0	1.179120e-02	0.95	25.7366000	32.0508000
thresh_age[57,1]	1.0000372	1.0004404	2.544108e+01	25.4340000	25.4005574	18557.4	1.226110e-02	0.95	22.2158000	28.7759000
thresh_age[58,1]	1.0000562	1.0004463	3.206062e+01	32.0537000	32.1327844	18778.5	1.179350e-02	0.95	28.9497000	35.2727000
thresh_age[59,1]	1.0000155	1.0003645	4.531462e+01	45.3027000	45.2842675	18967.3	1.507900e-02	0.95	41.1049000	49.2442000
thresh_age[60,1]	1.0002240	1.0002752	6.692894e+01	66.8832000	67.1903313	20001.0	1.891710e-02	0.95	61.7627000	72.2661000
thresh_age[61,1]	1.0002183	1.0011973	5.074385e+01	50.7258000	50.7682558	20675.3	1.631050e-02	0.95	46.1567000	55.2968000
thresh_age[62,1]	1.0001572	1.0009820	5.013910e+01	50.1243000	49.9724973	18603.9	1.539960e-02	0.95	46.0626000	54.3051000
thresh_age[63,1]	1.0000849	1.0006881	3.690301e+01	36.8788000	36.7352835	18687.2	1.308450e-02	0.95	33.4908000	40.4792000
thresh_age[64,1]	1.0001277	1.0008723	4.082235e+01	40.7870000	40.5622248	18389.6	1.357850e-02	0.95	37.2536000	44.4255000
thresh_age[10,2]	1.0001787	1.0002906	7.937124e+01	79.3319000	79.5025517	20001.0	2.005990e-02	0.95	74.0023000	85.1198000
thresh_age[11,2]	1.0000308	1.0003545	7.654180e+01	76.5047000	76.1537442	20001.0	1.891110e-02	0.95	71.4608000	81.8880000
thresh_age[12,2]	1.0001101	1.0006978	7.587593e+01	75.8459000	76.4197782	19263.7	1.973350e-02	0.95	70.4463000	81.2499000
thresh_age[13,2]	1.0001135	1.0007422	7.196943e+01	71.9700000	72.2706582	20001.0	1.695360e-02	0.95	67.2647000	76.6788000
thresh_age[14,2]	1.0000023	1.0001868	6.587739e+01	65.8879000	65.9044293	20001.0	1.524660e-02	0.95	61.6382000	70.0527000
thresh_age[15,2]	1.0002554	1.0010882	6.493260e+01	64.9322000	65.0219302	20001.0	1.481790e-02	0.95	60.7959000	68.9605000
thresh_age[16,2]	1.0000536	1.0001935	6.448731e+01	64.4919000	64.4101974	20001.0	1.514470e-02	0.95	60.3561000	68.7389000
thresh_age[17,2]	1.0001131	1.0001486	6.603270e+01	66.0162000	66.0212011	20001.0	1.594570e-02	0.95	61.7186000	70.5241000
thresh_age[18,2]	1.0001336	1.0005193	7.471213e+01	74.6698000	74.5320929	20001.0	1.893370e-02	0.95	69.4876000	79.9736000
thresh_age[19,2]	1.0002651	1.0011147	7.057850e+01	70.5727000	70.4162028	20001.0	1.709180e-02	0.95	65.9339000	75.3967000
thresh_age[20,2]	1.0001472	1.0008768	7.074560e+01	70.7317000	70.4105054	20001.0	1.671990e-02	0.95	66.1199000	75.3580000
thresh_age[21,2]	1.0002360	1.0005559	6.768264e+01	67.6818000	67.7554592	20001.0	1.602360e-02	0.95	63.3129000	72.1882000
thresh_age[22,2]	1.0001501	1.0005715	7.212702e+01	72.1448000	72.2793060	20001.0	1.653870e-02	0.95	67.7116000	76.8197000
thresh_age[23,2]	0.9999310	1.0001137	6.489441e+01	64.8933000	64.9628264	20001.0	1.546270e-02	0.95	60.5388000	69.1654000
thresh_age[24,2]	0.9998966	0.9999773	6.786161e+01	67.8500000	68.0376327	20001.0	1.719220e-02	0.95	63.0857000	72.6571000
thresh_age[25,2]	1.0000116	1.0001271	6.216195e+01	62.1631000	62.1813925	19578.9	1.567010e-02	0.95	57.8195000	66.3495000
thresh_age[26,2]	1.0003398	1.0016185	7.065772e+01	70.6442000	70.8065784	20001.0	1.758540e-02	0.95	65.8145000	75.5757000
thresh_age[51,2]	1.0001766	1.0007122	5.609577e+01	56.1064000	56.3489488	18748.3	1.393540e-02	0.95	52.3816000	59.8580000
thresh_age[53,2]	1.0000725	1.0001507	7.202583e+01	71.9967000	72.4233487	20001.0	1.804290e-02	0.95	67.1266000	77.1124000
thresh_age[55,2]	1.0002438	1.0007399	7.630267e+01	76.2269000	75.8661969	20001.0	2.025070e-02	0.95	70.7400000	81.9000000
thresh_age[56,2]	0.9999584	0.9999625	8.472145e+01	84.4623000	84.0265652	19278.9	3.176260e-02	0.95	76.2283000	93.5161000
thresh_age[57,2]	0.9999224	1.0000466	6.186776e+01	61.8142000	61.5412771	20001.0	1.927010e-02	0.95	56.4630000	67.1357000
thresh_age[58,2]	1.0001590	1.0009621	5.691906e+01	56.9033000	57.0574352	20001.0	1.621760e-02	0.95	52.4241000	61.3881000

thresh_age[62,2]	1.0005284	1.0013808	8.505313e+01	84.8398000	84.2633533	20001.0	2.785030e-02	0.95	77.4298000	92.7552000
thresh_age[63,2]	1.0004962	1.0021976	6.128818e+01	61.2350000	60.9934188	20001.0	1.760880e-02	0.95	56.4984000	66.2788000
thresh_age[64,2]	0.9999720	1.0002980	7.127200e+01	71.2071000	70.6736514	20001.0	1.978970e-02	0.95	65.8635000	76.8197000
age.s[1]	0.9999342	1.0001201	2.101594e+01	20.6573000	19.4126060	19186.2	1.148890e-02	0.95	19.0001000	24.1139000
age.s[2]	1.0002058	1.0010837	4.519086e+01	45.1547000	44.9855128	19019.1	1.643220e-02	0.95	40.7510000	49.6209000
age.s[3]	1.0000073	1.0003232	3.313067e+01	33.1119000	32.9116123	18415.5	1.946560e-02	0.95	28.0845000	38.3642000
age.s[4]	0.9999793	1.0002564	7.045589e+01	70.3730000	70.0052193	20001.0	2.286050e-02	0.95	64.2058000	76.8050000
age.s[5]	1.0005651	1.0016513	8.889733e+01	88.8318000	89.1600665	20001.0	3.375490e-02	0.95	80.1174000	98.4840000
age.s[6]	1.0002075	1.0010616	6.859042e+01	68.5289000	68.7257286	20001.0	2.228310e-02	0.95	62.2584000	74.6603000
age.s[7]	1.0002459	1.0010107	3.238608e+01	32.3591000	32.5052427	18769.1	1.923460e-02	0.95	27.2776000	37.5849000
age.s[8]	1.0014136	1.0054262	6.916735e+01	69.0951000	68.9236587	19577.4	2.260340e-02	0.95	63.1846000	75.4889000
age.s[9]	1.0001619	1.0005829	6.788526e+01	67.8417000	68.0048457	20001.0	2.180180e-02	0.95	61.9588000	74.0546000
age.s[10]	1.0000364	1.0003071	3.894039e+01	38.9372000	39.0789595	18440.9	1.741210e-02	0.95	34.3394000	43.6369000
age.s[11]	1.0004886	1.0011862	3.153749e+01	31.5127000	31.5398608	20001.0	1.872240e-02	0.95	26.3525000	36.6332000
age.s[12]	1.0008581	1.0023628	5.227781e+01	52.2326000	51.7665918	18269.1	1.752850e-02	0.95	47.6437000	56.9719000
age.s[13]	1.0005619	1.0017834	4.262350e+01	42.6014000	42.6871986	19139.8	1.668110e-02	0.95	38.1539000	47.1730000
age.s[14]	1.0003043	1.0011901	2.918212e+01	29.1139000	28.9982688	20001.0	1.816070e-02	0.95	24.2537000	34.3125000
age.s[15]	0.9999118	0.9999815	7.921629e+01	79.0802000	78.5886146	19001.0	2.897950e-02	0.95	71.7435000	87.3655000
age.s[16]	1.0000251	1.0001204	7.764182e+01	77.5343000	77.0412210	20001.0	2.621700e-02	0.95	70.5687000	84.9682000
age.s[17]	0.9999309	1.0000141	7.729273e+01	77.1530000	76.9898809	20001.0	2.707000e-02	0.95	69.9304000	84.9708000
age.s[18]	0.9999246	1.0000155	3.714962e+01	37.1381000	36.6480627	19879.4	1.735130e-02	0.95	32.5353000	42.1598000
age.s[19]	1.0000386	1.0002397	9.352107e+01	94.0087000	94.6063740	20001.0	2.914060e-02	0.95	86.0683000	99.9983000
age.s[20]	1.0000427	1.0003464	7.074171e+01	70.6791000	70.6639939	19502.6	2.316570e-02	0.95	64.5870000	77.1394000
age.s[21]	1.0001388	1.0008354	4.680431e+01	46.7603000	46.3507776	18824.0	1.645750e-02	0.95	42.4714000	51.2666000
age.s[22]	0.9999572	0.9999987	6.584600e+01	65.7775000	65.4352251	20001.0	2.046670e-02	0.95	60.2660000	71.5389000
age.s[23]	1.0008305	1.0031844	2.849905e+01	28.4233000	28.0366721	19207.7	1.863180e-02	0.95	23.5738000	33.5887000
age.s[24]	1.0002971	1.0010797	2.055850e+01	20.2086000	19.3118235	20001.0	9.419100e-03	0.95	19.0000000	23.1853000
age.s[25]	0.9999536	1.0001038	6.013277e+01	60.0865000	60.0804717	20001.0	1.909050e-02	0.95	54.7741000	65.3437000
age.s[26]	0.9999812	1.0002864	8.758596e+01	87.5307000	87.0917467	20001.0	3.343930e-02	0.95	78.8097000	97.2558000
age.s[27]	1.0001814	1.0010687	7.584791e+01	75.7654000	75.4809576	20001.0	2.551850e-02	0.95	68.8665000	83.0359000
age.s[28]	1.0002053	1.0007464	4.166743e+01	41.6464000	41.8283366	18402.8	1.650840e-02	0.95	37.2335000	46.0479000
age.s[29]	1.0003805	1.0014774	5.912048e+01	59.0518000	58.8766794	19546.6	1.888130e-02	0.95	54.0015000	64.3087000
age.s[30]	1.0003643	1.0012284	3.560027e+01	35.5837000	35.3171779	20001.0	1.801570e-02	0.95	30.6338000	40.6197000
age.s[31]	1.0000604	1.0006069	7.085999e+01	70.8120000	70.9479047	20001.0	2.302380e-02	0.95	64.8117000	77.4674000
age.s[32]	1.0000368	1.0004613	7.304413e+01	72.9433000	72.6258954	20001.0	2.411640e-02	0.95	66.3492000	79.6989000
age.s[33]	1.0001005	1.0003232	9.235073e+01	92.6681000	93.6690417	20001.0	3.140080e-02	0.95	84.4762000	99.9978000
age.s[34]	1.0002414	1.0009635	2.159826e+01	21.2706000	20.0097762	19121.9	1.323780e-02	0.95	19.0003000	25.0393000
age.s[35]	1.0006598	1.0026207	4.197016e+01	41.9353000	41.8023893	19379.3	1.656550e-02	0.95	37.5614000	46.5456000
age.s[36]	1.0000352	1.0005072	8.708633e+01	86.9701000	86.8214759	20001.0	3.305020e-02	0.95	78.3714000	96.6158000

age.s[37]	1.0003306	1.0013594	8.232621e+01	82.2136000	82.2418313	20001.0	3.035910e-02	0.95	74.0250000	90.7791000
age.s[38]	1.0010964	1.0037489	2.055824e+01	20.2053000	19.2996391	20001.0	9.447400e-03	0.95	19.0000000	23.2395000
age.s[39]	1.0001489	1.0009384	8.668675e+01	86.5114000	85.9699586	19562.1	3.400480e-02	0.95	77.7767000	96.3987000
age.s[40]	1.0000947	1.0007477	9.283925e+01	93.2067000	93.1067222	20001.0	3.041260e-02	0.95	85.1334000	99.9942000
age.s[41]	1.0002056	1.0011300	5.775072e+01	57.7312000	57.8590799	19569.5	1.836000e-02	0.95	52.7698000	62.8422000
age.s[42]	1.0000748	1.0003696	2.101322e+01	20.6436000	19.4323287	20001.0	1.124360e-02	0.95	19.0001000	24.1108000
age.s[43]	0.9999043	0.9999606	3.045544e+01	30.4170000	30.6958995	19079.0	1.942230e-02	0.95	25.2271000	35.6715000
age.s[44]	1.0000133	1.0001089	5.388331e+01	53.8480000	54.1778371	19342.6	1.774140e-02	0.95	49.2039000	58.8885000
age.s[45]	0.9998900	0.9999526	7.856791e+01	78.4619000	78.5047567	20001.0	2.751540e-02	0.95	71.0459000	86.2922000
age.s[46]	1.0001300	1.0002757	2.197940e+01	21.7189000	20.7958896	20001.0	1.387230e-02	0.95	19.0001000	25.5888000
age.s[47]	1.0001272	1.0004231	3.574108e+01	35.7120000	35.6492387	19110.9	1.806260e-02	0.95	30.9861000	40.7589000
age.s[48]	0.9998808	0.9998853	3.508344e+01	35.0734000	34.9357584	19081.8	1.862150e-02	0.95	29.9907000	40.0226000
age.s[49]	1.0000886	1.0003611	2.457591e+01	24.4789000	24.1882046	20895.4	1.662870e-02	0.95	19.8532000	29.1338000
age.s[50]	0.9999120	0.9999857	2.056016e+01	20.2048000	19.3306436	20001.0	9.375300e-03	0.95	19.0000000	23.1928000
age.s[51]	0.9999095	0.9999735	7.959317e+01	79.4460000	79.1429180	20485.8	2.770400e-02	0.95	72.2103000	87.7244000
age.s[52]	0.9998800	0.9998912	5.841388e+01	58.3792000	58.3493942	18639.5	1.895070e-02	0.95	53.3288000	63.4215000
age.s[53]	1.0001066	1.0006455	5.237649e+01	52.3377000	52.3831576	19176.4	1.731550e-02	0.95	47.6692000	57.0670000
age.s[54]	1.0002484	1.0012077	7.095982e+01	70.9015000	70.8152780	20001.0	2.300550e-02	0.95	64.6610000	77.3956000
age.s[55]	1.0005537	1.0016332	6.397725e+01	63.9144000	63.8093137	19416.3	2.056500e-02	0.95	58.4407000	69.6332000
age.s[56]	0.9998988	0.9999352	7.539693e+01	75.3196000	75.3815404	20001.0	2.560770e-02	0.95	68.5017000	82.5572000
age.s[57]	1.0000428	1.0003421	4.969173e+01	49.6835000	49.8842374	18904.5	1.707080e-02	0.95	45.0411000	54.2266000
age.s[58]	1.0002573	1.0011232	3.235671e+01	32.3142000	32.0910890	19213.3	1.930550e-02	0.95	27.1166000	37.5263000
age.s[59]	1.0006039	1.0025854	7.732742e+01	77.2260000	77.0064091	20001.0	2.689730e-02	0.95	69.8392000	84.7311000
age.s[60]	1.0002747	1.0012827	2.718791e+01	27.1084000	27.0986491	18706.8	1.839190e-02	0.95	22.4393000	32.2067000
age.s[61]	1.0001585	1.0002120	3.565846e+01	35.6395000	35.6861740	19084.2	1.801480e-02	0.95	30.8039000	40.4975000
age.s[62]	1.0001681	1.0007961	6.468985e+01	64.6291000	64.5618728	20001.0	2.064010e-02	0.95	58.9974000	70.3986000
age.s[63]	1.0004329	1.0019157	9.460648e+01	95.2638000	97.8230881	20001.0	2.685240e-02	0.95	87.3626000	99.9999000
age.s[64]	1.0001550	1.0008258	6.279846e+01	62.7633000	62.7352627	20001.0	1.995250e-02	0.95	57.2370000	68.2695000
age.s[65]	0.9998602	0.9998800	7.805975e+01	77.9508000	77.6943106	20524.2	2.710700e-02	0.95	70.8638000	86.0665000
age.s[66]	0.9998855	0.9999493	6.366826e+01	63.6197000	63.1446369	20001.0	1.986810e-02	0.95	58.3620000	69.3372000
age.s[67]	0.9998915	0.9999736	2.902839e+01	28.9272000	28.3712974	20001.0	1.890180e-02	0.95	23.8191000	34.1914000
age.s[68]	1.0001655	1.0009010	2.172957e+01	21.4368000	20.6796222	20001.0	1.328020e-02	0.95	19.0002000	25.2712000
age.s[69]	1.0007754	1.0029657	7.177313e+01	71.7128000	71.7966431	20967.8	2.286660e-02	0.95	65.3447000	78.2394000
age.s[70]	1.0002122	1.0011819	3.282248e+01	32.7952000	32.7836731	19267.9	1.900220e-02	0.95	27.7680000	38.0265000
age.s[71]	1.0008378	1.0034169	4.717064e+01	47.1466000	46.8842450	18405.0	1.682430e-02	0.95	42.8260000	51.8670000
age.s[72]	1.0003156	1.0011764	2.711788e+01	27.0357000	27.0157166	19203.7	1.837750e-02	0.95	22.3791000	32.3974000
age.s[73]	1.0004296	1.0015477	8.745405e+01	87.3520000	87.1698988	20001.0	3.368090e-02	0.95	77.9683000	96.6265000
age.s[74]	0.9999676	1.0002813	5.646843e+01	56.4357000	56.0541523	20001.0	1.791750e-02	0.95	51.5617000	61.4244000
age.s[75]	1.0001658	1.0005238	6.111363e+01	61.0929000	61.3855045	20001.0	1.924310e-02	0.95	55.8895000	66.5412000

age.s[76]	1.0001922	1.0010546	3.025366e+01	30.1830000	29.9146271	18455.6	1.969800e-02	0.95	25.1479000	35.5864000
age.s[77]	1.0004472	1.0015984	4.258481e+01	42.5454000	42.2394636	18621.9	1.647670e-02	0.95	38.2649000	46.9684000
age.s[78]	1.0000875	1.0004567	8.140523e+01	81.2794000	81.3081649	20001.0	2.992110e-02	0.95	73.0234000	89.5779000
age.s[79]	1.0006748	1.0028309	2.056259e+01	20.2065000	19.3322430	21768.0	9.055500e-03	0.95	19.0000000	23.2444000
age.s[80]	1.0000942	1.0006527	3.573850e+01	35.7124000	35.6788738	18500.2	1.835600e-02	0.95	30.8211000	40.5772000
age.s[81]	1.0005523	1.0021255	2.293973e+01	22.7687000	22.5407137	19472.0	1.543710e-02	0.95	19.0098000	26.7484000
age.s[82]	1.0000350	1.0000448	6.730569e+01	67.2821000	67.8436881	20001.0	2.135900e-02	0.95	61.4248000	73.1941000
age.s[83]	1.0007327	1.0024179	3.639670e+01	36.3945000	36.4423742	18809.6	1.814320e-02	0.95	31.5904000	41.3471000
age.s[84]	1.0001456	1.0004225	3.345583e+01	33.4324000	33.2683752	20001.0	1.839730e-02	0.95	28.4332000	38.5520000
age.s[85]	1.0006888	1.0028918	5.777143e+01	57.7179000	57.6142625	20001.0	1.835250e-02	0.95	52.6958000	62.8189000
age.s[86]	1.0001287	1.0005519	7.078268e+01	70.7087000	70.0418665	20001.0	2.273730e-02	0.95	64.5923000	77.0738000
age.s[87]	1.0006201	1.0025309	5.495129e+01	54.9250000	54.3488681	18855.5	1.809550e-02	0.95	50.1280000	59.8134000
age.s[88]	1.0002918	1.0011213	3.272666e+01	32.7021000	32.5380141	18731.4	1.934650e-02	0.95	27.6168000	37.8704000
age.s[89]	1.0001819	1.0004102	5.615276e+01	56.1312000	56.2737925	18941.7	1.854560e-02	0.95	50.9584000	60.9931000
age.s[90]	1.0000654	1.0003687	5.602291e+01	55.9821000	55.9390358	18723.7	1.857090e-02	0.95	51.1016000	61.0383000
age.s[91]	1.0000766	1.0005140	7.079928e+01	70.7358000	71.1356689	20001.0	2.293120e-02	0.95	64.5547000	77.1767000
age.s[92]	1.0002134	1.0011046	6.753571e+01	67.4743000	67.4855017	20001.0	2.161480e-02	0.95	61.7771000	73.7065000
age.s[93]	1.0000578	1.0004807	6.787888e+01	67.8276000	67.7427579	20001.0	2.156620e-02	0.95	62.1074000	74.0565000
age.s[94]	1.0000675	1.0001047	7.041581e+01	70.3537000	70.3495717	20021.5	2.294960e-02	0.95	64.2914000	77.0323000
age.s[95]	1.0002486	1.0007681	7.342870e+01	73.3787000	73.5764245	20001.0	2.445850e-02	0.95	66.7009000	80.2172000
age.s[96]	1.0001263	1.0005745	2.100145e+01	20.6389000	19.3968639	18898.8	1.153900e-02	0.95	19.0001000	24.0972000
age.s[97]	1.0001875	1.0008321	3.213690e+01	32.0979000	31.9493043	19119.5	1.893060e-02	0.95	27.0365000	37.2222000
age.s[98]	1.0002372	1.0009218	2.055417e+01	20.1931000	19.3189379	20464.0	9.323500e-03	0.95	19.0000000	23.1950000
age.s[99]	1.0001582	1.0005915	2.055580e+01	20.1929000	19.3332387	20001.0	9.439200e-03	0.95	19.0001000	23.2057000
age.s[100]	0.9999623	1.0001198	6.702672e+01	66.9940000	66.9405892	21659.1	2.068600e-02	0.95	61.2857000	73.1266000
age.s[101]	0.9999197	0.9999905	7.104296e+01	70.9703000	70.5106856	20001.0	2.294980e-02	0.95	64.8715000	77.4558000
age.s[102]	1.0001420	1.0009014	8.816928e+01	88.1079000	87.8679698	20001.0	3.313380e-02	0.95	79.2250000	97.5299000
age.s[103]	0.9999419	1.0001252	7.511116e+01	75.0257000	75.0097473	20592.9	2.491170e-02	0.95	68.2412000	82.2132000
age.s[104]	1.0001465	1.0007413	5.543497e+01	55.4209000	55.4507301	19499.8	1.766540e-02	0.95	50.6847000	60.3154000
age.s[105]	0.9999710	1.0000063	7.642048e+01	76.3894000	76.9723740	19568.9	2.630800e-02	0.95	69.3513000	83.6749000
age.s[106]	1.0002422	1.0008160	6.103002e+01	61.0024000	60.7190670	20001.0	1.920760e-02	0.95	55.6544000	66.2276000
age.s[107]	1.0000077	1.0004005	6.803453e+01	67.9477000	67.9435036	21118.0	2.138470e-02	0.95	61.9702000	74.0244000
age.s[108]	1.0003705	1.0016401	6.038559e+01	60.3370000	60.3582027	20001.0	1.908380e-02	0.95	54.9075000	65.5006000
age.s[109]	0.9999487	1.0000294	8.787955e+01	87.7700000	87.8420460	20545.3	3.358330e-02	0.95	78.6626000	97.3479000
age.s[110]	1.0000225	1.0001893	8.540952e+01	85.2999000	84.3021729	20001.0	3.236230e-02	0.95	76.3882000	94.2925000
age.s[111]	1.0001005	1.0003835	2.100753e+01	20.6413000	19.3917668	21521.4	1.079500e-02	0.95	19.0000000	24.1155000
age.s[112]	1.0000367	1.0002123	2.791568e+01	27.8418000	27.7652952	20001.0	1.771660e-02	0.95	23.0111000	32.8078000
age.s[113]	1.0003272	1.0014501	3.721560e+01	37.2071000	37.2185350	18965.6	1.773500e-02	0.95	32.4426000	42.0061000
age.s[114]	1.0000402	1.0000615	6.156462e+01	61.5445000	61.7989956	19484.5	1.971320e-02	0.95	56.3266000	67.0825000

age.s[115]	1.0009714	1.0036452	4.928772e+01	49.2568000	49.3047511	18924.7	1.676120e-02	0.95	44.7528000	53.8029000
age.s[116]	0.9999916	1.0000354	6.781053e+01	67.7676000	67.7074008	20001.0	2.163480e-02	0.95	61.9305000	73.8193000
age.s[117]	1.0001232	1.0001700	2.123212e+01	20.8886000	19.5311010	20001.0	1.195340e-02	0.95	19.0001000	24.4914000
age.s[118]	0.9999136	1.0000699	2.356671e+01	23.4570000	22.8595300	18982.8	1.681160e-02	0.95	19.0820000	27.6595000
age.s[119]	1.0001213	1.0005414	6.562108e+01	65.5875000	65.8150482	20001.0	2.081120e-02	0.95	59.8369000	71.4036000
age.s[120]	1.0001840	1.0003138	7.908117e+01	79.0030000	79.1422833	19524.2	2.754540e-02	0.95	71.7742000	86.8196000
age.s[121]	1.0003790	1.0015047	6.753048e+01	67.4812000	67.6424047	20001.0	2.147890e-02	0.95	61.7678000	73.6784000
age.s[122]	1.0002846	1.0005641	7.568600e+01	75.5909000	75.2008568	20001.0	2.584970e-02	0.95	68.4996000	82.8223000
age.s[123]	1.0001783	1.0008960	3.726579e+01	37.2471000	37.2313560	18479.4	1.705360e-02	0.95	32.6576000	41.6624000
age.s[124]	1.0004215	1.0009897	3.493218e+01	34.9099000	34.9280072	19216.2	1.844520e-02	0.95	29.9674000	39.9438000
age.s[125]	1.0002642	1.0010216	4.687469e+01	46.8323000	46.9193662	18656.6	1.655070e-02	0.95	42.4863000	51.2732000
age.s[126]	1.0004131	1.0017782	5.971476e+01	59.6597000	59.7664281	19586.8	1.918540e-02	0.95	54.5614000	64.9894000
age.s[127]	1.0001165	1.0005313	2.119972e+01	20.8433000	19.5408358	20254.9	1.181490e-02	0.95	19.0001000	24.4219000
age.s[128]	1.0001984	1.0008359	5.774534e+01	57.7036000	57.9473967	20001.0	1.804570e-02	0.95	52.8812000	62.8685000
age.s[129]	1.0000673	1.0002585	6.824779e+01	68.2067000	68.3530252	20942.8	2.142970e-02	0.95	62.2944000	74.4449000
age.s[130]	1.0001560	1.0009097	7.285127e+01	72.7687000	72.2741855	20001.0	2.368680e-02	0.95	66.3306000	79.3072000
age.s[131]	1.0005178	1.0021014	2.923123e+01	29.1730000	29.1245235	19924.1	1.809250e-02	0.95	24.4206000	34.4282000
age.s[132]	1.0003473	1.0008201	5.175284e+01	51.7209000	51.5190527	19368.9	1.701480e-02	0.95	47.3017000	56.5472000
age.s[133]	1.0002120	1.0003896	7.146997e+01	71.4302000	72.0153905	20001.0	2.361020e-02	0.95	64.9690000	77.9994000
age.s[134]	1.0002548	1.0011757	3.789708e+01	37.8652000	37.8609990	19216.4	1.764090e-02	0.95	33.1408000	42.6929000
age.s[135]	1.0003124	1.0014854	4.842507e+01	48.3994000	48.4897489	19024.0	1.666310e-02	0.95	43.9905000	52.9297000
age.s[136]	1.0006253	1.0026878	4.701107e+01	46.9890000	46.5930058	18348.3	1.689880e-02	0.95	42.5263000	51.4584000
age.s[137]	1.0001183	1.0005584	5.781361e+01	57.7666000	57.6368856	19451.6	1.865290e-02	0.95	52.7487000	62.8674000
age.s[138]	1.0001331	1.0007299	2.295202e+01	22.8195000	22.4371422	18753.1	1.584200e-02	0.95	19.0069000	26.7662000
age.s[139]	1.0000945	1.0005107	6.683452e+01	66.7864000	66.8541478	19561.8	2.119590e-02	0.95	61.0283000	72.6165000
age.s[140]	1.0001952	1.0010320	3.021756e+01	30.1651000	30.3336900	18824.2	1.896640e-02	0.95	25.2864000	35.3979000
age.s[141]	1.0000058	1.0003702	3.172006e+01	31.6713000	31.3485045	18831.4	1.836200e-02	0.95	26.8769000	36.7314000
age.s[142]	1.0005938	1.0022962	4.887294e+01	48.8028000	48.3410450	19321.6	1.667590e-02	0.95	44.3977000	53.4446000
age.s[143]	1.0000399	1.0004393	3.052026e+01	30.4537000	29.9932433	18878.0	1.899430e-02	0.95	25.5996000	35.8070000
age.s[144]	1.0002630	1.0011805	6.359046e+01	63.5485000	63.4971980	20001.0	1.970330e-02	0.95	58.3805000	69.1567000
age.s[145]	1.0007128	1.0029258	6.501515e+01	64.9544000	64.9021086	19300.1	2.081810e-02	0.95	59.3870000	70.7363000
age.s[146]	1.0003474	1.0006526	3.174134e+01	31.6836000	31.6570337	18768.8	1.956220e-02	0.95	26.4415000	36.8584000
age.s[147]	0.9999432	1.0001020	6.379421e+01	63.7361000	63.1971043	21802.3	1.941760e-02	0.95	58.2351000	69.4476000
age.s[148]	1.0005124	1.0015939	3.660715e+01	36.6109000	36.8129021	18895.2	1.827730e-02	0.95	31.6921000	41.5333000
age.s[149]	1.0001087	1.0007385	5.648644e+01	56.4230000	56.1184498	20521.5	1.784520e-02	0.95	51.5405000	61.4917000
age.s[150]	0.9999618	1.0001131	6.856100e+01	68.5108000	68.6269242	21176.0	2.125950e-02	0.95	62.6746000	74.8422000
age.s[151]	0.9999346	1.0001373	4.890283e+01	48.8887000	48.9020255	19235.4	1.637000e-02	0.95	44.3759000	53.2415000
age.s[152]	1.0002801	1.0014191	4.217331e+01	42.1573000	42.3525478	17648.7	1.753040e-02	0.95	37.6552000	46.7027000
age.s[153]	0.9999107	0.9999584	6.904239e+01	68.9818000	68.8385893	20001.0	2.228940e-02	0.95	62.9212000	75.2520000

age.s[154]	1.0004264	1.0016692	5.929204e+01	59.2644000	59.3700888	20001.0	1.887170e-02	0.95	54.0703000	64.5454000
age.s[155]	1.0002144	1.0005368	2.411401e+01	24.0082000	24.0755555	19208.5	1.692220e-02	0.95	19.5560000	28.5041000
age.s[156]	1.0002828	1.0012563	6.950625e+01	69.4459000	69.1641110	20001.0	2.295850e-02	0.95	63.2076000	75.8310000
age.s[157]	1.0003649	1.0004147	6.579188e+01	65.7517000	65.2986355	20001.0	2.080720e-02	0.95	60.1077000	71.5975000
age.s[158]	1.0000736	1.0003328	7.737153e+01	77.2810000	77.1693767	19465.9	2.644910e-02	0.95	70.0783000	84.5294000
age.s[159]	1.0001686	1.0005911	5.449123e+01	54.4563000	54.3320507	20001.0	1.723390e-02	0.95	49.7059000	59.3367000
age.s[160]	1.0002337	1.0012148	5.144004e+01	51.4043000	51.5064794	20001.0	1.656490e-02	0.95	46.8634000	56.0009000
age.s[161]	0.9999096	1.0000362	4.574120e+01	45.7034000	45.2651288	19341.2	1.637740e-02	0.95	41.1287000	50.0491000
age.s[162]	1.0001097	1.0007935	3.925143e+01	39.2437000	39.3527059	18388.0	1.748280e-02	0.95	34.7497000	43.9802000
age.s[163]	0.9999802	1.0000294	4.148550e+01	41.4551000	41.6154281	18900.7	1.675180e-02	0.95	36.9940000	46.0218000
age.s[164]	1.0003469	1.0007569	2.692060e+01	26.8859000	27.0817287	18033.0	1.777860e-02	0.95	22.3453000	31.6872000
age.s[165]	0.9999565	1.0000781	4.527265e+01	45.2354000	45.1609967	18832.8	1.656140e-02	0.95	40.8068000	49.7207000
age.s[166]	1.0016133	1.0037174	2.055597e+01	20.1986000	19.3304940	20001.0	9.420900e-03	0.95	19.0000000	23.2216000
age.s[167]	0.9998988	0.9999787	8.323043e+01	83.0763000	82.9476813	20001.0	3.014460e-02	0.95	75.2014000	91.8451000
age.s[168]	1.0002132	1.0008524	5.796943e+01	57.9418000	58.2743123	19219.9	1.855710e-02	0.95	53.1255000	63.2599000
age.s[169]	1.0001312	1.0007364	4.938003e+01	49.3688000	49.4770951	18585.8	1.712730e-02	0.95	44.8264000	53.9612000
age.s[170]	1.0000529	1.0005517	4.292182e+01	42.8803000	42.5695053	19240.0	1.633420e-02	0.95	38.3919000	47.2896000
age.s[171]	1.0000963	1.0007339	3.357652e+01	33.5531000	33.6031744	19278.0	1.874490e-02	0.95	28.4407000	38.6162000
age.s[172]	1.0001382	1.0009056	3.241064e+01	32.3743000	32.1522976	18851.1	1.960260e-02	0.95	27.2645000	37.7373000
age.s[173]	1.0000440	1.0003333	7.058326e+01	70.5038000	70.8716882	20001.0	2.282740e-02	0.95	64.2620000	76.8604000
age.s[174]	0.9998908	0.9999530	6.235072e+01	62.3094000	62.2327525	19500.2	1.992360e-02	0.95	56.9864000	67.8629000
age.s[175]	1.0007207	1.0028544	4.006380e+01	40.0417000	40.0117666	18598.3	1.689420e-02	0.95	35.4861000	44.5367000
age.s[176]	1.0002052	1.0003592	7.954576e+01	79.4444000	79.3249057	20001.0	2.825680e-02	0.95	71.7091000	87.2668000
age.s[177]	0.9999771	1.0002976	5.639179e+01	56.3231000	55.7931000	20083.4	1.802500e-02	0.95	51.5887000	61.5345000
age.s[178]	1.0005655	1.0023715	5.465402e+01	54.6125000	54.5614367	19141.9	1.793370e-02	0.95	49.8955000	59.6327000
age.s[179]	1.0001773	1.0008266	2.100318e+01	20.6300000	19.4711997	20001.0	1.124300e-02	0.95	19.0000000	24.0746000
age.s[180]	0.9999627	1.0000322	3.871269e+01	38.6862000	38.3809427	19183.7	1.718980e-02	0.95	34.1846000	43.5392000
age.s[181]	0.9999546	1.0001282	7.305676e+01	72.9836000	73.1820619	20258.3	2.427850e-02	0.95	66.5183000	80.0511000
age.s[182]	1.0004594	1.0016201	7.994581e+01	79.8841000	80.1634083	20001.0	2.821670e-02	0.95	72.4156000	88.0304000
age.s[183]	0.9999058	0.9999926	7.217642e+01	72.1300000	72.4011947	20001.0	2.371050e-02	0.95	65.8191000	78.8797000
age.s[184]	1.0002040	1.0009545	2.943580e+01	29.3825000	29.5679707	20001.0	1.862920e-02	0.95	24.4407000	34.6565000
age.s[185]	1.0000716	1.0006652	9.259907e+01	92.9827000	94.1273056	20001.0	3.073120e-02	0.95	84.8990000	99.9951000
age.s[186]	1.0000962	1.0006673	5.915325e+01	59.1061000	58.8837409	19421.9	1.899510e-02	0.95	54.2064000	64.5153000
age.s[187]	1.0004485	1.0020427	7.185839e+01	71.7861000	71.4828645	20001.0	2.350970e-02	0.95	65.4990000	78.4436000
age.s[188]	1.0003593	1.0016845	7.518424e+01	75.0904000	75.2153472	20001.0	2.552010e-02	0.95	68.4483000	82.5266000
age.s[189]	1.0000976	1.0002249	8.868060e+01	88.6440000	88.7912369	20001.0	3.351750e-02	0.95	79.9044000	98.2450000
age.s[190]	1.0001544	1.0007800	3.085843e+01	30.8138000	30.8764474	20001.0	1.900610e-02	0.95	25.6750000	36.1238000
age.s[191]	1.0006562	1.0027305	5.782375e+01	57.7892000	57.5766731	19347.5	1.841550e-02	0.95	52.9011000	62.9434000
age.s[192]	1.0001387	1.0008724	6.740086e+01	67.3392000	67.1829887	20001.0	2.160710e-02	0.95	61.4817000	73.3856000

age.s[193]	1.0006375	1.0026908	3.500822e+01	34.9982000	35.0843718	19499.3	1.834310e-02	0.95	30.0075000	40.0358000
age.s[194]	1.0005668	1.0022736	7.457447e+01	74.4976000	74.4403490	20001.0	2.503110e-02	0.95	67.9145000	81.6520000
age.s[195]	1.0000760	1.0005803	7.715396e+01	77.0593000	76.8786575	20001.0	2.612580e-02	0.95	69.8838000	84.2698000
age.s[196]	1.0001594	1.0009891	4.523545e+01	45.2301000	45.4083232	18215.1	1.665310e-02	0.95	40.7230000	49.5105000
age.s[197]	1.0000796	1.0002026	5.420464e+01	54.1717000	53.8885142	19465.5	1.755420e-02	0.95	49.3203000	58.8605000
age.s[198]	1.0006055	1.0020232	2.101062e+01	20.6559000	19.4480907	20001.0	1.118310e-02	0.95	19.0000000	24.0757000
age.s[199]	0.9999615	1.0000155	5.809805e+01	58.0448000	57.9702229	21352.4	1.780520e-02	0.95	53.0884000	63.1881000
age.s[200]	1.0000162	1.0004227	2.382997e+01	23.7065000	23.3576167	19137.6	1.682120e-02	0.95	19.3697000	28.1144000
age.s_c[1]	1.0000281	1.0003407	2.582237e+01	24.9192000	21.3038664	20001.0	3.495870e-02	0.95	19.0002000	35.2491000
age.s_c[2]	1.0000744	1.0001427	4.522630e+01	45.1407000	44.4574801	20001.0	5.512760e-02	0.95	29.6787000	60.3593000
age.s_c[3]	1.0001215	1.0002708	3.379669e+01	33.4914000	33.4772268	20001.0	5.169450e-02	0.95	19.3673000	46.6973000
age.s_c[4]	1.0002970	1.0008167	7.045303e+01	70.4622000	71.1749445	20001.0	5.722850e-02	0.95	54.8471000	86.3059000
age.s_c[5]	1.0000422	1.0004041	8.745638e+01	88.1110000	90.4681404	20001.0	5.208190e-02	0.95	74.3908000	99.9683000
age.s_c[6]	1.0002904	1.0013759	6.856206e+01	68.5550000	70.0130062	20001.0	5.791610e-02	0.95	52.9559000	84.8859000
age.s_c[7]	0.9999914	1.0002607	3.309538e+01	32.7834000	33.0372121	20001.0	5.077760e-02	0.95	19.0075000	45.6350000
age.s_c[8]	1.0002156	1.0010400	6.908278e+01	69.0434000	68.4852552	20001.0	5.774690e-02	0.95	53.1724000	85.1263000
age.s_c[9]	1.0000580	1.0005117	6.791160e+01	67.9409000	67.8276844	20001.0	5.727440e-02	0.95	51.4947000	83.1812000
age.s_c[10]	1.0000533	1.0004020	3.908276e+01	39.0181000	39.4422494	20001.0	5.437970e-02	0.95	23.8701000	54.0754000
age.s_c[11]	0.9999805	1.0003079	3.234243e+01	31.9056000	31.5275033	20001.0	4.967850e-02	0.95	19.0046000	44.7005000
age.s_c[12]	0.9999215	1.0000653	5.226750e+01	52.3096000	52.8776242	20001.0	5.551650e-02	0.95	36.7344000	67.5410000
age.s_c[13]	1.0000840	1.0002196	4.268553e+01	42.6828000	42.6378002	20001.0	5.516070e-02	0.95	27.7097000	58.1673000
age.s_c[14]	0.9999846	1.0000926	3.056613e+01	29.9821000	28.1293261	20787.4	4.598970e-02	0.95	19.0553000	42.3666000
age.s_c[15]	1.0003206	1.0011775	7.906698e+01	79.1627000	77.1104882	20001.0	5.846820e-02	0.95	62.9128000	95.2090000
age.s_c[16]	0.9999459	1.0001058	7.746624e+01	77.5045000	77.4799608	20001.0	5.812560e-02	0.95	61.2292000	93.4337000
age.s_c[17]	1.0003720	1.0014601	7.724305e+01	77.3133000	77.8801640	20437.2	5.777360e-02	0.95	61.1265000	93.5977000
age.s_c[18]	0.9998906	0.9999236	3.732227e+01	37.2465000	36.3644413	20001.0	5.402730e-02	0.95	22.1020000	51.7136000
age.s_c[19]	0.9999717	1.0001910	9.056162e+01	91.5037000	94.6642353	20001.0	4.503450e-02	0.95	78.7441000	99.9992000
age.s_c[20]	0.9998674	0.9998854	7.079751e+01	70.7691000	69.6875130	20001.0	5.748710e-02	0.95	54.8975000	86.6102000
age.s_c[21]	0.9998833	0.9999535	4.691347e+01	46.8454000	46.9717743	20001.0	5.480050e-02	0.95	32.0112000	62.2052000
age.s_c[22]	0.9999578	0.9999925	6.583440e+01	65.8114000	66.0199895	20001.0	5.705540e-02	0.95	50.3915000	81.8639000
age.s_c[23]	0.9998827	0.9999555	3.012476e+01	29.5198000	29.0335956	20159.1	4.598350e-02	0.95	19.0006000	41.9583000
age.s_c[24]	1.0000729	1.0004745	2.561530e+01	24.6874000	22.1852965	20466.3	3.382460e-02	0.95	19.0008000	34.9577000
age.s_c[25]	0.9999630	1.0000182	6.015699e+01	60.1356000	60.1159209	20001.0	5.650240e-02	0.95	45.1563000	76.3623000
age.s_c[26]	1.0001111	1.0004528	8.639561e+01	87.0383000	87.8538277	19093.5	5.537560e-02	0.95	72.7635000	99.9949000
age.s_c[27]	1.0000039	1.0000220	7.585381e+01	75.8293000	75.0355949	22195.2	5.554500e-02	0.95	59.9437000	92.0028000
age.s_c[28]	1.0001763	1.0008148	4.172500e+01	41.6437000	41.2190871	20001.0	5.406330e-02	0.95	26.6107000	56.4331000
age.s_c[29]	1.0000009	1.0002057	5.914143e+01	59.0993000	58.9763102	20001.0	5.659970e-02	0.95	43.3894000	74.5276000
age.s_c[30]	0.9999508	1.0000523	3.592107e+01	35.7806000	36.1610609	21289.8	5.163490e-02	0.95	20.9416000	50.0484000
age.s_c[31]	0.9999154	0.9999528	7.089184e+01	70.8750000	70.7638355	21047.0	5.632350e-02	0.95	54.7473000	86.7210000

age.s_c[32]	1.0000821	1.0001749	7.298254e+01	72.9307000	72.6502393	19553.7	5.908410e-02	0.95	56.6827000	89.1568000
age.s_c[33]	1.0000513	1.0003829	8.986228e+01	90.7603000	94.8175438	20001.0	4.708260e-02	0.95	77.6205000	99.9929000
age.s_c[34]	0.9999906	1.0001734	2.603852e+01	25.1106000	20.7601397	20001.0	3.577440e-02	0.95	19.0007000	35.6601000
age.s_c[35]	1.0001250	1.0005821	4.204282e+01	41.9979000	41.8912709	20001.0	5.502050e-02	0.95	27.1106000	57.6128000
age.s_c[36]	1.0000303	1.0002861	8.610597e+01	86.6495000	87.1654698	20001.0	5.400890e-02	0.95	72.6923000	99.9901000
age.s_c[37]	1.0000137	1.0003002	8.193453e+01	82.0491000	81.7944328	20001.0	5.777270e-02	0.95	67.0715000	98.3141000
age.s_c[38]	1.0002191	1.0009613	2.561142e+01	24.7059000	21.8629142	20001.0	3.413340e-02	0.95	19.0011000	34.8294000
age.s_c[39]	1.0001333	1.0005769	8.572736e+01	86.3021000	88.9539995	20499.4	5.445520e-02	0.95	72.1053000	99.9624000
age.s_c[40]	1.0002289	1.0008117	9.020910e+01	91.2121000	92.6696541	20001.0	4.634220e-02	0.95	78.0958000	99.9971000
age.s_c[41]	1.0001390	1.0003788	5.776768e+01	57.7212000	57.5070894	20001.0	5.554200e-02	0.95	43.0302000	73.7767000
age.s_c[42]	1.0002327	1.0007343	2.581252e+01	24.8790000	21.9837829	20001.0	3.493360e-02	0.95	19.0001000	35.1764000
age.s_c[43]	0.9999597	1.0001815	3.157693e+01	31.1349000	30.4739770	20461.8	4.809490e-02	0.95	19.0038000	43.6884000
age.s_c[44]	1.0002168	1.0011870	5.379141e+01	53.7659000	54.5278778	20001.0	5.585550e-02	0.95	38.9416000	69.8050000
age.s_c[45]	1.0002255	1.0008708	7.841605e+01	78.4693000	78.3496979	19595.0	5.918780e-02	0.95	62.7569000	95.2175000
age.s_c[46]	1.0000438	1.0002698	2.626540e+01	25.2848000	21.4221073	20001.0	3.677630e-02	0.95	19.0019000	36.2281000
age.s_c[47]	0.9999867	1.0002099	3.601812e+01	35.8131000	34.7988709	20001.0	5.333400e-02	0.95	21.4131000	50.2001000
age.s_c[48]	1.0001048	1.0004757	3.534182e+01	35.1330000	34.3421042	20001.0	5.319170e-02	0.95	19.8000000	48.6674000
age.s_c[49]	1.0002525	1.0007315	2.762758e+01	26.7924000	24.4805377	20044.2	4.048240e-02	0.95	19.0002000	38.2284000
age.s_c[50]	1.0006375	1.0024145	2.562621e+01	24.6661000	21.4381622	20001.0	3.428520e-02	0.95	19.0004000	34.9003000
age.s_c[51]	0.9999286	1.0000715	7.942321e+01	79.5280000	78.8489377	20001.0	5.861540e-02	0.95	63.7473000	96.0780000
age.s_c[52]	1.0000969	1.0006841	5.856069e+01	58.5568000	56.8333325	20001.0	5.596510e-02	0.95	43.2082000	74.0120000
age.s_c[53]	1.0006721	1.0028406	5.243738e+01	52.3913000	52.7134646	20001.0	5.607000e-02	0.95	36.9871000	67.9300000
age.s_c[54]	0.9999297	1.0000990	7.094882e+01	70.9538000	71.9151144	19393.8	5.897870e-02	0.95	54.7521000	87.1271000
age.s_c[55]	1.0000003	1.0002634	6.399227e+01	64.0648000	64.6103017	20951.9	5.552040e-02	0.95	48.0626000	79.4878000
age.s_c[56]	1.0001324	1.0007708	7.531829e+01	75.3089000	75.1756664	20001.0	5.856280e-02	0.95	59.0285000	91.3778000
age.s_c[57]	0.9999970	1.0000338	4.972796e+01	49.6259000	47.5148947	20001.0	5.506570e-02	0.95	34.8502000	64.9504000
age.s_c[58]	1.0003316	1.0015433	3.312624e+01	32.7568000	32.6463447	20001.0	5.069830e-02	0.95	19.0539000	45.5381000
age.s_c[59]	0.9999635	1.0001823	7.730186e+01	77.3580000	76.5376756	20001.0	5.839370e-02	0.95	61.6954000	94.1113000
age.s_c[60]	1.0000118	1.0001634	2.920704e+01	28.5182000	27.8311035	20495.2	4.402010e-02	0.95	19.0021000	40.5582000
age.s_c[61]	1.0000665	1.0002799	3.598534e+01	35.7973000	35.3185014	20001.0	5.308110e-02	0.95	21.2211000	50.0230000
age.s_c[62]	1.0004746	1.0016781	6.476145e+01	64.7512000	64.4405147	20543.4	5.620440e-02	0.95	48.8178000	80.5649000
age.s_c[63]	1.0005426	1.0015324	9.130344e+01	92.2828000	96.5022810	20581.1	4.215360e-02	0.95	79.8058000	99.9976000
age.s_c[64]	1.0000271	1.0002853	6.276272e+01	62.8259000	63.2423598	20001.0	5.654430e-02	0.95	47.6634000	78.9727000
age.s_c[65]	0.9998884	0.9999398	7.798992e+01	78.0694000	77.8180664	20001.0	5.805650e-02	0.95	62.2797000	94.3214000
age.s_c[66]	0.9999338	1.0000159	6.364466e+01	63.6719000	63.1783486	20001.0	5.632860e-02	0.95	47.5946000	78.8726000
age.s_c[67]	0.9999776	1.0002068	3.047927e+01	29.9519000	28.1431069	19553.7	4.754780e-02	0.95	19.0001000	42.2475000
age.s_c[68]	0.9999582	1.0001325	2.618935e+01	25.2548000	20.6025979	20001.0	3.651490e-02	0.95	19.0002000	35.9947000
age.s_c[69]	1.0000046	1.0004035	7.165980e+01	71.6415000	71.6821409	20001.0	5.819310e-02	0.95	55.6223000	87.6829000
age.s_c[70]	1.0002801	1.0010442	3.351642e+01	33.2443000	33.9290464	20001.0	5.087420e-02	0.95	19.6941000	46.5266000

age.s_c[71]	1.0007201	1.0028635	4.712187e+01	47.1523000	47.0588045	18799.4	5.667520e-02	0.95	31.8760000	62.1377000
age.s_c[72]	0.9999808	1.0002749	2.917012e+01	28.4768000	26.6804366	20001.0	4.416110e-02	0.95	19.0003000	40.5278000
age.s_c[73]	1.0002901	1.0008926	8.634791e+01	86.9653000	88.0572016	16900.9	5.924340e-02	0.95	72.6897000	99.9579000
age.s_c[74]	0.9999834	1.0001453	5.662905e+01	56.5923000	56.1531717	20001.0	5.596280e-02	0.95	40.9280000	71.7877000
age.s_c[75]	1.0000966	1.0006354	6.115053e+01	61.0906000	60.5265919	20001.0	5.678160e-02	0.95	45.6755000	77.2127000
age.s_c[76]	1.0004025	1.0015792	3.137374e+01	30.8619000	30.9858574	20001.0	4.867640e-02	0.95	19.0431000	43.5995000
age.s_c[77]	0.9999540	1.0001993	4.263087e+01	42.5836000	42.2204096	20001.0	5.468870e-02	0.95	27.3597000	57.7825000
age.s_c[78]	1.0000970	1.0006710	8.117693e+01	81.3501000	81.8947168	20001.0	5.793310e-02	0.95	65.1776000	96.8118000
age.s_c[79]	1.0001238	1.0006688	2.558338e+01	24.6686000	20.9546788	20001.0	3.409820e-02	0.95	19.0022000	34.8606000
age.s_c[80]	0.9999485	1.0000884	3.609404e+01	35.8867000	35.1207740	20001.0	5.351080e-02	0.95	21.2732000	50.1999000
age.s_c[81]	0.9999200	1.0000866	2.674886e+01	25.8452000	22.3778997	20001.0	3.821670e-02	0.95	19.0004000	36.8844000
age.s_c[82]	0.9999353	0.9999637	6.728084e+01	67.2996000	67.1158271	20464.4	5.668940e-02	0.95	50.9751000	82.4803000
age.s_c[83]	1.0001944	1.0009687	3.669473e+01	36.5731000	35.4660498	20577.5	5.271640e-02	0.95	21.8926000	51.2729000
age.s_c[84]	1.0000831	1.0006663	3.410251e+01	33.8606000	34.1364233	20001.0	5.151080e-02	0.95	19.7639000	47.0879000
age.s_c[85]	1.0002009	1.0009610	5.778023e+01	57.8126000	56.9239129	20001.0	5.610080e-02	0.95	42.1357000	73.1447000
age.s_c[86]	1.0000904	1.0001654	7.072163e+01	70.5990000	69.6928979	20001.0	5.727740e-02	0.95	54.8913000	86.6398000
age.s_c[87]	1.0001761	1.0003363	5.498317e+01	54.9756000	54.2023261	19858.2	5.618700e-02	0.95	39.2239000	70.1687000
age.s_c[88]	1.0002606	1.0003478	3.344312e+01	33.0912000	32.7001843	20001.0	5.108760e-02	0.95	19.3241000	46.0986000
age.s_c[89]	1.0000776	1.0005735	5.616538e+01	56.1333000	56.1625263	20001.0	5.627410e-02	0.95	40.1232000	71.2455000
age.s_c[90]	0.9999987	1.0000982	5.599817e+01	55.9027000	55.3785206	20001.0	5.627980e-02	0.95	40.9238000	72.2122000
age.s_c[91]	1.0002263	1.0011914	7.082070e+01	70.8521000	71.6131750	20001.0	5.760590e-02	0.95	55.2031000	87.0095000
age.s_c[92]	1.0002357	1.0010014	6.747280e+01	67.4968000	68.4515266	20001.0	5.777600e-02	0.95	52.0621000	84.0419000
age.s_c[93]	0.9999803	1.0001135	6.791379e+01	67.8431000	66.4285550	20001.0	5.685560e-02	0.95	51.8407000	83.3377000
age.s_c[94]	1.0001162	1.0005452	7.041920e+01	70.4076000	70.4998109	21133.2	5.635360e-02	0.95	54.6063000	86.6906000
age.s_c[95]	1.0000406	1.0002129	7.343052e+01	73.3461000	71.9111735	20001.0	5.809160e-02	0.95	57.3998000	89.4568000
age.s_c[96]	1.0000223	1.0003225	2.578003e+01	24.8421000	21.2073030	20001.0	3.486420e-02	0.95	19.0038000	35.3100000
age.s_c[97]	1.0006796	1.0023450	3.294692e+01	32.5950000	33.3859897	19504.8	5.124940e-02	0.95	19.0025000	45.5353000
age.s_c[98]	1.0003393	1.0015384	2.563429e+01	24.6945000	22.1427810	20001.0	3.428700e-02	0.95	19.0007000	34.7433000
age.s_c[99]	0.9999956	1.0002912	2.559878e+01	24.6353000	21.5270059	20001.0	3.431560e-02	0.95	19.0003000	34.7979000
age.s_c[100]	0.9999857	1.0002889	6.703356e+01	67.0380000	67.5624375	20001.0	5.694810e-02	0.95	51.3303000	82.5979000
age.s_c[101]	0.9998716	0.9998772	7.100576e+01	70.9909000	70.3518291	20001.0	5.761960e-02	0.95	55.0572000	86.9090000
age.s_c[102]	1.0001249	1.0003348	8.697740e+01	87.5885000	89.2509198	20253.8	5.288950e-02	0.95	73.4756000	99.9771000
age.s_c[103]	1.0000726	1.0003105	7.504330e+01	74.9930000	73.5063087	20001.0	5.814850e-02	0.95	58.6492000	90.9390000
age.s_c[104]	0.9999477	1.0001288	5.538674e+01	55.4161000	55.5207223	20001.0	5.573540e-02	0.95	39.9636000	70.6576000
age.s_c[105]	1.0000040	1.0003128	7.639005e+01	76.4425000	77.0693870	21347.5	5.668860e-02	0.95	60.6778000	93.0883000
age.s_c[106]	1.0000455	1.0003569	6.108835e+01	61.0619000	61.9792057	20001.0	5.648770e-02	0.95	45.2738000	76.5956000
age.s_c[107]	1.0001079	1.0006547	6.807633e+01	67.9853000	67.4841016	20579.9	5.655520e-02	0.95	51.7421000	83.4787000
age.s_c[108]	1.0006534	1.0022763	6.032473e+01	60.2821000	59.8240988	20001.0	5.569380e-02	0.95	44.5748000	75.5831000
age.s_c[109]	0.9999711	1.0002369	8.673250e+01	87.3280000	86.6625050	20001.0	5.375820e-02	0.95	73.2618000	99.9943000

age.s_c[110]	1.0000228	1.0003297	8.467935e+01	85.1086000	86.0163068	20001.0	5.593190e-02	0.95	70.5439000	99.5170000
age.s_c[111]	1.0000074	1.0003487	2.583668e+01	24.9155000	20.7649305	20001.0	3.527050e-02	0.95	19.0005000	35.4066000
age.s_c[112]	1.0013313	1.0051265	2.962885e+01	28.9859000	28.1452717	20001.0	4.539060e-02	0.95	19.0016000	41.1979000
age.s_c[113]	1.0002681	1.0012345	3.744620e+01	37.2919000	37.6954851	20001.0	5.416110e-02	0.95	22.0517000	51.9795000
age.s_c[114]	1.0001283	1.0004485	6.159779e+01	61.6291000	62.3501278	20001.0	5.622870e-02	0.95	46.1930000	77.3599000
age.s_c[115]	1.0002611	1.0013500	4.927758e+01	49.2888000	48.8177085	20001.0	5.523960e-02	0.95	33.7097000	64.3009000
age.s_c[116]	1.0001771	1.0010200	6.775574e+01	67.7638000	68.0666673	20001.0	5.679180e-02	0.95	51.7674000	83.3264000
age.s_c[117]	1.0000015	1.0003530	2.597208e+01	25.0271000	22.4150190	20001.0	3.570010e-02	0.95	19.0000000	35.5437000
age.s_c[118]	1.0001837	1.0009265	2.706480e+01	26.1685000	23.1774532	20001.0	3.920940e-02	0.95	19.0005000	37.4841000
age.s_c[119]	0.9999974	1.0000408	6.562263e+01	65.6655000	65.9317274	20001.0	5.670980e-02	0.95	49.9060000	81.0382000
age.s_c[120]	1.0000511	1.0004783	7.898702e+01	79.0020000	77.7470844	20001.0	5.771540e-02	0.95	63.0308000	94.9919000
age.s_c[121]	1.0002644	1.0008568	6.752189e+01	67.5047000	67.2509606	20001.0	5.675950e-02	0.95	51.3231000	82.7190000
age.s_c[122]	1.0001922	1.0009405	7.558899e+01	75.5650000	75.2088639	20001.0	5.863810e-02	0.95	59.0501000	91.6903000
age.s_c[123]	1.0002052	1.0009811	3.749818e+01	37.3195000	36.3547292	20001.0	5.373680e-02	0.95	23.1137000	52.7548000
age.s_c[124]	1.0000565	1.0003571	3.528293e+01	35.1497000	35.9716502	20001.0	5.246900e-02	0.95	20.8635000	49.2444000
age.s_c[125]	1.0001301	1.0005381	4.689669e+01	46.8885000	46.4268265	20001.0	5.543970e-02	0.95	31.5595000	62.2654000
age.s_c[126]	1.0003764	1.0016260	5.974765e+01	59.7417000	59.4672389	20001.0	5.615910e-02	0.95	44.2617000	75.2340000
age.s_c[127]	1.0009270	1.0029038	2.591717e+01	24.9579000	20.9968748	20001.0	3.558300e-02	0.95	19.0001000	35.5056000
age.s_c[128]	1.0000484	1.0005406	5.776295e+01	57.7005000	56.1003027	20001.0	5.630640e-02	0.95	42.1854000	73.3041000
age.s_c[129]	1.0005004	1.0015992	6.824373e+01	68.2079000	67.7250426	20819.0	5.614140e-02	0.95	52.3304000	83.7805000
age.s_c[130]	1.0000076	1.0004198	7.283407e+01	72.9243000	74.5387510	19434.9	5.832830e-02	0.95	57.0856000	88.7796000
age.s_c[131]	1.0001166	1.0005462	3.059917e+01	30.0547000	30.3514690	20001.0	4.712060e-02	0.95	19.0045000	42.4515000
age.s_c[132]	1.0001788	1.0006484	5.178858e+01	51.8249000	52.1057515	20001.0	5.608850e-02	0.95	36.7345000	67.5998000
age.s_c[133]	0.9999073	1.0000495	7.154964e+01	71.5418000	71.5487432	18532.8	6.022410e-02	0.95	55.5470000	87.4146000
age.s_c[134]	1.0000941	1.0006626	3.813110e+01	38.0149000	37.3128710	21149.1	5.234900e-02	0.95	22.8539000	52.7279000
age.s_c[135]	1.0001461	1.0005987	4.840114e+01	48.3453000	47.9228376	20404.2	5.483420e-02	0.95	32.4872000	63.1302000
age.s_c[136]	1.0001202	1.0001721	4.684900e+01	46.8290000	46.9994316	20001.0	5.553980e-02	0.95	31.6438000	62.2979000
age.s_c[137]	1.0002784	1.0007745	5.781790e+01	57.8571000	58.2863950	20001.0	5.648830e-02	0.95	42.3709000	73.4695000
age.s_c[138]	1.0002970	1.0008281	2.669197e+01	25.7828000	23.3153286	20913.7	3.706280e-02	0.95	19.0004000	36.8391000
age.s_c[139]	0.9999865	1.0002320	6.686002e+01	66.9300000	67.6809589	19600.8	5.741620e-02	0.95	51.2867000	82.7543000
age.s_c[140]	1.0001383	1.0006554	3.146630e+01	31.0132000	29.9418611	20001.0	4.861920e-02	0.95	19.0047000	43.6046000
age.s_c[141]	1.0003917	1.0018257	3.260095e+01	32.2204000	32.2694975	20001.0	5.008320e-02	0.95	19.0006000	44.9860000
age.s_c[142]	1.0002134	1.0008510	4.884880e+01	48.7464000	48.4167826	20611.5	5.501440e-02	0.95	33.7847000	64.6592000
age.s_c[143]	1.0000345	1.0003084	3.164530e+01	31.2034000	30.6500240	20001.0	4.894180e-02	0.95	19.0049000	43.8349000
age.s_c[144]	1.0003321	1.0011701	6.358144e+01	63.5837000	64.8330820	20001.0	5.677730e-02	0.95	48.4508000	79.8033000
age.s_c[145]	1.0003252	1.0009236	6.504931e+01	64.9791000	64.5093369	19252.5	5.810110e-02	0.95	49.2850000	80.7311000
age.s_c[146]	1.0002429	1.0009802	3.263500e+01	32.1727000	30.6743766	20001.0	5.028930e-02	0.95	19.0093000	45.0810000
age.s_c[147]	0.9999104	0.9999365	6.373710e+01	63.7074000	63.1714584	20001.0	5.673500e-02	0.95	47.9516000	79.3471000
age.s_c[148]	1.0002265	1.0010163	3.688467e+01	36.7579000	36.3959741	20001.0	5.396100e-02	0.95	21.9167000	51.3147000

age.s_c[149]	1.0001365	1.0002392	5.655358e+01	56.5054000	56.1162851	20423.2	5.530300e-02	0.95	40.8400000	71.7833000
age.s_c[150]	1.0002248	1.0006517	6.857508e+01	68.5774000	68.1767972	20001.0	5.744190e-02	0.95	52.9074000	84.9065000
age.s_c[151]	1.0001889	1.0007785	4.889866e+01	48.9049000	49.2463854	20001.0	5.572400e-02	0.95	33.5184000	64.3017000
age.s_c[152]	1.0000654	1.0002725	4.218552e+01	42.1624000	41.9372693	20216.6	5.457730e-02	0.95	27.0402000	57.3299000
age.s_c[153]	1.0003618	1.0016484	6.908391e+01	69.1440000	69.9014182	18499.5	5.965630e-02	0.95	53.4332000	85.0818000
age.s_c[154]	1.0001609	1.0003286	5.924758e+01	59.3201000	59.9660128	19567.4	5.653070e-02	0.95	43.8824000	74.7082000
age.s_c[155]	0.9999753	1.0002439	2.736958e+01	26.4729000	23.8446664	20615.9	3.979400e-02	0.95	19.0022000	38.0855000
age.s_c[156]	1.0002934	1.0014597	6.948402e+01	69.4781000	69.9510621	20001.0	5.749850e-02	0.95	54.0201000	85.7380000
age.s_c[157]	1.0002636	1.0013147	6.581301e+01	65.8891000	66.1564143	21140.0	5.523200e-02	0.95	49.5850000	81.1842000
age.s_c[158]	1.0001164	1.0003146	7.738276e+01	77.4432000	77.7774652	19476.0	5.892820e-02	0.95	61.5898000	93.5685000
age.s_c[159]	0.9999177	1.0000494	5.460617e+01	54.5741000	55.0696357	20001.0	5.577590e-02	0.95	39.2912000	69.9438000
age.s_c[160]	1.0001706	1.0004107	5.145569e+01	51.4339000	51.5922579	20001.0	5.527140e-02	0.95	35.6477000	66.1903000
age.s_c[161]	1.0000827	1.0003881	4.576855e+01	45.7625000	45.8099579	20001.0	5.502200e-02	0.95	30.8490000	61.2482000
age.s_c[162]	1.0002359	1.0011243	3.939947e+01	39.3269000	39.5498897	20001.0	5.431020e-02	0.95	23.4975000	53.6755000
age.s_c[163]	0.9999687	1.0000488	4.151297e+01	41.5133000	41.5842918	20001.0	5.482160e-02	0.95	26.0614000	56.5701000
age.s_c[164]	0.9999549	1.0000119	2.905850e+01	28.3785000	26.5879404	20001.0	4.420220e-02	0.95	19.0015000	40.4271000
age.s_c[165]	1.0000159	1.0004333	4.533387e+01	45.2187000	45.2817052	19410.0	5.616720e-02	0.95	29.7096000	60.3290000
age.s_c[166]	0.9999243	0.9999576	2.566353e+01	24.7186000	20.9707444	20001.0	3.448380e-02	0.95	19.0025000	35.0116000
age.s_c[167]	1.0000131	1.0002642	8.274579e+01	82.9656000	83.3935065	20001.0	5.717900e-02	0.95	68.0778000	98.6181000
age.s_c[168]	0.9999685	1.0001806	5.793272e+01	57.8593000	56.9465207	20001.0	5.568190e-02	0.95	42.4979000	73.5093000
age.s_c[169]	1.0000464	1.0002319	4.941101e+01	49.4210000	49.3528386	18903.1	5.760670e-02	0.95	33.6278000	64.5371000
age.s_c[170]	1.0001152	1.0005723	4.290539e+01	42.9114000	42.5829814	20001.0	5.530380e-02	0.95	27.2660000	57.9365000
age.s_c[171]	1.0001147	1.0004042	3.418481e+01	33.8959000	33.7633944	20001.0	5.210380e-02	0.95	19.9518000	47.6206000
age.s_c[172]	1.0001040	1.0006407	3.315275e+01	32.8169000	32.7160909	20001.0	5.102360e-02	0.95	19.0969000	45.6865000
age.s_c[173]	1.0003331	1.0010529	7.056477e+01	70.5903000	71.0010261	20001.0	5.805000e-02	0.95	54.4622000	86.9947000
age.s_c[174]	1.0000264	1.0000270	6.234149e+01	62.3499000	62.1831108	20001.0	5.674200e-02	0.95	46.7378000	78.0328000
age.s_c[175]	0.9999927	1.0003218	4.021421e+01	40.1764000	39.6928763	20001.0	5.444750e-02	0.95	25.4621000	55.7074000
age.s_c[176]	1.0000304	1.0003735	7.930906e+01	79.4196000	79.7285802	20001.0	5.811080e-02	0.95	63.7679000	95.8537000
age.s_c[177]	1.0001580	1.0008895	5.631371e+01	56.3626000	56.3417239	20001.0	5.619710e-02	0.95	41.1698000	72.3630000
age.s_c[178]	0.9999842	1.0002192	5.466312e+01	54.7416000	55.5776147	20001.0	5.593720e-02	0.95	38.8264000	69.9448000
age.s_c[179]	1.0000757	1.0006293	2.584493e+01	24.9243000	20.5100871	21410.7	3.407450e-02	0.95	19.0010000	35.3166000
age.s_c[180]	1.0003113	1.0006760	3.884302e+01	38.7483000	38.7317355	20001.0	5.449070e-02	0.95	23.5653000	53.7209000
age.s_c[181]	1.0000658	1.0005001	7.293875e+01	72.9499000	73.3687535	20001.0	5.809760e-02	0.95	57.2435000	89.3528000
age.s_c[182]	1.0002444	1.0010859	7.962696e+01	79.7146000	79.7203431	20001.0	5.823000e-02	0.95	63.9262000	95.8113000
age.s_c[183]	0.9999955	1.0001474	7.219101e+01	72.1251000	71.5205562	20001.0	5.859410e-02	0.95	55.8774000	88.3783000
age.s_c[184]	1.0002327	1.0008908	3.076364e+01	30.2241000	30.3235364	20001.0	4.745660e-02	0.95	19.0009000	42.5604000
age.s_c[185]	1.0000766	1.0005683	9.003946e+01	91.0243000	93.3114435	20001.0	4.664520e-02	0.95	77.7103000	99.9992000
age.s_c[186]	1.0000241	1.0003775	5.916192e+01	59.1330000	58.6254516	20001.0	5.618500e-02	0.95	43.4298000	74.6644000
age.s_c[187]	1.0000882	1.0001368	7.181450e+01	71.8627000	72.6671433	20001.0	5.785660e-02	0.95	55.7638000	87.9429000

age.s_c[188]	0.9999121	1.0000570	7.512161e+01	75.1018000	75.3885445	20001.0	5.812950e-02	0.95	58.5323000	90.9066000
age.s_c[189]	1.0001161	1.0007046	8.734995e+01	88.0236000	89.6873237	20001.0	5.286140e-02	0.95	73.9509000	99.9954000
age.s_c[190]	1.0001896	1.0010896	3.179615e+01	31.3260000	30.1363542	20942.7	4.798220e-02	0.95	19.0032000	44.0256000
age.s_c[191]	1.0001284	1.0008022	5.789036e+01	57.9457000	57.9519748	20001.0	5.590780e-02	0.95	42.2348000	73.1241000
age.s_c[192]	1.0001938	1.0005803	6.736121e+01	67.3090000	66.1230057	20001.0	5.724700e-02	0.95	51.9559000	83.7384000
age.s_c[193]	1.0000746	1.0004024	3.541024e+01	35.2274000	35.2780417	20426.6	5.245470e-02	0.95	20.6788000	49.2715000
age.s_c[194]	1.0000447	1.0005098	7.451450e+01	74.5497000	75.0265879	20001.0	5.854770e-02	0.95	58.0099000	90.6032000
age.s_c[195]	1.0003644	1.0012011	7.711993e+01	77.1226000	76.9875594	20001.0	5.788940e-02	0.95	60.9990000	92.9138000
age.s_c[196]	1.0002309	1.0012271	4.522540e+01	45.2769000	46.6210390	20001.0	5.511910e-02	0.95	30.1416000	60.7669000
age.s_c[197]	1.0007145	1.0028165	5.421518e+01	54.1117000	52.3897927	20001.0	5.615110e-02	0.95	38.2325000	69.1771000
age.s_c[198]	0.9998901	0.9999691	2.575846e+01	24.8716000	20.5419527	20001.0	3.496320e-02	0.95	19.0002000	35.1506000
age.s_c[199]	1.0000538	1.0003535	5.803073e+01	57.9732000	57.4468098	20001.0	5.649490e-02	0.95	42.8420000	74.3810000
age.s_c[200]	1.0002942	1.0013039	2.720388e+01	26.3169000	23.2589137	20001.0	3.977440e-02	0.95	19.0019000	37.6232000

2.2 Exact Binomial Test

Table 24: Exact binomial test for CAMSAD dataset (n traits = 64) with calibrated age-at-death estimate.

coverage	n_in	perc	CI_low	CI_up	p_value
0.10	20	0.100	0.062	0.150	1.000
0.20	39	0.195	0.142	0.257	0.930
0.30	51	0.255	0.196	0.321	0.189
0.40	81	0.405	0.336	0.477	0.885
0.50	99	0.495	0.424	0.566	0.944
0.60	117	0.585	0.513	0.654	0.666
0.70	135	0.675	0.605	0.739	0.441
0.80	158	0.790	0.727	0.844	0.724
0.90	178	0.890	0.838	0.930	0.637
0.95	180	0.900	0.850	0.938	0.003

3 Spitalfields

Supplementary information for the Spitalfields dataset (Buckberry & Chamberlain, 2002).

3.1 Diagnostic data

3.1.1 Summarized diagnostic data

Table 25: Spitalfields dataset summarized diagnostics.

	full set	20% missing	30% missing	40% missing	50% missing
PSRF_max	1.291	1.015	1.002	1.019	1.023
PSRF_upper_max	1.303	1.015	1.006	1.020	1.023
ESS_min	13411.200	20247.700	4710.800	16079.500	13890.900

3.1.2 Full set

Table 26: MCMC diagnostic data Spitalfields dataset.

	PSRF Point est.	PSRF Upper C.I.	Mean	Median	Mode	ESS	MCSE	HDImass	HDIlow	HDIhigh
M	1.0003681	1.0007695	64.9653143	67.3770853	70.7310188	31798.1	0.0623859	0.95	43.5794484	81.7655102
Ustar[1, 1]	NaN	NaN	1.0000000	1.0000000	0.9993457	0.0	NaN	0.95	1.0000000	1.0000000
Ustar[2, 1]	NaN	NaN	0.0000000	0.0000000	-0.0006543	0.0	NaN	0.95	0.0000000	0.0000000
Ustar[3, 1]	NaN	NaN	0.0000000	0.0000000	-0.0006543	0.0	NaN	0.95	0.0000000	0.0000000
Ustar[4, 1]	NaN	NaN	0.0000000	0.0000000	-0.0006543	0.0	NaN	0.95	0.0000000	0.0000000
Ustar[5, 1]	NaN	NaN	0.0000000	0.0000000	-0.0006543	0.0	NaN	0.95	0.0000000	0.0000000
Ustar[1, 2]	1.0003375	1.0011444	0.2408449	0.2593509	0.2910211	32013.9	0.0011620	0.95	-0.1832918	0.6347196
Ustar[2, 2]	1.0006091	1.0016380	0.9461494	0.9637376	0.9952659	30922.9	0.0003396	0.95	0.8365431	1.0000000
Ustar[3, 2]	NaN	NaN	0.0000000	0.0000000	-0.0006543	0.0	NaN	0.95	0.0000000	0.0000000
Ustar[4, 2]	NaN	NaN	0.0000000	0.0000000	-0.0006543	0.0	NaN	0.95	0.0000000	0.0000000
Ustar[5, 2]	NaN	NaN	0.0000000	0.0000000	-0.0006543	0.0	NaN	0.95	0.0000000	0.0000000
Ustar[1, 3]	0.9999447	1.0000417	-0.0105597	0.0211694	0.0964914	29530.2	0.0016082	0.95	-0.5610786	0.4739487
Ustar[2, 3]	1.0000954	1.0004797	0.1976280	0.2276833	0.2898517	33381.5	0.0013231	0.95	-0.3357729	0.6548580
Ustar[3, 3]	1.0000084	1.0000291	0.9038349	0.9320838	0.9800702	33951.5	0.0005180	0.95	0.7111784	0.9999991
Ustar[4, 3]	NaN	NaN	0.0000000	0.0000000	-0.0006543	0.0	NaN	0.95	0.0000000	0.0000000
Ustar[5, 3]	NaN	NaN	0.0000000	0.0000000	-0.0006543	0.0	NaN	0.95	0.0000000	0.0000000
Ustar[1, 4]	1.0000684	1.0002581	0.0493767	0.0851122	0.2238961	25618.8	0.0016932	0.95	-0.4902852	0.5089748
Ustar[2, 4]	1.0001456	1.0006021	0.3473660	0.3829081	0.4360425	26807.6	0.0013800	0.95	-0.1402473	0.7447979
Ustar[3, 4]	1.0001961	1.0005299	0.1814289	0.2122591	0.2541127	32946.9	0.0014133	0.95	-0.3674087	0.6731012
Ustar[4, 4]	1.0000357	1.0002432	0.7990299	0.8210082	0.8358265	32263.2	0.0006869	0.95	0.5537191	0.9928429
Ustar[5, 4]	NaN	NaN	0.0000000	0.0000000	-0.0006543	0.0	NaN	0.95	0.0000000	0.0000000
Ustar[1, 5]	1.0000030	1.0001602	-0.2545922	-0.2451185	-0.2562421	28931.4	0.0012914	0.95	-0.6928934	0.1448987
Ustar[2, 5]	1.0001340	1.0005624	0.0748601	0.1159061	0.1837000	24388.7	0.0015242	0.95	-0.4285167	0.4814682
Ustar[3, 5]	0.9999156	0.9999708	-0.2137548	-0.1993949	-0.1183837	30312.4	0.0015599	0.95	-0.8031829	0.2631187
Ustar[4, 5]	1.0001380	1.0003873	-0.1201957	-0.1051932	-0.0924824	31606.6	0.0016733	0.95	-0.7652455	0.3981168
Ustar[5, 5]	1.0000720	1.0001816	0.7487846	0.8103278	0.9293482	33331.9	0.0011172	0.95	0.3196594	0.9958344
a	1.0003843	1.0008100	0.0048734	0.0038127	0.0017696	31757.4	0.0000220	0.95	0.0001234	0.0127114
age.s[1]	0.9999964	1.0000744	20.9927391	18.9567306	15.9368440	34352.0	0.0345375	0.95	15.0000153	33.6843750
age.s[2]	0.9999605	1.0000150	22.5318239	20.3380703	16.2378124	30493.7	0.0420106	0.95	15.0001358	37.0563067
age.s[3]	1.0000103	1.0001242	20.9607010	18.7405490	15.8744064	30338.8	0.0382445	0.95	15.0001882	34.7058740
age.s[4]	1.0000665	1.0003587	42.6415447	41.7311708	39.8164991	26817.2	0.0776974	0.95	19.1335725	67.2161122
age.s[5]	1.0000229	1.0001124	20.7819809	18.7112928	15.9008191	32114.0	0.0367556	0.95	15.0004802	33.3838481
age.s[6]	0.9999916	1.0001437	28.6725591	26.8427222	23.9556204	34352.0	0.0503926	0.95	15.0010699	46.6665271
age.s[7]	1.0000228	1.0002099	59.8701593	59.9534294	59.6144366	31050.4	0.0759698	0.95	33.9743175	85.8740958
age.s[8]	1.0003548	1.0012009	44.4305721	43.0814986	39.4794217	30716.1	0.0737460	0.95	20.8114693	70.4741113
age.s[9]	0.9999396	1.0000569	47.7856306	46.7446127	45.9205298	31802.7	0.0716247	0.95	24.6359779	73.9295737

age.s[10]	0.9999373	1.0000433	59.8895509	59.9990222	61.7290437	31115.0	0.0748421	0.95	34.0857817	85.2487314
age.s[11]	1.0000536	1.0002858	31.5905520	29.7431120	24.9489582	32910.7	0.0582984	0.95	15.0036680	51.5545776
age.s[12]	1.0004629	1.0015640	44.4466424	43.1630180	40.5769111	29436.0	0.0753168	0.95	20.7929441	70.6025282
age.s[13]	1.0000931	1.0004397	44.0798019	43.1423971	41.8777641	31312.3	0.0691981	0.95	20.8932286	68.1290477
age.s[14]	1.0000758	1.0004227	33.9182901	32.1060524	28.6273573	26786.0	0.0759329	0.95	15.0042127	57.0312834
age.s[15]	1.0000039	1.0001330	37.3453881	35.8340489	32.9191040	31211.3	0.0674224	0.95	16.4591504	60.8390283
age.s[16]	1.0001183	1.0002118	36.0806550	34.7799036	30.8206829	27428.0	0.0724991	0.95	15.0550333	57.8233110
age.s[17]	0.9999889	1.0001969	81.0889586	83.4172406	87.7936496	22533.2	0.0822696	0.95	57.0010936	99.9954514
age.s[18]	1.0000254	1.0002677	44.4098298	43.0712487	40.1142767	29172.9	0.0750306	0.95	20.9976833	70.2053514
age.s[19]	1.0000438	1.0002320	59.8455592	59.8995479	58.7118155	30794.0	0.0757376	0.95	34.1065794	85.3013287
age.s[20]	1.0002672	1.0008618	51.4699090	50.7179935	48.2301093	34352.0	0.0665239	0.95	28.5842960	76.4266053
age.s[21]	1.0001011	1.0005270	48.6318309	47.0549012	45.0168275	30555.3	0.0820774	0.95	22.7515491	77.9178945
age.s[22]	1.0001478	1.0006138	66.4769738	66.9266596	68.1375800	30630.8	0.0705510	0.95	41.6780041	89.6950185
age.s[23]	0.9999875	1.0000849	59.8648781	59.9341890	58.1004365	30098.1	0.0762514	0.95	33.3774646	84.8860240
age.s[24]	0.9999667	1.0000442	66.5026823	66.8489308	67.3436996	30393.7	0.0708623	0.95	41.7314132	89.5463887
age.s[25]	0.9999949	1.0001824	65.2449023	65.3120633	64.3222654	30222.7	0.0761974	0.95	40.3001019	91.1872972
age.s[26]	1.0001587	1.0006429	40.7811286	38.8671344	35.9900677	28890.3	0.0764639	0.95	18.1158390	67.6920175
age.s[27]	0.9999413	1.0000184	74.4358415	75.7883349	80.5766920	32130.4	0.0745107	0.95	49.4032886	98.6571547
age.s[28]	0.9999657	1.0001150	59.8051618	59.9066611	60.8172420	30102.5	0.0764076	0.95	34.6811538	86.0140115
age.s[29]	1.0000050	1.0000674	59.8454495	59.9331420	61.0528854	30673.7	0.0760891	0.95	33.6527825	85.1439381
age.s[30]	1.0002085	1.0007184	63.7350236	63.8203269	64.0418229	31693.5	0.0719228	0.95	39.7226976	89.4385747
age.s[31]	1.0000769	1.0004333	70.8254663	71.2318820	72.2668570	31579.7	0.0648992	0.95	48.7528517	93.2311439
age.s[32]	0.9999627	1.0000833	62.7807972	63.0307322	63.2759118	32468.8	0.0760108	0.95	36.3771774	88.3269730
age.s[33]	1.0000645	1.0002637	76.3674736	77.1133241	78.4508282	34352.0	0.0587179	0.95	55.0598853	96.8239170
age.s[34]	1.0000523	1.0001088	75.1747290	76.8267651	82.5563088	23595.7	0.0867322	0.95	49.5819197	98.9193729
age.s[35]	0.9999732	1.0000692	31.5979912	29.8036445	25.8958837	33310.8	0.0581194	0.95	15.0050832	51.7007474
age.s[36]	0.9999537	1.0000454	66.4694485	66.9536520	69.0159459	30716.3	0.0706263	0.95	43.0273853	91.1177114
age.s[37]	1.0000944	1.0003068	45.9649940	44.9528262	40.7334632	22639.2	0.1061303	0.95	16.7117684	75.0711525
age.s[38]	0.9999413	0.9999648	86.3147083	87.5813326	90.0725763	33540.4	0.0475112	0.95	70.2432020	99.9935584
age.s[39]	1.0003196	1.0009534	40.8274518	38.9929152	34.9563342	31064.5	0.0732616	0.95	18.0293666	67.0198982
age.s[40]	0.9999457	1.0000711	52.8885134	51.6033536	46.5905168	34352.0	0.0809665	0.95	25.5331840	82.1498667
age.s[41]	0.9999266	1.0000162	61.5627440	61.2886608	62.4388458	28680.6	0.0887594	0.95	34.2610501	91.1084956
age.s[42]	1.0003102	1.0010781	70.9317713	71.3381058	72.0742755	33160.1	0.0626683	0.95	48.0578700	92.3587004
age.s[43]	0.9999796	1.0001217	65.1899087	65.3582320	65.5712370	28879.9	0.0778446	0.95	39.0282093	89.9246052
age.s[44]	1.0001277	1.0003710	51.4319945	50.8071611	47.9824219	32305.7	0.0684061	0.95	27.8866448	75.5744117
age.s[45]	1.0000379	1.0002537	65.2127524	65.2972006	66.0994756	30404.3	0.0755314	0.95	39.9995920	90.8802327
age.s[46]	1.0001091	1.0003253	59.8261681	59.9261470	61.2310907	30299.0	0.0757682	0.95	34.5098114	85.6309493
age.s[47]	1.0000074	1.0001550	59.8605291	59.9751815	60.2672643	30762.7	0.0759229	0.95	34.3860262	85.6438294
age.s[48]	0.9999968	1.0001884	52.0255773	51.2326012	50.2307647	29295.3	0.0769380	0.95	27.2212472	78.2033151

age.s[49]	1.0001336	1.0004534	71.0161854	71.4261421	72.3593493	34352.0	0.0618946	0.95	48.1321727	92.6023635
age.s[50]	0.9999745	1.0001245	70.8692593	71.2972940	71.7059391	34352.0	0.0616102	0.95	47.9097010	92.2396548
age.s[51]	1.0000620	1.0004088	62.6817386	62.9505208	62.8603924	32671.9	0.0754793	0.95	36.9881653	88.9945504
age.s[52]	1.0000713	1.0003529	71.8955600	72.7447262	74.9374908	28101.0	0.0754599	0.95	47.7481381	96.1045289
age.s[53]	0.9999422	0.9999690	66.4731090	66.8495148	67.6015347	30421.0	0.0706304	0.95	41.7889944	89.6222263
age.s[54]	1.0000755	1.0003760	44.0967360	43.0270254	41.2031224	32716.1	0.0677451	0.95	21.2576442	68.3392885
age.s[55]	1.0000152	1.0002743	55.9739614	55.7708626	52.9452281	30902.0	0.0797932	0.95	29.2888983	82.7984913
age.s[56]	0.9999935	1.0002119	66.9245728	66.9525614	65.0866422	27719.8	0.0826576	0.95	41.2188559	93.0831361
age.s[57]	1.0000093	1.0002551	62.0456913	61.4981758	58.6972664	24939.7	0.0843229	0.95	37.7221716	88.7495827
age.s[58]	0.9999977	1.0001741	40.7704761	39.0124282	34.3885267	28724.1	0.0760479	0.95	18.3922903	67.1796156
age.s[59]	1.0000671	1.0003963	70.8558558	71.1687656	71.5291259	33276.4	0.0623618	0.95	48.0751328	92.3866481
age.s[60]	1.0001605	1.0007022	59.8083753	59.7956965	58.5015323	30565.9	0.0760476	0.95	34.2131598	85.3638742
age.s[61]	1.0000078	1.0001874	66.5572007	66.9860350	68.8227121	31346.1	0.0698145	0.95	42.9667505	90.5572933
age.s[62]	1.0003705	1.0009309	66.4053784	66.8634566	69.6712983	29084.4	0.0724092	0.95	41.5331718	89.3820129
age.s[63]	1.0000067	1.0001441	50.8801806	50.1953972	49.7037972	34352.0	0.0674248	0.95	27.2864204	75.7624719
age.s[64]	0.9999735	0.9999956	52.0721804	51.6290341	50.5166446	29025.0	0.0820480	0.95	25.7431141	79.5231234
age.s[65]	0.9999647	1.0001249	70.8679381	71.2108103	71.1456936	33104.6	0.0629631	0.95	48.6748160	92.7359529
age.s[66]	0.9999758	1.0001110	56.9103164	55.6370412	53.4937286	23984.7	0.0937534	0.95	30.7026892	86.3616943
age.s[67]	1.0000053	1.0000660	52.0443416	51.5823885	51.5713102	30592.9	0.0793091	0.95	25.7120647	79.0255814
age.s[68]	1.0001061	1.0003238	77.8079080	79.1292558	80.9375685	27820.7	0.0715365	0.95	55.7997720	99.8413030
age.s[69]	1.0000697	1.0003183	66.5003048	66.9819808	67.9229811	30636.9	0.0706287	0.95	41.9489954	89.9094461
age.s[70]	1.0000788	1.0004359	64.8255596	65.4313274	66.8834139	29963.9	0.0819183	0.95	37.0024194	91.2967744
age.s[71]	1.0000620	1.0003578	65.3548642	65.4031361	65.5182380	32383.3	0.0682605	0.95	41.7410302	89.7647591
age.s[72]	1.0001806	1.0007651	69.8556984	70.5729143	74.4234331	33792.9	0.0712364	0.95	43.8797288	93.7852354
age.s[73]	1.0001187	1.0004282	67.2565422	67.4213601	67.6193899	27072.5	0.0805426	0.95	42.5969281	93.3597712
age.s[74]	1.0000464	1.0001770	60.1171503	61.5220003	67.3915758	20652.0	0.1195275	0.95	25.7279430	89.6502980
age.s[75]	1.0003104	1.0010659	63.4147437	63.4510129	63.7790546	32030.8	0.0737879	0.95	37.7308634	89.2374442
age.s[76]	1.0000247	1.0002918	76.3102152	77.0262214	78.5595093	33642.0	0.0593586	0.95	55.8116112	97.5443913
age.s[77]	1.0000472	1.0001670	73.2642868	74.0766470	75.7631726	30664.4	0.0675741	0.95	50.2679243	95.9394120
age.s[78]	0.9999394	0.9999605	59.2414961	59.3805452	61.2755394	30024.7	0.0782277	0.95	32.3933947	85.0035853
age.s[79]	0.9999463	1.0000395	62.7674364	63.1004276	64.5484191	31804.2	0.0765803	0.95	37.4792866	89.2762432
age.s[80]	1.0001989	1.0007861	81.8209211	83.2427639	86.0530249	30960.1	0.0617551	0.95	61.6953566	99.9926902
age.s[81]	1.0001430	1.0003963	66.6510343	68.2260386	74.2031269	26133.2	0.0977173	0.95	35.8550504	94.7217544
age.s[82]	1.0002979	1.0005104	89.5762881	90.9263964	93.7344060	34352.0	0.0402957	0.95	75.3744730	99.9991447
age.s[83]	0.9999777	1.0001602	81.5687506	82.4261061	82.4116349	36172.5	0.0521452	0.95	63.6899959	99.9135994
age.s[84]	1.0001771	1.0007292	78.8852441	79.9113512	80.5101606	29946.5	0.0621009	0.95	58.7761557	99.2801227
age.s[85]	1.0001363	1.0002362	70.8935390	71.2566348	70.3973374	33134.7	0.0628698	0.95	48.5911211	92.8073853
age.s[86]	1.0003218	1.0010157	76.3946007	77.1036284	77.9837599	34352.0	0.0590693	0.95	54.9047408	96.8827088
age.s[87]	1.0000064	1.0001494	59.8886976	60.0078613	61.2590329	31390.9	0.0754350	0.95	33.7876400	85.3266576

age.s[88]	1.0000990	1.0004280	78.8209313	79.8287297	82.5664588	29718.8	0.0630414	0.95	58.9800548	99.7038141
age.s[89]	0.9999718	1.0001238	76.4241043	77.1145777	77.7392811	33675.7	0.0594148	0.95	55.5122076	97.2308976
age.s[90]	0.9999512	0.9999897	81.5866957	82.5785788	84.3529396	34352.0	0.0537955	0.95	63.5840123	99.9964716
age.s[91]	0.9999754	1.0000223	78.7600917	79.8478306	80.6544727	30603.4	0.0621346	0.95	58.7881701	99.5897883
age.s[92]	1.0000930	1.0004576	81.6444517	82.6163178	84.0509934	34352.0	0.0535077	0.95	63.7301934	99.9911478
age.s[93]	1.0004252	1.0015150	63.5169751	63.5107072	63.0139761	33182.4	0.0721785	0.95	37.7928557	88.6872380
age.s[94]	1.0001812	1.0005024	76.3701271	77.0227275	77.5072989	34352.0	0.0584800	0.95	55.6279595	97.0985414
age.s[95]	1.0000039	1.0001907	39.4272564	37.4122901	33.2095605	34352.0	0.0740406	0.95	15.2654168	65.8237920
age.s[96]	1.0001084	1.0002575	45.4275811	44.2307974	40.1547390	30475.8	0.0910803	0.95	16.8960355	74.9692993
age.s[97]	1.0000784	1.0003268	32.0615545	29.0428177	23.5644152	27255.1	0.0777272	0.95	15.0015716	57.9334660
age.s[98]	1.0000886	1.0004518	40.6768670	39.3177627	34.3528354	31210.8	0.0711037	0.95	17.9629246	65.0693049
age.s[99]	0.9999561	1.0000970	38.2095289	35.5218061	30.7427954	31503.5	0.0798284	0.95	15.0836655	66.2627079
age.s[100]	0.9999604	1.0000897	26.8755009	24.1355445	17.2785509	29031.9	0.0611213	0.95	15.0010366	47.4968986
age.s[101]	0.9999219	0.9999958	40.0595913	38.8240526	34.0895182	29700.5	0.0776449	0.95	15.8132322	64.7285570
age.s[102]	1.0000598	1.0003670	51.4471501	50.8559103	51.9053782	34352.0	0.0663484	0.95	28.4581942	76.1514465
age.s[103]	0.9999903	1.0000248	66.4898855	66.9543634	67.2037771	32291.8	0.0688144	0.95	42.3010321	90.1116447
age.s[104]	1.0000875	1.0001353	66.5687754	68.0674479	74.0155112	26123.7	0.0976455	0.95	33.9568769	93.0222293
age.s[105]	1.0000287	1.0003035	34.8434239	33.2404109	30.2846381	33541.9	0.0596053	0.95	16.7266803	56.5764065
age.s[106]	0.9999648	1.0000804	40.5664349	39.1535473	37.1325596	31698.4	0.0702346	0.95	18.3764997	65.4831785
age.s[107]	1.0000661	1.0003560	53.0842546	53.1552060	54.0601363	21910.4	0.1091424	0.95	21.9807630	82.7535628
age.s[108]	1.0000346	1.0001973	48.0087376	47.1356687	43.5959365	28843.1	0.0854233	0.95	21.1248542	75.8332843
age.s[109]	1.0000937	1.0003020	39.0800857	37.8238845	36.5509684	24479.6	0.0815048	0.95	15.8810606	62.6042713
age.s[110]	0.9999714	1.0001442	38.1683040	35.5431921	30.6228156	30484.2	0.0809404	0.95	15.1467868	66.1343835
age.s[111]	1.0000300	1.0002990	51.1993595	49.0633315	43.4931430	21273.4	0.1120289	0.95	23.5963593	85.0260080
age.s[112]	1.0000361	1.0001315	28.7872751	26.8964123	23.5905375	34352.0	0.0508837	0.95	15.0007568	46.9249781
age.s[113]	1.0001312	1.0003485	38.1700560	36.5830315	34.1802062	31395.1	0.0676539	0.95	17.0557683	61.6959009
age.s[114]	1.0000302	1.0002131	37.2778816	35.7090251	33.1500438	31052.8	0.0674027	0.95	16.0449977	60.0365219
age.s[115]	1.0002274	1.0006456	59.8442331	59.8359710	58.2890052	32089.3	0.0738155	0.95	34.3337383	85.4633968
age.s[116]	1.0000312	1.0002170	47.9911609	47.1140473	45.4754299	29042.7	0.0851443	0.95	20.8461608	75.3970556
age.s[117]	1.0000507	1.0002870	37.3231632	35.6819676	33.1065482	29989.4	0.0694189	0.95	15.8008022	60.4418654
age.s[118]	1.0000168	1.0001692	68.5349171	69.7294931	69.6470994	23218.6	0.0983026	0.95	40.1706311	96.4979782
age.s[119]	1.0001697	1.0006151	47.8385558	46.7072951	45.8037893	33638.3	0.0698447	0.95	24.3434761	73.6679250
age.s[120]	0.9999344	1.0000080	53.7259625	53.6605952	54.5074129	24145.9	0.0960702	0.95	24.0214045	81.7461785
age.s[121]	1.0002091	1.0006555	55.9715180	55.8302980	56.5714612	31404.6	0.0796505	0.95	29.1492786	82.8955615
age.s[122]	0.9999528	1.0000325	66.9050389	66.9758635	66.3159554	31046.1	0.0778823	0.95	40.9107217	93.0467042
age.s[123]	1.0003207	1.0009068	73.8382413	76.0699972	81.6958003	26700.2	0.0905790	0.95	45.6230347	99.7674023
age.s[124]	1.0001184	1.0003690	73.2603896	73.9311536	73.7924003	29595.1	0.0686309	0.95	49.8499635	95.3299468
age.s[125]	1.0001022	1.0003949	45.4921893	44.1617789	39.1985645	29574.1	0.0926864	0.95	17.2717513	75.5022236
age.s[126]	1.0000174	1.0002923	52.0282851	51.5781884	49.9503443	29809.5	0.0810073	0.95	25.3368694	79.0058874

age.s[127]	1.0001201	1.0005368	57.4940033	57.2364210	57.9261595	29648.8	0.0846411	0.95	29.5814324	85.3578528
age.s[128]	1.0000612	1.0003040	66.4980289	66.8360172	67.6534668	30267.7	0.0709560	0.95	43.3874157	91.5541517
age.s[129]	1.0000616	1.0001672	52.1606125	51.7451810	50.8320767	29323.9	0.0809745	0.95	26.1683916	79.3962592
age.s[130]	1.0000924	1.0002365	83.1316838	84.3678012	87.2813509	29940.3	0.0567013	0.95	65.0632160	99.9999453
age.s[131]	0.9999743	1.0000604	59.8235112	59.8321231	56.5761766	31412.2	0.0749429	0.95	33.7295626	85.1075901
age.s[132]	0.9999904	1.0001046	74.0103651	75.1611833	77.8076417	30401.5	0.0732208	0.95	49.3216442	97.8624819
age.s[133]	0.9999568	1.0000525	70.9453651	71.3819701	72.1655931	32384.6	0.0636558	0.95	48.8005617	93.3295776
age.s[134]	0.9999471	0.9999914	61.5581315	61.2690394	60.6532787	29201.4	0.0874812	0.95	33.8676063	90.4242672
age.s[135]	1.0000168	1.0002067	59.8679114	59.9040012	60.2352293	30019.7	0.0765352	0.95	34.1635325	85.5183069
age.s[136]	1.0000088	1.0001898	59.8444611	59.9235532	58.1729138	32206.7	0.0740576	0.95	33.8980417	85.3597210
age.s[137]	0.9999143	0.9999719	51.4367898	50.6595657	49.2723323	34352.0	0.0665690	0.95	28.1582209	75.9071298
age.s[138]	1.0000843	1.0004134	40.7522850	38.8559382	35.9333333	29260.1	0.0753210	0.95	18.5306623	67.0528398
age.s[139]	1.0000398	1.0003465	55.0136340	53.8673986	50.0554854	25808.4	0.0864789	0.95	30.1328409	83.6535088
age.s[140]	1.0001030	1.0003679	70.9098645	71.2706012	70.9675997	32447.0	0.0635630	0.95	48.5658303	92.8995856
age.s[141]	1.0001450	1.0003603	76.3472591	76.9973776	78.5050887	34352.0	0.0588539	0.95	55.4109163	97.1479181
age.s[142]	1.0001160	1.0005243	76.3188763	76.9508197	76.9787446	34352.0	0.0588362	0.95	54.9808783	96.6756724
age.s[143]	1.0000114	1.0002123	70.9510256	71.3665500	72.8447673	32163.2	0.0641354	0.95	48.1711581	92.7035957
age.s[144]	0.9999964	1.0002000	46.0077153	44.9986035	44.2786527	22268.3	0.1066960	0.95	16.6470985	74.7870816
age.s[145]	0.9999679	1.0001274	58.1205712	57.7147850	57.5755831	34352.0	0.0652523	0.95	35.0511454	82.0507124
age.s[146]	0.9999977	1.0000679	66.5112417	66.9838496	68.5009914	31500.8	0.0693026	0.95	41.3457785	88.9859198
age.s[147]	0.9999924	1.0000572	50.9474328	50.2394937	49.9181718	34352.0	0.0674513	0.95	26.1010804	74.8776776
age.s[148]	1.0000406	1.0003429	63.7360022	63.2121529	58.2092723	24753.8	0.0922420	0.95	37.3871564	92.3644537
age.s[149]	0.9999377	1.0000112	83.1424363	84.3938954	86.9317174	32780.3	0.0541383	0.95	65.0581421	99.9991551
age.s[150]	1.0000446	1.0003330	78.8230755	79.8756586	82.3346247	31022.0	0.0618209	0.95	58.3340770	99.1580478
age.s[151]	0.9999652	1.0000790	81.6030461	82.5236825	85.3806824	33478.2	0.0544982	0.95	63.5874781	99.9699205
age.s[152]	1.0001693	1.0007075	77.0309478	77.6265450	77.7498797	33784.5	0.0587822	0.95	56.4775193	97.5154675
age.s[153]	1.0002365	1.0009587	59.8840057	59.9318077	60.7118301	29271.5	0.0776254	0.95	34.5563237	86.0019373
age.s[154]	0.9999773	1.0000128	75.1422048	76.9012344	81.7114413	22471.9	0.0886702	0.95	49.0527026	98.4367348
age.s[155]	0.9999590	1.0000048	62.8552033	63.0651906	61.9488917	32867.4	0.0752656	0.95	35.8455760	88.1119251
age.s[156]	1.0000087	1.0000836	71.9674494	72.6728253	72.9066829	32206.6	0.0707711	0.95	47.4455865	95.7915726
age.s[157]	0.9999552	1.0000834	59.8039916	59.8483475	59.3621582	31220.9	0.0750987	0.95	33.7752597	85.1057873
age.s[158]	0.9999239	0.9999892	78.8255386	79.8267408	81.0137128	30082.9	0.0623415	0.95	58.0313805	98.5436904
age.s[159]	0.9999628	1.0000232	71.8650796	72.7407130	73.8813751	29370.8	0.0745095	0.95	47.4456200	96.1181621
age.s[160]	1.0000524	1.0002629	86.2969812	87.5469219	90.6581522	31117.6	0.0495416	0.95	70.0363903	99.9980288
age.s[161]	0.9999832	1.0000031	65.2348125	65.4918760	67.5059629	31070.2	0.0747976	0.95	40.0877744	90.6288100
age.s[162]	1.0000603	1.0001123	83.1643604	84.4766534	87.3183922	31103.3	0.0557269	0.95	64.8217726	99.9877431
age.s[163]	1.0000458	1.0003448	62.1257347	61.5221248	59.3207072	23238.1	0.0873575	0.95	37.0086430	88.3189317
age.s[164]	1.0000214	1.0002796	67.4544795	68.0875754	70.8370148	31489.1	0.0736410	0.95	42.4420433	92.9001216
age.s[165]	0.9999992	1.0001365	66.5413498	67.0057807	69.9593961	31400.2	0.0696830	0.95	41.8884247	89.7599209

age.s[166]	1.0001472	1.0003011	66.4890871	66.9571505	67.6255885	31050.5	0.0703176	0.95	42.1197287	90.0913315
age.s[167]	0.9999574	0.9999990	73.4444616	75.4049696	83.1056976	23054.4	0.0979476	0.95	45.5791839	99.4862236
age.s[168]	0.9999960	1.0002144	59.7723828	59.8355420	60.1373677	31781.9	0.0747996	0.95	33.8061201	85.6103178
age.s[169]	1.0002734	1.0008668	70.8784769	71.2873332	73.4267922	30853.7	0.0649592	0.95	48.9060852	93.1407430
age.s[170]	1.0000638	1.0002920	76.9998142	77.6406156	78.4238110	32911.9	0.0592816	0.95	56.3549351	97.5744084
age.s[171]	0.9999907	1.0001605	66.4861997	66.8401295	68.4136811	29510.6	0.0720185	0.95	41.5095647	89.3653366
age.s[172]	0.9999296	0.9999951	58.1500170	57.7755286	57.4936289	33267.5	0.0661120	0.95	35.4875221	82.5309836
age.s[173]	1.0000440	1.0003563	52.3951432	51.6259951	48.1831207	30460.0	0.0808904	0.95	26.1713200	80.6577004
age.s[174]	0.9999635	1.0000310	53.0417329	52.6804958	53.4004852	31525.6	0.0892953	0.95	23.0494764	82.5261375
age.s[175]	1.0000002	1.0000417	78.8603435	79.8946402	81.7019644	31007.2	0.0614715	0.95	58.4794611	99.0116298
age.s[176]	0.9999790	1.0000186	71.8790317	72.8281318	74.9997653	29395.9	0.0739002	0.95	47.3936269	95.5523727
age.s[177]	1.0000024	1.0002165	51.4409903	50.6538129	49.3278326	34352.0	0.0664639	0.95	27.6648852	75.7273163
age.s[178]	1.0000366	1.0001159	59.8649245	59.9504577	60.0846596	31303.6	0.0753246	0.95	33.8986586	85.5362382
age.s[179]	1.0001488	1.0004845	71.8219352	72.5269833	73.8398934	32206.3	0.0707709	0.95	47.3327398	95.4314683
age.s[180]	0.9999270	0.9999687	81.6471639	82.5581151	84.6459685	32428.1	0.0550164	0.95	63.5200277	99.6523028
b	0.9999731	1.0001364	0.0499412	0.0485921	0.0463564	32169.8	0.0000742	0.95	0.0253308	0.0768359
beta[1]	1.0006794	1.0013595	3.7898152	3.1831554	2.2311242	14810.6	0.0171527	0.95	0.9231884	8.1556355
beta[2]	1.0002878	1.0007009	3.3349926	2.7400191	2.2459335	13411.2	0.0158914	0.95	1.0572925	7.5610526
beta[3]	1.0001684	1.0002130	1.6622973	1.5022047	1.3198953	31658.5	0.0046785	0.95	0.4746276	3.1551700
beta[4]	1.0003516	1.0010086	3.2995963	3.0798781	2.5691664	33017.1	0.0073362	0.95	1.0768921	6.0290049
beta[5]	1.0001093	1.0003921	1.7242806	1.6025124	1.4094058	32598.1	0.0042309	0.95	0.4300622	3.1963294
beta0[1]	1.0005032	1.0011033	-10.3249152	-8.5012537	-5.2879741	15496.6	0.0543774	0.95	-24.2939544	-0.6228112
beta0[2]	1.0003165	1.0008015	-9.1380335	-7.3408718	-5.5857288	14197.5	0.0498865	0.95	-22.5310665	-1.1944899
beta0[3]	1.0000840	1.0001134	-4.4181367	-3.8185019	-3.2202039	31703.0	0.0175609	0.95	-9.8940129	-0.0009385
beta0[4]	1.0003454	1.0010207	-12.9854933	-12.0445782	-9.9594506	33095.0	0.0305417	0.95	-24.4629253	-3.8877263
beta0[5]	1.0000903	1.0003852	-5.5541080	-5.0410361	-4.3487962	32905.2	0.0170538	0.95	-11.5998069	-0.4329256
thresh_age[1, 1]	0.9999941	1.0002096	16.3246762	16.2083893	16.5846626	28191.4	0.0336973	0.95	5.0645121	27.7078700
thresh_age[2, 1]	1.0001336	1.0005670	17.2488042	16.7874594	16.2965105	31905.0	0.0309129	0.95	6.7504724	28.7139401
thresh_age[3, 1]	0.9999566	1.0000625	17.8943317	17.5247945	17.6302114	33177.2	0.0409460	0.95	3.0622271	31.4607127
thresh_age[4, 1]	1.0001028	1.0003531	59.3824948	59.6623995	59.9147425	33295.9	0.0431859	0.95	43.7825787	73.8198921
thresh_age[5, 1]	1.0001373	1.0006470	31.6284899	31.4628722	31.4581302	34352.0	0.0489916	0.95	13.8885560	50.7835642
thresh_age[1, 2]	1.0000637	1.0002314	35.6061563	35.3960067	35.4898233	25524.6	0.0553745	0.95	19.0155841	52.9541589
thresh_age[2, 2]	1.0001664	1.0007539	30.9080776	30.2118195	28.3092266	32194.0	0.0429688	0.95	17.0368677	47.0446608
thresh_age[3, 2]	0.9999237	0.9999808	32.9090797	32.6115833	32.2288463	32451.6	0.0465025	0.95	16.2227979	49.5523502
thresh_age[4, 2]	1.2905282	1.3029688	98.1520220	90.2007037	50.7562062	34352.0	2.6471901	0.95	67.4841609	123.1147097
thresh_age[5, 2]	1.0310384	1.0314659	152.7260913	125.3260381	112.9784073	32912.6	0.7919222	0.95	73.2173426	275.1674669
thresh_age[1, 3]	1.0000475	1.0001790	47.9921444	48.1288808	48.7953424	26512.3	0.0536080	0.95	30.6810241	63.8932337
thresh_age[2, 3]	1.0000511	1.0001986	73.3711838	73.5205744	75.4765607	27525.4	0.0534133	0.95	55.3992287	90.0345060
thresh_age[3, 3]	NA	NA	0.0000000	0.0000000	-0.0006543	0.0	NaN	0.95	0.0000000	0.0000000

thresh_age[4, 3]	NA	NA	0.0000000	0.0000000	-0.0006543	0.0	NaN	0.95	0.0000000	0.0000000
thresh_age[5, 3]	NA	NA	0.0000000	0.0000000	-0.0006543	0.0	NaN	0.95	0.0000000	0.0000000
thresh_age[1, 4]	1.0002743	1.0005527	99.3327636	94.8391899	89.2453578	24030.1	0.1183694	0.95	73.3317268	134.8955791
thresh_age[2, 4]	1.0022804	1.0032291	161.5873022	148.5408787	134.6932609	25595.5	0.3937585	0.95	89.4209143	267.7110202
thresh_age[3, 4]	NA	NA	0.0000000	0.0000000	-0.0006543	0.0	NaN	0.95	0.0000000	0.0000000
thresh_age[4, 4]	NA	NA	0.0000000	0.0000000	-0.0006543	0.0	NaN	0.95	0.0000000	0.0000000
thresh_age[5, 4]	NA	NA	0.0000000	0.0000000	-0.0006543	0.0	NaN	0.95	0.0000000	0.0000000
thresh_age_log[1, 1]	1.0001645	1.0004734	2.7229759	2.7855290	2.8402578	26887.6	0.0024285	0.95	1.9204853	3.4642849
thresh_age_log[2, 1]	1.0000909	1.0004970	2.7934317	2.8206321	2.8519673	31930.3	0.0019112	0.95	2.1037407	3.4462811
thresh_age_log[3, 1]	0.9999179	0.9999891	2.7831291	2.8636167	2.9760098	32614.1	0.0026704	0.95	1.7558083	3.6691032
thresh_age_log[4, 1]	1.0000310	1.0002858	4.0749208	4.0887020	4.1227741	33207.4	0.0007473	0.95	3.8059115	4.3218394
thresh_age_log[5, 1]	1.0001864	1.0006678	3.4062957	3.4488082	3.5008744	34352.0	0.0017674	0.95	2.7673562	3.9952011
thresh_age_log[1, 2]	1.0001335	1.0003785	3.5392346	3.5665990	3.5872904	25804.8	0.0016502	0.95	3.0299460	4.0147273
thresh_age_log[2, 2]	1.0001962	1.0008213	3.3992485	3.4082332	3.4369550	32360.2	0.0014205	0.95	2.9225406	3.9022031
thresh_age_log[3, 2]	0.9999382	1.0000128	3.4592032	3.4846675	3.5117874	32821.5	0.0014891	0.95	2.8955537	3.9600102
thresh_age_log[4, 2]	1.0144815	1.0152392	4.5203536	4.5020372	4.4837468	33456.5	0.0009439	0.95	4.2392100	4.8366146
thresh_age_log[5, 2]	1.0005034	1.0008556	4.9201123	4.8309186	4.7110538	30979.8	0.0021645	0.95	4.3915972	5.6728986
thresh_age_log[1, 3]	1.0001026	1.0002840	3.8535151	3.8738824	3.9348249	26774.9	0.0011651	0.95	3.4810021	4.1937919
thresh_age_log[2, 3]	1.0000822	1.0002721	4.2881403	4.2975653	4.3294034	27782.9	0.0007338	0.95	4.0316283	4.5141122
thresh_age_log[3, 3]	NA	NA	0.0000000	0.0000000	-0.0006543	0.0	NaN	0.95	0.0000000	0.0000000
thresh_age_log[4, 3]	NA	NA	0.0000000	0.0000000	-0.0006543	0.0	NaN	0.95	0.0000000	0.0000000
thresh_age_log[5, 3]	NA	NA	0.0000000	0.0000000	-0.0006543	0.0	NaN	0.95	0.0000000	0.0000000
thresh_age_log[1, 4]	1.0001105	1.0004121	4.5844615	4.5521827	4.4975090	22792.7	0.0010653	0.95	4.3185288	4.9222542
thresh_age_log[2, 4]	1.0005119	1.0014321	5.0323968	5.0008602	5.0067649	21800.0	0.0020728	0.95	4.5196924	5.6049944
thresh_age_log[3, 4]	NA	NA	0.0000000	0.0000000	-0.0006543	0.0	NaN	0.95	0.0000000	0.0000000
thresh_age_log[4, 4]	NA	NA	0.0000000	0.0000000	-0.0006543	0.0	NaN	0.95	0.0000000	0.0000000
thresh_age_log[5, 4]	NA	NA	0.0000000	0.0000000	-0.0006543	0.0	NaN	0.95	0.0000000	0.0000000

3.1.3 20% missing

Table 27: MCMC diagnostic data Spitalfields dataset (20 % missing).

	PSRF Point est.	PSRF Upper C.I.	Mean	Median	Mode	ESS	MCSE	HDImass	HDIlow	HDIhigh
b	1.0001171	1.0002717	0.0479325	0.0473277	0.0465228	42580.3	0.0000462	0.95	0.0294124	0.0668391
a	1.0001682	1.0002222	0.0048038	0.0041486	0.0029455	44287.6	0.0000142	0.95	0.0003864	0.0106344
M	1.0002047	1.0002651	64.9460195	66.4355000	69.3870835	44046.0	0.0396256	0.95	48.3779000	78.3154000
beta0[1]	1.0003120	1.0004256	-8.3380750	-7.8612400	-7.2133555	34774.6	0.0156113	0.95	-14.2058000	-3.3484200
beta0[2]	1.0000772	1.0001416	-11.3153599	-10.7268000	-9.6681645	24847.7	0.0236848	0.95	-18.9065000	-4.9612500
beta0[3]	1.0000548	1.0003046	-5.8555838	-5.6831200	-5.4800464	47632.3	0.0083046	0.95	-9.4887700	-2.5047800
beta0[4]	1.0002546	1.0004135	-18.7214055	-18.1393000	-16.7646237	39363.4	0.0272197	0.95	-29.2909000	-8.8353200
beta0[5]	1.0001185	1.0005141	-3.8542339	-3.7375500	-3.4518587	49871.3	0.0071572	0.95	-7.0416200	-0.8407260
beta[1]	1.0002785	1.0003593	3.1428953	3.0152900	2.7884074	32146.1	0.0045235	0.95	1.7655500	4.7899600
beta[2]	1.0001328	1.0002387	4.0186207	3.8415900	3.4966674	22484.3	0.0073884	0.95	2.1320800	6.2322000
beta[3]	1.0000911	1.0004317	2.0984290	2.0591400	1.9959226	47901.7	0.0021167	0.95	1.2403200	3.0285100
beta[4]	1.0002856	1.0004457	4.7032794	4.5715800	4.2301186	39312.8	0.0064280	0.95	2.4413600	7.2765900
beta[5]	1.0001006	1.0004612	1.3095654	1.2837600	1.2381366	50119.4	0.0017289	0.95	0.5455730	2.0551200
thresh[1,1]	NaN	NaN	0.5000000	0.5000000	0.4996965	0.0	NaN	0.95	0.5000000	0.5000000
thresh[2,1]	NaN	NaN	0.5000000	0.5000000	0.4996965	0.0	NaN	0.95	0.5000000	0.5000000
thresh[3,1]	NaN	NaN	0.5000000	0.5000000	0.4996965	0.0	NaN	0.95	0.5000000	0.5000000
thresh[4,1]	NaN	NaN	0.5000000	0.5000000	0.4996965	0.0	NaN	0.95	0.5000000	0.5000000
thresh[5,1]	NaN	NaN	0.5000000	0.5000000	0.4996965	0.0	NaN	0.95	0.5000000	0.5000000
thresh[1,2]	1.0003106	1.0003778	2.9485854	2.8480700	2.6392853	30882.4	0.0039068	0.95	1.7923700	4.3283400
thresh[2,2]	0.9999508	0.9999785	2.5858879	2.4692200	2.2368963	24966.7	0.0044556	0.95	1.3988500	3.9877000
thresh[3,2]	1.0000518	1.0003074	1.7194840	1.7008000	1.6748599	50556.8	0.0010458	0.95	1.2744400	2.1837400
thresh[4,2]	1.0001483	1.0001979	2.1322456	2.0921700	1.9804808	42566.4	0.0015253	0.95	1.5790600	2.7773500
thresh[5,2]	1.0000487	1.0002636	2.6995644	2.6934300	2.6833726	50001.0	0.0008702	0.95	2.3227700	3.0824900
thresh[1,3]	1.0002126	1.0002943	3.8014723	3.6869400	3.5213252	29878.8	0.0044240	0.95	2.4875000	5.3077500
thresh[2,3]	1.0001827	1.0003415	5.8385813	5.6154800	5.2567792	20247.7	0.0086083	0.95	3.8465500	8.3152300
thresh[1,4]	1.0001710	1.0002039	5.9801750	5.8472300	5.6239217	29516.3	0.0052434	0.95	4.4252300	7.7552800
thresh[2,4]	1.0004144	1.0006318	8.0113691	7.7563900	7.3903052	20832.4	0.0099265	0.95	5.6261100	10.8746000
thresh_age[1,1]	1.0000871	1.0002369	16.4140633	16.0190000	15.2799697	46619.7	0.0189555	0.95	8.9368000	24.7780000
thresh_age[2,1]	1.0000219	1.0001120	18.7745881	18.3377000	17.7090060	46178.8	0.0180709	0.95	11.6388000	26.7466000
thresh_age[3,1]	0.9999670	1.0000301	20.4414280	20.0465000	18.8776891	46335.4	0.0229588	0.95	11.0247000	30.3748000
thresh_age[4,1]	1.0000350	1.0001037	59.2068001	59.4972000	61.0779940	43720.6	0.0289718	0.95	47.2778000	70.9271000
thresh_age[5,1]	1.0001231	1.0006076	26.8701088	26.9335000	26.4047051	48388.2	0.0338078	0.95	11.6759000	41.1674000
thresh_age[1,2]	1.0001007	1.0001628	35.6084314	35.3288000	34.6229597	40484.4	0.0311298	0.95	23.6892000	48.1817000
thresh_age[2,2]	0.9999975	1.0001076	31.5429223	31.1525000	30.6294696	42012.8	0.0273701	0.95	21.0249000	42.7295000
thresh_age[3,2]	1.0000083	1.0001845	36.6067364	36.3575000	35.6698012	44847.9	0.0276224	0.95	25.5313000	48.4743000

thresh_age[4,2]	1.0000011	1.0000692	84.8674759	84.8028000	84.7933157	46285.8	0.0305795	0.95	71.4326000	97.6811000
thresh_age[5,2]	1.0150911	1.0152928	172.5931881	149.3160000	141.4221749	50001.0	0.4817172	0.95	91.9846000	301.8210000
thresh_age[1,3]	1.0000556	1.0001183	46.8636080	46.8410000	47.0648591	41021.4	0.0316311	0.95	34.2914000	59.3379000
thresh_age[2,3]	1.0000220	1.0002240	71.5326774	71.8432000	71.9593293	44382.8	0.0289668	0.95	59.1557000	83.0473000
thresh_age[1,4]	0.9999834	1.0000986	96.2197297	95.4823000	95.3702315	47226.9	0.0399088	0.95	79.6990000	113.6820000
thresh_age[2,4]	1.0005903	1.0006984	127.1533890	123.6600000	118.3523280	34692.5	0.1034813	0.95	95.6867000	165.1240000
age.s[1]	1.0000031	1.0001180	18.3896040	17.4623000	15.5871253	50001.0	0.0143464	0.95	15.0000000	24.7225000
age.s[2]	1.0002216	1.0007726	20.2331227	19.0199000	15.9767249	47299.0	0.0210984	0.95	15.0000000	29.3379000
age.s[3]	1.0001472	1.0003907	20.3517150	19.2658000	16.0956499	47888.5	0.0202306	0.95	15.0002000	29.0521000
age.s[4]	1.0000903	1.0002533	33.9383383	33.0972000	31.3611782	44673.7	0.0425181	0.95	17.7480000	51.7734000
age.s[5]	1.0000632	1.0000821	19.9587709	18.8672000	16.0139180	50001.0	0.0191759	0.95	15.0002000	28.3988000
age.s[6]	1.0001871	1.0004122	27.5359736	26.5413000	25.0908940	47599.3	0.0338660	0.95	15.0005000	41.2983000
age.s[7]	1.0000721	1.0003820	62.2787474	62.3764000	62.8629127	48275.6	0.0593134	0.95	37.1978000	87.5613000
age.s[8]	1.0000074	1.0000635	46.6551701	45.7360000	43.1218933	48067.3	0.0549543	0.95	23.9563000	69.9668000
age.s[9]	1.0000167	1.0001055	46.6755589	46.0023000	45.5630235	44646.8	0.0545143	0.95	25.0226000	69.3664000
age.s[10]	1.0000640	1.0000818	55.2508825	55.0389000	53.6112294	48065.1	0.0530530	0.95	32.8929000	77.9601000
age.s[11]	1.0000708	1.0001411	27.3238818	26.1003000	23.3619394	47159.5	0.0355347	0.95	15.0032000	41.7928000
age.s[12]	0.9999746	1.0000373	41.3641062	40.6870000	38.6383639	48626.7	0.0491314	0.95	20.7619000	62.2753000
age.s[13]	1.0000220	1.0000641	48.8004253	48.4059000	47.1296867	48105.6	0.0498586	0.95	27.7546000	70.0644000
age.s[14]	0.9999746	1.0000020	24.6480750	23.2234000	20.3360502	48559.3	0.0321400	0.95	15.0001000	38.2959000
age.s[15]	1.0000434	1.0002814	43.0900263	42.4444000	40.6783954	48619.7	0.0484546	0.95	22.8309000	63.9036000
age.s[16]	1.0000192	1.0001663	28.4840256	27.1973000	24.1787667	48639.1	0.0381359	0.95	15.0059000	44.3572000
age.s[17]	1.0000139	1.0001182	75.3404154	76.0387000	78.3308359	48179.6	0.0531663	0.95	53.5300000	97.7744000
age.s[18]	1.0000727	1.0003857	43.0575310	42.4758000	40.6845384	47251.3	0.0492770	0.95	23.0399000	63.9471000
age.s[19]	0.9999528	0.9999810	62.2323458	62.2453000	61.9626386	48299.3	0.0591989	0.95	36.4324000	86.7314000
age.s[20]	1.0001248	1.0005480	53.4153676	52.9312000	52.8083698	49312.0	0.0559694	0.95	30.2636000	78.0118000
age.s[21]	1.0000025	1.0001246	40.3008664	39.5262000	38.1858000	48466.9	0.0525111	0.95	18.4151000	62.5092000
age.s[22]	1.0001635	1.0006990	65.0086836	65.1386000	63.9153610	47386.4	0.0505967	0.95	43.2629000	86.1543000
age.s[23]	0.9999863	1.0000296	54.1148665	53.9251000	53.6719772	49371.3	0.0528144	0.95	31.1442000	76.6019000
age.s[24]	1.0000907	1.0004134	69.4713615	69.7084000	70.3957324	50001.0	0.0514149	0.95	47.2401000	91.9755000
age.s[25]	0.9999612	1.0000004	64.7628440	64.7408000	62.4842066	46742.2	0.0519648	0.95	43.0795000	86.7226000
age.s[26]	1.0000340	1.0001270	37.8867354	37.0781000	35.3241876	46487.2	0.0462387	0.95	19.4425000	57.5448000
age.s[27]	1.0000302	1.0002597	76.0771269	76.5314000	76.2983276	48489.7	0.0478179	0.95	55.9142000	96.4721000
age.s[28]	1.0000514	1.0003358	62.2252492	62.2926000	61.8156213	48792.2	0.0590360	0.95	37.0670000	87.3517000
age.s[29]	0.9999534	0.9999752	52.3894262	52.0746000	51.0427549	47675.5	0.0567853	0.95	28.9769000	76.9219000
age.s[30]	0.9999559	0.9999604	70.8109667	71.2187000	71.9563580	50001.0	0.0534153	0.95	47.5377000	93.7302000
age.s[31]	1.0000118	1.0000320	69.5543996	69.7799000	70.0725315	47611.4	0.0524780	0.95	47.8282000	92.3437000
age.s[32]	1.0001526	1.0007054	54.4414088	54.0124000	53.2488096	47966.1	0.0586671	0.95	29.9861000	79.2938000
age.s[33]	0.9999927	1.0000583	80.4609775	81.0718000	82.4803217	49113.6	0.0432811	0.95	63.0623000	99.0913000

age.s[34]	1.0000084	1.0001086	72.2546069	72.5525000	72.8923352	46951.6	0.0497562	0.95	51.0426000	92.8030000
age.s[35]	0.9999764	1.0000717	33.3176460	32.0890000	29.2704193	47103.3	0.0453937	0.95	15.9906000	52.2935000
age.s[36]	1.0000294	1.0002443	65.3269960	65.3997000	65.8873601	48434.6	0.0522339	0.95	42.8464000	87.6822000
age.s[37]	1.0000536	1.0002198	42.3504969	41.5584000	39.9820339	42619.7	0.0526729	0.95	21.9823000	63.4270000
age.s[38]	1.0000953	1.0004024	87.1755765	88.3640000	91.8927695	48913.5	0.0374793	0.95	71.7286000	99.9996000
age.s[39]	1.0000636	1.0000672	45.3680030	44.7501000	42.7008072	48143.5	0.0543937	0.95	22.9307000	68.4103000
age.s[40]	0.9999800	1.0000451	59.1877288	59.1261000	58.5071337	47970.1	0.0495333	0.95	38.2028000	80.5079000
age.s[41]	1.0000185	1.0000924	59.2902819	59.1354000	59.0805593	44334.7	0.0566179	0.95	36.2657000	82.5730000
age.s[42]	1.0002460	1.0010317	73.8958639	74.1313000	73.0039967	49492.9	0.0466132	0.95	53.8029000	94.1623000
age.s[43]	1.0000236	1.0001921	74.2719303	74.8242000	75.4639781	48667.4	0.0521363	0.95	52.3601000	96.3329000
age.s[44]	0.9999749	1.0000244	47.3535804	46.9826000	45.7991002	47377.8	0.0506052	0.95	26.2612000	69.0433000
age.s[45]	1.0000537	1.0000652	65.0858676	65.0494000	64.0436051	46649.4	0.0545674	0.95	42.4494000	88.1952000
age.s[46]	1.0001389	1.0006492	55.1900733	54.9270000	53.3512384	47581.0	0.0530466	0.95	33.1157000	78.0327000
age.s[47]	1.0000595	1.0002192	52.4317831	52.2023000	52.3829225	47943.6	0.0566883	0.95	29.0927000	76.9597000
age.s[48]	1.0000475	1.0003165	46.6810505	45.7957000	45.0073715	49019.6	0.0547406	0.95	24.5889000	70.9253000
age.s[49]	0.9999728	0.9999865	74.2281141	74.8124000	76.1737924	49034.8	0.0519200	0.95	52.5993000	96.7374000
age.s[50]	1.0000049	1.0001539	73.9965818	74.2855000	74.8305831	49185.7	0.0465926	0.95	54.2361000	94.2085000
age.s[51]	1.0000013	1.0000876	64.9997373	65.0438000	65.4841575	48725.7	0.0500958	0.95	43.6510000	86.5818000
age.s[52]	1.0000651	1.0002557	78.6615742	79.3792000	81.4758289	48650.2	0.0476814	0.95	59.7988000	99.4228000
age.s[53]	1.0000850	1.0004618	65.0823949	65.1854000	63.5778654	44800.7	0.0523775	0.95	43.5825000	86.6897000
age.s[54]	1.0001160	1.0005327	47.7674003	47.2807000	46.7906003	48829.5	0.0554654	0.95	24.2835000	71.4092000
age.s[55]	1.0000396	1.0002494	49.4650826	49.1947000	49.4993320	46428.3	0.0525008	0.95	27.8282000	71.5221000
age.s[56]	0.9999904	1.0000351	63.3130524	63.3602000	63.7317812	47935.1	0.0533938	0.95	40.4615000	86.0400000
age.s[57]	1.0000178	1.0001880	67.7875515	67.8718000	68.4121418	48185.4	0.0479808	0.95	47.5337000	88.7473000
age.s[58]	1.0000466	1.0002600	40.2659936	39.2824000	37.2200132	47416.5	0.0498948	0.95	20.4181000	61.6965000
age.s[59]	0.9999809	1.0000749	74.1857123	74.6711000	74.5645247	48834.6	0.0518976	0.95	52.2807000	96.4710000
age.s[60]	0.9999530	0.9999827	55.2644090	55.0537000	55.1844196	47938.7	0.0530057	0.95	32.7239000	77.8378000
age.s[61]	1.0000174	1.0000434	65.0749057	65.1661000	63.8716680	48453.3	0.0504414	0.95	43.5558000	86.9196000
age.s[62]	0.9999890	1.0000897	69.5433389	69.7227000	71.5116855	47891.9	0.0522408	0.95	47.6325000	92.1234000
age.s[63]	1.0000625	1.0002287	56.3026385	56.1987000	55.5037999	47632.3	0.0494899	0.95	35.2909000	77.1911000
age.s[64]	0.9999504	0.9999753	52.9698146	52.6927000	51.7998538	46366.2	0.0587213	0.95	28.2988000	77.5004000
age.s[65]	1.0001051	1.0002714	69.5253544	69.7563000	68.2950711	48964.0	0.0519261	0.95	46.9519000	91.7297000
age.s[66]	1.0002122	1.0009406	65.0683184	65.1380000	65.9341554	45216.8	0.0552568	0.95	41.7925000	87.6959000
age.s[67]	1.0000996	1.0005035	52.8931621	52.5618000	51.2302208	46560.5	0.0584721	0.95	28.4895000	77.3509000
age.s[68]	1.0000322	1.0001506	83.0054574	83.7661000	85.2174386	48984.6	0.0411857	0.95	66.7509000	99.9949000
age.s[69]	0.9999537	0.9999691	65.7268237	65.7856000	65.5682762	48416.6	0.0501519	0.95	43.9867000	86.9961000
age.s[70]	1.0000179	1.0002213	57.5805710	57.3415000	53.8401059	48252.0	0.0597915	0.95	31.9295000	82.6962000
age.s[71]	1.0000094	1.0001630	68.6460008	68.7893000	68.8828225	48850.1	0.0515971	0.95	46.1854000	90.5783000
age.s[72]	1.0001026	1.0004948	58.9967121	58.8302000	59.2250705	48381.1	0.0586821	0.95	34.4064000	84.1925000

age.s[73]	1.0000293	1.0002595	81.8953511	82.6267000	83.5259888	48790.2	0.0433690	0.95	64.8474000	99.9968000
age.s[74]	1.0001034	1.0002758	45.1219630	44.0244000	40.4540342	42667.6	0.0585530	0.95	23.7133000	70.0042000
age.s[75]	0.9999826	0.9999853	72.1233602	72.5373000	73.6586261	49343.3	0.0517708	0.95	49.4228000	94.1458000
age.s[76]	0.9999899	1.0000154	80.5217429	81.0921000	81.5263307	48900.8	0.0433586	0.95	63.1922000	99.2132000
age.s[77]	1.0000424	1.0002843	72.6243037	72.9402000	73.0776283	48716.8	0.0507275	0.95	50.7893000	93.9345000
age.s[78]	1.0000412	1.0002687	62.1101789	62.1517000	61.4890756	48116.4	0.0510555	0.95	40.0738000	83.9066000
age.s[79]	1.0001258	1.0006069	60.5821002	60.5640000	60.5468899	47433.1	0.0504726	0.95	38.9818000	81.9506000
age.s[80]	0.9999463	0.9999563	85.3885867	86.4798000	87.5831789	48564.2	0.0405074	0.95	69.1496000	99.9993000
age.s[81]	1.0001653	1.0007042	64.8180321	65.1407000	66.3357278	46224.9	0.0600665	0.95	39.5169000	89.4620000
age.s[82]	1.0001210	1.0005491	88.7491845	89.8619000	92.8873367	50244.7	0.0331829	0.95	74.8763000	99.9998000
age.s[83]	1.0000469	1.0003140	77.2366378	77.8321000	78.1515253	49268.9	0.0493246	0.95	56.8413000	98.1209000
age.s[84]	1.0000425	1.0003045	74.4054470	74.7980000	75.7037451	49230.9	0.0482389	0.95	53.9579000	95.2521000
age.s[85]	0.9999972	1.0001327	74.2026439	74.6286000	73.7844620	48479.2	0.0520131	0.95	51.9399000	95.9555000
age.s[86]	0.9999620	0.9999745	74.2851987	74.7856000	76.9980852	48233.0	0.0523753	0.95	52.1376000	96.2164000
age.s[87]	0.9999927	1.0001239	57.4560699	57.2892000	56.8219576	47254.9	0.0578623	0.95	32.7965000	81.8339000
age.s[88]	0.9999786	1.0000785	76.5665335	77.1469000	78.1776738	46629.4	0.0498847	0.95	56.2271000	97.3516000
age.s[89]	1.0000644	1.0002262	78.6426228	79.4215000	81.7733441	50001.0	0.0472922	0.95	58.7326000	98.4054000
age.s[90]	1.0000154	1.0001479	77.2656955	77.9036000	78.0722989	49318.7	0.0493816	0.95	56.6859000	98.2618000
age.s[91]	1.0000415	1.0002871	76.4528380	76.9454000	76.1356337	47957.3	0.0491022	0.95	55.8229000	96.9752000
age.s[92]	0.9999629	1.0000034	81.1480220	81.9682000	83.8000024	49038.8	0.0450996	0.95	63.3078000	99.9965000
age.s[93]	1.0000608	1.0003461	67.6969042	68.0348000	70.0533959	47748.1	0.0514117	0.95	45.3354000	88.9354000
age.s[94]	0.9999888	1.0000146	78.6407930	79.4129000	81.4309660	47590.2	0.0482670	0.95	58.7739000	98.3890000
age.s[95]	0.9999877	1.0000624	25.4658042	24.3267000	21.3826008	47603.1	0.0315855	0.95	15.0019000	38.4315000
age.s[96]	1.0001545	1.0005281	35.3555891	34.1486000	32.6752906	45123.1	0.0512925	0.95	15.0125000	55.2666000
age.s[97]	0.9999561	0.9999907	24.8361863	23.0874000	17.8988099	48412.9	0.0351083	0.95	15.0007000	39.9144000
age.s[98]	1.0002985	1.0010099	43.5546173	43.0342000	40.1243219	47270.4	0.0483435	0.95	23.5981000	63.9424000
age.s[99]	1.0001726	1.0004878	33.7936577	32.8471000	30.9944623	45236.9	0.0443697	0.95	16.4860000	51.9341000
age.s[100]	1.0001768	1.0003444	24.6235501	23.3284000	20.9643542	44876.9	0.0321734	0.95	15.0000000	37.7745000
age.s[101]	0.9999898	1.0000285	30.8009243	29.8419000	27.6894894	43049.1	0.0411385	0.95	15.2671000	46.5798000
age.s[102]	1.0002151	1.0007047	53.5311860	53.1501000	54.0182842	48927.1	0.0565756	0.95	29.5854000	77.9367000
age.s[103]	1.0000913	1.0001353	65.7801389	65.9329000	66.8860826	47298.0	0.0504465	0.95	44.4587000	87.0982000
age.s[104]	1.0001351	1.0004874	60.4764878	60.4367000	60.8544354	46675.8	0.0585484	0.95	36.3957000	85.1737000
age.s[105]	1.0000045	1.0000822	31.9458871	30.7201000	28.1387108	48212.9	0.0424402	0.95	15.2721000	49.2625000
age.s[106]	1.0000353	1.0001833	43.6674466	43.1576000	41.2167587	46926.8	0.0485653	0.95	23.7079000	64.0992000
age.s[107]	0.9999954	1.0000777	57.4578090	57.3213000	56.7753618	48371.3	0.0572299	0.95	32.8396000	81.7288000
age.s[108]	0.9999729	1.0000366	49.4375215	49.1057000	48.4577818	47668.5	0.0519821	0.95	27.9515000	71.7609000
age.s[109]	0.9999943	1.0001124	37.6873881	36.9100000	34.8317800	43281.4	0.0465983	0.95	19.7172000	56.6786000
age.s[110]	0.9999944	1.0000146	41.2938236	40.5748000	38.3032084	47731.1	0.0496294	0.95	21.5114000	62.9885000
age.s[111]	1.0000354	1.0002806	55.7203694	55.5026000	54.1240023	42668.7	0.0543827	0.95	33.7681000	77.4383000

age.s[112]	0.9999641	1.0000278	30.0136635	28.5436000	25.2245545	48824.3	0.0433371	0.95	15.0003000	47.8424000
age.s[113]	1.0000040	1.0001605	33.9224487	32.9009000	30.6876387	47125.7	0.0423735	0.95	17.2768000	51.8850000
age.s[114]	1.0000783	1.0002250	43.0683524	42.4953000	42.5048140	48764.1	0.0484137	0.95	23.0482000	64.0374000
age.s[115]	0.9999926	1.0000420	53.9973920	53.8350000	54.6882257	46928.7	0.0540526	0.95	31.4438000	76.9469000
age.s[116]	0.9999938	1.0000966	50.2412169	49.9573000	48.7342832	46089.3	0.0541930	0.95	27.8278000	72.5215000
age.s[117]	0.9999670	0.9999713	43.0558609	42.4588000	39.6029095	47807.8	0.0489989	0.95	23.1619000	64.3616000
age.s[118]	1.0000647	1.0003453	62.0159575	62.0953000	62.3208703	48099.1	0.0534127	0.95	39.1665000	84.5608000
age.s[119]	1.0000443	1.0001869	43.6278896	43.0734000	41.9172721	46452.0	0.0487381	0.95	24.2084000	64.5415000
age.s[120]	1.0001313	1.0005241	37.6988500	36.8874000	35.0198951	44742.1	0.0459373	0.95	19.9954000	57.0141000
age.s[121]	1.0000275	1.0001235	45.2993737	44.6280000	43.3381972	47116.7	0.0548318	0.95	22.2471000	67.9891000
age.s[122]	1.0000373	1.0001493	68.9569778	69.1150000	69.4320340	45939.7	0.0518002	0.95	47.5241000	90.6691000
age.s[123]	1.0000054	1.0001547	58.0576835	57.9805000	56.8298183	46366.0	0.0544799	0.95	34.9267000	80.4839000
age.s[124]	1.0000555	1.0002903	66.8082229	67.0851000	69.5817233	50001.0	0.0566734	0.95	42.7020000	91.6167000
age.s[125]	0.9999720	1.0000426	45.1976577	44.6855000	44.6621803	46098.0	0.0524524	0.95	23.8446000	66.9885000
age.s[126]	1.0000730	1.0003719	55.1154293	54.9913000	55.5388556	48562.0	0.0529242	0.95	32.3538000	77.5479000
age.s[127]	0.9999831	1.0000374	46.5926375	45.7021000	44.0282209	48867.6	0.0541178	0.95	24.4280000	70.2398000
age.s[128]	1.0001468	1.0006681	62.2864867	62.2616000	62.3291403	48460.4	0.0591517	0.95	37.2224000	87.4618000
age.s[129]	1.0001645	1.0005765	57.5304313	57.2909000	56.4705858	47494.4	0.0602278	0.95	32.1769000	82.8249000
age.s[130]	1.0000641	1.0003944	83.8733524	84.7568000	84.9025153	50001.0	0.0414218	0.95	67.2956000	99.9959000
age.s[131]	1.0001855	1.0005259	57.4834274	57.3751000	57.8573263	48326.1	0.0572916	0.95	32.9552000	81.8585000
age.s[132]	1.0001078	1.0005354	66.9629838	67.1104000	67.3758605	46928.2	0.0567955	0.95	42.8400000	90.6527000
age.s[133]	1.0000480	1.0000680	74.4165691	74.7615000	75.7546557	47821.9	0.0487354	0.95	53.2375000	94.5392000
age.s[134]	1.0000253	1.0000974	59.2906370	59.1865000	58.3616721	43463.6	0.0572693	0.95	35.9945000	82.5996000
age.s[135]	0.9999803	1.0000270	54.0235140	53.8648000	53.6155091	46706.6	0.0542977	0.95	31.4077000	77.0815000
age.s[136]	0.9999968	1.0001023	57.4601089	57.3336000	56.5429692	47154.9	0.0580808	0.95	32.7065000	81.8371000
age.s[137]	1.0001364	1.0004721	47.4098220	47.0873000	47.7557269	45371.0	0.0518631	0.95	26.1294000	68.6665000
age.s[138]	0.9999789	1.0000402	37.9640495	37.1400000	35.2780264	45186.4	0.0470024	0.95	19.4228000	57.4724000
age.s[139]	0.9999560	0.9999591	59.2401243	59.1831000	60.5586075	48580.6	0.0524659	0.95	36.6257000	81.6579000
age.s[140]	1.0000331	1.0002669	73.2535647	73.5374000	74.0207480	49165.3	0.0469177	0.95	53.1912000	93.5970000
age.s[141]	1.0002099	1.0007611	78.5539224	79.2352000	79.4418326	48882.6	0.0477076	0.95	59.2261000	99.0482000
age.s[142]	1.0000656	1.0002276	80.5022702	81.1136000	81.6784591	48616.4	0.0433888	0.95	62.5691000	98.6137000
age.s[143]	0.9999630	0.9999795	74.4354272	74.7565000	75.4607076	49182.8	0.0480597	0.95	54.2606000	95.6512000
age.s[144]	1.0000837	1.0002595	52.9467476	52.7346000	53.0676111	46425.4	0.0588284	0.95	28.3174000	77.3624000
age.s[145]	1.0000740	1.0001686	53.6032071	53.0371000	51.2805579	47598.7	0.0572978	0.95	29.8213000	77.8969000
age.s[146]	1.0000061	1.0000194	65.3923143	65.4634000	67.9210857	48509.8	0.0524713	0.95	42.6954000	87.6736000
age.s[147]	1.0000551	1.0001175	62.0189314	62.0140000	62.6580352	47247.9	0.0536238	0.95	39.1361000	84.5549000
age.s[148]	1.0000092	1.0000388	59.7684764	59.6128000	60.1992715	43996.3	0.0522224	0.95	38.6433000	81.3430000
age.s[149]	0.9999745	1.0000282	80.9121032	81.7766000	82.2773828	47926.1	0.0465221	0.95	62.6423000	99.8695000
age.s[150]	0.9999807	1.0000702	79.2165605	79.7493000	80.6290055	50001.0	0.0436861	0.95	60.9814000	98.3078000

age.s[151]	1.0000348	1.0001214	81.0440073	81.8522000	82.2959086	50001.0	0.0448293	0.95	63.2604000	99.9933000
age.s[152]	0.9999757	0.9999825	77.3100517	78.0347000	79.8123955	48590.4	0.0500310	0.95	57.2548000	98.9172000
age.s[153]	1.0000325	1.0002545	62.3841037	62.4095000	63.0940176	48625.2	0.0590779	0.95	37.6880000	88.0154000
age.s[154]	1.0001963	1.0003579	71.6759273	71.9034000	71.7735571	47376.7	0.0497206	0.95	50.4692000	92.4867000
age.s[155]	1.0000981	1.0003430	65.1043582	65.2561000	66.0342460	48879.0	0.0501586	0.95	43.4713000	86.6178000
age.s[156]	1.0000345	1.0001767	68.2512184	68.4086000	68.5728444	46833.7	0.0537178	0.95	45.1521000	90.2120000
age.s[157]	1.0000515	1.0002283	62.2196711	62.2606000	63.7178091	47711.0	0.0594371	0.95	37.2373000	87.3201000
age.s[158]	1.0000942	1.0004882	79.2187330	79.6893000	80.5307501	48915.4	0.0440653	0.95	60.9469000	98.0966000
age.s[159]	1.0000957	1.0003857	74.1353021	74.6081000	76.6922303	48528.3	0.0489933	0.95	52.9063000	94.8703000
age.s[160]	0.9999601	0.9999975	83.8871782	84.8182000	87.0365296	49370.0	0.0417699	0.95	66.9958000	99.9764000
age.s[161]	0.9999996	1.0000954	74.3068241	74.8200000	76.7692236	48710.3	0.0518527	0.95	52.6812000	96.5124000
age.s[162]	1.0000060	1.0000455	85.2887609	86.0752000	86.0846630	50001.0	0.0379197	0.95	70.0139000	99.9987000
age.s[163]	1.0000109	1.0000194	68.3691044	68.4229000	67.4255270	47077.6	0.0487477	0.95	48.0102000	89.1958000
age.s[164]	1.0001048	1.0004448	66.6629429	66.8895000	67.8525911	48097.6	0.0579688	0.95	41.6224000	90.7416000
age.s[165]	0.9999995	1.0000611	69.5174379	69.7081000	69.5815762	47885.6	0.0522963	0.95	46.9832000	91.4920000
age.s[166]	0.9999866	1.0000697	65.8476121	66.0224000	66.0295935	48231.9	0.0504605	0.95	43.9588000	86.8613000
age.s[167]	1.0000107	1.0001822	76.6007881	77.1213000	78.9425749	46624.2	0.0488329	0.95	56.6581000	97.1392000
age.s[168]	1.0000407	1.0002452	55.2561331	55.0653000	55.2122829	47592.3	0.0529840	0.95	32.8468000	77.6824000
age.s[169]	1.0001255	1.0005562	73.8783160	74.1405000	73.7616492	47799.1	0.0473499	0.95	53.4037000	93.6771000
age.s[170]	1.0001413	1.0005433	73.9521596	74.2850000	75.0643498	48907.4	0.0466596	0.95	53.8130000	93.9799000
age.s[171]	0.9999815	1.0000650	65.0947687	65.1395000	64.9659805	47526.6	0.0506832	0.95	43.8828000	86.8253000
age.s[172]	0.9999526	0.9999747	65.3665718	65.5039000	66.2182359	46455.4	0.0534836	0.95	43.2094000	87.8679000
age.s[173]	0.9999947	1.0001302	45.5837821	45.0678000	44.7252637	47811.6	0.0513722	0.95	24.1607000	67.2750000
age.s[174]	1.0000157	1.0001904	54.0929123	53.8958000	53.8480336	47200.9	0.0538468	0.95	31.4420000	76.8571000
age.s[175]	0.9999836	1.0000417	78.7044964	79.1823000	79.3610835	48275.1	0.0446432	0.95	60.0803000	97.6231000
age.s[176]	1.0000248	1.0001784	74.6752573	75.2297000	76.3629616	47578.9	0.0514226	0.95	52.9988000	96.4196000
age.s[177]	1.0001512	1.0006462	53.4645633	52.9636000	51.1809327	48846.3	0.0561423	0.95	29.5886000	77.3786000
age.s[178]	1.0000459	1.0001570	52.4682167	52.1149000	50.9203719	45931.2	0.0576434	0.95	28.8611000	76.5667000
age.s[179]	1.0000708	1.0002963	77.2985810	77.9767000	79.3544238	48152.7	0.0498067	0.95	56.4153000	97.8555000
age.s[180]	1.0000368	1.0001824	82.5062766	83.2471000	84.5290351	50001.0	0.0411686	0.95	66.0596000	99.8663000

3.1.4 30% missing

Table 28: MCMC diagnostic data Spitalfields dataset (30 % missing).

	PSRF Point est.	PSRF Upper C.I.	Mean	Median	Mode	ESS	MCSE	HDImass	HDIlow	HDIhigh
b	1.0014026	1.0036896	0.0409711	0.0397777	0.0380840	21696.4	0.0000753	0.95	0.0203189	0.0613243
a	1.0004217	1.0016287	0.0080216	0.0068681	0.0044615	24501.3	0.0000332	0.95	0.0003950	0.0188170
M	1.0004535	1.0017428	56.0848990	59.1560000	64.1103951	23394.1	0.0949933	0.95	25.1962000	78.6534000
beta0[1]	1.0003265	1.0010106	-5.3287413	-4.6589900	-3.7798245	42005.1	0.0182381	0.95	-12.4614000	-0.0004151
beta0[2]	1.0002949	1.0010723	-14.0373833	-13.3163000	-11.8394618	21044.4	0.0354253	0.95	-24.3485000	-5.1383200
beta0[3]	1.0001259	1.0002231	-2.3647458	-2.2297700	-1.9695438	50001.0	0.0059448	0.95	-4.7026100	-0.0009058
beta0[4]	1.0003534	1.0010517	-12.3611690	-11.5531000	-9.8747874	40658.8	0.0218015	0.95	-21.1615000	-5.1338700
beta0[5]	1.0002653	1.0008135	-2.1420003	-1.9892800	-1.7801035	48575.6	0.0056943	0.95	-4.3935600	-0.0009190
beta[1]	1.0009436	1.0028765	3.4579641	3.2529100	2.9163280	9969.8	0.0111827	0.95	1.6709700	5.7489700
beta[2]	1.0001871	1.0006617	4.9782322	4.7482700	4.2965284	19766.6	0.0114205	0.95	2.2335900	8.2154100
beta[3]	1.0000317	1.0001813	1.1652448	1.1340900	1.0965558	50001.0	0.0015414	0.95	0.5497280	1.8125900
beta[4]	1.0002837	1.0008654	3.2267237	3.0489300	2.7309846	41489.1	0.0050608	0.95	1.5145800	5.3034000
beta[5]	1.0001490	1.0005245	0.8796515	0.8449590	0.8072592	47980.3	0.0014292	0.95	0.3353860	1.4653800
thresh[1,1]	NaN	NaN	0.5000000	0.5000000	0.4996965	0.0	NaN	0.95	0.5000000	0.5000000
thresh[2,1]	NaN	NaN	0.5000000	0.5000000	0.4996965	0.0	NaN	0.95	0.5000000	0.5000000
thresh[3,1]	NaN	NaN	0.5000000	0.5000000	0.4996965	0.0	NaN	0.95	0.5000000	0.5000000
thresh[4,1]	NaN	NaN	0.5000000	0.5000000	0.4996965	0.0	NaN	0.95	0.5000000	0.5000000
thresh[5,1]	NaN	NaN	0.5000000	0.5000000	0.4996965	0.0	NaN	0.95	0.5000000	0.5000000
thresh[1,2]	1.0024864	1.0063026	6.7218000	6.2508900	4.9748166	4758.6	0.0392544	0.95	2.4295200	12.1379000
thresh[2,2]	1.0001346	1.0004862	2.5820926	2.4293500	2.1351242	25174.3	0.0052897	0.95	1.2306800	4.2782700
thresh[3,2]	1.0002951	1.0010774	1.5233637	1.5126600	1.4979039	50001.0	0.0008170	0.95	1.1739800	1.8801100
thresh[4,2]	1.0003650	1.0012884	2.1758806	2.1447900	2.0835353	40866.0	0.0013999	0.95	1.6638300	2.7383800
thresh[5,2]	1.0001277	1.0003198	2.5672270	2.5615400	2.5445060	45682.2	0.0008724	0.95	2.2073600	2.9349000
thresh[1,3]	1.0024979	1.0063568	7.8538236	7.3627800	6.0305675	4710.8	0.0411594	0.95	3.3315800	13.4983000
thresh[2,3]	1.0002671	1.0005132	6.7560076	6.4460100	5.8584413	19106.3	0.0131190	0.95	3.8112800	10.4395000
thresh[1,4]	1.0023883	1.0060644	9.9002304	9.3969200	8.1835961	4803.1	0.0428908	0.95	4.9662800	15.7661000
thresh[2,4]	1.0001300	1.0002031	9.5111905	9.1201800	8.5777391	19324.3	0.0162181	0.95	5.8083900	14.0800000
thresh_age[1,1]	1.0003597	1.0009846	6.1736671	5.2373400	2.0432097	22360.2	0.0269942	0.95	1.1319200	13.9702000
thresh_age[2,1]	1.0013420	1.0032031	18.4486282	17.7498000	16.7815413	27211.4	0.0243241	0.95	11.6730000	27.0775000
thresh_age[3,1]	1.0004306	1.0008082	11.5257769	11.0161000	11.1464555	42151.3	0.0250173	0.95	2.5288700	20.9088000
thresh_age[4,1]	1.0005857	1.0021993	53.0947416	52.9993000	52.6706731	24059.2	0.0511273	0.95	37.8273000	67.9607000
thresh_age[5,1]	1.0004757	1.0015217	19.3105600	18.8222000	18.5459321	44210.8	0.0363335	0.95	4.7680500	33.4912000
thresh_age[1,2]	1.0011439	1.0033408	31.8992972	31.0285000	29.2500138	15958.8	0.0582481	0.95	19.1354000	46.8504000
thresh_age[2,2]	1.0010475	1.0028965	28.2314618	27.1707000	24.6788154	23208.0	0.0409624	0.95	18.1173000	41.1055000
thresh_age[3,2]	1.0002446	1.0005616	27.5495864	27.0166000	26.0847929	34410.1	0.0372865	0.95	14.4020000	41.3776000

thresh_age[4,2]	1.0003387	1.0009180	92.2114940	91.2668000	90.1343378	38682.8	0.0587220	0.95	69.7886000	114.3740000
thresh_age[5,2]	1.0005334	1.0005861	319.1852299	216.7710000	152.9253935	47798.3	1.6756501	0.95	85.7493000	849.8390000
thresh_age[1,3]	1.0007731	1.0027546	44.3877706	43.9575000	43.1850340	16885.9	0.0611546	0.95	29.2032000	59.2843000
thresh_age[2,3]	1.0003847	1.0015356	65.4625809	65.8278000	66.9540264	23573.4	0.0514069	0.95	49.4572000	79.6090000
thresh_age[1,4]	1.0001422	1.0006693	82.3580999	82.4545000	82.8551095	48568.2	0.0402019	0.95	64.4513000	99.2618000
thresh_age[2,4]	1.0003255	1.0004417	118.5263561	114.5700000	107.7821595	32702.1	0.1135288	0.95	86.7902000	159.2750000
age.s[1]	1.0002866	1.0006174	20.1029455	18.6459000	15.8501252	40875.4	0.0246469	0.95	15.0002000	29.9692000
age.s[2]	1.0013963	1.0029880	18.9261588	17.7563000	15.6634758	38852.5	0.0197531	0.95	15.0000000	26.6342000
age.s[3]	1.0010544	1.0030293	23.8205187	22.1941000	19.2994807	35849.5	0.0366243	0.95	15.0006000	37.3593000
age.s[4]	1.0008670	1.0029334	41.8346195	40.6790000	37.8910920	33111.7	0.0715982	0.95	17.8731000	66.6234000
age.s[5]	1.0004338	1.0007892	20.0839165	18.6355000	15.8405502	38652.4	0.0252918	0.95	15.0001000	29.8710000
age.s[6]	1.0004949	1.0010100	25.0258767	23.5095000	21.0249521	32129.2	0.0404906	0.95	15.0007000	39.0778000
age.s[7]	1.0001008	1.0004650	45.0823505	44.0758000	40.8729944	35025.7	0.0729762	0.95	20.5425000	71.9593000
age.s[8]	1.0007675	1.0018303	32.8267636	31.0376000	25.7365378	46173.6	0.0540408	0.95	15.0023000	54.6458000
age.s[9]	1.0005869	1.0020729	43.2490203	42.1994000	36.5702478	33678.5	0.0663235	0.95	21.5611000	67.2363000
age.s[10]	1.0005871	1.0023131	53.8879957	53.5001000	52.1422509	32336.2	0.0787218	0.95	27.4775000	81.6444000
age.s[11]	1.0002785	1.0009183	41.0653098	39.1056000	34.8753885	39394.6	0.0818114	0.95	15.0010000	70.8195000
age.s[12]	1.0002835	1.0007051	32.8953386	31.0976000	28.1822781	44729.1	0.0552454	0.95	15.0087000	54.7678000
age.s[13]	1.0001148	1.0005565	40.1759246	39.1347000	36.7314094	30183.6	0.0723516	0.95	17.0923000	63.9917000
age.s[14]	1.0011598	1.0033159	24.7161567	23.0567000	19.0447741	32478.6	0.0414121	0.95	15.0000000	39.4014000
age.s[15]	1.0003132	1.0011902	33.4388742	32.0451000	29.6642013	38354.1	0.0515909	0.95	15.6716000	52.6235000
age.s[16]	1.0001573	1.0003123	33.2330619	29.9955000	18.3855910	42094.1	0.0697460	0.95	15.0003000	61.6914000
age.s[17]	1.0001552	1.0005222	74.7182899	75.6859000	78.1461718	35710.2	0.0660784	0.95	51.2407000	98.0296000
age.s[18]	1.0002642	1.0007138	32.8567855	30.9664000	27.4497753	47034.1	0.0536703	0.95	15.0009000	54.8933000
age.s[19]	0.9999887	1.0000832	45.2090256	44.1635000	41.2569151	39910.3	0.0685540	0.95	20.0752000	71.4820000
age.s[20]	1.0000204	1.0002221	52.9380800	52.4537000	51.0946845	36771.0	0.0794801	0.95	23.4483000	81.0269000
age.s[21]	1.0000457	1.0003275	35.0114569	32.7678000	27.1879639	48742.7	0.0604087	0.95	15.0006000	60.3181000
age.s[22]	1.0001893	1.0007415	65.2640641	65.8129000	66.7019226	38900.2	0.0760067	0.95	36.5863000	93.6167000
age.s[23]	1.0001181	1.0005924	54.9584546	54.8214000	56.1756826	33444.1	0.0746664	0.95	28.7092000	81.0497000
age.s[24]	1.0001414	1.0006474	58.9034553	58.8288000	60.5749768	33854.4	0.0678234	0.95	34.9335000	83.0076000
age.s[25]	1.0002286	1.0009867	64.4269727	64.5320000	64.0566598	32435.8	0.0669689	0.95	41.2811000	88.0623000
age.s[26]	1.0004755	1.0017913	42.3737568	41.1408000	37.5410797	31763.3	0.0737316	0.95	19.2609000	68.3273000
age.s[27]	1.0001313	1.0005977	54.9496640	54.6798000	55.6990217	30870.3	0.0768452	0.95	29.3579000	80.7886000
age.s[28]	1.0000189	1.0002301	53.0285011	52.5318000	52.0528686	36398.1	0.0799978	0.95	23.7625000	81.3805000
age.s[29]	1.0003248	1.0013162	45.1712846	44.1241000	41.8240259	36308.4	0.0716960	0.95	19.5301000	70.7969000
age.s[30]	1.0005805	1.0021957	61.6647287	61.7338000	60.3453739	35896.0	0.0726108	0.95	35.1277000	88.0217000
age.s[31]	0.9999953	1.0000627	70.7681319	71.1448000	71.2116950	41355.6	0.0574969	0.95	48.0071000	93.1133000
age.s[32]	1.0002751	1.0011509	55.8933878	55.7028000	53.5773186	33485.0	0.0688501	0.95	32.4422000	80.9593000
age.s[33]	1.0002023	1.0007887	78.9598943	79.8196000	81.1027919	44217.0	0.0528455	0.95	58.9608000	99.8774000

age.s[34]	1.0002258	1.0009910	66.3187365	66.5186000	66.4989776	27336.7	0.0766266	0.95	41.8767000	90.3539000
age.s[35]	1.0006391	1.0016291	25.4653075	23.9970000	21.0982550	34423.8	0.0395212	0.95	15.0008000	39.6764000
age.s[36]	1.0000943	1.0004937	58.9318257	58.8725000	58.5546663	35179.2	0.0666366	0.95	34.9908000	83.0650000
age.s[37]	0.9999887	1.0000990	50.3734256	49.8017000	47.3346150	28281.8	0.0844061	0.95	23.9409000	78.1658000
age.s[38]	1.0000838	1.0002904	87.5361494	88.8687000	92.8532659	44772.8	0.0397121	0.95	71.7051000	99.9961000
age.s[39]	1.0002252	1.0008463	30.3130213	28.0870000	20.8973360	47109.4	0.0515709	0.95	15.0013000	51.8792000
age.s[40]	1.0003239	1.0013325	48.0932914	47.3242000	44.9346519	37810.3	0.0697727	0.95	22.7871000	74.5149000
age.s[41]	1.0000847	1.0003722	61.6108345	61.6334000	59.5175244	34798.1	0.0652420	0.95	38.3425000	85.4222000
age.s[42]	1.0002523	1.0010357	64.6362674	64.8598000	65.7111523	38144.0	0.0691802	0.95	38.8035000	90.8473000
age.s[43]	1.0001273	1.0005427	53.9217459	53.5711000	53.9728196	32833.6	0.0782770	0.95	27.5658000	81.9466000
age.s[44]	1.0000543	1.0003000	52.9914187	52.4846000	50.2491205	36809.0	0.0796682	0.95	24.4953000	82.2173000
age.s[45]	1.0001102	1.0005458	63.8760128	63.9179000	62.8520816	37292.2	0.0629596	0.95	39.9409000	87.2642000
age.s[46]	1.0003580	1.0013095	46.8668891	45.9402000	44.3507911	36635.3	0.0711595	0.95	21.5293000	72.7387000
age.s[47]	1.0002407	1.0008946	46.8429390	45.9187000	43.8705666	38972.4	0.0689368	0.95	21.9367000	73.3408000
age.s[48]	1.0002935	1.0010836	35.2706422	33.0586000	27.0341775	49185.2	0.0605664	0.95	15.0055000	60.8264000
age.s[49]	1.0000223	1.0002422	70.3223944	70.5918000	70.5828871	42699.8	0.0564300	0.95	47.4396000	92.5566000
age.s[50]	1.0001515	1.0004969	72.0442628	72.4770000	73.9986868	43346.1	0.0580366	0.95	49.0125000	95.2717000
age.s[51]	1.0001449	1.0005386	54.3382635	53.9674000	52.7147647	33328.7	0.0745624	0.95	28.5945000	80.6954000
age.s[52]	1.0001882	1.0007705	65.9776075	66.3692000	67.8168127	41022.3	0.0677216	0.95	40.0144000	92.5221000
age.s[53]	1.0002968	1.0012158	55.6786828	55.4581000	53.9726425	31353.8	0.0764816	0.95	30.0733000	81.8547000
age.s[54]	1.0001672	1.0005781	40.0881365	39.0390000	36.3473924	30625.1	0.0714613	0.95	17.0450000	63.9204000
age.s[55]	0.9999951	1.0001362	42.4468746	41.1827000	35.7277212	35884.9	0.0697523	0.95	19.6498000	68.8497000
age.s[56]	1.0000113	1.0001894	69.2483659	69.4902000	68.2132353	36965.9	0.0647514	0.95	45.2248000	93.1795000
age.s[57]	0.9999954	1.0000972	72.6901570	73.3765000	74.4233468	46973.8	0.0586852	0.95	48.9176000	97.2505000
age.s[58]	1.0001377	1.0004349	33.8645922	32.5706000	29.1834185	37023.0	0.0524589	0.95	16.1918000	53.5256000
age.s[59]	1.0001441	1.0005644	72.5799500	73.1362000	74.2421698	42345.4	0.0584862	0.95	49.0799000	95.1552000
age.s[60]	0.9999510	0.9999775	47.7558505	46.8632000	44.3052707	43913.2	0.0799665	0.95	15.7194000	77.4896000
age.s[61]	1.0000200	1.0002296	64.7751350	65.0359000	64.6089821	43720.1	0.0649559	0.95	38.1123000	90.4577000
age.s[62]	1.0003291	1.0012552	55.9668549	55.6912000	53.9616143	31711.6	0.0760331	0.95	30.4621000	82.1788000
age.s[63]	1.0006000	1.0020461	54.3269454	53.9315000	52.8424752	38945.2	0.0705176	0.95	27.8428000	81.2174000
age.s[64]	1.0002168	1.0009397	50.7554051	50.3339000	50.2835939	29399.8	0.0730110	0.95	26.7133000	74.3922000
age.s[65]	1.0000974	1.0002568	72.6071921	73.0749000	75.5387750	41923.2	0.0589165	0.95	48.8602000	95.1478000
age.s[66]	1.0001241	1.0005395	64.9748666	65.2045000	66.7051405	39328.7	0.0656438	0.95	40.0778000	90.2503000
age.s[67]	1.0000107	1.0001624	53.0846379	52.8469000	53.6921420	28954.5	0.0802283	0.95	26.9882000	78.8922000
age.s[68]	1.0001326	1.0003709	71.8845994	73.1403000	76.9262141	29366.8	0.0835683	0.95	44.5585000	98.0469000
age.s[69]	1.0001183	1.0005183	58.2353969	58.1646000	60.9244375	31664.3	0.0761643	0.95	32.1864000	84.0881000
age.s[70]	1.0000909	1.0004910	52.9851924	52.5696000	50.5824011	31324.9	0.0765349	0.95	26.9382000	78.5194000
age.s[71]	1.0000140	1.0001724	53.3248533	53.0155000	53.5051705	44245.2	0.0582738	0.95	30.0768000	77.1017000
age.s[72]	1.0001724	1.0007863	63.4507977	63.6851000	64.6488394	39689.1	0.0685618	0.95	37.7752000	90.3663000

age.s[73]	1.0000425	1.0003050	62.6544864	62.6664000	62.7427871	44612.4	0.0600792	0.95	37.5411000	86.6137000
age.s[74]	1.0005669	1.0015228	29.8267511	27.7977000	23.6917847	31700.9	0.0563820	0.95	15.0038000	49.0190000
age.s[75]	1.0000072	1.0001768	57.8619071	57.6936000	58.1082159	47909.0	0.0591415	0.95	33.0237000	82.7961000
age.s[76]	1.0000031	1.0001494	73.2607526	74.1175000	76.6899493	48723.4	0.0580573	0.95	48.6882000	97.3942000
age.s[77]	1.0003044	1.0010820	67.4738223	67.8811000	68.5209279	45098.1	0.0625514	0.95	41.3770000	92.5972000
age.s[78]	1.0003090	1.0012017	55.3919990	55.2402000	56.0243936	34831.3	0.0733440	0.95	29.0079000	81.3452000
age.s[79]	1.0004545	1.0016187	52.0657942	51.5584000	50.5323023	30699.4	0.0773371	0.95	27.0364000	78.3308000
age.s[80]	1.0001577	1.0006877	79.2371522	80.3766000	83.4512156	41147.0	0.0571271	0.95	58.2694000	99.9985000
age.s[81]	1.0006641	1.0024913	61.6263119	61.7943000	61.3448508	24262.3	0.0938321	0.95	33.3960000	89.1272000
age.s[82]	1.0000795	1.0003871	89.3881369	90.6755000	94.6500168	46793.3	0.0343267	0.95	75.3270000	99.9992000
age.s[83]	1.0000931	1.0003170	77.8044697	78.4745000	80.0777206	48824.4	0.0494118	0.95	58.0040000	99.1385000
age.s[84]	1.0002140	1.0007483	77.3148651	78.0212000	79.9485517	37293.6	0.0568561	0.95	56.9095000	98.4046000
age.s[85]	1.0000071	1.0001741	72.5098967	72.9354000	72.9449157	41818.6	0.0588622	0.95	49.1946000	95.1644000
age.s[86]	1.0000083	1.0000271	75.7168130	76.9666000	81.3744603	46786.0	0.0611803	0.95	51.7731000	99.9229000
age.s[87]	1.0006959	1.0023924	50.0028484	49.5376000	48.1282774	28727.4	0.0743791	0.95	26.0782000	74.2389000
age.s[88]	0.9999836	1.0000582	74.5578809	75.4461000	77.1656639	38369.2	0.0639281	0.95	51.2708000	98.4168000
age.s[89]	0.9999787	0.9999825	72.6622223	73.3794000	74.7764396	41471.9	0.0627380	0.95	47.8223000	96.3682000
age.s[90]	1.0001688	1.0007063	72.9528466	73.4258000	74.3361324	45293.7	0.0563691	0.95	49.8230000	95.6947000
age.s[91]	1.0002369	1.0008435	74.4727374	75.3597000	76.9789782	34840.9	0.0671592	0.95	50.5925000	97.8334000
age.s[92]	0.9999947	1.0000778	75.0925413	76.0076000	79.2528086	50001.0	0.0556521	0.95	52.0141000	98.6458000
age.s[93]	1.0001254	1.0005908	57.9800673	57.8305000	57.9030889	46275.5	0.0600099	0.95	33.3538000	82.8293000
age.s[94]	1.0000707	1.0003397	76.0347009	76.6922000	77.8134206	48484.9	0.0508121	0.95	54.6869000	97.2544000
age.s[95]	1.0003534	1.0014249	27.2360048	25.4424000	22.3071697	33574.6	0.0469301	0.95	15.0007000	43.9716000
age.s[96]	1.0000593	1.0001551	46.5116333	45.6458000	42.6202396	25733.9	0.0839760	0.95	21.5773000	72.2408000
age.s[97]	1.0004790	1.0015026	32.8714958	31.3189000	27.6941642	38352.8	0.0529736	0.95	15.0233000	52.4530000
age.s[98]	1.0002328	1.0009403	42.7129996	41.6617000	38.3500717	31655.8	0.0686990	0.95	21.0104000	66.6714000
age.s[99]	1.0005058	1.0018697	45.2263193	44.3491000	41.6464151	37919.3	0.0697807	0.95	20.5541000	71.3465000
age.s[100]	1.0011139	1.0024783	20.1865581	18.6697000	15.9412001	36259.8	0.0269682	0.95	15.0000000	30.2534000
age.s[101]	1.0005265	1.0013198	25.3369551	23.6475000	20.7815113	33029.1	0.0423032	0.95	15.0001000	40.4058000
age.s[102]	1.0001987	1.0007519	42.1469813	41.1460000	40.2871698	32270.0	0.0644324	0.95	21.1292000	64.7743000
age.s[103]	1.0001411	1.0006786	65.1453075	65.7883000	69.6013961	39706.3	0.0753212	0.95	36.2895000	93.8187000
age.s[104]	1.0003336	1.0013455	62.4844753	62.6700000	63.9136329	25645.9	0.0861314	0.95	35.3159000	88.4073000
age.s[105]	1.0012776	1.0041236	28.9250525	26.7159000	20.2475309	43308.7	0.0497528	0.95	15.0008000	49.0232000
age.s[106]	1.0002523	1.0009056	38.7931625	37.6523000	37.0043801	28922.5	0.0733887	0.95	16.3966000	62.4863000
age.s[107]	1.0000222	1.0002339	53.6689355	53.4227000	54.0561867	34595.2	0.0770110	0.95	26.5066000	81.3667000
age.s[108]	1.0000096	1.0001415	45.9912671	45.1886000	42.9854852	25272.8	0.0887356	0.95	19.6986000	72.9913000
age.s[109]	1.0009693	1.0031216	30.0191646	28.4902000	24.0065602	21098.4	0.0627126	0.95	15.0022000	47.2315000
age.s[110]	1.0003001	1.0012655	31.9905240	30.5757000	27.7787404	35277.4	0.0523099	0.95	15.1213000	50.3313000
age.s[111]	1.0003856	1.0013593	32.8855369	31.0510000	26.4544180	45695.5	0.0544110	0.95	15.0004000	54.7126000

age.s[112]	1.0012845	1.0029180	24.6779280	23.2135000	20.0207694	29440.6	0.0409092	0.95	15.0004000	38.3903000
age.s[113]	1.0004788	1.0010195	28.3670937	26.5530000	23.2107152	35288.4	0.0483612	0.95	15.0001000	45.6951000
age.s[114]	1.0004332	1.0016242	34.6552933	33.1491000	29.4108461	41516.9	0.0533128	0.95	15.1410000	55.0287000
age.s[115]	1.0005119	1.0018133	46.8347788	45.9521000	44.0029867	34913.0	0.0724469	0.95	22.4168000	73.1734000
age.s[116]	1.0002399	1.0007375	47.5772099	46.9744000	44.5468142	26214.6	0.0770651	0.95	24.6245000	72.1190000
age.s[117]	1.0001777	1.0005763	30.7338137	28.2697000	20.2504911	46190.0	0.0548371	0.95	15.0011000	53.5957000
age.s[118]	1.0004737	1.0015601	71.8637584	73.0349000	73.9100445	32577.7	0.0791071	0.95	45.1224000	98.5623000
age.s[119]	1.0001101	1.0005568	43.0055887	41.6615000	38.1577684	33855.1	0.0765217	0.95	16.6717000	69.3358000
age.s[120]	1.0001514	1.0005678	45.5074706	44.6036000	42.6140669	40125.0	0.0670840	0.95	20.6314000	71.7259000
age.s[121]	1.0002912	1.0011506	47.5282943	46.9247000	44.4641095	25930.7	0.0774090	0.95	24.4470000	71.8108000
age.s[122]	1.0000994	1.0004520	67.4160894	67.7817000	68.7003874	39841.2	0.0629974	0.95	43.0322000	91.5365000
age.s[123]	1.0001647	1.0007475	64.7166748	65.0816000	66.7460584	27407.8	0.0862321	0.95	37.0931000	91.6583000
age.s[124]	1.0001195	1.0005358	67.4950636	67.8402000	70.5530093	43515.1	0.0634321	0.95	41.9596000	92.9091000
age.s[125]	1.0004821	1.0015697	51.9326958	51.4574000	48.7685921	29484.0	0.0800513	0.95	25.8171000	78.2525000
age.s[126]	1.0005376	1.0021048	47.5550781	46.9090000	45.1340475	25597.9	0.0783584	0.95	24.3670000	71.8294000
age.s[127]	1.0000590	1.0003489	38.7258105	37.3098000	35.2509478	47599.6	0.0534904	0.95	18.1399000	61.5301000
age.s[128]	1.0002689	1.0009232	58.2081873	58.0368000	57.4212129	35731.2	0.0716336	0.95	31.6974000	83.7494000
age.s[129]	1.0002229	1.0006895	52.9955979	52.6280000	51.4733236	31452.2	0.0764303	0.95	27.5315000	79.1360000
age.s[130]	1.0000791	1.0004084	78.3092639	79.1895000	82.1957683	42008.3	0.0545992	0.95	57.8302000	99.3180000
age.s[131]	1.0001115	1.0005316	62.0735847	62.3232000	59.6387693	37479.3	0.0786378	0.95	32.4581000	90.5560000
age.s[132]	1.0003550	1.0014113	78.2713901	79.1403000	82.1838698	40625.0	0.0556479	0.95	58.1788000	99.6285000
age.s[133]	0.9999849	1.0000924	63.7993124	64.0341000	65.8406997	38538.5	0.0695416	0.95	36.8701000	89.5316000
age.s[134]	1.0001209	1.0005773	61.6165550	61.6640000	61.7123144	35505.0	0.0699113	0.95	35.6409000	86.7194000
age.s[135]	1.0003210	1.0013411	62.1557029	62.4489000	62.4922956	41250.2	0.0748767	0.95	32.3199000	90.5122000
age.s[136]	1.0003837	1.0015134	55.9405129	55.6222000	54.0839225	35854.0	0.0716566	0.95	30.0273000	81.9900000
age.s[137]	1.0002672	1.0009790	42.8247769	41.7982000	38.7554360	35921.1	0.0682083	0.95	18.7640000	67.7645000
age.s[138]	1.0001479	1.0006954	47.1108140	45.8535000	42.8074181	39439.0	0.0752368	0.95	20.4873000	76.2012000
age.s[139]	1.0003874	1.0015388	63.3016315	63.2817000	62.3469020	43308.1	0.0576463	0.95	39.7202000	86.2134000
age.s[140]	1.0002301	1.0009215	63.8826170	64.1707000	64.7102242	36625.2	0.0714947	0.95	37.8482000	90.4227000
age.s[141]	1.0001347	1.0006341	76.5667263	77.2641000	80.8737650	47668.5	0.0510940	0.95	55.1412000	97.6839000
age.s[142]	1.0001098	1.0004091	72.7151702	73.3477000	74.7414502	45310.3	0.0598732	0.95	48.7336000	97.0921000
age.s[143]	1.0000200	1.0001239	72.4775933	72.9240000	75.1068532	42947.0	0.0581392	0.95	49.4208000	95.5882000
age.s[144]	1.0006717	1.0017027	27.2086229	25.4517000	22.1077170	30508.6	0.0486769	0.95	15.0001000	43.7286000
age.s[145]	1.0000344	1.0002682	58.2605196	58.2273000	59.0219802	38300.8	0.0693643	0.95	31.5261000	83.6377000
age.s[146]	1.0003373	1.0013124	55.7623754	55.5419000	53.9044184	32467.7	0.0753666	0.95	30.1479000	82.3780000
age.s[147]	1.0002091	1.0008929	54.3125313	54.1318000	54.7678817	35865.2	0.0733268	0.95	27.9073000	80.9967000
age.s[148]	1.0001840	1.0006661	57.6455086	57.4987000	55.9265364	39101.2	0.0603075	0.95	34.1752000	80.3805000
age.s[149]	1.0001155	1.0004890	78.4184875	79.3009000	80.1770419	38316.8	0.0571117	0.95	58.1388000	99.4797000
age.s[150]	1.0001158	1.0003407	74.0246147	74.8991000	76.3574656	34044.9	0.0685557	0.95	50.4135000	98.3492000

age.s[151]	1.0003176	1.0008548	75.5934875	76.4748000	79.8518498	49108.5	0.0556255	0.95	52.3041000	98.4405000
age.s[152]	1.0002494	1.0010756	70.7236764	70.9879000	71.2566159	42002.0	0.0564063	0.95	47.9194000	92.7908000
age.s[153]	1.0001287	1.0006258	46.2764544	45.3838000	41.4528425	36405.0	0.0714608	0.95	21.4334000	72.7875000
age.s[154]	1.0004532	1.0018200	66.2668123	66.4864000	69.0241993	27621.4	0.0754659	0.95	41.6509000	89.8313000
age.s[155]	1.0001455	1.0006749	58.3840907	58.3445000	58.4432202	33621.3	0.0684226	0.95	33.9887000	82.4252000
age.s[156]	1.0002925	1.0010705	67.2988173	67.5501000	68.5979593	42801.2	0.0614070	0.95	43.3051000	92.3892000
age.s[157]	1.0001807	1.0007381	47.7477255	46.7101000	45.5334756	41552.9	0.0821573	0.95	16.2114000	78.0178000
age.s[158]	1.0001446	1.0006447	77.0483548	77.7208000	79.4650258	35540.3	0.0586844	0.95	56.3404000	98.2050000
age.s[159]	1.0000445	1.0001035	52.8454143	52.2799000	51.2906222	39663.5	0.0765219	0.95	24.7707000	82.1219000
age.s[160]	1.0001309	1.0001388	74.9346201	76.1342000	78.9457673	39059.0	0.0673676	0.95	50.3999000	99.2737000
age.s[161]	0.9999741	1.0000569	70.7750866	71.0459000	70.4323367	45078.4	0.0574696	0.95	46.8185000	93.8325000
age.s[162]	1.0000595	1.0002864	75.8032565	77.0884000	79.8695848	48022.6	0.0601380	0.95	51.9590000	99.9827000
age.s[163]	1.0000345	1.0002322	54.4330939	53.9979000	53.7837728	46589.3	0.0637498	0.95	28.7315000	81.3378000
age.s[164]	1.0000272	1.0002346	66.1603908	66.6674000	71.1726897	48440.8	0.0668767	0.95	37.8373000	94.3296000
age.s[165]	1.0000442	1.0002064	62.1417788	62.5152000	66.2367754	42701.4	0.0736125	0.95	33.3799000	91.5195000
age.s[166]	1.0001341	1.0006479	58.3639354	58.2808000	57.6671640	33823.5	0.0682041	0.95	34.0921000	82.4594000
age.s[167]	1.0001901	1.0008373	63.5628451	63.8278000	64.1803119	24639.0	0.0873582	0.95	36.6397000	89.7991000
age.s[168]	1.0005084	1.0019638	55.7727115	55.4435000	53.2146526	30539.9	0.0777474	0.95	29.7885000	81.9926000
age.s[169]	0.9999839	1.0000550	72.0807208	72.5616000	75.0564664	39172.2	0.0615812	0.95	48.5812000	95.3287000
age.s[170]	1.0000432	1.0003139	64.7014629	64.9497000	65.8738418	41861.8	0.0667387	0.95	38.2687000	90.7985000
age.s[171]	1.0000889	1.0004855	58.1874666	58.0752000	58.5059986	37880.0	0.0696297	0.95	32.3345000	84.3747000
age.s[172]	1.0006027	1.0022941	54.6196406	54.1467000	52.2059261	47651.9	0.0622407	0.95	28.5177000	80.6297000
age.s[173]	1.0002452	1.0008691	46.2111888	45.2206000	41.1003859	36710.5	0.0710905	0.95	20.7852000	71.9786000
age.s[174]	1.0002666	1.0008310	33.7733207	30.4001000	18.5128318	43802.1	0.0703212	0.95	15.0016000	62.8819000
age.s[175]	1.0000394	1.0002012	73.9298236	74.7998000	75.2070935	34768.5	0.0680687	0.95	49.7372000	97.6501000
age.s[176]	1.0001246	1.0006178	65.3091004	65.5304000	64.9054209	37267.8	0.0663519	0.95	40.6356000	89.8092000
age.s[177]	1.0004273	1.0015270	42.5388979	41.6805000	39.3610629	33918.4	0.0623936	0.95	21.8991000	65.1641000
age.s[178]	1.0002231	1.0007140	46.7555242	45.9291000	43.8742156	34593.0	0.0726278	0.95	22.1934000	72.9414000
age.s[179]	1.0000976	1.0005078	72.3436854	72.7996000	73.8780116	47331.0	0.0554719	0.95	49.0089000	95.2852000
age.s[180]	1.0002817	1.0006726	79.0322496	79.9131000	82.0560102	47463.6	0.0507947	0.95	59.3699000	99.9923000

3.1.5 40% missing

Table 29: MCMC diagnostic data Spitalfields dataset (40 % missing).

	PSRF Point est.	PSRF Upper C.I.	Mean	Median	Mode	ESS	MCSE	HDImass	HDIlow	HDIhigh
b	1.0000512	1.0002475	0.0527035	0.0516651	0.0506463	36893.7	0.0000917	0.95	0.0203980	0.0840764
a	1.0004714	1.0010364	0.0052020	0.0031055	0.0008915	41274.0	0.0000268	0.95	0.0001234	0.0175252
M	1.0005013	1.0010694	64.3220587	69.4201000	76.4408414	40848.4	0.0774326	0.95	29.9417000	81.9900000
beta0[1]	1.0003584	1.0011539	-4.8607338	-4.1778700	-3.0595108	41150.3	0.0157853	0.95	-11.1236000	-0.0124135
beta0[2]	1.0004703	1.0016002	-11.1189767	-10.3098000	-8.7744569	20869.2	0.0371047	0.95	-22.1482000	-2.4624400
beta0[3]	1.0000384	1.0001815	-9.3775265	-8.4600100	-6.7157560	41269.1	0.0230405	0.95	-19.0106000	-1.8298100
beta0[4]	1.0001329	1.0005170	-13.9566501	-13.1071000	-10.9084034	39500.0	0.0288744	0.95	-25.2098000	-4.1776300
beta0[5]	1.0000022	1.0001402	-4.8407127	-4.2519900	-3.4796412	46856.5	0.0146584	0.95	-10.8153000	-0.0023698
beta[1]	1.0003584	1.0010224	1.9331114	1.7764900	1.5985254	41672.2	0.0038892	0.95	0.6891360	3.5061900
beta[2]	1.0005295	1.0018370	4.1631203	3.9225700	3.4637702	17595.4	0.0118207	0.95	1.5450000	7.2736600
beta[3]	1.0000758	1.0003139	2.9289094	2.7098800	2.3608041	42386.9	0.0057201	0.95	0.9545750	5.3047500
beta[4]	1.0001098	1.0004596	3.5596107	3.3688900	2.8906651	39778.2	0.0066924	0.95	1.2668900	6.2037400
beta[5]	0.9999845	1.0000721	1.5626470	1.4356800	1.2380971	47527.4	0.0034502	0.95	0.3568210	2.9809700
thresh[1,1]	NaN	NaN	0.5000000	0.5000000	0.4996965	0.0	NaN	0.95	0.5000000	0.5000000
thresh[2,1]	NaN	NaN	0.5000000	0.5000000	0.4996965	0.0	NaN	0.95	0.5000000	0.5000000
thresh[3,1]	NaN	NaN	0.5000000	0.5000000	0.4996965	0.0	NaN	0.95	0.5000000	0.5000000
thresh[4,1]	NaN	NaN	0.5000000	0.5000000	0.4996965	0.0	NaN	0.95	0.5000000	0.5000000
thresh[5,1]	NaN	NaN	0.5000000	0.5000000	0.4996965	0.0	NaN	0.95	0.5000000	0.5000000
thresh[1,2]	1.0004278	1.0006207	1.8348352	1.7766900	1.6783196	45495.7	0.0018945	0.95	1.1549900	2.6270100
thresh[2,2]	1.0007066	1.0021246	3.5700713	3.2956900	2.8132457	19197.6	0.0097034	0.95	1.4713100	6.2680800
thresh[3,2]	1.0001191	1.0005455	1.6944829	1.6308800	1.5072161	44437.8	0.0017547	0.95	1.1050800	2.4163600
thresh[4,2]	1.0002782	1.0007986	1.7793732	1.7355000	1.6586735	42452.7	0.0014265	0.95	1.2947000	2.3683600
thresh[5,2]	1.0003326	1.0008924	2.9653929	2.9450300	2.9036870	50001.0	0.0012656	0.95	2.4469100	3.5431100
thresh[1,3]	1.0003795	1.0004096	2.6712436	2.6069400	2.5269294	44224.4	0.0022073	0.95	1.8761300	3.5825600
thresh[2,3]	1.0005249	1.0018845	6.7810875	6.4247700	5.5102406	16079.5	0.0147731	0.95	3.8582500	10.6169000
thresh[1,4]	1.0002701	1.0002922	4.4096320	4.3354500	4.1994666	44108.6	0.0026454	0.95	3.4402400	5.4805600
thresh[2,4]	1.0005202	1.0019289	9.4111901	9.0200100	8.2703916	16921.1	0.0165549	0.95	5.8979300	13.7571000
thresh_age[1,1]	1.0002836	1.0011343	15.3713355	13.9710000	10.7485727	40773.9	0.0397137	0.95	2.1991700	30.8755000
thresh_age[2,1]	1.0000287	1.0002593	15.9846908	15.1847000	14.2156521	39142.1	0.0294900	0.95	5.5846900	28.2253000
thresh_age[3,1]	0.9999589	0.9999958	28.2727461	27.2452000	21.1786955	37724.0	0.0491595	0.95	12.1772000	46.8730000
thresh_age[4,1]	1.0001018	1.0004160	57.1486036	58.4493000	63.7653646	38409.5	0.0523424	0.95	37.5244000	74.3898000
thresh_age[5,1]	1.0000692	1.0004026	28.0397003	27.5620000	24.7178258	43196.3	0.0566905	0.95	5.5964000	49.4706000
thresh_age[1,2]	1.0003113	1.0012786	30.1948029	29.2299000	25.8573006	39322.8	0.0529963	0.95	11.5403000	49.9928000
thresh_age[2,2]	1.0001701	1.0007832	33.3518601	32.8258000	26.1333001	32371.9	0.0553269	0.95	16.6332000	51.8341000
thresh_age[3,2]	1.0000168	1.0001536	42.8374785	42.8826000	45.0248118	37574.6	0.0538604	0.95	24.6956000	61.6408000

thresh_age[4,2]	1.0001738	1.0003195	84.5024878	84.9227000	86.6307945	44249.1	0.0575823	0.95	59.0376000	105.2380000
thresh_age[5,2]	1.0171671	1.0171964	215.7406755	151.9750000	125.4995266	50001.0	1.3063082	0.95	78.2764000	507.8490000
thresh_age[1,3]	1.0002783	1.0011163	47.3599401	47.6105000	48.7625197	37926.8	0.0552752	0.95	27.4308000	66.4258000
thresh_age[2,3]	1.0000985	1.0002159	73.5421222	75.2067000	78.6872136	42544.3	0.0466090	0.95	52.6919000	88.9013000
thresh_age[1,4]	1.0000483	1.0001426	133.3794739	122.6450000	113.6653842	48442.7	0.1949410	0.95	78.1267000	213.6680000
thresh_age[2,4]	1.0194788	1.0197729	154.2828741	140.7200000	132.0587872	40204.2	0.3520383	0.95	94.2117000	244.6090000
age.s[1]	0.9999649	1.0000308	29.5723751	25.4418000	17.3323966	48587.5	0.0609064	0.95	15.0004000	57.4867000
age.s[2]	1.0000859	1.0003115	29.4610115	26.5313000	17.5319512	44342.9	0.0561776	0.95	15.0010000	52.9477000
age.s[3]	1.0001000	1.0002415	26.8964942	23.1198000	16.8352177	48549.5	0.0537741	0.95	15.0006000	51.6565000
age.s[4]	1.0004136	1.0015714	43.3094324	42.4864000	37.4089091	42168.8	0.0741373	0.95	15.5266000	70.2683000
age.s[5]	1.0000107	1.0000800	42.0224705	40.5256000	33.6486202	44299.6	0.0756792	0.95	15.0004000	70.4645000
age.s[6]	0.9999770	1.0000711	32.2383057	30.6458000	21.4219041	42090.5	0.0549183	0.95	15.0007000	52.9322000
age.s[7]	1.0002178	1.0007798	55.8914796	56.2833000	59.1956480	44073.1	0.0721750	0.95	27.5479000	84.3727000
age.s[8]	1.0000143	1.0001111	49.4264756	49.1496000	50.0585181	42053.3	0.0813289	0.95	18.7519000	79.8802000
age.s[9]	1.0000711	1.0004155	49.4078933	49.3554000	50.3756162	41590.3	0.0744921	0.95	20.8512000	76.7301000
age.s[10]	1.0000847	1.0004414	68.5147797	70.2500000	73.8035603	45648.9	0.0731837	0.95	37.0838000	95.7653000
age.s[11]	0.9999526	0.9999833	31.0874918	28.6554000	19.5238022	42841.5	0.0578469	0.95	15.0006000	53.9604000
age.s[12]	1.0000949	1.0003847	49.8459706	49.7920000	50.1208714	41087.3	0.0725104	0.95	22.0599000	76.7777000
age.s[13]	1.0000605	1.0000935	53.1345819	53.3550000	53.0652921	42312.6	0.0717077	0.95	25.2431000	80.5250000
age.s[14]	1.0002622	1.0007568	36.4811968	33.5349000	20.5878336	43651.4	0.0748482	0.95	15.0000000	66.1955000
age.s[15]	1.0000837	1.0000906	50.7019353	50.7137000	52.2029535	41825.6	0.0728524	0.95	22.6111000	78.4532000
age.s[16]	1.0000970	1.0005117	32.3281515	30.1505000	21.2805517	44041.2	0.0583254	0.95	15.0005000	55.2935000
age.s[17]	1.0000808	1.0003736	75.2075264	77.1407000	82.9471457	47324.6	0.0648712	0.95	48.6418000	99.9334000
age.s[18]	1.0002358	1.0009778	54.4645528	54.8233000	55.9957156	43720.1	0.0760134	0.95	23.6012000	82.8785000
age.s[19]	1.0001069	1.0005186	59.9428846	60.6614000	62.0062947	43762.6	0.0761461	0.95	28.8305000	88.6774000
age.s[20]	1.0000497	1.0003387	56.8601993	57.4721000	59.6500577	44722.7	0.0808775	0.95	24.4487000	88.4874000
age.s[21]	1.0000688	1.0003310	41.4507176	39.8756000	28.6131769	43823.0	0.0763519	0.95	15.0279000	70.3772000
age.s[22]	1.0000707	1.0002425	68.8723382	70.3754000	71.9884840	45873.9	0.0684909	0.95	39.6474000	95.2923000
age.s[23]	1.0001497	1.0003136	54.9664894	55.4451000	60.3048821	43136.3	0.0744970	0.95	25.0982000	83.1850000
age.s[24]	0.9999955	1.0000226	63.8784151	65.0794000	68.7829230	46156.1	0.0705683	0.95	34.0547000	91.4332000
age.s[25]	0.9999677	1.0000075	75.8553005	77.5122000	81.8017296	43729.9	0.0608131	0.95	51.0980000	98.6902000
age.s[26]	1.0000238	1.0000989	46.5141865	46.2087000	46.3654053	41245.6	0.0736409	0.95	18.6587000	73.9427000
age.s[27]	1.0001356	1.0003802	67.2724684	69.1533000	73.6366913	44728.5	0.0733106	0.95	36.2278000	94.8866000
age.s[28]	1.0001347	1.0003996	56.6953235	57.3890000	61.7208111	43609.6	0.0818374	0.95	23.3872000	87.2854000
age.s[29]	1.0002007	1.0008430	68.5557259	70.3930000	75.4214362	46719.2	0.0736681	0.95	37.0631000	96.5908000
age.s[30]	1.0000244	1.0001098	69.5720838	70.9564000	75.3012668	44255.7	0.0672521	0.95	41.2132000	94.8647000
age.s[31]	1.0000938	1.0002711	75.9747463	77.6367000	81.1504377	44669.1	0.0616606	0.95	51.1272000	99.2719000
age.s[32]	1.0001176	1.0003442	59.8160544	60.8135000	64.5429135	43076.3	0.0741829	0.95	29.3621000	87.4741000
age.s[33]	1.0001849	1.0005859	80.5530790	82.0120000	85.1399055	46352.7	0.0511010	0.95	59.9574000	99.9329000

age.s[34]	1.0000427	1.0001116	69.7513371	71.1246000	74.4009834	46674.3	0.0627134	0.95	42.3277000	93.7905000
age.s[35]	1.0001025	1.0003953	30.4966472	27.7062000	18.1424235	44175.2	0.0574692	0.95	15.0002000	53.9822000
age.s[36]	1.0001032	1.0005095	67.1533075	69.0899000	73.6343503	47048.5	0.0766649	0.95	34.6467000	96.8138000
age.s[37]	1.0000133	1.0001708	50.0613568	49.9410000	50.6888939	42805.2	0.0804072	0.95	18.0594000	79.9339000
age.s[38]	1.0001946	1.0004149	80.5823243	82.3808000	85.5737512	48316.1	0.0530647	0.95	58.5853000	99.9965000
age.s[39]	1.0000818	1.0003869	45.9314312	45.5415000	49.5163269	39578.1	0.0740345	0.95	18.4874000	72.6455000
age.s[40]	1.0002818	1.0011936	49.0097860	49.0971000	49.0773940	42484.2	0.0767840	0.95	18.7953000	77.4562000
age.s[41]	1.0003548	1.0014589	61.1155427	62.2523000	66.4243569	35160.3	0.0791454	0.95	31.0915000	87.1411000
age.s[42]	1.0002871	1.0011854	65.6175213	67.6543000	73.3352591	47550.1	0.0793104	0.95	31.2230000	96.1814000
age.s[43]	1.0001023	1.0005169	67.0764379	68.9863000	75.1216113	46096.9	0.0772550	0.95	34.3634000	96.6704000
age.s[44]	1.0000531	1.0002504	54.8895454	55.1804000	59.0540035	43987.4	0.0760126	0.95	24.2358000	84.1909000
age.s[45]	1.0000965	1.0005014	75.8715621	77.4009000	80.6518743	43061.6	0.0611437	0.95	52.1590000	99.6940000
age.s[46]	1.0002219	1.0009687	54.1898785	54.5951000	59.4183397	43039.1	0.0757443	0.95	23.9681000	82.7954000
age.s[47]	1.0000883	1.0004736	61.3946894	62.6335000	67.4440607	45175.5	0.0757471	0.95	29.3812000	90.1475000
age.s[48]	1.0000912	1.0002705	64.8053745	65.8996000	66.9900600	44166.4	0.0673394	0.95	36.1222000	89.8035000
age.s[49]	1.0002923	1.0011454	76.1789379	77.7442000	82.2659180	43666.6	0.0611909	0.95	51.7561000	99.2963000
age.s[50]	1.0000266	1.0002517	68.7532229	70.1859000	74.3992530	46060.4	0.0675096	0.95	39.9190000	94.6975000
age.s[51]	1.0002009	1.0005569	65.5332426	67.4004000	76.3121911	47327.3	0.0789421	0.95	31.8243000	96.2098000
age.s[52]	1.0001629	1.0004960	70.8682457	72.4010000	76.8287581	46646.5	0.0648208	0.95	42.7001000	95.8214000
age.s[53]	1.0000727	1.0001453	69.4436711	70.7972000	74.8946310	44651.6	0.0664568	0.95	41.0198000	94.4606000
age.s[54]	1.0000067	1.0001684	50.0707913	49.8602000	50.2971595	43069.8	0.0799563	0.95	19.3703000	80.8266000
age.s[55]	1.0001682	1.0007764	47.5504702	47.3919000	48.3378893	40292.3	0.0732736	0.95	20.5275000	74.9420000
age.s[56]	1.0000073	1.0001015	74.2270014	75.7051000	79.5066656	42332.2	0.0610341	0.95	49.2716000	96.7799000
age.s[57]	1.0000483	1.0001918	71.2343700	72.8621000	76.3905831	39604.3	0.0710817	0.95	42.7494000	96.2580000
age.s[58]	1.0000320	1.0002677	46.4953429	46.1389000	46.2324219	38849.9	0.0750738	0.95	19.2993000	73.6551000
age.s[59]	0.9999747	0.9999979	68.8591556	70.2802000	76.7924623	46501.5	0.0678031	0.95	39.7181000	95.0111000
age.s[60]	1.0000952	1.0004757	63.9792917	65.2465000	69.7068036	45419.8	0.0709265	0.95	33.6564000	90.8757000
age.s[61]	1.0001338	1.0004483	64.2109658	65.2303000	69.8019292	45404.1	0.0666813	0.95	35.9995000	90.0764000
age.s[62]	0.9999821	1.0000538	64.8798630	66.0444000	69.4561308	45056.1	0.0662787	0.95	37.0086000	90.5916000
age.s[63]	1.0001763	1.0007894	53.2189939	53.4589000	54.4231251	43213.6	0.0739398	0.95	23.5882000	80.9835000
age.s[64]	1.0001243	1.0004076	54.3191408	54.6763000	57.6729580	43591.2	0.0788158	0.95	22.3281000	83.7830000
age.s[65]	1.0001192	1.0005883	68.0214140	69.5155000	73.2641972	46285.5	0.0682819	0.95	39.0780000	94.9118000
age.s[66]	1.0000960	1.0003123	71.1865075	72.7578000	78.5387757	41494.1	0.0690103	0.95	43.3247000	96.6107000
age.s[67]	1.0001133	1.0004875	63.7008032	64.8037000	69.5946156	44892.8	0.0726776	0.95	33.1429000	91.2763000
age.s[68]	1.0000323	1.0001826	75.0464130	77.0457000	82.9164482	45909.8	0.0662355	0.95	48.2777000	99.9714000
age.s[69]	1.0002153	1.0008618	64.0851495	65.1010000	65.5288129	45495.4	0.0676458	0.95	35.3501000	90.3740000
age.s[70]	1.0003237	1.0013023	60.9149013	62.0774000	65.2106280	45718.8	0.0767111	0.95	28.9491000	90.6560000
age.s[71]	0.9999978	1.0000659	68.9932942	70.3276000	74.2466102	45926.7	0.0644861	0.95	41.2439000	93.6737000
age.s[72]	1.0000573	1.0001731	66.5810513	68.2817000	73.6322256	47494.5	0.0733687	0.95	34.5310000	94.8747000

age.s[73]	1.0002797	1.0005985	80.5171140	82.0248000	84.8546793	44684.5	0.0524831	0.95	59.7764000	99.9991000
age.s[74]	0.9999860	1.0000945	50.8115431	50.9798000	53.4643610	43414.7	0.0718122	0.95	22.0536000	77.8972000
age.s[75]	1.0002368	1.0007672	72.1443177	73.7783000	75.9691844	46816.0	0.0651239	0.95	44.9682000	98.2371000
age.s[76]	0.9999648	0.9999746	75.2964467	76.9693000	80.6158562	44825.2	0.0619560	0.95	49.9712000	98.7973000
age.s[77]	1.0000560	1.0002003	64.8217497	65.9267000	70.6275076	45733.0	0.0668935	0.95	36.2031000	90.8329000
age.s[78]	0.9999878	1.0001124	57.6662866	58.5538000	61.1662171	46040.2	0.0846563	0.95	23.0112000	90.3450000
age.s[79]	1.0000169	1.0001676	59.1768021	60.1542000	63.3840747	44337.5	0.0801573	0.95	25.4875000	88.8921000
age.s[80]	1.0000026	1.0001258	70.4925821	72.2497000	77.7177627	47028.2	0.0693223	0.95	40.8081000	97.5676000
age.s[81]	1.0000820	1.0003939	59.1000707	59.9424000	65.4532083	45031.5	0.0765174	0.95	27.6819000	88.0269000
age.s[82]	1.0001637	1.0006060	87.7406247	89.2870000	92.7704530	44494.7	0.0405045	0.95	71.3527000	99.9998000
age.s[83]	1.0001094	1.0003446	80.3443549	81.8347000	85.8121124	47068.6	0.0510608	0.95	59.6415000	99.9409000
age.s[84]	1.0000425	1.0003074	74.8076173	76.1868000	78.6676971	43408.7	0.0598507	0.95	50.0577000	97.2009000
age.s[85]	1.0001614	1.0007089	68.8263530	70.1939000	72.7397408	46265.9	0.0670412	0.95	40.4635000	95.2053000
age.s[86]	1.0001046	1.0004089	76.1447070	77.8383000	82.6800573	48420.6	0.0592115	0.95	51.1764000	99.3329000
age.s[87]	1.0000339	1.0001701	59.1894228	60.2494000	64.4279297	45080.0	0.0794696	0.95	26.2094000	89.4892000
age.s[88]	1.0000525	1.0001640	75.7888329	77.3960000	81.2453719	45433.8	0.0605004	0.95	51.3159000	99.3005000
age.s[89]	1.0001778	1.0006123	75.2167589	76.8562000	80.9331668	44145.4	0.0619366	0.95	49.6940000	98.1232000
age.s[90]	1.0000675	1.0001765	76.4083721	78.2103000	83.7860386	46844.0	0.0602516	0.95	51.9129000	99.7868000
age.s[91]	1.0000105	1.0000289	79.9923354	81.6330000	84.1316287	47023.6	0.0534789	0.95	58.1421000	99.9551000
age.s[92]	1.0002434	1.0007403	77.2291618	78.9291000	82.4425504	47531.6	0.0577578	0.95	53.4733000	99.8114000
age.s[93]	1.0000858	1.0003556	71.8227888	73.2264000	77.4797359	46400.3	0.0622876	0.95	44.7796000	95.6658000
age.s[94]	0.9999901	1.0000964	77.2201409	78.9589000	82.0572226	47032.0	0.0581638	0.95	53.2716000	99.5540000
age.s[95]	1.0000472	1.0003197	26.8682748	24.6309000	17.0903300	41672.3	0.0461969	0.95	15.0012000	45.2389000
age.s[96]	1.0000905	1.0004602	37.9008681	35.2644000	20.1085327	42380.9	0.0776544	0.95	15.0003000	67.8725000
age.s[97]	1.0002864	1.0011756	42.2254897	40.7130000	33.5258238	39224.2	0.0802915	0.95	15.0124000	70.6312000
age.s[98]	1.0000703	1.0004169	48.5301947	48.3946000	51.0432358	40717.9	0.0757000	0.95	20.5296000	76.7661000
age.s[99]	1.0004093	1.0014380	41.3257421	40.4670000	34.8105670	40129.6	0.0717858	0.95	15.4072000	66.4638000
age.s[100]	0.9999777	1.0000477	19.4534166	17.8700000	15.7073711	47473.2	0.0221629	0.95	15.0000000	29.0079000
age.s[101]	1.0000726	1.0003292	27.6137419	25.2011000	17.6454106	43238.4	0.0482451	0.95	15.0002000	47.2028000
age.s[102]	1.0001131	1.0004459	61.7949937	63.0405000	70.8857422	47081.3	0.0790650	0.95	28.6683000	92.7957000
age.s[103]	1.0002378	1.0008581	68.8010256	70.1434000	74.1176851	46367.2	0.0673781	0.95	40.4504000	95.2922000
age.s[104]	0.9999635	0.9999890	67.7193600	69.3349000	73.0373558	47176.1	0.0708756	0.95	37.1026000	95.3005000
age.s[105]	1.0001502	1.0006146	35.7402267	34.3773000	26.0792479	43783.3	0.0598137	0.95	15.0056000	58.1408000
age.s[106]	1.0001925	1.0008180	41.7219914	40.3241000	34.4557305	41015.6	0.0762434	0.95	15.0144000	69.1819000
age.s[107]	0.9999489	0.9999522	51.9697172	52.2280000	55.1439638	45273.7	0.0859796	0.95	16.7164000	83.1660000
age.s[108]	1.0001400	1.0006546	46.4512377	46.0957000	44.3026006	41694.3	0.0727098	0.95	18.8637000	73.3529000
age.s[109]	1.0000904	1.0004375	31.5702533	29.5002000	19.5392472	42237.3	0.0568853	0.95	15.0007000	53.5199000
age.s[110]	1.0001675	1.0007114	36.0814700	33.4751000	19.3551349	43910.6	0.0715781	0.95	15.0025000	64.2181000
age.s[111]	1.0001786	1.0006423	67.7850439	69.3609000	72.9581482	39044.2	0.0764918	0.95	36.9748000	94.3095000

age.s[112]	0.9999587	1.0000044	34.9138841	33.0134000	25.3367191	43971.0	0.0616753	0.95	15.0024000	58.6093000
age.s[113]	1.0001168	1.0002131	56.7682017	57.3730000	61.3935222	44432.2	0.0806973	0.95	24.0688000	87.8603000
age.s[114]	0.9999999	1.0001078	51.1421081	51.2331000	55.8210510	41809.2	0.0794844	0.95	20.5859000	81.1323000
age.s[115]	1.0002172	1.0009484	54.9909826	55.5243000	60.1858610	42593.7	0.0747177	0.95	25.3540000	83.1438000
age.s[116]	1.0000304	1.0001919	45.4774818	44.8276000	44.8187440	40473.5	0.0754941	0.95	17.7009000	73.1971000
age.s[117]	1.0000406	1.0002945	54.4427800	54.6972000	58.5682194	43429.9	0.0763027	0.95	24.1486000	83.5771000
age.s[118]	1.0000647	1.0003711	64.0134835	65.2088000	69.1125002	45838.3	0.0711340	0.95	33.4638000	91.3570000
age.s[119]	1.0001616	1.0005703	54.7827208	55.2883000	60.8782062	44355.1	0.0792191	0.95	22.7691000	85.0087000
age.s[120]	1.0000248	1.0000871	45.0783234	43.5958000	37.8835558	45239.7	0.0818896	0.95	15.1050000	76.2880000
age.s[121]	1.0004877	1.0018638	53.8341469	54.2907000	57.1640968	42158.2	0.0763231	0.95	23.5674000	81.5580000
age.s[122]	1.0000813	1.0003887	75.1111478	76.7513000	79.9493518	43966.8	0.0622677	0.95	49.9253000	98.8083000
age.s[123]	1.0001313	1.0005004	54.0021086	54.3184000	55.4563181	42150.3	0.0743782	0.95	24.6117000	81.5899000
age.s[124]	0.9999945	1.0000527	68.9771196	70.3032000	73.7636534	46452.3	0.0640092	0.95	41.0347000	93.6058000
age.s[125]	1.0001753	1.0007979	43.2304455	42.5189000	39.2772792	39633.5	0.0739522	0.95	16.2316000	69.4109000
age.s[126]	1.0000223	1.0001928	56.7367790	57.4458000	63.6790285	43991.9	0.0816731	0.95	24.4172000	88.4435000
age.s[127]	1.0001675	1.0005920	66.2514006	67.5127000	69.7158156	45826.8	0.0663760	0.95	37.7657000	92.0906000
age.s[128]	0.9999870	1.0000812	67.0457627	68.6393000	74.7785776	46724.7	0.0706278	0.95	37.4292000	95.2294000
age.s[129]	1.0001924	1.0008354	55.2087350	55.9366000	57.9738252	44308.2	0.0902300	0.95	17.9534000	87.3852000
age.s[130]	1.0000468	1.0001892	80.4256469	81.8977000	85.0463888	47237.8	0.0508644	0.95	59.9801000	99.9945000
age.s[131]	0.9999757	1.0000484	52.3294206	52.5248000	53.5216012	42455.3	0.0774567	0.95	22.0282000	81.8577000
age.s[132]	1.0002687	1.0010088	68.2138427	69.5461000	71.4779419	38752.1	0.0722950	0.95	39.9580000	94.1332000
age.s[133]	1.0000052	1.0001346	75.0282729	76.4488000	78.7541656	42888.1	0.0596766	0.95	50.6125000	97.2883000
age.s[134]	1.0002499	1.0008224	67.7171356	69.2065000	70.9858768	39352.6	0.0726643	0.95	38.6084000	93.3756000
age.s[135]	1.0000879	1.0000914	52.3822448	52.6376000	54.4120851	43322.3	0.0766326	0.95	22.3578000	81.8785000
age.s[136]	0.9999903	1.0000317	63.9434159	65.0580000	66.5265820	45761.8	0.0706020	0.95	32.7884000	90.0814000
age.s[137]	1.0000821	1.0004571	59.1334646	59.9260000	60.4032411	43814.6	0.0736881	0.95	28.4601000	86.4230000
age.s[138]	1.0002101	1.0008107	40.6544416	39.6131000	36.7304478	42694.1	0.0698485	0.95	15.0706000	66.0783000
age.s[139]	1.0001133	1.0004136	65.8406220	67.3609000	70.4369843	38451.4	0.0773762	0.95	34.7948000	92.8572000
age.s[140]	1.0003694	1.0012097	74.8548614	76.6014000	79.6941570	43394.7	0.0643525	0.95	49.3037000	99.1288000
age.s[141]	1.0000143	1.0000987	76.7724934	78.4839000	81.8337683	47865.8	0.0585281	0.95	52.7665000	99.9497000
age.s[142]	1.0002893	1.0009780	80.3620594	81.9142000	84.7474037	46571.9	0.0519619	0.95	59.3642000	99.9993000
age.s[143]	0.9999726	1.0000168	75.9554563	77.6940000	81.4555744	46219.6	0.0608109	0.95	51.0096000	99.2855000
age.s[144]	0.9999710	1.0000123	38.0147669	36.3947000	29.5330944	44981.0	0.0662594	0.95	15.0026000	63.2803000
age.s[145]	1.0001501	1.0005797	61.9933754	62.9386000	67.0830402	44229.0	0.0671573	0.95	33.3391000	86.9726000
age.s[146]	1.0000842	1.0004111	64.3343082	65.4291000	69.1459891	45964.3	0.0659690	0.95	36.2221000	90.0065000
age.s[147]	0.9999657	1.0000047	61.8083802	63.0361000	66.0213234	45216.5	0.0805906	0.95	28.1113000	92.1734000
age.s[148]	1.0001333	1.0006128	67.0268820	69.1677000	76.3152361	46218.8	0.0791561	0.95	33.6100000	97.3397000
age.s[149]	1.0001324	1.0003719	80.0207799	81.6947000	85.8498689	45477.6	0.0547770	0.95	58.0337000	99.9998000
age.s[150]	1.0000179	1.0000293	74.7514513	76.5432000	79.3841559	43319.6	0.0643796	0.95	48.5929000	98.5342000

age.s[151]	1.0000179	1.0000846	78.9945492	80.7721000	84.4859967	47269.8	0.0558578	0.95	56.1343000	99.9973000
age.s[152]	1.0001764	1.0007221	77.3890132	78.9672000	81.8233570	44168.0	0.0574423	0.95	53.9533000	98.9346000
age.s[153]	1.0000464	1.0002675	59.9128912	60.7265000	64.1087847	43590.5	0.0762667	0.95	28.9871000	88.9262000
age.s[154]	1.0000865	1.0003910	68.5628128	69.8586000	73.2180948	46324.2	0.0638091	0.95	41.0065000	93.0046000
age.s[155]	1.0001344	1.0005810	59.1669269	60.2108000	61.4499214	44805.3	0.0799528	0.95	25.7849000	89.1145000
age.s[156]	1.0001760	1.0002690	67.7213919	69.0804000	72.1003226	38066.4	0.0736812	0.95	39.1532000	93.7234000
age.s[157]	1.0000610	1.0002611	52.3390587	52.5927000	52.7774279	42493.0	0.0772888	0.95	21.0290000	80.8637000
age.s[158]	1.0001650	1.0004243	74.7690753	76.1799000	78.4765161	43088.2	0.0600356	0.95	49.9774000	97.4051000
age.s[159]	1.0001290	1.0004692	71.4627336	73.0054000	77.4235076	46843.7	0.0627709	0.95	44.4254000	95.9996000
age.s[160]	0.9999786	0.9999965	80.4643535	82.1587000	86.2587636	45203.3	0.0550003	0.95	58.5084000	99.9995000
age.s[161]	1.0001175	1.0005401	56.7755877	57.3421000	60.5474327	44976.9	0.0802848	0.95	23.5052000	87.3501000
age.s[162]	1.0002339	1.0008117	75.9754289	77.8456000	81.5954235	48537.5	0.0605759	0.95	50.8777000	99.9030000
age.s[163]	0.9999809	1.0000279	65.3461618	66.4796000	71.9516715	45514.1	0.0684323	0.95	36.8402000	92.0738000
age.s[164]	1.0000588	1.0002218	54.1870405	54.6665000	58.1819695	43500.9	0.0756565	0.95	22.7736000	82.0472000
age.s[165]	0.9999945	1.0001147	65.6359257	66.7762000	70.5111022	45143.8	0.0655686	0.95	38.5012000	91.4363000
age.s[166]	1.0000133	1.0001925	63.0402041	64.1955000	68.0953315	46193.4	0.0691234	0.95	33.7882000	90.4321000
age.s[167]	1.0000901	1.0004563	70.8835737	72.3828000	74.4436456	47607.5	0.0643817	0.95	43.0576000	96.2384000
age.s[168]	1.0000684	1.0002661	57.2818748	57.8550000	59.3824624	43836.9	0.0712511	0.95	28.1040000	84.3355000
age.s[169]	1.0000879	1.0003534	74.3353686	75.8146000	78.1985998	43364.4	0.0599616	0.95	49.6773000	97.1678000
age.s[170]	1.0000184	1.0000486	73.8068727	75.3921000	78.9737891	47002.3	0.0618358	0.95	47.0987000	97.5872000
age.s[171]	1.0001957	1.0005428	64.3000602	65.4065000	70.9378000	45875.0	0.0663744	0.95	35.7114000	89.7255000
age.s[172]	1.0001667	1.0007327	59.1159871	59.8327000	61.8468843	44656.2	0.0730925	0.95	28.9998000	87.3214000
age.s[173]	1.0000916	1.0004043	64.8820405	66.4294000	73.5378464	46502.2	0.0781350	0.95	31.3329000	94.4973000
age.s[174]	0.9999962	1.0001103	59.1613481	60.1355000	63.5563201	45611.9	0.0788856	0.95	25.9237000	89.2353000
age.s[175]	1.0001401	1.0004839	79.5778493	81.1793000	84.1801619	46054.0	0.0543025	0.95	57.4657000	99.9620000
age.s[176]	0.9999875	1.0001015	59.2019998	60.2057000	62.5299560	44925.6	0.0799151	0.95	26.1901000	89.7582000
age.s[177]	0.9999509	0.9999788	52.3410074	52.6068000	52.3660677	41766.6	0.0783241	0.95	22.0055000	81.7359000
age.s[178]	1.0001284	1.0004897	68.5044869	70.3438000	76.7281301	46573.6	0.0739226	0.95	37.1906000	96.9895000
age.s[179]	1.0002352	1.0007929	63.5039429	64.9105000	71.5875214	45147.5	0.0775229	0.95	31.0377000	92.8310000
age.s[180]	1.0000403	1.0001042	77.2082690	78.7915000	82.9431264	47851.5	0.0572689	0.95	53.3558000	99.4181000

3.1.6 50% missing

Table 30: MCMC diagnostic data Spitalfields dataset (50 % missing).

	PSRF Point est.	PSRF Upper C.I.	Mean	Median	Mode	ESS	MCSE	HDImass	HDIlow	HDIhigh
b	1.0004954	1.0010570	0.0266840	0.0248032	0.0211085	48757.7	0.0000291	0.95	0.0200003	0.0393484
a	1.0002041	1.0006786	0.0177029	0.0186665	0.0241392	50001.0	0.0000252	0.95	0.0070678	0.0257238
M	1.0001858	1.0006596	28.3902427	26.4600000	9.2530602	50001.0	0.0752298	0.95	2.4167500	58.6335000
beta0[1]	1.0002787	1.0011785	-10.5180454	-9.7231500	-8.2796598	35364.3	0.0220697	0.95	-19.2664000	-3.8809500
beta0[2]	1.0004190	1.0013611	-7.3700042	-6.7676500	-5.7240347	42495.8	0.0214814	0.95	-15.6137000	-0.0033887
beta0[3]	1.0002061	1.0005049	-8.0435237	-7.3131400	-6.2693735	44923.1	0.0194233	0.95	-16.4647000	-1.2736800
beta0[4]	1.0003661	1.0008640	-12.1003800	-11.3580000	-10.0311129	47659.6	0.0192655	0.95	-20.7322000	-5.2168400
beta0[5]	1.0000258	1.0000869	-2.9955150	-2.8607000	-2.6477013	50001.0	0.0068846	0.95	-5.7659300	-0.0867773
beta[1]	1.0003279	1.0013214	4.0236738	3.7569100	3.3925291	34738.3	0.0072861	0.95	1.8683100	6.8197100
beta[2]	1.0007461	1.0024932	4.4038576	4.1564300	3.6979194	25210.2	0.0088509	0.95	2.1014300	7.2646700
beta[3]	1.0002591	1.0005851	3.0509199	2.7990200	2.4253576	44763.1	0.0062416	0.95	0.8879370	5.6890100
beta[4]	1.0002828	1.0007239	3.2711181	3.0948800	2.7452813	47576.7	0.0048207	0.95	1.5109400	5.4183500
beta[5]	1.0000152	1.0000786	1.1629725	1.1306600	1.0931643	50001.0	0.0018590	0.95	0.3924720	1.9453400
thresh[1,1]	NaN	NaN	0.5000000	0.5000000	0.4996965	0.0	NaN	0.95	0.5000000	0.5000000
thresh[2,1]	NaN	NaN	0.5000000	0.5000000	0.4996965	0.0	NaN	0.95	0.5000000	0.5000000
thresh[3,1]	NaN	NaN	0.5000000	0.5000000	0.4996965	0.0	NaN	0.95	0.5000000	0.5000000
thresh[4,1]	NaN	NaN	0.5000000	0.5000000	0.4996965	0.0	NaN	0.95	0.5000000	0.5000000
thresh[5,1]	NaN	NaN	0.5000000	0.5000000	0.4996965	0.0	NaN	0.95	0.5000000	0.5000000
thresh[1,2]	1.0001707	1.0006723	2.5476707	2.4199000	2.2228450	39948.8	0.0035950	0.95	1.3846500	3.9780500
thresh[2,2]	1.0002936	1.0009733	6.4310428	6.0052500	4.8714787	15055.4	0.0213517	0.95	2.1516400	11.5309000
thresh[3,2]	1.0000384	1.0000679	1.7376360	1.6631000	1.5787360	46678.1	0.0020630	0.95	0.9953350	2.6182500
thresh[4,2]	1.0001073	1.0003287	1.8103671	1.7697300	1.6776844	48856.1	0.0014385	0.95	1.2425700	2.4399200
thresh[5,2]	0.9999956	1.0000246	2.6981530	2.6838100	2.6359990	49067.9	0.0011838	0.95	2.2064600	3.2201200
thresh[1,3]	1.0001300	1.0005102	3.7834413	3.5918400	3.3292831	36115.2	0.0051774	0.95	2.1845600	5.7355900
thresh[2,3]	1.0005428	1.0018402	10.1982606	9.7569000	8.7730918	13890.9	0.0261960	0.95	4.9621400	16.2916000
thresh[1,4]	1.0003376	1.0011654	7.0895326	6.7331800	6.3705831	34478.7	0.0092202	0.95	4.4762400	10.6042000
thresh[2,4]	1.0006203	1.0021098	13.1685618	12.6832000	11.3402414	14383.7	0.0288610	0.95	7.3076900	20.1008000
thresh_age[1,1]	1.0002460	1.0005084	15.2252068	15.0759000	15.1099505	48447.7	0.0129138	0.95	9.6110400	20.7214000
thresh_age[2,1]	1.0001261	1.0004127	6.5639611	6.1702700	3.1221585	33176.7	0.0192568	0.95	1.1581200	12.7818000
thresh_age[3,1]	1.0000478	1.0000665	15.9203120	15.9646000	16.2868405	49039.7	0.0173697	0.95	7.7395200	23.4842000
thresh_age[4,1]	1.0001151	1.0003236	46.9485611	45.8718000	44.2057545	48700.0	0.0285416	0.95	36.0925000	59.8534000
thresh_age[5,1]	1.0000686	1.0001998	19.3600742	19.3226000	19.3050607	50001.0	0.0269963	0.95	6.6202300	30.9324000
thresh_age[1,2]	1.0001266	1.0001288	25.6207457	24.7201000	23.5171963	47647.0	0.0202295	0.95	18.4184000	34.8432000
thresh_age[2,2]	1.0004245	1.0007723	23.0314112	22.2288000	21.2689769	47325.9	0.0176915	0.95	17.0367000	31.0597000
thresh_age[3,2]	1.0000193	1.0001344	24.3904578	23.8689000	23.2093230	48089.8	0.0209791	0.95	15.8499000	34.4535000

thresh_age[4,2]	1.0000971	1.0003467	71.5538472	70.3455000	68.3578038	50001.0	0.0418557	0.95	55.3257000	89.7029000
thresh_age[5,2]	1.0227536	1.0229545	194.0613770	134.7640000	110.2985637	48375.0	1.4773725	0.95	65.9372000	450.7340000
thresh_age[1,3]	1.0002380	1.0005547	35.0213283	33.8522000	32.1215125	48663.3	0.0245709	0.95	26.4093000	46.3976000
thresh_age[2,3]	1.0000729	1.0003829	54.6454244	53.5093000	51.1082715	50001.0	0.0299942	0.95	43.6163000	68.8138000
thresh_age[1,4]	1.0011037	1.0025952	81.5605613	80.1460000	76.9819675	48925.2	0.0483602	0.95	62.8353000	102.3860000
thresh_age[2,4]	1.0009959	1.0015845	111.2196457	107.2450000	99.9613222	38787.9	0.1057441	0.95	79.0538000	152.1220000
age.s[1]	1.0000456	1.0003150	18.7976170	17.5956000	15.6122787	50001.0	0.0178443	0.95	15.0001000	26.4645000
age.s[2]	1.0001737	1.0006962	34.2595380	31.6775000	20.0973558	49443.5	0.0633086	0.95	15.0006000	61.2938000
age.s[3]	1.0002816	1.0004349	17.6366679	16.8903000	15.4270123	50001.0	0.0114447	0.95	15.0000000	22.6552000
age.s[4]	1.0002756	1.0003541	21.9542378	20.7092000	18.3423626	50001.0	0.0252417	0.95	15.0004000	32.7247000
age.s[5]	1.0002816	1.0005519	18.2409706	17.2673000	15.5326339	50001.0	0.0146753	0.95	15.0000000	24.6224000
age.s[6]	1.0000291	1.0000384	20.2474049	19.3389000	17.4496653	45476.1	0.0195756	0.95	15.0001000	28.2252000
age.s[7]	0.9999666	1.0000265	52.8466914	51.5542000	46.2010726	50001.0	0.0664176	0.95	25.9395000	82.1731000
age.s[8]	1.0004330	1.0010703	25.1602196	23.5087000	20.5851022	50001.0	0.0336364	0.95	15.0001000	39.5970000
age.s[9]	1.0002088	1.0007399	28.5574647	27.0745000	25.3556362	49191.0	0.0368493	0.95	15.0038000	44.1118000
age.s[10]	1.0001073	1.0005273	41.0617014	39.9359000	38.4021729	50001.0	0.0457884	0.95	22.8762000	61.8566000
age.s[11]	1.0000566	1.0000861	21.9526812	20.1875000	16.2075592	50001.0	0.0289602	0.95	15.0004000	34.3997000
age.s[12]	1.0007452	1.0014308	24.8147763	22.9378000	19.6064972	48452.9	0.0360045	0.95	15.0000000	40.0991000
age.s[13]	1.0000739	1.0002011	34.0271537	29.6487000	18.3267373	50001.0	0.0721445	0.95	15.0002000	66.8520000
age.s[14]	0.9999836	1.0000284	24.1532101	23.0196000	20.8458799	43214.2	0.0304019	0.95	15.0003000	36.0459000
age.s[15]	1.0003891	1.0013864	26.3357429	24.8900000	22.7329909	50001.0	0.0338866	0.95	15.0025000	40.5905000
age.s[16]	0.9999815	1.0000312	21.9524417	20.2113000	16.3357009	48331.8	0.0294100	0.95	15.0000000	34.5102000
age.s[17]	1.0000221	1.0000279	60.2210700	59.6642000	59.4345019	50001.0	0.0655583	0.95	32.5608000	89.0946000
age.s[18]	0.9999894	1.0000403	34.1995434	31.4426000	18.9851841	50001.0	0.0631874	0.95	15.0009000	61.4522000
age.s[19]	1.0000898	1.0003962	42.0167671	40.8054000	38.3988431	50001.0	0.0453439	0.95	24.0231000	62.5275000
age.s[20]	0.9999885	1.0001049	39.0666566	37.0973000	33.3444624	50001.0	0.0617294	0.95	15.0020000	65.0607000
age.s[21]	1.0009007	1.0015867	30.3648950	28.9640000	26.5074187	50001.0	0.0372472	0.95	16.2322000	46.8820000
age.s[22]	1.0000854	1.0003355	46.6558735	44.8419000	36.5691153	48425.7	0.0862429	0.95	15.0012000	80.8637000
age.s[23]	0.9999732	1.0000362	40.0694217	38.2136000	34.3642081	50001.0	0.0602915	0.95	15.8899000	65.6296000
age.s[24]	1.0000702	1.0002177	43.1824625	41.4663000	38.6445255	50001.0	0.0581475	0.95	20.1704000	68.6522000
age.s[25]	1.0004913	1.0018441	34.2171892	31.5826000	19.3174822	51004.2	0.0618281	0.95	15.0010000	61.0602000
age.s[26]	1.0000323	1.0000428	40.0544278	38.1904000	33.7704793	50001.0	0.0607203	0.95	17.1873000	67.0863000
age.s[27]	1.0000794	1.0003919	58.3544616	57.3997000	54.6358261	48131.6	0.0560113	0.95	35.4301000	82.6740000
age.s[28]	0.9999771	1.0000093	38.4718331	37.1425000	34.8214449	50001.0	0.0481113	0.95	19.5710000	59.7555000
age.s[29]	1.0000187	1.0001494	46.3794557	44.9824000	42.5035427	50001.0	0.0523123	0.95	25.2633000	69.6324000
age.s[30]	0.9999532	0.9999858	51.7396107	50.4743000	46.8871654	48982.5	0.0689251	0.95	24.2346000	81.9754000
age.s[31]	1.0002574	1.0007593	61.9763581	61.2004000	60.2463003	50001.0	0.0549983	0.95	39.2138000	86.6033000
age.s[32]	1.0000261	1.0001870	36.6527997	35.0353000	32.1015128	49021.1	0.0450156	0.95	19.9338000	56.8792000
age.s[33]	1.0000563	1.0002457	70.8391421	70.7565000	70.0755879	50001.0	0.0569548	0.95	47.6105000	96.1022000

age.s[34]	1.0001156	1.0004428	55.1548977	54.2505000	52.3467320	50001.0	0.0647396	0.95	27.1267000	82.8025000
age.s[35]	1.0003711	1.0010022	25.4635714	23.7663000	20.9919994	45691.2	0.0369338	0.95	15.0006000	40.6025000
age.s[36]	1.0000276	1.0001812	48.9326068	47.8769000	45.4050766	50001.0	0.0486548	0.95	29.3006000	71.1527000
age.s[37]	0.9999591	0.9999788	33.9752845	29.5950000	18.3593799	50001.0	0.0721844	0.95	15.0017000	66.7861000
age.s[38]	0.9999669	0.9999847	50.5594037	48.9612000	42.9483907	50001.0	0.0789426	0.95	19.2491000	84.6360000
age.s[39]	1.0002074	1.0005506	25.1341244	23.5438000	20.4385346	51077.3	0.0331680	0.95	15.0004000	39.7064000
age.s[40]	1.0000940	1.0002475	34.8754092	33.3847000	31.4091072	50001.0	0.0417318	0.95	18.8395000	53.6333000
age.s[41]	0.9999815	1.0000728	51.8363299	50.5170000	47.2982503	50001.0	0.0665247	0.95	25.6049000	82.0066000
age.s[42]	1.0000631	1.0002436	46.6932667	44.8267000	38.6629710	50001.0	0.0845964	0.95	15.0064000	80.9934000
age.s[43]	1.0001316	1.0004294	53.6231056	52.7044000	50.1332542	48611.1	0.0510200	0.95	32.6644000	76.0753000
age.s[44]	1.0001401	1.0005922	40.9696123	39.3014000	34.9786064	50001.0	0.0600971	0.95	17.8253000	67.4969000
age.s[45]	0.9999697	1.0000131	53.6142626	52.6166000	49.2373170	48063.7	0.0559235	0.95	30.9811000	78.3642000
age.s[46]	1.0000825	1.0004182	45.5169431	44.2180000	40.2646972	50001.0	0.0528752	0.95	24.1582000	69.2833000
age.s[47]	1.0001592	1.0003498	38.4972416	37.1398000	33.5230113	49129.7	0.0483397	0.95	19.4405000	59.6816000
age.s[48]	1.0001187	1.0004691	35.9718700	34.5594000	31.3491078	48591.9	0.0423823	0.95	19.9491000	54.5846000
age.s[49]	1.0001707	1.0004943	54.4732938	53.5784000	51.2944894	49070.2	0.0570832	0.95	31.1490000	79.6265000
age.s[50]	0.9999733	0.9999736	60.8714003	60.1342000	58.3163013	48976.4	0.0567470	0.95	37.0194000	85.3834000
age.s[51]	0.9999742	1.0000490	45.2604568	43.9130000	41.4326964	48960.5	0.0542704	0.95	23.6908000	69.4181000
age.s[52]	1.0000893	1.0001534	51.7986021	50.5578000	48.3289062	50001.0	0.0681572	0.95	24.4154000	82.1267000
age.s[53]	1.0000500	1.0003211	45.5836950	44.3044000	41.5763526	50001.0	0.0528414	0.95	24.5946000	69.5214000
age.s[54]	1.0000422	1.0001870	35.3306133	33.8673000	29.8917544	50001.0	0.0493624	0.95	15.7503000	56.2668000
age.s[55]	1.0001310	1.0004055	46.5833333	44.7921000	39.0292636	49399.9	0.0845928	0.95	15.0119000	80.2677000
age.s[56]	1.0000646	1.0002053	54.1965960	53.3223000	50.0569650	50001.0	0.0669398	0.95	26.3897000	84.3951000
age.s[57]	0.9999578	0.9999813	50.0664449	48.2971000	41.9263590	50001.0	0.0821485	0.95	18.1820000	85.4165000
age.s[58]	1.0005075	1.0010927	26.2950699	25.0965000	23.0186089	48736.2	0.0296141	0.95	15.8217000	39.4407000
age.s[59]	0.9999562	0.9999980	60.6166882	59.8184000	57.1004926	50001.0	0.0512274	0.95	39.0828000	83.3071000
age.s[60]	1.0001302	1.0006335	49.8559943	48.2105000	41.0042357	50001.0	0.0819215	0.95	17.5130000	84.5310000
age.s[61]	1.0000123	1.0002057	48.8470741	47.7415000	45.1743015	50001.0	0.0489126	0.95	28.8625000	70.7482000
age.s[62]	1.0000637	1.0002151	45.6469937	44.2472000	42.9472811	48182.6	0.0542260	0.95	24.7119000	69.9349000
age.s[63]	1.0003467	1.0005747	34.3758139	32.9072000	29.7379206	50001.0	0.0416980	0.95	18.7396000	53.6797000
age.s[64]	1.0001377	1.0002969	44.6421713	43.2507000	40.7956595	50001.0	0.0538130	0.95	23.4960000	69.2481000
age.s[65]	1.0001213	1.0006039	60.1118709	59.2834000	56.9153130	48199.5	0.0517673	0.95	38.9462000	82.7464000
age.s[66]	0.9999704	0.9999984	33.8126161	32.4382000	30.3482740	48736.5	0.0414214	0.95	18.3529000	52.6320000
age.s[67]	0.9999609	1.0000112	52.8416177	51.5589000	49.7943965	50001.0	0.0660999	0.95	26.1713000	81.9079000
age.s[68]	0.9999674	1.0000219	70.3692038	70.1778000	69.5488426	49131.8	0.0547855	0.95	47.5897000	94.4251000
age.s[69]	1.0000185	1.0001657	55.1095049	54.1195000	53.0112640	51390.4	0.0626737	0.95	28.2092000	82.9689000
age.s[70]	1.0001847	1.0005355	33.8431727	29.5961000	18.2478056	50001.0	0.0712865	0.95	15.0005000	66.5410000
age.s[71]	1.0001303	1.0006058	50.0044306	48.3557000	41.9487828	50001.0	0.0820376	0.95	17.8074000	84.6014000
age.s[72]	1.0000676	1.0003434	45.2824671	43.8795000	41.0715332	49162.1	0.0541677	0.95	23.2640000	69.0209000

age.s[73]	0.9999866	1.0000917	70.2818763	70.0674000	68.0992594	50001.0	0.0567155	0.95	47.0308000	95.4705000
age.s[74]	1.0000171	1.0001179	46.7390246	44.9130000	39.8001781	49222.1	0.0852163	0.95	15.0039000	80.6830000
age.s[75]	1.0000333	1.0000697	55.7588150	54.7631000	51.4392124	47342.3	0.0617162	0.95	30.8566000	82.9533000
age.s[76]	1.0000681	1.0003700	67.1908862	66.8282000	66.3330966	50001.0	0.0532072	0.95	45.0099000	90.7967000
age.s[77]	1.0000715	1.0002136	48.9050690	47.8847000	45.7731090	50215.4	0.0490339	0.95	28.5856000	70.8909000
age.s[78]	1.0004161	1.0015616	50.1733063	48.6822000	41.7543752	50001.0	0.0820633	0.95	17.1878000	84.5214000
age.s[79]	1.0000715	1.0004081	38.1612489	36.7130000	33.2455520	47083.8	0.0492496	0.95	19.4815000	59.0933000
age.s[80]	0.9999899	1.0000535	77.8654360	78.4403000	79.7574227	50001.0	0.0518975	0.95	57.8044000	99.9439000
age.s[81]	1.0000551	1.0002898	34.2059853	31.5704000	19.6170081	50001.0	0.0629724	0.95	15.0010000	61.3283000
age.s[82]	1.0000106	1.0000474	86.5189879	87.6505000	92.3214875	48190.5	0.0396159	0.95	70.4627000	99.9957000
age.s[83]	1.0001963	1.0008580	67.6165563	67.2094000	65.1655220	50001.0	0.0535333	0.95	45.2846000	91.6989000
age.s[84]	0.9999959	1.0000410	69.3834045	69.1385000	67.8770584	47973.2	0.0566128	0.95	46.0155000	94.0522000
age.s[85]	1.0000329	1.0001686	51.7907646	50.4893000	47.0550668	51062.4	0.0675141	0.95	24.1198000	81.8374000
age.s[86]	1.0001453	1.0005699	70.3996230	70.2345000	67.6056015	50919.0	0.0555556	0.95	47.1308000	94.9499000
age.s[87]	0.9999737	1.0000052	39.1073113	37.0977000	32.9959418	50001.0	0.0614775	0.95	15.1167000	64.8096000
age.s[88]	1.0000279	1.0001544	63.8073841	63.2872000	59.7816600	50001.0	0.0627341	0.95	38.2261000	92.0863000
age.s[89]	1.0002853	1.0010461	67.6551960	67.2636000	66.4204721	50001.0	0.0535620	0.95	45.7449000	91.8586000
age.s[90]	0.9999900	1.0001143	61.4353631	60.8008000	60.2351048	50001.0	0.0566352	0.95	37.6522000	86.5267000
age.s[91]	1.0000136	1.0001710	73.9752385	74.1009000	75.0654133	51350.8	0.0523675	0.95	51.9913000	96.9650000
age.s[92]	1.0001888	1.0007636	67.5872975	67.1832000	65.1440794	52265.0	0.0525085	0.95	45.5007000	91.8641000
age.s[93]	1.0003105	1.0009441	45.5047895	44.0729000	39.5941249	48313.7	0.0509973	0.95	25.8465000	67.9726000
age.s[94]	1.0000676	1.0003398	63.8064434	63.2578000	61.7084403	48915.2	0.0635776	0.95	37.5772000	91.5879000
age.s[95]	1.0003429	1.0010635	20.9013766	19.8052000	17.4949537	50001.0	0.0215308	0.95	15.0000000	30.0674000
age.s[96]	1.0003188	1.0007063	23.6507995	22.1168000	18.8194657	47079.5	0.0308870	0.95	15.0010000	36.6964000
age.s[97]	1.0000064	1.0001531	35.3589105	33.8910000	30.0045711	50001.0	0.0495017	0.95	15.8493000	56.4402000
age.s[98]	1.0002659	1.0006079	26.5108330	25.3939000	24.4660378	51101.3	0.0309576	0.95	15.0019000	39.6262000
age.s[99]	1.0002806	1.0003780	20.8714152	19.5218000	16.5366000	50001.0	0.0233466	0.95	15.0000000	31.0129000
age.s[100]	1.0000358	1.0001171	34.1372788	31.4790000	20.3264332	49266.5	0.0630410	0.95	15.0003000	61.2139000
age.s[101]	0.9999896	1.0001114	18.8061037	17.7683000	15.6694785	50001.0	0.0164246	0.95	15.0001000	25.8444000
age.s[102]	1.0002137	1.0003388	32.5009309	31.1224000	29.2340768	50001.0	0.0415933	0.95	15.9778000	50.8072000
age.s[103]	1.0000654	1.0003074	48.1780308	47.0729000	45.3801437	50001.0	0.0524785	0.95	27.1913000	72.2836000
age.s[104]	0.9999473	0.9999612	46.4337485	44.6026000	39.2421236	49318.2	0.0847213	0.95	15.0016000	80.3650000
age.s[105]	1.0004747	1.0004858	21.7662124	20.5548000	18.0640629	50001.0	0.0243171	0.95	15.0001000	32.0883000
age.s[106]	1.0002463	1.0009718	28.5395176	25.5947000	17.4732146	50001.0	0.0509526	0.95	15.0001000	51.1511000
age.s[107]	1.0001263	1.0002803	29.7634021	28.3943000	25.7188068	43552.7	0.0405065	0.95	15.6825000	46.4867000
age.s[108]	0.9999572	0.9999986	34.8799750	32.8086000	27.6968678	50001.0	0.0536988	0.95	15.0655000	57.8142000
age.s[109]	1.0000636	1.0001948	26.0550449	24.9739000	23.5298915	47828.6	0.0299070	0.95	15.0170000	38.3700000
age.s[110]	1.0009933	1.0015112	21.4953028	19.1343000	16.0672113	51788.4	0.0327371	0.95	15.0000000	35.8608000
age.s[111]	1.0001574	1.0005245	54.0566511	52.9745000	51.2111345	47057.7	0.0559404	0.95	32.0486000	78.7488000

age.s[112]	1.0000333	1.0001974	28.5239796	25.6420000	17.4130698	50001.0	0.0511892	0.95	15.0003000	51.2650000
age.s[113]	1.0004247	1.0004295	24.8139305	22.9448000	19.2442641	49337.1	0.0355979	0.95	15.0001000	40.2382000
age.s[114]	0.9999865	1.0000774	33.8253100	31.8098000	27.6843461	50001.0	0.0533815	0.95	15.0044000	56.5381000
age.s[115]	0.9999696	0.9999832	46.3481952	44.9901000	41.7644574	50001.0	0.0516946	0.95	26.3287000	70.2637000
age.s[116]	0.9999549	0.9999917	41.0666530	39.8891000	38.3049719	48520.2	0.0465740	0.95	22.7569000	61.8900000
age.s[117]	1.0006527	1.0018849	25.9754569	24.7820000	23.3958454	48164.0	0.0308556	0.95	15.0009000	38.9273000
age.s[118]	1.0003461	1.0005442	34.8983212	32.8832000	28.5024196	50001.0	0.0536711	0.95	15.0222000	57.6619000
age.s[119]	1.0001528	1.0006851	39.9909981	38.2731000	33.4980661	50001.0	0.0605328	0.95	16.4699000	66.2246000
age.s[120]	1.0000238	1.0000345	27.2121244	25.9454000	24.1945185	48743.6	0.0330177	0.95	15.0231000	41.1455000
age.s[121]	1.0000888	1.0001943	27.7215495	24.7937000	20.1596388	50001.0	0.0489812	0.95	15.0032000	49.8652000
age.s[122]	1.0001062	1.0004862	51.8319848	50.6028000	46.6044342	47444.4	0.0700678	0.95	24.8765000	82.7837000
age.s[123]	0.9999776	1.0000532	35.4469623	31.8739000	24.8000267	50001.0	0.0655985	0.95	15.0031000	65.3060000
age.s[124]	0.9999907	1.0001004	58.7712435	59.0558000	59.1017412	50968.0	0.0821407	0.95	23.3989000	93.3957000
age.s[125]	1.0005773	1.0014092	23.6794579	22.2085000	18.8048523	46310.4	0.0310778	0.95	15.0014000	36.6947000
age.s[126]	1.0000062	1.0001317	39.3585761	38.0284000	37.2927744	49314.7	0.0515327	0.95	18.3180000	62.0758000
age.s[127]	1.0003167	1.0009534	28.6452835	27.1678000	24.4716034	48899.9	0.0361776	0.95	15.4873000	44.2902000
age.s[128]	1.0002475	1.0010670	54.9072341	53.9449000	50.7580922	50001.0	0.0564655	0.95	31.4435000	79.7704000
age.s[129]	1.0000920	1.0002778	34.6397490	33.1445000	29.7081863	50001.0	0.0443346	0.95	17.5568000	54.3610000
age.s[130]	0.9999602	0.9999884	77.1936796	77.6835000	78.2268910	50001.0	0.0522665	0.95	56.9531000	99.7010000
age.s[131]	1.0000636	1.0001914	37.8386421	36.3886000	34.4640517	50001.0	0.0484102	0.95	19.2955000	59.6659000
age.s[132]	0.9999865	1.0000736	53.4319580	52.4801000	49.9697545	48270.9	0.0557661	0.95	30.7611000	78.1933000
age.s[133]	1.0001429	1.0006376	63.3603407	62.7483000	59.5860393	49620.6	0.0622846	0.95	38.0182000	91.4495000
age.s[134]	1.0002200	1.0007466	27.7183697	24.7208000	20.2552412	50001.0	0.0493473	0.95	15.0001000	49.7994000
age.s[135]	1.0002786	1.0007842	35.4404145	33.8862000	31.1661062	50001.0	0.0498405	0.95	16.3393000	57.3123000
age.s[136]	1.0000134	1.0000652	43.1389989	41.4263000	36.0079293	50861.9	0.0577151	0.95	20.3185000	68.9129000
age.s[137]	1.0002825	1.0010552	34.2904999	33.0259000	31.2549366	50001.0	0.0371766	0.95	19.6418000	50.9704000
age.s[138]	0.9999836	1.0000846	46.6072788	44.7595000	36.1933078	49357.8	0.0850844	0.95	15.0093000	80.6579000
age.s[139]	1.0002411	1.0009961	49.3657769	48.3134000	46.7268636	44893.0	0.0515278	0.95	29.1537000	70.7227000
age.s[140]	1.0002449	1.0009251	61.7784044	60.9352000	60.1268147	50001.0	0.0549856	0.95	38.9894000	86.4222000
age.s[141]	1.0000284	1.0001613	67.5292927	67.0985000	67.2384866	50001.0	0.0538804	0.95	45.1028000	91.6201000
age.s[142]	0.9999961	1.0001262	63.4169452	62.9751000	61.3730017	50001.0	0.0629821	0.95	37.3577000	91.5052000
age.s[143]	1.0002705	1.0011461	63.8927681	63.3039000	62.7180015	50001.0	0.0625565	0.95	38.4943000	92.0706000
age.s[144]	1.0002424	1.0005792	23.6859395	22.3704000	19.8654899	47723.8	0.0296554	0.95	15.0000000	36.0224000
age.s[145]	1.0002737	1.0008159	34.3164007	32.8560000	30.8265600	50001.0	0.0415103	0.95	18.6714000	53.3970000
age.s[146]	1.0001439	1.0003457	54.9494664	54.0745000	50.2176949	49253.6	0.0573001	0.95	30.4869000	79.2640000
age.s[147]	1.0003147	1.0011545	34.2898622	32.8181000	29.9596497	50001.0	0.0413405	0.95	18.4118000	52.9323000
age.s[148]	1.0000310	1.0002365	49.9578694	48.1349000	45.7026980	50001.0	0.0820411	0.95	18.4932000	85.8284000
age.s[149]	1.0000598	1.0001513	73.3240145	73.8452000	74.7271754	50001.0	0.0597318	0.95	50.1777000	99.6545000
age.s[150]	0.9999748	0.9999886	69.3734647	69.2493000	69.4178949	48913.6	0.0562521	0.95	45.8823000	93.6996000

age.s[151]	1.0000314	1.0001205	63.5539650	63.1164000	62.2299772	50001.0	0.0635014	0.95	37.2915000	91.9222000
age.s[152]	0.9999449	0.9999581	58.7957984	59.0789000	60.9978077	50001.0	0.0829500	0.95	23.5073000	93.2562000
age.s[153]	1.0000741	1.0002879	42.0394914	40.8555000	38.5402765	50001.0	0.0451938	0.95	24.0839000	62.6035000
age.s[154]	0.9999788	0.9999983	50.6234560	49.0983000	45.7647121	49363.9	0.0794405	0.95	20.1282000	85.5395000
age.s[155]	1.0001435	1.0006391	51.6839059	50.4223000	46.3616561	50001.0	0.0679482	0.95	23.8463000	81.4430000
age.s[156]	0.9999615	0.9999994	34.2216263	31.6106000	19.5557373	49898.5	0.0629969	0.95	15.0019000	61.3204000
age.s[157]	1.0002432	1.0007686	52.8256921	51.4896000	47.8095755	50001.0	0.0663253	0.95	26.3225000	82.3742000
age.s[158]	1.0001557	1.0007179	61.9106513	61.1398000	58.3696675	50001.0	0.0549409	0.95	39.3117000	86.5961000
age.s[159]	1.0001716	1.0004438	46.1861881	44.7779000	41.8222322	49025.4	0.0534043	0.95	24.7327000	69.5237000
age.s[160]	1.0003320	1.0012954	80.6414496	81.7574000	83.4482177	46824.7	0.0529972	0.95	59.9564000	99.9857000
age.s[161]	1.0000217	1.0001604	50.1091576	48.5038000	37.5838070	49124.8	0.0830598	0.95	17.2444000	84.5796000
age.s[162]	1.0000553	1.0002274	77.8109284	78.0730000	77.3232827	48333.5	0.0500767	0.95	58.2789000	99.0566000
age.s[163]	1.0004012	1.0009611	36.7689221	34.9427000	31.0909824	49014.9	0.0485495	0.95	18.4706000	58.6994000
age.s[164]	0.9999769	1.0000570	59.1298343	58.4869000	56.0533192	50001.0	0.0663662	0.95	31.1768000	87.6431000
age.s[165]	1.0001253	1.0006119	49.3845319	48.2904000	45.8755567	50001.0	0.0487962	0.95	29.7078000	71.3993000
age.s[166]	1.0000015	1.0001096	43.1155118	41.4506000	38.9771459	50001.0	0.0578971	0.95	20.2604000	68.5471000
age.s[167]	0.9999914	1.0000244	33.9891711	29.6648000	18.3258020	50001.0	0.0718754	0.95	15.0001000	66.6564000
age.s[168]	1.0000190	1.0001432	41.4072467	40.2652000	38.1189659	50001.0	0.0454520	0.95	23.3260000	62.0393000
age.s[169]	1.0001275	1.0003216	62.3932580	61.7003000	59.9015348	49317.1	0.0560560	0.95	39.3222000	87.4742000
age.s[170]	1.0000573	1.0003538	58.4549459	57.8842000	57.6098082	50001.0	0.0676909	0.95	30.6098000	88.3672000
age.s[171]	1.0000622	1.0000774	46.4057083	45.0912000	42.1512646	50001.0	0.0521715	0.95	25.4380000	69.7737000
age.s[172]	1.0001341	1.0005308	35.7941916	34.2671000	31.7901737	50001.0	0.0413705	0.95	20.4313000	54.9931000
age.s[173]	1.0008509	1.0017308	27.9252511	26.4493000	23.5668372	47203.5	0.0362533	0.95	15.0039000	42.9167000
age.s[174]	1.0001904	1.0006347	40.9304148	39.2955000	36.4032396	50001.0	0.0596345	0.95	17.7419000	67.1387000
age.s[175]	1.0000323	1.0001061	63.4652459	63.0316000	61.6482108	49155.0	0.0638395	0.95	36.8286000	91.2026000
age.s[176]	1.0000522	1.0003283	55.9631185	54.9335000	51.6970343	52439.3	0.0573268	0.95	31.7897000	82.4840000
age.s[177]	0.9999904	1.0000327	35.4419851	33.9294000	30.0959131	50001.0	0.0498142	0.95	16.4552000	57.3190000
age.s[178]	1.0000434	1.0002890	38.5065509	37.1005000	33.3564192	50691.3	0.0476955	0.95	20.3010000	60.4241000
age.s[179]	0.9999691	1.0000133	49.4810008	48.1898000	43.8011758	50076.4	0.0561171	0.95	26.3356000	74.3205000
age.s[180]	1.0000515	1.0002732	50.0120057	48.3521000	41.3719067	50001.0	0.0820283	0.95	18.0419000	85.0909000

3.2 Arithmetic mean as point estimate of age-at-death

Table 31: Spitalfields. Goodness-of-fit measures for the whole data-set, mit arithmetic mean as point estimate.

	Gof measures
Bias	3.53
corrPearson	0.67
corr_p	0.00
Residual_age_slope	0.42
Inaccuracy	10.86
RMSE	13.77
TMNLP	4.42
CRPS	7.81
Coverage	92.22
HDI_Diff_median	48.42

For comparison with Figure 7 in the main text: Estimation of age-at-death with arithmetic mean as point estimate for the data-set Spitalfields with the multivariate normal model.

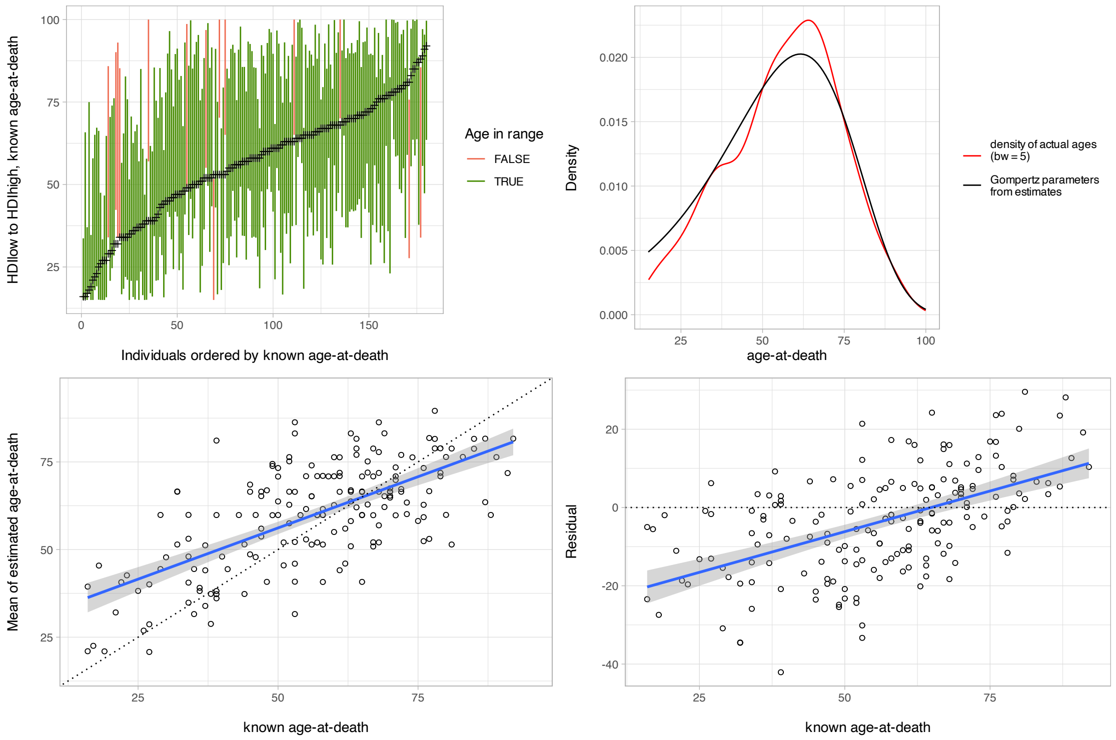

Figure 3: Estimation of age-at-death for data-set Spitalfields with the multivariate normal model. Left top – comparison between known age-at-death and the estimated 95%-HDIs, coloured green if the true age lies within the range and red if not; top right – density of known age-at-death and Gompertz function with estimated parameters; bottom left – known age-at-death vs. arithmetic mean of estimated age-at-death, the dotted line marks complete equivalence, regression line in blue; bottom right – known age-at-death vs. residuals (known age-at-death minus arithmetic mean of estimated age-at-death), the dotted line marks complete equivalence, regression line in blue.

3.3 Counts per trait and level with missing data

Tables for the count per trait and level of the Spitalfields dataset, calculated with an increasing number of missing data. Traits AP, MA and MI only have three levels each, therefore, the last two columns are

“NA” for them. For the samples with missing data, the last column “NA” specifies the number of missing entries per trait.

Table 32: Spitalfields. Count per trait and level for complete data-set.

name	1	2	3	4	5	NA
AP	39	118	23	NA	NA	NA
MA	92	58	30	NA	NA	NA
MI	13	31	136	NA	NA	NA
ST	4	18	100	54	4	NA
TO	3	27	31	96	23	NA

Table 33: Spitalfields. Count per trait and level for 20 % missing data.

name	1	2	3	4	5	NA
AP	26	92	18	NA	NA	44
MA	70	45	22	NA	NA	43
MI	10	27	110	NA	NA	33
ST	4	15	86	41	3	31
TO	3	24	23	81	20	29

Table 34: Spitalfields. Count per trait and level for 30 % missing data.

name	1	2	3	4	5	NA
AP	30	88	16	NA	NA	46
MA	64	48	16	NA	NA	52
MI	9	27	100	NA	NA	44
ST	4	11	67	36	2	60
TO	NA	19	21	58	22	60

Table 35: Spitalfields. Count per trait and level for 40 % missing data.

name	1	2	3	4	5	NA
AP	18	72	10	NA	NA	80
MA	50	34	23	NA	NA	73
MI	10	20	76	NA	NA	74
ST	1	12	69	34	1	63
TO	3	15	23	55	13	71

Table 36: Spitalfields. Count per trait and level for 50 % missing data.

name	1	2	3	4	5	NA
AP	23	61	10	NA	NA	86
MA	50	23	16	NA	NA	91
MI	5	11	67	NA	NA	97
ST	NA	14	57	31	2	76

TO	3	14	16	48	9	90

3.4 Exact Binomial Tests

Table 37: Exact binomial test for Spitalfields (20 % missing).

coverage	n_in	perc	CI_low	CI_up	p_value
0.10	15	0.083	0.047	0.134	0.535
0.20	32	0.178	0.125	0.242	0.514
0.30	52	0.289	0.224	0.361	0.807
0.40	62	0.344	0.275	0.419	0.148
0.50	75	0.417	0.344	0.492	0.030
0.60	88	0.489	0.414	0.564	0.003
0.70	100	0.556	0.480	0.629	0.000
0.80	120	0.667	0.593	0.735	0.000
0.90	138	0.767	0.698	0.826	0.000
0.95	160	0.889	0.834	0.931	0.001

Table 38: Exact binomial test for Spitalfields (30 % missing).

coverage	n_in	perc	CI_low	CI_up	p_value
0.10	10	0.056	0.027	0.100	0.046
0.20	25	0.139	0.092	0.198	0.040
0.30	40	0.222	0.164	0.290	0.023
0.40	58	0.322	0.255	0.396	0.033
0.50	73	0.406	0.333	0.481	0.014
0.60	90	0.500	0.425	0.575	0.008
0.70	111	0.617	0.541	0.688	0.018
0.80	130	0.722	0.651	0.786	0.012
0.90	151	0.839	0.777	0.889	0.012
0.95	160	0.889	0.834	0.931	0.001

Table 39: Exact binomial test for Spitalfields (40 % missing).

coverage	n_in	perc	CI_low	CI_up	p_value
0.10	16	0.089	0.052	0.140	0.710
0.20	33	0.183	0.130	0.248	0.642
0.30	45	0.250	0.189	0.320	0.166
0.40	57	0.317	0.249	0.390	0.022
0.50	79	0.439	0.365	0.515	0.117
0.60	99	0.550	0.474	0.624	0.172
0.70	126	0.700	0.627	0.766	1.000
0.80	141	0.783	0.716	0.841	0.576
0.90	160	0.889	0.834	0.931	0.618
0.95	170	0.944	0.900	0.973	0.730

Table 40: Exact binomial test for Spitalfields (50 % missing).

coverage	n_in	perc	CI_low	CI_up	p_value
0.10	7	0.039	0.016	0.078	0.004

0.20	16	0.089	0.052	0.140	0.000
0.30	27	0.150	0.101	0.211	0.000
0.40	39	0.217	0.159	0.284	0.000
0.50	42	0.233	0.174	0.302	0.000
0.60	58	0.322	0.255	0.396	0.000
0.70	72	0.400	0.328	0.476	0.000
0.80	93	0.517	0.441	0.592	0.000
0.90	116	0.644	0.570	0.714	0.000
0.95	133	0.739	0.668	0.801	0.000

References

Brooks, S., & Suchey, J. M. (1990). Skeletal age determination based on the os pubis: A comparison of the Acsádi-Nemeskéri and Suchey-Brooks methods. *Human Evolution*, *5*(3), 227–238. https://doi.org/10.1007/BF02437238

Buckberry, J. L., & Chamberlain, A. T. (2002). Age estimation from the auricular surface of the ilium: A revised method. *American Journal of Physical Anthropology*, *119*(3), 231–239. https://doi.org/10.1002/ajpa.10130

Godde, K., & Hens, S. M. (2025). A Bayesian approach to Suchey-Brooks age estimation from the pubic symphysis using modern American samples. *Journal of Forensic Sciences*, *70*(1), 9–18. https://doi.org/10.1111/1556-4029.15651

Jeliazkov, I., Graves, J., & Kutzbach, M. (2008). Fitting and comparison of models for multivariate ordinal outcomes. In S. Chib, W. Griffiths, G. Koop, & D. Terrell (Eds.), *Bayesian econometrics*: *Vol. None* (None, pp. 115–156). Emerald Group Publishing Limited. https://doi.org/10.1016/S0731-9053(08)23004-5

Konigsberg, L. W., & Herrmann, N. P. (2002). Markov chain Monte Carlo estimation of hazard model parameters in paleodemography. In R. D. Hoppa & J. W. Vaupel (Eds.), *Paleodemography: Age Distributions from Skeletal Samples* (pp. 222–242). Cambridge: Cambridge University Press.

Konigsberg, L. W., Herrmann, N. P., Wescott, D. J., & Kimmerle, E. H. (2008). Estimation and Evidence in Forensic Anthropology: Age-at-Death. *Journal of Forensic Sciences*, *53*(3), 541–557. https://doi.org/10.1111/j.1556-4029.2008.00710.x

Kruschke, J. K. (2015). *Doing Bayesian data analysis: a tutorial with R, JAGS, and Stan* (pp. XII, 759). Amsterdam: Academic Press.

Lemoine, N. P. (2019). Moving beyond noninformative priors: why and how to choose weakly informative priors in Bayesian analyses. *Oikos*, *128*(7), 912–928. https://doi.org/10.1111/oik.05985

Lewandowski, D., Kurowicka, D., & Joe, H. (2009). Generating random correlation matrices based on vines and extended onion method. *Journal of Multivariate Analysis*, *100*(9), 1989–2001. https://doi.org/10.1016/j.jmva.2009.04.008

Merkle, E. C., & Rosseel, Y. (2018). blavaan: Bayesian structural equation models via parameter expansion. *Journal of Statistical Software*, *85*(4), 1–30. https://doi.org/10.18637/jss.v085.i04

Navega, D., Costa, E., & Cunha, E. (2022). Adult Skeletal Age-at-Death Estimation through Deep Random Neural Networks: A New Method and Its Computational Analysis. *Biology*, *11*(4). https://doi.org/10.3390/biology11040532

Plummer, M. (2003). JAGS: A Program for Analysis of Bayesian Graphical Models Using Gibbs Sampling. In K. Hornik, F. Leisch, & A. Zeileis (Eds.), *Proceedings of the 3rd International Workshop on Distributed Statistical Computing (DSC 2003), Vienna, 20-22 March 2003* (pp. 1–10). Wien: Technische Universität Wien.

Riecke, T. V., Sedinger, B. S., Williams, P. J., Leach, A. G., & Sedinger, J. S. (2019). Estimating correlations among demographic parameters in population models. *Ecology and Evolution*, *9*(23), 13521–13531. https://doi.org/10.1002/ece3.5809

Schuurman, N. K., Grasman, R. P. P. P., & Hamaker, E. L. (2016). A comparison of Inverse-Wishart prior specifications for covariance matrices in multilevel autoregressive nodels. *Multivariate Behavioral Research*, *51*(2-3), 185–206. https://doi.org/10.1080/00273171.2015.1065398
